# Cell‐Based Optimization of Covalent Reversible Ketoamide Inhibitors Bridging the Unprimed to the Primed Site of the Proteasome β5 Subunit

**DOI:** 10.1002/cmdc.201900472

**Published:** 2019-11-12

**Authors:** Daniel Stubba, Dennis Bensinger, Janika Steinbacher, Lilia Proskurjakov, Álvaro Salcedo Gómez, Uwe Schmidt, Stefan Roth, Katja Schmitz, Boris Schmidt

**Affiliations:** ^1^ Clemens-Schoepf-Institute for Organic Chemistry & Biochemistry Technische Universität Darmstadt Alarich-Weiss-Str. 4 64287 Darmstadt Germany; ^2^ Visual Inference Lab, Department of Computer Science Technische Universität Darmstadt Huchschulstr. 10 64289 Darmstadt Germany

**Keywords:** 20 S proteasome, α-ketoamides, cancer, drug discovery, ubiquitin

## Abstract

The ubiquitin‐proteasome system (UPS) is an established therapeutic target for approved drugs to treat selected hematologic malignancies. While drug discovery targeting the UPS focuses on irreversibly binding epoxyketones and slowly‐reversibly binding boronates, optimization of novel covalent‐reversibly binding warheads remains largely unattended. We previously reported α‐ketoamides to be a promising reversible lead motif, yet the cytotoxic activity required further optimization. This work focuses on the lead optimization of phenoxy‐substituted α‐ketoamides combining the structure‐activity relationships from the primed and the non‐primed site of the proteasome β5 subunit. Our optimization strategy is accompanied by molecular modeling, suggesting occupation of P1′ by a 3‐phenoxy group to increase β5 inhibition and cytotoxic activity in leukemia cell lines. Key compounds were further profiled for time‐dependent inhibition of cellular substrate conversion. Furthermore, the α‐ketoamide lead structure **27** does not affect escape response behavior in *Danio rerio* embryos, in contrast to bortezomib, which suggests increased target specificity.

## Graphical abstract

**Bridging the gap**: We report the identification of novel covalent reversible binding P1′‐site extended α‐ketoamide inhibitors for the proteasome β5 subunit. The development was guided by a new in‐cell proteasome inhibition assay and the lead compound was profiled in a *Danio rerio* embryo escape response assay showing superior properties in comparison to bortezomib. BODIPY conjugated activity‐based probes give insight into in‐cell localization and distribution in zebrafish embryos.

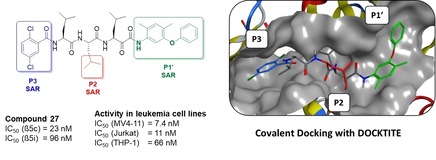

## Introduction

The Ubiquitin‐proteasome‐system (UPS) is the main non‐lysosomal proteolytic pathway for the degradation of misfolded, altered or short‐lived proteins in eukaryotes. It exerts a crucial role in cellular protein turnover e. g. the production of building blocks for the *de novo* synthesis of proteins and controls several cellular functions such as protein homeostasis, proliferation, apoptosis, signal transduction and antigen production.^1^ The ultimate proteolytic component of the UPS is the large, cylindric 26 S proteasome complex, consisting of two regulatory particles (19 S caps) and the 20 S proteasome core particle (CP), being responsible for the proteolysis of the designated proteins. The 20 S proteasome consists of four heptameric rings (α1‐7, β1‐7, β1‐7, α1‐7) bearing 28 subunits. Just three subunits per proteolytic β‐ring are catalytically active. The β1c subunit is referred to *caspase‐like activity*, the β2c to *trypsin‐like activity* and the β5c is found to show *chymotrypsin‐ and elastase‐like activity*.^2^, ^3^ Exposure to tumor necrosis factor α (TNFα) and/or interferon γ (IFNγ) leads to substitution of those subunits by β1i (LMP2), β2i (MECL‐1) and β5i (LMP7) resulting in formation of the immunoproteasome.^4^, ^5^ As a consequence of deviant substrate binding pockets in the β5 subunit, the iCP exerts different recognition and cleavage patterns. The major structural difference is an enlarged S1 pocket in β5i due to a different conformational orientation of Met45. Additional alterations in the amino acid sequence between cCP and iCP such as the thiol group of Cys48 pointing in the S4 pocket of the β5i subunit allow for selective targeting of the immunoproteasome.^6^, ^7^

**Figure 1:**
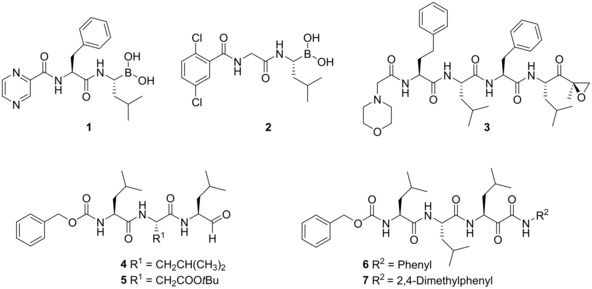
Overview of approved and experimental proteasome inhibitors. **1**: Bortezomib. **2**: Ixazomib. **3**: Carfilzomib. **4**: MG132. **5**: BSc2118. **6**: BSc2189. **7**: BSc4999.

Defects in the UPS affects cell cycle progression as well as immune response thus are recognized as an attractive target for several diseases such as cancer indicating that inhibitors of this pathway may prevent malignant cells from proliferation.^8^, ^9^, ^10^ Inhibition of the proteasome results in an imbalance between proteasome load and proteasome capacity that serves as a trigger for apoptosis^11^ as well as for inhibition of NFκB signaling by blocking the degradation of the NFκB‐inhibitor IκB preventing nuclear translocation of NFκB.^12^ Bortezomib (**1**) was the first proteasome inhibitor (PI) approved by the FDA in 2003 followed by carfilzomib (**3**) in 2012 and ixazomib (**2**) in 2015, the first orally available PI, for the treatment of multiple myeloma and mantle‐cell lymphoma (Figure 1).^7^ Multiple clinical trials are still in progress for the treatment of leukemia, non‐Hodgkin lymphoma and solid tumors. All approved PIs are covalently binding inhibitors, bearing an electrophilic warhead which predominantly binds to the catalytically active Thr1Oγ of the β5c subunit. The first‐generation PI bortezomib is a slowly reversible binding peptidic boronic acid inhibiting the β5 and to a less extent the β2 and β1 subunits of the immuno as well as the constitutive proteasome and numerous off‐target proteases.^13^ Insufficient selectivity frequently results in a major adverse event upon treatment with bortezomib: peripheral neuropathy, a dose limiting neurotoxic effect occurring in 30 % to 39 % of the patients.^14^ Unlike reversibly binding boronates carfilzomib bears an epoxyketone warhead that binds irreversibly to the hydroxy group of Thr1Oγ forming a morpholino ring with Thr1 N.^15^, ^16^ Recent crystal structure analysis suggests formation of a seven‐membered 1,4‐oxazepane ring due to a nucleophilic attack of Thr1 N on the β‐carbon atom of the epoxide instead of the α‐carbon atom.^17^ Irreversible inhibitors can overcome drug resistance to reversible inhibitors but are less likely to penetrate deep into tissue, resulting in impaired efficacy for solid, non‐vascularized tumors.^18^ Ixazomib is formulated as a citrate boronate prodrug improving distribution characteristics that allow oral uptake.^19^ It shows affinity to the β5 and β1 subunit comparable to bortezomib combined with a shorter residence time that may account for deeper tissue penetration.^18^ In general, irreversible as well as slowly‐reversible covalent inhibitors may provide unique advantages when forming long‐lived ties with their target. In contrast, highly reactive inhibitors are likely to be trapped by the high number of proteasomes in the red blood cells, which limits the therapeutic availability to non‐solid tumors. Besides the approved drugs several inhibitors bearing reversible warheads have been developed; the peptidic aldehydes MG132 (**4**), BSc2118 (**5**) and the peptidic α‐ketoamides BSc2189 (**6**) and BSc4999 (**7**).^20^, ^21^, ^22^

Our previous work highlighted the α‐ketoamide electrophile as a highly active reversible lead motif with potential for the treatment of solid tumors or autoimmune disorders.^21^, ^22^ However, the reduced cytotoxic activity of **6** in malignant cell lines (in comparison to carfilzomib) motivated us to optimize the potency of ketoamide proteasome inhibitors targeting the primed site of the β5 subunit.

## Results and Discussion

**Figure 2:**
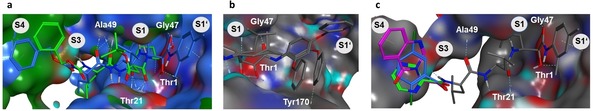
(a) Superposition of aldehyde **5** (green) and ketoamide **7** (blue). (b) Energy‐minimized models of 2‐, 3‐ and 4‐phenoxy substituted ketoamides **8–10** and (c) P4‐substituted tripeptides **22** (purple), **23** (green) and **24** (blue) targeting the β5c subunit.

Optimization of the ketoamide warhead started with the examination of the yeast CP cocrystal structures of aldehyde **4** (PDB: 4NNN) as well as ketoamides **6** (PDB: 4NO8) and **7** (PDB: 4R02) that were obtained by our previous optimization efforts (Figure 2a).^20^, ^21^, ^22^

**Scheme 1:**
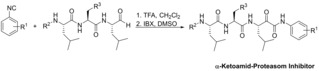
Synthesis outline for the development of phenoxy‐substituted tripeptidic ketoamides.

All three inhibitors bind to the β5 subunit and adopt similar conformations of the triple leucyl peptide residues as well as the Cbz‐protecting groups, located in P4, close to the β6 subunit. However, the dimethyl phenyl group of ketoamide **7** is slightly shifted (∼0.8 Å) towards the primed site when compared to the unsubstituted phenyl moiety in **6**. While the hydroxyl group of the hemiacetal adduct of **4** with Thr1Oγ is situated in the oxy anionic hole of this site, the hemiketal hydroxyl group of **6** and **7** is switched to the opposite site enabling the carbonyl oxygen to constrain the conformation of the phenyl amide head group. Peptidic phenyl ketoamides were synthesized using the Passerini reaction of functionalized isocyanides and peptidic aldehydes with trifluoro acetic acids to give α‐hydroxy amides that were oxidized using 2‐iodoxybenzoic acid to provide the final ketoamides (Scheme 1, SI chapter 1).^21^ Phenoxy‐substituted isocyanides were prepared starting from commercially available fluoro nitro benzenes that were reacted with phenol or by conversion of nitrophenols with diphenyl iodonium chloride. Subsequent reduction of the nitro group gave the corresponding anilines, followed by *N*‐formylation and subsequent dehydration leading to isocyanides that were subjected to the Passerini reaction. The peptidic aldehydes have been synthesized as reported previously.^20^, ^21^

Our first goal was to evaluate whether substitutions in the 2‐, 3‐ or 4‐position are beneficial to bridge the inhibitor to substrate pockets of the primed site. We decided to introduce a phenoxy group as a substituent that is sufficiently bulky to occupy S1’ but still flexible enough to allow an induced fit to the primed site. Compound **7** from the cocrystal structure served as starting point for the in silico analysis of analogues. The energy‐minimization of the virtual ligands revealed that all positions might tolerate such a substitution (Figure 2b). However, the cytotoxic activity of 3‐phenoxy ketoamide **9** in MV4‐11 leukemia cells is substantially higher in comparison to the 2‐ and 4‐phenoxy ketoamides **8** and **10**, which correlates with the increased inhibition of the proteasome. However, the inhibition of β5c by **9** is decreased (in comparison to 7) while inhibition of β5i is increased resulting in decreased selectivity β5c over β5i (2.6‐ to 4.3‐fold, Table 1). We therefore aimed to increase selectivity by re‐introducing the methyl groups in ortho or para position to the ketoamide NH.

**Table 1:** Structure–activity relationship of Cbz‐protected phenoxy‐substituted ketoamides **8**–**21** and previously reported ketoamide **7**.^22^

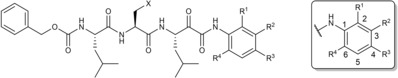								
Compound	X	R^1^	R^2^	R^3^	R^4^	IC_50_ MV4‐11 [nM]^[a]^	Rel. Inhibition (c =100 nM)	
							β5c	β5i
**Bortezomib 1**	–	–	–	–	–	34±5	89 %	85 %
**Carfilzomib 3**	–	–	–	–	–	3.6±1.8	86 %	43 %
**7**	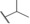	Me	H	Me	H	19±3	64 %	33 %
**8**		OPh	H	H	H	203±22	12 %	8 %
**9**		H	OPh	H	H	18±3	56 %	23 %
**10**		H	H	OPh	H	45±3	25 %	5 %
**11**		H	OPh	Me	Me	19±1	46 %	12 %
**12**		Me	OPh	Me	H	26±8	20 %	9 %
**13**		H	OPh	Me	H	23±2	49 %	28 %
**14**		Me	OPh	H	H	27±8	41 %	29 %
**15**		H	OPh	H	Me	16±1	26 %	19 %
**16**	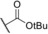	H	H	H	H	46±6	57 %	48 %
**17**		Me	H	Me	H	46±1	43 %	36 %
**18**		OPh	H	H	H	76±14	28 %	18 %
**19**		H	OPh	H	H	36±5	53 %	43 %
**20**		H	H	OPh	H	68±9	41 %	40 %
**21**		H	OPh	Me	Me	297±27	33 %	28 %

[a] Values are the mean±SD from two independent experiments, each carried out in technical triplicates.

Introduction of methyl groups results in the 1,2,3,4‐substituted phenyl ketoamide **12** and decreases cellular activity substantially whereas the 1,3,4,6‐substituted ketoamide **11** retains the cytotoxic activity of **9**. Remarkably, the selectivity is now shifted towards the inhibition of β5c over β5i. Mono‐methyl substitution in the 2‐ or 4‐position decreases cytotoxic activity (**13** and **14**) as well as selectivity over β5i. 6‐Methyl substitution leading to ketoamide **15** decreases inhibition of β5c and β5i while cytotoxicity is slightly increased. In previous work, the occupation of P2 by AspOtBu increased the selective inhibition of β5c by peptidic aldehydes.^20^ However, as proteasomes miss a defined P2 pocket, the increased activity might be due to hydrophobic interactions correlating with increased molecular weight and resulting in the displacement of active site water molecules.

As the cytotoxic activity of **5** (Asp(OtBu) in P2) is slightly higher than of **4** (Leu in P2) correlating with higher β5c inhibition while selectivity over β5i is retained, we investigated if this pattern can be also applied to the ketoamide warhead. Subsequent synthesis of ketoamides **16**–**21** began with aldehyde **5**. In general, the cytotoxicity of all P2=Asp(OtBu) derivatives in this series is lower than the one for all compounds featuring leucine in P2. Interestingly, the dimethyl‐substituted ketoamide **17** shows similar cytotoxic activity like the unsubstituted ketoamide **16** in MV4‐11 cells. The trend in inhibition by phenoxy‐substituted inhibitors featuring leucine in P2 can also be observed for the AspOtBu derivatives **18**–**20**, where m‐phenoxy ketoamide **19** is more potent than o‐ and p‐substituted phenoxy ketoamides **18** and **20**. While **18** and **20** are more potent inhibitors of β5c than their leucyl counterparts, inhibition by **19** is comparable and the highest in this series.

However, inhibition of β5i is increased relatively to β5c by m‐phenoxy ketoamide **19** leading to isoform unselective inhibition. In strong contrast to **11**, it was important to see that re‐introduction of the 4,6‐dimethyl group giving the tetra substituted ketoamide **21** results in decreased cytotoxic activity as well as in β5i and β5c inhibition compared to **19**.

A possible reason for the opposing activity trend by AspOtBu‐substituted ketoamides and aldehydes might be due to a slight shift in the positioning of the amide backbone in aldehydes **4** and **5** compared to ketoamides as visible in reported cocrystals. The phenyl amide group hinders close vicinity of the prochiral carbon to Thr1 leading to a changed induced fit of the ligand and possible unfavorable placement of the bulky *t*Bu residue in P2.

The next optimization step was the replacement of the Cbz protecting group occupying P4 of the β5c subunit. We hypothesized that fewer rotatable bonds as well as additional polar interactions might improve potency and cell permeability and so allow alternative induced fit mechanisms to the active site. We decided to introduce three different P4 groups that are inspired by previously reported inhibitors (R^1^–R^3^, Table 2). Ixazomib **2** harbors a dichloro benzamide residue (R^3^) following P2, comparable to the pyrazine‐2‐amide (R^2^) in bortezomib **1** but might act sterically and electronically distinct. Furthermore, we aimed to probe if a bicyclic ring is tolerated and introduced a quinazolinone group, that is linked to P3 leucine by an acetyl group, similar to an approach reported by Micale et al (R^1^).^23^ To explore the plausibility of these substitutions, we modified **7** using the cocrystal structure with subsequent energy minimization of the ligand (Figure 2c). All three residues are able to fit into the S4 pocket close to the β6 subunit, similar to the carboxybenzyl group, and form hydrogen bond interactions by the P4 amide NH with the Asp126 side chain of β6. Additionally, R^1^ might form π‐H interactions with Pro127 of β6, while R^2^ might form two π‐H interactions with Val128. Exchange of the Cbz‐protecting group to amides was performed by catalytic hydrogenation to give peptidic amino alcohols. These have been converted to the corresponding amides using standard peptide coupling methods with the functionalized acids and subsequent oxidation of the hydroxyl group using IBX gave aldehydes that served as the second starting material for the Passerini reaction.

**Table 2:** Structure−activity relationship of aldehydes **4**, **5** and **22− 25** and dichloro benzamide‐substituted m‐phenoxy ketoamides **26**–**29**.

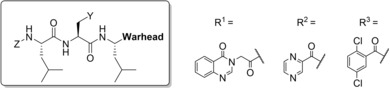							
Compound	Warhead	Y	Z	R^4^	IC_50_ MV4‐11 [nM]^[a]^	Rel. Inhibition (*c*=100 nM)	
						β5c	β5i
**4**	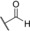	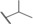	Cbz	–	131±12	43 %	6 %
**22**			R^1^	–	>1000	6 %	11 %
**23**			R^2^	–	145±23	30 %	4 %
**24**			R^3^	–	16±1	76 %	60 %
**5**		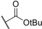	Cbz	–	123±12	54 %	11 %
**25**			R^3^	–	28±5	64 %	38 %
**26**	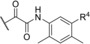	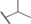	R^3^	H	25±5	25 %	4 %
**27**			R^3^	OPh	7.4±2.6	83 %	58 %
**28**		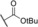	R^3^	H	47±5	26 %	35 %
**29**			R^3^	OPh	58±10	26 %	8 %

[a] Values are the mean±SD from two independent experiments, each carried out in technical triplicates.

The dichloro benzamide‐substituted aldehyde **24** exerts strongly increased cytotoxic activity in comparison to **4**, while the bicyclic derivative **22** is inactive. The pyrazinyl moiety in **23** provides no improvement in cytotoxicity. Introduction of Asp(OtBu) in P2 resulted in the R^3^‐substituted aldehyde **25**, which leads to significantly increased cytotoxicity in comparison to the Cbz‐protected aldehyde **5**, but remains less potent than the leucyl counterpart similar to the ketoamide series.

Then we combined our SAR efforts exploring phenoxy‐substituted ketoamides and P4 variation to give hybrid ketoamides **26**–**29**. In line with the SAR described so far, 3‐phenoxy‐4,6‐dimethylphenyl ketoamide **27** resulted in the most potent compound in this series, displaying the highest cytotoxic activity and inhibition of β5c (IC_50_=23 nM, Table 3) as well as selectivity over β5i (IC_50_=96 nM, 4.2‐fold). Combination of the dimethyl phenyl ketoamide with the dichloro benzamide group in P4 provides no benefit in cytotoxicity. Furthermore substitution of leucine in P2 with Asp(OtBu) (**28**) decreases cytotoxic activity favoring inhibition of β5i. Introduction of the m‐phenoxy group resulting in the Asp(OtBu)‐analogue of **27** decreases cytotoxic activity even more. For all compounds the inhibition of β2c and β1c is low at 1 μM of inhibitor while they strongly inhibit β5c (Table S1).

**Table 3:** Comparison of cytotoxicity in leukemia cell lines and inhibition of β5i and β5c by lead compounds **7**, **9** and **27** as well as carfilzomib **3**.

Compound	Cell viability IC_50_ [nM]^[a]^			IC_50_ [nM]		Fold‐IC_50_
	MV4‐11	THP‐1	Jurkat	β5c	β5i	β5i/β5c
3	3.6±1.8	135±39	14±3	8	142	17.8
27	7.4±2.6	66±1	11±1	23	96	4.2
9	18±3	30±10	24±4	66	170	2.6
7	19±3	24±0.1	30±0.3	48	204	4.3

[a] Values are the mean±SD from two independent experiments, each carried out in technical triplicates.

### Covalent Docking of BSc4999 (7) and Lead Compound 27

Due to the surprising advantage of the m‐phenoxy substituted dichloro benzamide ketoamide **27** which our initial binding model could not show any explanation for we challenged our original binding hypothesis using covalent docking to explain increased cellular and proteasomal activity of **27**. During the last decade extensive approaches of the computational supported drug discovery have been applied for the modelling of proteasome inhibitors.^24^ These include docking of dipeptidyl boronates starting from known conformations similar to bortezomib^25^ as well as non‐peptidic boronates.^26^ However, true *de novo* docking of more complex peptides as tri‐ and tetra peptides remains rare as ligand flexibility, size and solvent‐exposed binding pockets increase computational time exponentially and generate a large number of possible poses. Therefore most approaches use simplifications. Examples include covalent docking of peptidyl ketoamides targeting the HCV serine protease in which the common peptidic core needs to be constraint;^27^ as well as docking approaches in which the covalent attachment point is not included in modelling^28^ or only the non‐peptidic warhead is modelled.^22^

In 2014, our group reported the development of DOCKTITE, a covalent docking workflow, that closed this knowledge gap and allowed precise redocking of proteasome inhibitors such as the tripeptide vinyl sulfonamides LU‐122.^29^

In enhancement of this methodology we performed *de novo* covalent docking of tripeptidic phenyl ketoamides simultaneously targeting the primed and unprimed site of the β5 subunit. Covalent redocking of **7** was successful using the conformation extracted from the yCP cocrystal structure as input conformation for docking only changing the hemiketal to the parental ketoamide warhead (PDB: 4r02, top‐scored pose RMSD=1.3 Å) which is similar to redocking of vinyl sulfonamide LU‐122 (PDB: 4int).^29^ However this approach is not straightforward for prospective docking of new ligands with unknown bound conformations. This is due to the fact that only random conformations can be chosen as input. To illustrate this issue, we performed unpretentious redocking of **7** using a random, energy‐minimized input conformation that only gave high root‐mean‐square deviation (RMSD) poses (no pose <2.0 Å, top‐scored RMSD=5.2 Å; top‐RMSD pose on scoring rank 17, see SI chapter 3 for details). This probably happens as LU‐122 contains polar amino acids engaging in hydrogen bonds with β5 residues, that are energetically favored by scoring functions compared to simple hydrophobic interactions. Occurrence of three leucyl residues in P1–P3 in **7** as well as the lead compound **27** increases ligand size as well as the number of rotatable bonds, thus hampering reliable modeling.

We therefore applied a novel screening strategy to address this problem, that is, in principle, customizable to other scaffolds and protein targets.

**Figure 3:**
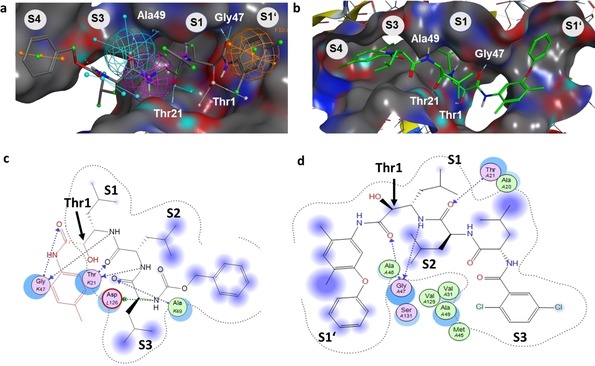
(a) Pharmacophore features of **7** used for covalent docking. (b) Top‐scored binding pose of compound **27**. (c) Ligand interaction map of compound **7** and (d) compound **27**.

In contrast to redocking of native poses, where the conformation obtained from the cocrystal structure is used as input, conformational sampling of manually sketched structures prior to docking is necessary as no definite starting points are available. We therefore performed a stochastic conformational search, subsequently tagged the ketoamide warhead and performed ligand attachment. For redocking of **7**, 624 conformations have been obtained that gave 1248 input conformations after sidechain attachment as the warhead is prochiral creating *R* and *S* enantiomeric adducts with Thr1Oγ. An automated pharmacophore defining the heavy chain atoms of Thr1 was generated (d=0.4 Å) during the side‐chain attachment step of DOCKTITE. In order to constrain docking to the substrate binding channel we introduced an aromatic pharmacophore feature as well as a donor and an acceptor feature, defined by the amide bond between P2 and P3 of **7** (each d=2.0 Å, Figure 3a, c). Then up to 5.000 conformations were sampled again from each input conformation during pharmacophore placement prior to energy‐minimization steps. MOE gave approximately 6 million conformations that were screened based on the defined pharmacophore features. From all docking conformations of one input conformation fitting the pharmacophore, up to 100 top‐scored poses were subjected to forcefield refinement. Using this strategy, 9.625 poses resulted from covalent docking that were subsequently cleaved from the nucleophile and rescored using the knowledge‐based DSX scoring function.^30^ Among the top 1 % DSX‐scored poses, 7 poses had a RMSD value of less than 2 Å compared to the native structure. Interestingly, between 0 and 30 docking poses resulted per input conformation (mean: 7.7 poses), suggesting that the conformational pre‐sampling of the tripeptides increased the success rates of covalent docking.

**Figure 4:**
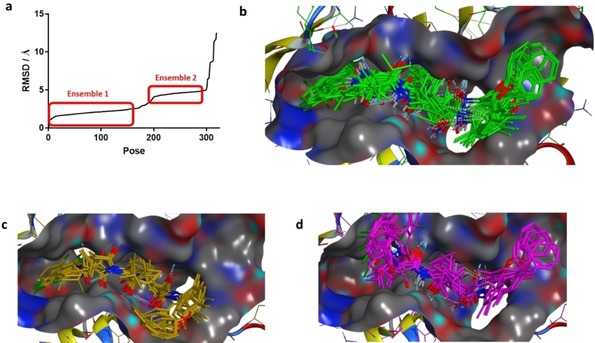
(a) Pose distribution sorted by increasing RMSD to top DSX‐scored pose of **27**. (b) Random selection of poses for ensemble 1 (pose 1 to 158; RMSD<2.5 Å) and the subgroups of ensemble 2 comprised of poses 201–297 (4.14 Å–4.95 Å) consisting of (c) group 1 (51 of 97 poses) and (d) group 2 (46 of 97 poses).

This method was applied to *de novo* docking of the lead compound **27** in the β5 subunit using the pharmacophore model created before. Stochastic conformational search showed 343 unbound conformations of **27** that were subsequently tagged and attached to the side chain giving 686 input conformations for covalent docking. Approximately 32.000 poses resulted and were cleaved again from the nucleophile and rescored by DSX. The top1 % of poses (320, DSX score<−200) were analyzed for RMSD values to the top‐scored pose (Figure 3b, d), giving two ensembles of poses (Figure 4a).

Ensemble 1 gave 158 poses with RMSD values smaller than 2.5 Å compared to the top‐scored pose of **27** (Figure 4b). In this group, the dichloro benzamide tail is situated in S3 instead of leucine. This was already observed in the reported cocrystal structures of **6** and **7** (Figure 2a).

This might be of advantage since entropy is increased by displacement of three water molecules due to the size of this group. As the dimethyl phenyl group of **27** obtains a similar orientation as in the cocrystal structure of **7**, the phenoxy group is allowed to reside in the S1’ pocket. Superposition of the top‐scored pose with the cocrystal structure of bis‐benzyl protected homobelactosin c (PDB: 4j70) shows similar occupation of the phenoxy group in S1’ as the benzyl protecting group in homobelactosin c.

The occupation of S1 and S2 by the leucine residues agrees well in comparison to ensemble 2 (RMSD‐ranked pose 201–297). However, two distinct pose clusters can be distinguished further. In cluster one (51 poses, Figure 4c) 180° rotation around the phenyl‐nitrogen bond leads to reorientation of the phenoxy group to the opposite direction of S1’ while binding of the dichloro benzamide group in S3 is changed in group two (46 poses, Figure 4d).

Ensemble 1 comprises ∼3‐fold more poses as the two subgroups in ensemble 2, furthermore the phenyl groups of **27** and homobelactosin C display similar binding in S1’, and so this model was favored for further structure‐based optimization.

The R‐configuration dominates the stereochemistry of adduct formation in ensemble 1 of **27** (77 %). This fact is observed for **7** in the cocrystal structure. This preference is decreased regarding the remaining top‐scored poses including ensemble 2 (62 % R). Interestingly, while aldehydes are attacked by Thr1Oγ to give the hemi‐acetal hydroxyl group residing in the oxyanion hole, ketoamides are attacked while the amide carbonyl group resides in the oxyanion hole having the hemiketal hydroxide in the opposite direction, which was confirmed by our docking model. Noteworthy, there is no correlation in pose scoring (DSX and London dG) to the RMSD to the native pose, suggesting that this conformational clustering approach applied here can obtain a clearer picture than scoring alone. However, the ultimate proof of the binding mode of **27** reside in proteasome crystallography.

### Cellular Inhibition of Proteasome Activity and Time‐Dependent Inhibition of Cell Viability

**Figure 5:**
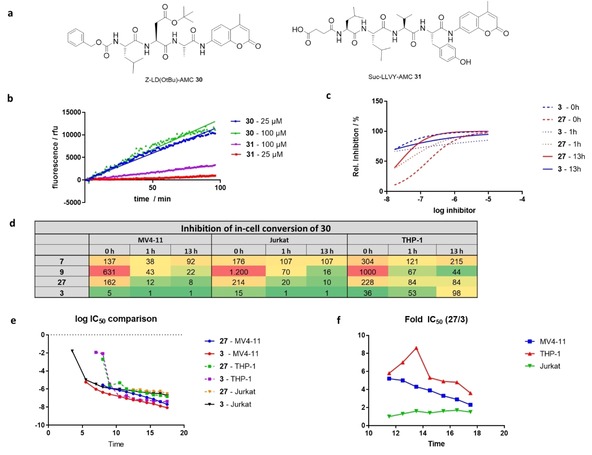
(a) Structure of proteasome substrates **30** and **31**. (b) Comparison of substrate conversion by MV4‐11 cells (50.000 cells/well). (c) Inhibition of substrate **30** conversion displays increased time‐dependency by ketoamide inhibitor **27** compared to epoxyketone **3**. (d) Overview of time‐dependent inhibition of substrate **30** conversion by **7**, **9**, **27** and **3** in different leukemia cell lines. (e) Comparison of time‐dependent cytotoxicity by compounds **27** and **3** and (f) relative cytotoxic activity (Fold‐IC_50_=$\frac{{IC}_{50}27}{{IC}_{50}\;3}$ ; Table S2).

In 2016, our group reported the development of subunit‐specific, fluorogenic proteasome substrate **30**, benefiting from an AspOtBu residue in P2 (Z‐LD(OtBu)A‐AMC, Figure 5a). This indicates increased selectivity and catalytic efficiency for small P1 residues, such as alanine, while inhibition performance shows the opposite trend, favoring spacious P1 residues.^2^ In this work, we want to apply this methodology for the determination of intracellular proteasome inhibition among different cell lines.

First, we benchmarked the fluorogenic substrate **30** towards the standard substrate **31** (Suc‐LLVY‐AMC) using varying substrate concentrations and cell numbers of MV4‐11 cells (Figure 5b). The highest substrate conversion was detected using 100 μM **30** in 50.000 cells (135 rfu/min) in MV4‐11 cells and was converted ∼4 times faster than **31** (33.3 rfu/min). There was an increase of differences in conversion rates with smaller number of cells (10.000 cells, 100 μM substrate: 10‐fold conversion) and lower substrate concentration (50.000 cells, 25 μM substrate: 15‐fold conversion).

Interestingly, while THP‐1 cells show similar turnover of **30** as MV4‐11 cells, conversion in Jurkat cells is ∼2‐fold increased. Then we used this optimized cellular proteasome activity assay to determine proteasome inhibition with our benchmark inhibitors **7**, **9**, **27** as well as reference compound carfilzomib **3**, using different pre‐incubation times (0 h–13 h) of inhibitor with cells, prior to addition of substrate conversion and fluorescence detection (Figure 5c, d).

In MV4‐11 cells, **3** shows a rapid, almost complete inhibition of substrate conversion in the single‐digit nanomolar range. This happens even though substrate and inhibitor are added at the same time and incubated for only 30 min, prior to fluorescence detection. Inhibition of cellular conversion is even more pronounced for 1 h and 13 h pre‐incubation. Compound **3** is characterized by a fast onset of inhibition by **3**, whereas ketoamides show a slower onset of inhibition increasing with incubation time with regard to phenoxy‐substituted ketoamides **9** and **27**, and decreasing at 13 h for **7** only. In line with the cytotoxic activity, **27** is more potent than **9** and **7** in MV4‐11 cells after 13 h pre‐incubation. Interestingly, **7** is 4‐fold more potent than **9** without pre‐incubation of inhibitor and cells, suggesting that the on‐rate of inhibitor binding is decreased or cell permeability of **9** is slower. A similar trend can be observed for the inhibition of proteasome substrate conversion in Jurkat cells. Carfilzomib **3** is the most potent compound displaying fast onset and sustained inhibition at 13 h pre‐incubation time while the ketoamides show slower onset of inhibition highest for **27** and lower for **9** and **7**. However, in THP‐1 cells the inhibitors show altered potency in inhibiting conversion of **30**, presumably as iCP expression is higher than in MV4‐11 and Jurkat cells.^31^ After short incubation times **3** is the most potent compound, while inhibition is decreased with incubation time, leading to lower inhibition after 13 h incubation time for **9** and **27**. Inhibition by **9** and **27** increases with incubation time, while, in contrast to Jurkat and MV4‐11 cells **9** is more potent than **27** after 13 h pre‐incubation. **7** displays the same trend as in MV4‐11 cells being the least potent compound displaying increasing inhibition after 1 h compared to 0 h that is decreasing after 13 h incubation time.

We further investigated time‐dependent cytotoxicity comparing our lead compound **27** to carfilzomib **3** using a continuous luminescent cell viability assay over 18 h (Figure 5e, f). In line with the results for cytotoxicity determined after 72 h treatment of cell lines as well as cellular substrate conversion, **3** shows stronger cytotoxicity in MV4‐11 cells and comparable cytotoxicity in Jurkat cells having a faster onset of cytotoxic activity (Table S2). Remarkably, in THP‐1 cells the initial cytotoxicity is increasing for 3 compared to 27 until 14 h treatment (9‐fold) but is approaching to the end of the assay. As the 72‐h endpoint cytotoxicity assay shows increased cytotoxicity for **27** than for **3** we assume that this trend is continuing and reversing relative inhibition favoring **27**.

It was interesting to find that after 13 h incubation cytotoxicity is about 50 nM to 400 nM for **3** and 300 nM to 600 nM for **27** suggesting cell death becomes relevant to the cellular conversion assay after this time scale. This effect might lead to artifacts, for example by leading to competing proteolysis in the media and increasing substrate influx through disrupted membranes.

### Development of BODIPY‐Conjugated Ketoamide Inhibitors

**Figure 6:**
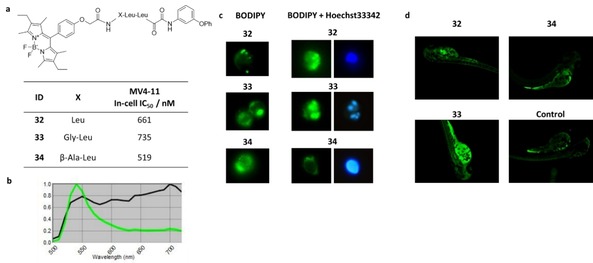
(a) Structure of BODIPY‐conjugated ketoamides **32**–**34** and inhibition of cellular substrate conversion of **30** in MV4‐11 cells. (b) Exemplary spectrum of BODIPY‐conjugated inhibitors analyzed using the Nuance Fx Imaging system on Zeiss AxioScope A.1. Fluorescence is measured using AF488 fluorescence filter sampling from 500 nm to 720 nm. Fluorescence intensity is normalized to highest fluorescence signal. (c) Staining of MV4‐11 cells with compounds **32**–**34** with or without Hoechst33342 DNA staining (2500 ms exposure time; Filter 365 nm or AF488, 40x magnification). (d) Uptake of fluorescent inhibitors **32**–**34** in Danio rerio embryos (3225.5 ms exposure time, Filter AF488, 5x magnification).

We further aimed to visualize distribution of ketoamide proteasome inhibitors in MV4‐11 cells and in *Danio rerio* embryos using BODIPY conjugations to **9** connected by different linking groups (Figure 6a). Synthesis was performed starting with the hydroxyamide precursor of **9** being deprotected by catalytic hydrogenation and coupled with acetyl, acetyl‐glycine or acetyl‐β‐alanine substituted BODIPYs that were subsequently oxidized using IBX to give ketoamides **32**–**34**. BODIPY‐conjugated ketoamides show similar inhibition of cellular conversion of substrate **30** (1 h incubation) as the parent compound **9** (IC_50_=631 nM) in MV4‐11 cells. The highly flexible β‐alanine linking group in **34** might facilitate ligand binding leading to most pronounced inhibition (IC_50_=519 nM). In MV4‐11 cells treated with **32**–**34** an emission spectrum similar to isolated BODIPY fluorescence detection can be recorded (Excitation maximum ∼540 nm, Figure 6b).

Localization of all BODIPYs is mainly observed in the cytoplasm, while the nucleus shows lower fluorescence (Figure 6c). Furthermore fluorescence is concentrated in clusters, which are most likely aggresomes consisting of misfolded polyubiquitinated proteins aggregating with proteasomes, which is reported to be a result of proteasome inhibition.^32^ These results are in line with previous reports of green^33^ as well as red‐shifted^34^ fluorescent derivatives of peptidic aldehyde **5** colocalizing with proteasome in aggresomes and allowing selective proteasome‐staining. The uptake of fluorescent ketoamides in *Danio rerio* embryos is most intense upon treatment with **33**, while **32** and **34** show only slightly increased fluorescence compared to control embryos (Figure 6d). Fluorescence is predominantly localized in the intestine and colon for **32** and **34** whereas **33** shows increased fluorescence in the yolk sac suggesting increased uptake.

### *Danio rerio* Neurotoxicity Assay

**Figure 7:**
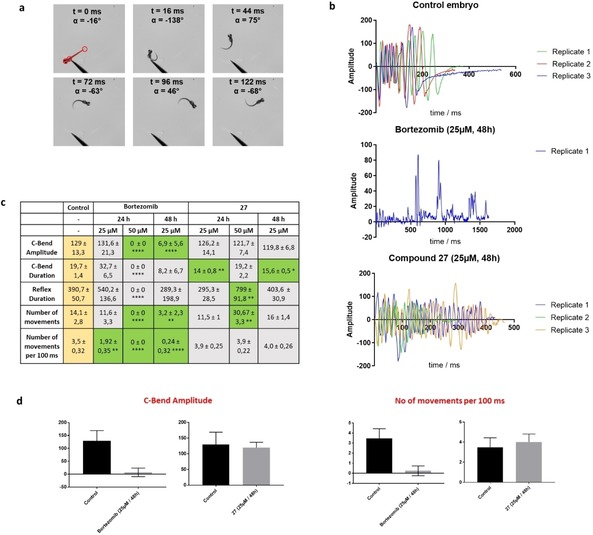
(a) Example for the touch‐evoked escape response of a control embryo and determination of body‐bend amplitude α. (b) Comparison of the body‐bend amplitude over time observed after treatment with bortezomib or **27** and control embryos after 48 h. (c) Analysis of treatment effects using different incubation parameters. Comparison of the C‐bend amplitude, C‐bend duration, reflex duration, number of movement and number of movements per 100 ms. Yellow: Kinematic parameters of control embryos. Grey: no statistically significant differences in mean compared to control. Green: Statistically significant differences of mean value. *=p<0.05. **=p<0.01. ***=p<0.001. ****=p<0.0001. n.s.=p>0.05. (d) Comparison of the C‐Bend amplitude in degrees and number of movements per 100 ms observed in bortezomib or **27** treated embryos after 48 h.

As the primary dose‐limiting side effect of bortezomib is peripheral neuropathy, we further characterized our lead compound **27** in a *Danio rerio* embryo escape response assay established in our group similar to reported approaches.^35^, ^36^ For the determination of neurotoxic effects embryos were dechorionated at 24 hpf and treated with two escalating doses of bortezomib **1** and ketoamide **27** (25 μM, 50 μM) for 24 h to 72 h. Subsequently, escape responses of surviving embryos were analyzed at 96 hpf evoked by touch stimulation and tracked with a high‐speed camera at 500 fps to allow quantification of embryo movement characteristics (Figure 7a).

Toxicity of bortezomib was high at 50 μM and 48 h to 72 h incubation time as well as 25 μM inhibitor and 72 h incubation time (100 % of bortezomib‐treated embryos dead) while embryos treated with **27** showed a higher survival rate (25 % to 75 %). However, overall survival in **27**‐treated embryos was lower than for bortezomib at low doses (92 % in bortezomib group, 58 % with **27**). In general, toxicity in treated, but still chorion‐protected embryos was low for both compounds (80 % to 100 % survival, Table S3). All control embryos, either dechorionated or in chorion were alive up to the end of the experiment. As we aimed to compare toxic effects between ketoamide **27** and bortezomib **1** to control, kinematic analysis was focused on treatment groups where at least 50 % of bortezomib‐ as well as **27**‐treated embryos survived (Tables S4, S5).

The characteristic escape response of control as well as unaffected treated embryos starts with a large body bend (C‐bend) pointing away from the stimulus followed by a counter bend for reorientation and the escape movement itself (Figure 7b, c). In control embryos, the mean C‐bend amplitude is 129±13,3° (3 embryos, 3 replicates of each embryos) and the C‐bend duration 19.7±1.4 ms. While mean C‐bend amplitude (131.6±21.3°) as well as duration (32.7±6.5 ms) is unchanged for 24 h/25 μM bortezomib‐treated embryos (each p>0.05) a huge decrease of C‐bend amplitude (6.9±5.6°, p<0.0001, Figure 7d) is observed in 48 h/25 μM bortezomib‐treated embryos, while mean C‐bend duration is not statistically significant changed but apparently highly variable (95 % CI=−26 to +3 ms compared to control). Interestingly, 24 h/50 μM bortezomib‐treated embryos show no response to touch stimuli (50 stimuli per embryo, 150 stimuli total, 0 responses). Therefore we decided to set body amplitudes to zero, describing an absent escape response (0 movements, p<0.0001, n=9). Accordingly, C‐bend amplitude (0±0°, p<0.0001) as well as C‐bend duration (0±0 ms, p<0.0001) are absent and thus massively decreased compared to control.

In contrast, **27**‐treated embryos showed similar escape responses to control. However, a slight decrease in C‐bend duration can be observed for 25 μM/24 h treated embryos (14±0.8 ms, p<0.01) as well as 25 μM/48 h treated embryos (15.6±0.5 ms, p<0.05).

We also observed a strong difference with regard to the touch responses of the other treatment groups, a hallmark of sensory neuropathy. In the control group, the number of mean reflexes per stimuli is 47 %. In bortezomib‐treated groups a strong decrease can be observed (8 % for 24 h incubation time, 2 % for 48 h; each 25 μM bortezomib) while an increase in touch responsiveness is observed for **27** (100 % for 24 h treated embryos with 25 μM and 50 μM) as well as a comparable responsiveness to control in 25 μM 48 h treated embryos (55 %).

Besides the C‐bend and touch responsiveness, the total duration of response as well as the total number of movements and ultimately, the ratio of number of movements per time is another important kinematic parameter. This parameter demonstrates a statistically significant decrease of movements per 100 ms in all bortezomib treated groups compared to control (3.5±0.32) which effects approximately half for 25 μM/24 h treated embryos (1.92±0.35, p<0.01). It is almost completely diminished for 25 μM/48 h (0.24±0.32, p<0.0001) as well as for non‐responding 50 μM/24 h treated embryos (0±0, p<0.0001). In strong contrast, mean values of movements per 100 ms for **27**‐treated embryos are not changed statistically significant for 25 μM at 24 h (3.9±0.25, p>0.05) as well as for 48 h incubation time (4.0±0.26, p>0.05). Interestingly, embryos treated with 50 μM **27** for 24 h showed an increased reflex duration (799±0.92 ms, p<0.01) as well as an increased number of movements (30.7±3.3, p<0.01) but a comparable movement relative to time (3.9±0.22, p>0.05).

These results suggest that **27** does not cause off‐target effects in vivo that lead to alterations in touch responsiveness or escape reflex behavior suggesting the assay as an additional preclinical safety screen in the development of proteasome and protease inhibitors.

## Conclusions

Despite covalent‐reversible inhibitors of the proteasome representing the first class of proteasome inhibitors, e. g. peptidic boronic acids and aldehydes, the drug development of reversible binding warheads has remained underexplored over the last decade, focusing instead on irreversible acting warheads such as epoxyketones and sulfonyl fluorides.^37^, ^38^, ^39^ Furthermore, explorations of structure–activity relationships of inhibitors targeting the primed site of β5 have been reported only occasionally. Our group previously reported the identification of ketoamide **7** that displays increased inhibitory activity compared to unsubstituted phenyl ketoamide **6** and allows for subsequent modification occupying the S1’ pocket.^20^, ^21^, ^22^

This work focuses on bridging the tripeptidic backbone in P1–P3 to the primed site P1′ pocket, increasing proteasome inhibition as well as cytotoxic activity in leukemia cell lines. Phenoxy substitutions in meta position to the phenyl ketoamide are most beneficial while reintroduction of the dimethyl group increases further proteasome inhibition. Remarkably, the occupation of P2 by AspOtBu instead of leucine is, in contrast to reversible peptidic aldehydes, not beneficial for proteasome inhibition. Inhibition by ketoamides increases strongly upon substitution of the carboxybenzyl group by dichloro benzamide in P4. Merging of the P1′‐, P2‐ and P4‐SAR yielded compound **27**, the most potent ketoamide inhibitor in this series. To our surprise, cytotoxic activity of the inhibitors investigated in this study varies strongly by cell line. Carfilzomib **3** is the most potent compound tested in the acute monocytic leukemia‐derived cell line MV4‐11, whereas **27** is more potent in Jurkat cells than **3**, a cell line derived from T‐cell leukemia. Interestingly, both inhibitors show much reduced activity in THP‐1 cells, also derived from acute monocytic leukemia expressing presumably iCP^31^ correlating with more potent inhibition of β5c over β5i by **3** and **27**. Furthermore, inhibition of cellular conversion of substrate **30** correlates with altered sensitivity in these cell lines. Our lead compound **27** was further assessed in an escape response assay using *Danio rerio* embryos. While bortezomib leads to a strongly impaired escape response and movement behavior in treated embryos, behavior in **27**‐treated embryos remains largely unchanged. This indicates that neurotoxicity resulting from off‐target inhibition such as Htra2/Omi, as observed for bortezomib **1**, is reduced.

Further work expanding the field of reversible covalent inhibition by targeting the primed site of proteasome subunits should focus on substitution of the amino acid side chains in P1 and P2 as well as substitution of the phenoxy group in P1′. As reported recently, small hydrophobic P1 residues such as alanine favor selectivity of binding to β5c rather than β5i while potency is decreasing.^40^ We therefore assume that selectivity to either β5i or β5c might be achieved most effectively if the non‐conserved residues in P1′ are targeted as well (β5c: Ser^116^, Glu^117^; β5i: Glu^116^, His^117^).

Altogether, the approach we refer to in this work uses iterative molecular modeling, synthesis and cellular profiling that highlight the use of covalent reversible inhibitors targeting the proteasome as effective as irreversible inhibitors enabling pharmacokinetic and pharmacodynamic altered drug development approaches in principle.

## Experimental Section

### Molecular Modeling

***Structural analysis of BSc4999 (8) and derivatization***. Molecular modeling was performed using the Molecular Operating Environment (Version 2016.0802, Chemical Computing Group). BSc4999 (**7**) in complex with the yeast proteasome was derived from the protein data bank (PDB: 4r02) and prepared using the LigX function without refinement (Figure S26–S34). Subunits β4 (chain L), β5 (chain K) and β6 (chain J) as well as ligands and waters in these subunits were isolated. Derivatization of the initial *N*‐terminal Cbz‐group as well as 2‐, 3‐, and 4‐phenoxy substitution of the phenyl ketoamide were built starting from the BSc4999‐CP complex and energy‐minimized while placed the CP β5 subunit using the Amber12:EHT forcefield.

***Covalent docking***. The lead compound **27** was docked using a methodology that was validated by docking of **7** (Figure S35–S38). Conformational search of compound **7** and **27** was performed using the built‐in conformational search method of MOE2016.0802.^41^ Conformations were sampled using the *stochastic* method with a rejection limit of 100; RMS gradient of 0.001, iteration limit of 1×10^6^ and MM iteration limit of 500. Amide bond rotation was not allowed and RMSD limit was set to 0.15. Conformations were then filtered for electrophilic warheads and tagged as reported in the standard protocol for using DOCKTITE.^29^ Thr1 was marked as covalent attachment point and ligands were attached to this nucleophile. A pharmacophore model of the nucleophile was generated automatically defining the position of Thr1 by elements (d=0.4 Å, 7 atoms). Thr1 as well as the neighboring amine in Thr2 were deleted to avoid steric clash during docking of sidechain‐attached ligands. The pharmacophore was enhanced by 3 features defined by inhibitor **7** placed in PDB: 4r02. Docking was performed using the pharmacophore placement method with prior conformational sampling of the input structure (up to 5000 conformations), pharmacophore filtering and refinement of up to 100 top‐scored poses (London dG scoring). Further refinement was performed using the GBVI/WSA dG scoring function (force constant 1e9). Docked poses were cleaved from the nucleophilic side chain and rescored using the DSX standalone version in Linux as described before.^29^

### Chemistry

**General remarks**. All reactions requiring anhydrous conditions were performed in dried glassware under argon atmosphere. All reagents and solvents were obtained from commercial suppliers without further purification. IBX, MG132 **4** and BSc2118 **5** were synthesized as described before.^20^, ^21^ NMR spectra were recorded with a Bruker AR 300 (300 MHz ^1^H and 75 MHz ^13^C) and a Bruker DRX 500 (500 MHz ^1^H, 126 MHz ^13^C, 471 MHz ^19^F and 160 MHz ^11^B). Deuterated solvents were used as internal standard. The δ values are reported in parts per million (ppm) downfield from TMS and were referenced to the residual solvent signal (CDCl_3_, DMSO‐*d_6_*). Coupling constants *J* are given in Hertz (Hz). The spectra were analyzed using MestReNova 11 (Mestrelab Research). ESI‐MS spectra were recorded on a Bruker Daltonics qTOF spectrometer. Ionization was achieved by an electron‐spray‐ionization source (ESI). HPLC was performed using an Agilent 1100 system with a Phenomenex synergi polar reversed phase column (4 μm particle size, 150×3.0 mm, pore size 80 Å) connected to a variable wavelength detector. The mobile phase consists of water/acetonitrile+0.1 % trifluoro acetic acid forming a linear gradient starting with 30 % water (held for 1 min) increased to 90 % acetonitrile within 10 min and held for 1 min with a constant flow of 1 mL/min. Chromatography was performed using flash chromatography of the indicated solvent system on 40–63 μm silica gel (VWR). Thin‐layer chromatography was carried out on 0.2 mm silica gel 60 plates (F‐254 Merck). They were detected by UV light (254 and 365 nm). All compounds used in biochemical assays have a purity of more than 95 % as determined by HPLC and are reported at the corresponding experiments.

**Synthesis of** α **‐ketophenylamides (GP i)**. The corresponding peptidic aldehyde (1.0 equiv) and aromatic isocyanides (1.5 equiv) were dissolved in a minimal amount of CH_2_Cl_2_ and cooled to 0 °C under an atmosphere of argon. A mixture of trifluoroacetic acid (2.0 equiv) in CH_2_Cl_2_ was added dropwise and the mixture was stirred for 2 h at 0 °C and 24 h at room temperature. Completion of the reaction was monitored by HPLC and TLC. CH_2_Cl_2_ was added and the organic layer was washed with 0.1 N aq. HCl (3x), sat. aq. NaHCO_3_ (3x) and sat. aq. NaCl. The organic extract was dried over Na_2_SO_4_ and the solvent was evaporated under reduced pressure. The resulting solid was dissolved in DMSO and IBX (2.0 equiv) was added. The reaction mixture was stirred 24 h at rt. After completion CH_2_Cl_2_ was added and the mixture was washed with water (3x), sat. aq. NaHCO_3_ (3x) and sat. aq. NaCl. The organic extract was dried over Na_2_SO_4_ and the solvent was evaporated under reduced pressure. Purification was done by column chromatography of the residue on silica gel (cyclohexane/AcOEt 2 : 1).

**Oxidation of alcohols via IBX (GP ii)**. The corresponding peptidic alcohol (1.0 equiv) was dissolved in DMSO and IBX (1.5 equiv) was added. The mixture was stirred overnight at rt. Completion of the reaction was monitored by HPLC and TLC. CH_2_Cl_2_ was added and the organic layer was washed with water (3x), sat. aq. NaHCO_3_ (3x) and sat. aq. NaCl. The organic extract was dried over Na_2_SO_4_ and the solvent was evaporated under reduced pressure.

**Peptide synthesis via HATU (GP iii)**. The carboxylic acid (1.0 equiv) was dissolved in DMF and HATU (1.1 equiv) was added. The mixture was stirred for 20 min. at rt before the amine (1.0 equiv) and DIPEA (2.9 equiv) were added. The reaction mixture was stirred overnight at rt. Completion of the reaction was monitored by HPLC and TLC. CH_2_Cl_2_ was added and the organic layer was washed with 0.1 N aq. HCl (5x), 0.1 N aq. NaOH (3x) and sat. aq. NaCl. The organic extract was dried over Na_2_SO_4_ and the solvent was evaporated under reduced pressure. If necessary, purification was done by column chromatography of the residue on silica gel.

**Benzyl‐((*S*)‐4‐methyl‐1‐(((*S*)‐4‐methyl‐1‐(((*S*)‐5‐methyl‐1,2‐dioxo‐1‐((2‐phenoxyphenyl)‐amino)hexan‐3‐yl)amino)‐1‐oxopentan‐2‐yl)amino)‐1‐oxopentan‐2‐yl)carbamate (8)** Cbz‐Leu‐Leu‐Leu‐al (338 mg, 0.711 mmol, 1.0 equiv), 1‐isocyano‐2‐phenoxybenzene (209 mg, 1.07 mmol, 1.5 equiv), trifluoroacetic acid (109 μL, 162 mg, 1.42 mmol, 2.0 equiv) and IBX (398 mg, 1.42 mmol, 2.0 equiv) were reacted according to GP i to afford 8 (184 mg, 0.268 mmol, 35 %) as a pale yellow amorphous solid. R_*f*_=0.10 (cyclohexane/AcOEt=5 : 1).

HPLC (254 nm, VWD): t_R_=9.06 min (98.03 %). ^1^H‐NMR (300 MHz, CDCl_3_): *δ*=9.32 (s, 1H), 8.52–8.44 (m, 1H), 7.41–7.28 (m, 7H), 7.19–6.98 (m, 6H), 6.88–6.81 (m, 1H), 6.64–6.44 (m, 1H), 5.52–5.39 (m, 1H), 5.39–5.24 (m, 1H), 5.14–5.01 (m, 2H), 4.58–4.43 (m, 1H), 4.25–4.08 (m, 1H), 1.81–1.60 (m, 6H), 1.55–1.45 (m, 3H), 1.02–0.84 (m, 18H). ^13^C‐NMR (75 MHz, CDCl_3_): *δ*=196.1, 172.5, 171.9, 171.7, 156.8, 156.1, 147.0, 136.1, 130.1, 128.7, 128.4, 128.2, 127.9, 125.4, 124.4, 123.8, 120.8, 119.3, 119.2, 117.5, 67.4, 54.2, 52.8, 51.6, 51.5, 41.4, 40.8, 40.6, 40.3, 40.2, 39.9, 27.1, 25.4, 24.9, 23.3, 23.0, 22.2, 22.0, 21.5. ESI‐MS: *m/z*=687.39 [M+H]^+^.

**Benzyl‐((*S*)‐4‐methyl‐1‐(((*S*)‐4‐methyl‐1‐(((*S*)‐5‐methyl‐1,2‐dioxo‐1‐((3‐phenoxyphenyl)‐amino)hexan‐3‐yl)amino)‐1‐oxopentan‐2‐yl)amino)‐1‐oxopentan‐2‐yl)carbamate (9)** Cbz‐Leu‐Leu‐Leu‐al (275 mg, 0.578 mmol, 1.0 equiv), 1‐isocyano‐3‐phenoxybenzene (170 mg, 0.871 mmol, 1.5 equiv), trifluoroacetic acid (89 μL, 132 mg, 1.16 mmol, 2.0 equiv) and IBX (325 mg, 1.16 mmol, 2.0 equiv) were reacted according to GP i to afford 9 (123 mg, 0.179 mmol, 29 %) as a pale yellow amorphous solid. R_*f*_=0.10 (cyclohexane/AcOEt=5 : 1). HPLC (205 nm, VWD): t_R_=9.91 min (97.83 %). ^1^H‐NMR (500 MHz, CDCl_3_): *δ*=8.64 (s, 1H), 7.39–7.27 (m, 10H), 7.15–7.11 (m, 1H), 7.03–7.00 (m, 2H), 6.82–6.76 (m, 2H), 6.47–6.41 (m, 1H), 5.35–5.30 (m, 1H), 5.17 (d, *J*=7.7 Hz, 1H), 5.11 (s, 2H), 4.49–4.42 (m, 1H), 4.21–4.14 (m, 1H), 1.80–1.56 (m, 6H), 1.55–1.46 (m, 3H), 1.01–0.86 (m, 18H). ^13^C‐NMR (126 MHz, CDCl_3_): *δ*=196.5, 172.5, 171.7, 158.2, 156.9, 137.7, 136.2, 130.4, 130.0, 128.8, 128.5, 128.2, 123.8, 119.3, 115.7, 114.7, 110.6, 67.4, 53.9, 53.0, 51.7, 41.3, 40.5, 40.2, 25.4, 24.9, 23.3, 23.1, 22.9, 22.2, 22.1, 21.5. ESI‐MS: *m/z*=687.39 [M+H]^+^.

**Benzyl‐((*S*)‐4‐methyl‐1‐(((*S*)‐4‐methyl‐1‐(((*S*)‐5‐methyl‐1,2‐dioxo‐1‐((4‐phenoxyphenyl)‐amino)hexan‐3‐yl)amino)‐1‐oxopentan‐2‐yl)amino)‐1‐oxopentan‐2‐yl)carbamate (10)** Cbz‐Leu‐Leu‐Leu‐al (275 mg, 0.578 mmol, 1.0 equiv), 1‐isocyano‐4‐phenoxybenzene (170 mg, 0.871 mmol, 1.5 equiv), trifluoroacetic acid (89 μL, 132 mg, 1.16 mmol, 2.0 equiv) and IBX (325 mg, 1.16 mmol, 2.0 equiv) were reacted according to GP i to afford 10 (106 mg, 0.154 mmol, 25 %) as a pale yellow amorphous solid. R_*f*_=0.11 (cyclohexane/AcOEt=5 : 1). HPLC (205 nm, VWD): t_R_=9.23 min (97.85 %). ^1^H‐NMR (500 MHz, CDCl_3_): *δ*=8.66 (s, 1H), 7.61–7.57 (m, 2H), 7.38–7.30 (m, 7H), 7.13–7.08 (m, 1H), 7.03–6.97 (m, 4H), 6.83–6.78 (m, 1H), 6.49–6.45 (m, 1H), 5.42–5.32 (m, 1H), 5.18 (d, *J*=7.7 Hz, 1H), 5.11 (s, 2H), 4.51–4.43 (m, 1H), 4.18 (br s, 1H), 1.81–1.59 (m, 6H), 1.56–1.45 (m, 3H), 1.03–0.83 (m, 18H). ^13^C‐NMR (126 MHz, CDCl_3_): *δ*=196.7, 172.5, 171.8, 157.3, 156.8, 154.7, 136.2, 131.7, 130.0, 128.8, 128.5, 128.2, 123.5, 121.7, 119.7, 118.9, 67.4, 53.1, 51.7, 41.3, 40.6, 40.3, 29.9, 25.4, 24.9, 23.4, 23.1, 22.9, 22.2, 22.1, 21.6. ESI‐MS: *m/z*=687.39 [M+H]^+^.

**Benzyl((*S*)‐1‐(((*S*)‐1‐(((*S*)‐1‐((2,4‐dimethyl‐5‐phenoxyphenyl)amino)‐5‐methyl‐1,2‐dioxo‐hexan‐3‐yl)amino)‐4‐methyl‐1‐oxopentan‐2‐yl)amino)‐4‐methyl‐1‐oxopentan‐2‐yl)carbama‐te (11)** Cbz‐Leu‐Leu‐Leu‐al (250 mg, 0.527 mmol, 1.0 equiv), 1‐isocyano‐2,4‐dimethyl‐5‐phenoxybenzene (176 mg, 0.788 mmol, 1.5 equiv), trifluoroacetic acid (81 μL, 120 mg, 1.05 mmol, 2.0 equiv) and IBX (295 mg, 1.05 mmol, 2.0 equiv) were reacted according to GP i to afford 11 (122 mg, 0.171 mmol, 32 %) as a pale yellow amorphous solid. R_*f*_=0.27 (cyclohexane/AcOEt=2 : 1). HPLC (254 nm, VWD): t_R_=9.13 min (97.81 %). ^1^H‐NMR (500 MHz, CDCl_3_): *δ*=8.58–8.54 (m, 1H), 7.75–7.71 (m, 1H), 7.37–7.24 (m, 7H), 7.09–7.05 (m, 1H), 7.03–6.99 (m, 1H), 6.94–6.76 (m, 3H), 6.45–6.36 (m, 1H), 5.36–5.29 (m, 1H), 5.21–5.14 (m, 1H), 5.10 (s, 2H), 4.54–4.42 (m, 1H), 4.19–4.12 (m, 1H), 2.29–2.21 (m, 3H), 2.18–2.14 (m, 3H), 1.80–1.55 (m, 6H), 1.55–1.44 (m, 3H), 1.02–0.84 (m, 18H). ^13^C‐NMR (126 MHz, CDCl_3_): *δ*=196.5, 172.3, 171.7, 171.5, 158.0, 156.4, 152.3, 136.0, 133.1, 132.8, 129.6, 128.6, 128.3, 128.1, 127.7, 124.2, 122.1, 116.6, 113.9, 67.4, 67.2, 54.1, 52.9, 51.5, 40.4, 40.2, 40.0, 39.9, 25.2, 24.7, 23.2, 22.9, 22.0, 21.9, 21.3, 16.8, 15.8. ESI‐MS: *m/z*=715.41 [M+H]^+^.

**Benzyl((*S*)‐1‐(((*S*)‐1‐(((*S*)‐1‐((2,4‐dimethyl‐3‐phenoxyphenyl)amino)‐5‐methyl‐1,2‐dioxo‐hexan‐3‐yl)amino)‐4‐methyl‐1‐oxopentan‐2‐yl)amino)‐4‐methyl‐1‐oxopentan‐2‐yl)carbama‐te (12)** Cbz‐Leu‐Leu‐Leu‐al (343 mg, 0.721 mmol, 1.0 equiv), 1‐isocyano‐2,4‐dimethyl‐3‐phenoxybenzene (242 mg, 1.08 mmol, 1.5 equiv), trifluoroacetic acid (111 μL, 164 mg, 1.44 mmol, 2.0 equiv) and IBX (404 mg, 1.44 mmol, 2.0 equiv) were reacted according to GP i to afford 12 (168 mg, 0.235 mmol, 33 %) as a pale yellow amorphous solid. R_*f*_=0.32 (cyclohexane/AcOEt=2 : 1). HPLC (254 nm, VWD): t_R_=9.09 min (96.18 %). ^1^H‐NMR (500 MHz, CDCl_3_): *δ*=8.66–8.57 (m, 1H), 7.91–7.81 (m, 1H), 7.37–7.28 (m, 5H), 7.28–7.22 (m, 2H), 7.15–7.11 (m, 1H), 6.98 (t, *J*=7.3 Hz, 1H), 6.88–6.83 (m, 1H), 6.75–6.71 (m, 2H), 6.51–6.42 (m, 1H), 5.45–5.37 (m, 1H), 5.25–5.18 (m, 1H), 5.14–5.06 (m, 2H), 4.56–4.45 (m, 1H), 4.23–4.13 (m, 1H), 2.12–2.03 (m, 6H), 1.82–1.57 (m, 6H), 1.57–1.48 (m, 3H), 1.04–0.99 (m, 3H), 0.92 (s, 15H). ^13^C‐NMR (126 MHz, CDCl_3_): *δ*=196.8, 172.5, 171.7, 157.8, 156.9, 151.2, 136.2, 133.5, 129.9, 129.1, 128.7, 128.4, 128.2, 121.7, 118.9, 114.8, 67.6, 67.4, 54.3, 53.9, 53.0, 51.7, 51.5, 41.3, 40.6, 40.3, 25.4, 25.0, 24.9, 23.4, 23.1, 23.0, 22.2, 21.6, 16.5, 10.7. ESI‐MS: *m/z*=715.41 [M+H]^+^.

**Benzyl((*S*)‐4‐methyl‐1‐(((*S*)‐4‐methyl‐1‐(((*S*)‐5‐methyl‐1‐((4‐methyl‐3‐phenoxyphenyl)‐amino)‐1,2‐dioxohexan‐3‐yl)amino)‐1‐oxopentan‐2‐yl)amino)‐1‐oxopentan‐2‐yl)carbamate (13)** Cbz‐Leu‐Leu‐Leu‐al (250 mg, 0.526 mmol, 1.0 equiv), 4‐isocyano‐1‐methyl‐2‐phenoxybenzene (165 mg, 0.789 mmol, 1.5 equiv), trifluoroacetic acid (81 μL, 120 mg, 1.05 mmol, 2.0 equiv) and IBX (295 mg, 1.05 mmol, 2.0 equiv) were reacted according to GP i to afford 13 (146 mg, 0.208 mmol, 40 %) as a pale yellow amorphous solid. R_*f*_=0.33 (cyclohexane/AcOEt=2 : 1). HPLC (254 nm, VWD): t_R_=9.09 min (98.02 %). ^1^H‐NMR (500 MHz, CDCl_3_): *δ*=8.59–8.55 (m, 1H), 7.39–7.28 (m, 8H), 7.21 (dd, *J*=8.2, 3.4 Hz, 1H), 7.18–7.16 (m, 1H), 7.08–7.03 (m, 1H), 6.92–6.88 (m, 2H), 6.78 (d, *J*=7.3 Hz, 1H), 6.48–6.41 (m, 1H), 5.36–5.30 (m, 1H), 5.24–5.16 (m, 1H), 5.09 (s, 2H), 4.53–4.42 (m, 1H), 4.21–4.14 (m, 1H), 2.23–2.19 (m, 3H), 1.78–1.55 (m, 6H), 1.54–1.44 (m, 3H), 1.01–0.84 (m, 18H). ^13^C‐NMR (126 MHz, CDCl_3_): *δ*=196.6, 172.4, 171.7, 157.7, 156.7, 155.0, 135.3, 131.9, 129.9, 128.7, 128.4, 128.2, 127.3, 122.9, 117.6, 117.5, 115.6, 111.5, 67.6, 67.4, 52.9, 51.7, 41.3, 40.6, 40.5, 40.2, 40.0, 25.5, 25.4, 25.0, 24.9, 23.3, 23.1, 22.9, 22.2, 22.0, 21.5, 16.0. ESI‐MS: *m/z*=701.39 [M+H]^+^.

**Benzyl((*S*)‐4‐methyl‐1‐(((*S*)‐4‐methyl‐1‐(((*S*)‐5‐methyl‐1‐((2‐methyl‐3‐phenoxyphenyl)‐amino)‐1,2‐dioxohexan‐3‐yl)amino)‐1‐oxopentan‐2‐yl)amino)‐1‐oxopentan‐2‐yl)carbamate (14)** Cbz‐Leu‐Leu‐Leu‐al (250 mg, 0.526 mmol, 1.0 equiv), 1‐isocyano‐2‐methyl‐3‐phenoxybenzene (165 mg, 0.789 mmol, 1.5 equiv), trifluoroacetic acid (81 μL, 120 mg, 1.05 mmol, 2.0 equiv) and IBX (295 mg, 1.05 mmol, 2.0 equiv) were reacted according to GP i to afford 14 (152 mg, 0.217 mmol, 41 %) as a pale yellow amorphous solid. R_*f*_=0.35 (cyclohexane/AcOEt=2 : 1). HPLC (254 nm, VWD): t_R_=8.98 min (97.81 %). ^1^H‐NMR (500 MHz, CDCl_3_): *δ*=8.73–8.67 (m, 1H), 7.91–7.84 (m, 1H), 7.36–7.27 (m, 7H), 7.21–7.16 (m, 1H), 7.06 (t, *J*=7.4 Hz, 1H), 7.00–6.97 (m, 1H), 6.92–6.87 (m, 2H), 6.80–6.76 (m, 1H), 6.61–6.51 (m, 1H), 5.45–5.38 (m, 1H), 5.34 (t, *J*=8.6 Hz, 1H), 5.14–5.06 (m, 2H), 4.57–4.50 (m, 1H), 4.22–4.14 (m, 1H), 2.22–2.15 (m, 3H), 1.83–1.58 (m, 6H), 1.58–1.47 (m, 3H), 1.01 (dt, *J*=11.2, 6.2 Hz, 3H), 0.97–0.86 (m, 15H). ^13^C‐NMR (126 MHz, CDCl_3_): *δ*=196.7, 172.6, 171.9, 171.8, 157.8, 156.9, 155.0, 135.8, 129.9, 128.7, 128.4, 128.2, 127.3, 122.9, 121.2, 117.7, 117.5, 117.2, 67.4, 54.2, 53.1, 53.0, 51.7, 51.5, 40.8, 40.2, 25.5, 25.4, 25.0, 24.9, 24.9, 23.3, 23.1, 22.2, 22.0, 21.6, 10.2. ESI‐MS: *m/z*=701.40 [M+H]^+^.

**Benzyl((*S*)‐4‐methyl‐1‐(((*S*)‐4‐methyl‐1‐(((*S*)‐5‐methyl‐1‐((2‐methyl‐5‐phenoxyphenyl)‐amino)‐1,2‐dioxohexan‐3‐yl)amino)‐1‐oxopentan‐2‐yl)amino)‐1‐oxopentan‐2‐yl)carbamate (15)** Cbz‐Leu‐Leu‐Leu‐al (250 mg, 0.526 mmol, 1.0 equiv), 2‐isocyano‐1‐methyl‐4‐phenoxybenzene (165 mg, 0.789 mmol, 1.5 equiv), trifluoroacetic acid (81 μL, 120 mg, 1.05 mmol, 2.0 equiv) and IBX (295 mg, 1.05 mmol, 2.0 equiv) were reacted according to GP i to afford 15 (151 mg, 0.215 mmol, 41 %) as a pale yellow amorphous solid. R_*f*_=0.35 (cyclohexane/AcOEt=2 : 1). HPLC (254 nm, VWD): t_R_=9.01 min (98.44 %). ^1^H‐NMR (500 MHz, CDCl_3_): *δ*=8.62 (d, *J*=9.2 Hz, 1H), 7.87 (dd, *J*=7.4, 2.5 Hz, 1H), 7.37–7.28 (m, 7H), 7.15–7.11 (m, 1H), 7.10–7.06 (m, 1H), 7.00–6.97 (m, 2H), 6.88–6.83 (m, 1H), 6.77–6.72 (m, 1H), 6.49–6.44 (m, 1H), 5.38–5.32 (m, 1H), 5.24 (d, *J*=7.7 Hz, 1H), 5.13–5.06 (m, 2H), 4.55–4.43 (m, 1H), 4.20–4.12 (m, 1H), 2.28 (s, 1.5H), 2.24 (s, 1.5H), 1.81–1.57 (m, 6H), 1.57–1.45 (m, 3H), 1.00–0.97 (m, 3H), 0.96–0.85 (m, 15H). ^13^C‐NMR (126 MHz, CDCl_3_): *δ*=196.6, 172.5, 171.9, 171.7, 157.5, 156.7, 155.9, 136.2, 136.1, 135.4, 131.5, 129.8, 128.7, 128.4, 128.2, 128.1, 123.3, 123.1, 118.6, 116.2, 112.7, 112.7, 67.4, 53.1, 53.0, 51.7, 51.5, 40.6, 40.2, 25.5, 25.4, 25.0, 24.9, 24.9, 23.3, 23.3, 23.1, 23.0, 23.0, 22.2, 22.1, 22.0, 21.6, 21.5, 17.0, 16.9. ESI‐MS: *m/z*=701.40 [M+H]^+^.

***tert*** **‐Butyl‐(*S*)‐3‐((*S*)‐2‐(((benzyloxy)carbonyl)amino)‐4‐methylpentanamido)‐4‐(((*S*)‐5‐methyl‐1,2‐dioxo‐1‐(phenylamino)hexan‐3‐yl)amino)‐4‐oxobutanoate (16)** Cbz‐Leu‐Asp(O*t*Bu)‐Leu‐al (265 mg, 0.497 mmol, 1.0 equiv), isocyanobenzene (77 mg, 0.746 mmol, 1.5 quiv), trifluoroacetic acid (77 μL, 113 mg, 0.994 mmol, 2.0 equiv) and IBX (278 mg, 0.994 mmol, 2.0 equiv) were reacted according to GP i to afford 16 (133 mg, 0.204 mmol, 41 %) as a pale yellow amorphous solid. R_*f*_=0.29 (cyclohexane/AcOEt=2 : 1). HPLC (254 nm, VWD): t_R_=8.27 min (98.52 %). ^1^H‐NMR (500 MHz, CDCl_3_): *δ*=8.65 (s, 1H), 7.64–7.60 (m, 2H), 7.38–7.25 (m, 9H), 7.19–7.15 (m, 1H), 5.44–5.37 (m, 1H), 5.18–5.14 (m, 1H), 5.13 (d, *J*=2.8 Hz, 2H), 4.81–4.74 (m, 1H), 4.20 (s, 1H), 2.98–2.89 (m, 1H), 2.59–2.51 (m, 1H), 1.84–1.60 (m, 4H), 1.58–1.49 (m, 2H), 1.45 (s, 9H), 1.04–0.99 (m, 3H), 0.97–0.92 (m, 9H). ^13^C‐NMR (126 MHz, CDCl_3_): *δ*=196.6, 172.2, 171.7, 170.5, 156.8, 136.4, 136.1, 129.3, 128.7, 128.5, 128.2, 125.5, 120.0, 82.2, 67.5, 54.2, 53.2, 49.3, 41.4, 40.2, 36.8, 28.2, 25.4, 25.0, 23.4, 23.1, 21.9, 21.5. ESI‐MS: *m/z*=653.35 [M+H]^+^.

***tert*** **‐Butyl‐(*S*)‐3‐((*S*)‐2‐(((benzyloxy)carbonyl)amino)‐4‐methylpentanamido)‐4‐(((*S*)‐1‐((2,4‐dime‐thylphenyl)amino)‐5‐methyl‐1,2‐dioxohexan‐3‐yl)amino)‐4‐oxobutanoate (17)** Cbz‐Leu‐Asp(O*t*Bu)‐Leu‐al (196 mg, 0.367 mmol, 1.0 equiv), 1‐isocyano‐2,4‐dimethylbenzene (72 mg, 0.549 mmol, 1.5 equiv), trifluoroacetic acid (56 μL, 83 mg, 0.734 mmol, 2.0 equiv) and IBX (206 mg, 0.734 mmol, 2.0 equiv) were reacted according to GP i to afford 17 (96 mg, 0.141 mmol, 40 %) as a pale yellow amorphous solid. R_*f*_=0.33 (CH_2_Cl_2_/MeOH=50 : 1). HPLC (254 nm, VWD): t_R_=9.47 min (97.38 %). ^1^H‐NMR (500 MHz, DMSO‐*d_6_*): *δ*=9.96 (s, 1H), 8.22 (d, *J*=8.1 Hz, 1H), 8.05 (d, *J*=7.3 Hz, 1H), 7.42 (d, *J*=8.0 Hz, 1H), 7.39–7.27 (m, 6H), 7.22 (d, *J*=8.0 Hz, 1H), 7.06 (d, *J*=1.9 Hz, 1H), 7.93–6.08 (m, 1H), 5.17–5.11 (m, 1H), 5.02 (d, *J*=4.9 Hz, 2H), 4.67–4.59 (m, 1H), 4.08–4.01 (m, 1H), 2.69 (dd, *J*=16.1, 5.3 Hz, 1H), 2.26 (s, 3H), 2.14 (s, 3H), 1.74–1.41 (m, 6H), 1.38 (s, 9H), 0.92–0.82 (m, 12H). ^13^C‐NMR (126 MHz, DMSO‐*d_6_*): *δ*=196.9, 172.1, 170.4, 169.1, 159.4, 155.9, 136.9, 135.5, 132.6, 132.0, 130.9, 127.7, 127.6, 126.5, 125.4, 80.2, 65.3, 53.1, 52.2, 49.1, 40.7, 38.4, 37.2, 27.6, 24.4, 24.1, 23.0, 22.9, 21.4, 21.1, 20.5, 17.5. ESI‐MS: *m/z*=681.37 [M+H]^+^.

***tert*** **‐Butyl‐(*S*)‐3‐((*S*)‐2‐(((benzyloxy)carbonyl)amino)‐4‐methylpentanamido)‐4‐(((*S*)‐5‐methyl‐1,2‐dioxo‐1‐((2‐phenoxyphenyl)amino)hexan‐3‐yl)amino)‐4‐oxobutanoate (18)** Cbz‐Leu‐Asp(O*t*Bu)‐Leu‐al (180 mg, 0.337 mmol, 1.0 equiv), 1‐isocyano‐2‐phenoxybenzene (209 mg, 0.506 mmol, 1.5 equiv), trifluoroacetic acid (53 μL, 80.0 mg, 0.675 mmol, 2.0 equiv) and IBX (189 mg, 0.675 mmol, 2.0 equiv) were reacted according to GP i. Column chromatography was done with a mixture of CH_2_Cl_2/_MeOH (400 : 1) to afford 18 (28.0 mg, 0.038 mmol, 11 %) as a colorless amorphous solid. R_*f*_=0.31 (CH_2_Cl_2_/MeOH=50 : 1). HPLC (254 nm, VWD): t_R_=9.27 min (99.12 %). ^1^H‐NMR (500 MHz, CDCl_3_): *δ*=9.33 (s, 1H), 8.49 (dd, *J*=8.1, 1.7 Hz, 1H), 7.38–7.30 (m, 8H), 7.23 (d, *J*=7.7 Hz, 1H), 7.18–7.14 (m, 1H), 7.12 (td, *J*=7.8, 1.5 Hz, 1H), 7.07 (dd, *J*=8.0, 1.7 Hz, 1H), 7.05–7.02 (m, 2H), 6.86 (dd, *J*=8.1, 1.5 Hz, 1H), 5.48–5.40 (m, 1H), 5.16–5.08 (m, 2H), 4.78–4.73 (m, 1H), 4.23–4.17 (m, 1H), 2.93 (d, *J*=17.2 Hz, 1H), 2.58–2.50 (m, 1H), 1.80–1.60 (m, 6H), 1.58–1.45 (m, 2H), 1.44 (s, 9H), 1.03–0.99 (m, 3H), 0.98–0.90 (m, 9H). ^13^C‐NMR (126 MHz, CDCl_3_): *δ*=196.1, 172.1, 171.7, 170.4, 156.7, 156.2, 145.0, 136.1, 130.1, 128.7, 128.4, 128.2, 128.0, 125.4, 124.4, 123.9, 120.8, 119.2, 117.6, 82.2, 67.4, 54.1, 53.1, 49.2, 41.5, 40.3, 36.8, 28.1, 25.4, 25.0, 23.4, 23.1, 21.9, 21.5. ESI‐MS: *m/z*=745.40 [M+H]^+^.

***tert*** **‐Butyl‐(*S*)‐3‐((*S*)‐2‐(((benzyloxy)carbonyl)amino)‐4‐methylpentanamido)‐4‐(((*S*)‐5‐methyl‐1,2‐dioxo‐1‐((3‐phenoxyphenyl)amino)hexan‐3‐yl)amino)‐4‐oxobutanoate (19)** Cbz‐Leu‐Asp(O*t*Bu)‐Leu‐al (280 mg, 0.525 mmol, 1.0 equiv), 1‐isocyano‐3‐phenoxybenzene (154 mg, 0.787 mmol, 1.5 equiv), trifluoroacetic acid (81 μL, 120 mg, 1.05 mmol, 2.0 equiv) and IBX (294 mg, 1.05 mmol, 2.0 equiv) were reacted according to GP i to afford 19 (153 mg, 0.205 mmol, 39 %) as a pale yellow amorphous solid. R_*f*_=0.23 (cyclohexane/AcOEt=2 : 1). HPLC (254 nm, VWD): t_R_=9.12 min (99.08 %). ^1^H‐NMR (500 MHz, CDCl_3_): *δ*=8.63 (s, 1H), 7.38–7.27 (m, 11H), 7.15–7.10 (m, 1H), 7.03–6.99 (m, 2H), 6.82–6.78 (m, 1H), 5.39–5.33 (m, 1H), 5.18–5.13 (m, 1H), 5.12 (d, *J*=2.0 Hz, 2H), 4.80–4.73 (m, 1H), 4.19 (s, 1H), 2.97–2.88 (m, 1H), 2.58–2.50 (m, 1H), 1.79–1.63 (m, 4H), 1.56–1.47 (m, 2H), 1.45 (s, 9H), 1.00–0.97 (m, 3H), 0.97–0.91 (m, 9H). ^13^C‐NMR (126 MHz, CDCl_3_): *δ*=196.3, 172.0, 171.5, 170.3, 158.0, 156.7, 156.6, 137.6, 136.0, 130.2, 129.8, 128.6, 128.3, 128.1, 123.6, 119.1, 115.5, 114.6, 110.5, 82.0, 67.3, 54.0, 52.9, 49.1, 41.3, 39.9, 36.6, 28.0, 25.2, 24.8, 23.2, 23.0, 21.7, 21.4. ESI‐MS: *m/z*=745.39 [M+H]^+^.

***tert*** **‐Butyl‐(*S*)‐3‐((*S*)‐2‐(((benzyloxy)carbonyl)amino)‐4‐methylpentanamido)‐4‐(((*S*)‐5‐methyl‐1,2‐dioxo‐1‐((4‐phenoxyphenyl)amino)hexan‐3‐yl)amino)‐4‐oxobutanoate (20)** Cbz‐Leu‐Asp(O*t*Bu)‐Leu‐al (270 mg, 0.525 mmol, 1.0 equiv), 1‐isocyano‐4‐phenoxybenzene (154 mg, 0.787 mmol, 1.5 equiv), trifluoroacetic acid (81 μL, 130 mg, 1.14 mmol, 2.0 equiv) and IBX (294 mg, 1.05 mmol, 2.0 equiv) were reacted according to GP i to afford 20 (130 mg, 0.175 mmol, 33 %) as a pale yellow amorphous solid. R_*f*_=0.29 (CH_2_Cl_2_/AcOEt=2 : 1). HPLC (254 nm, VWD): t_R_=9.05 min (97.53 %). ^1^H‐NMR (500 MHz, CDCl_3_): *δ*=8.65 (s, 1H), 7.62–7.56 (m, 2H), 7.41–7.26 (m, 9H), 7.13–7.08 (m, 1H), 7.02–6.97 (m, 4H), 5.43–5.37 (m, 1H), 5.19–5.14 (m, 1H), 5.13 (d, *J*=2.8 Hz, 2H), 4.80–4.75 (m, 1H), 4.21 (s, 1H), 2.94 (d, *J*=17.4 Hz, 1H), 2.60–2.52 (m, 1H), 1.82–1.61 (m, 4H), 1.56–1.49 (m, 2H), 1.45 (s, 9H), 1.03–0.99 (m, 3H), 0.97–0.92 (m, 9H). ^13^C‐NMR (126 MHz, CDCl_3_): *δ*=196.6, 172.2, 171.7, 170.5, 157.4, 156.7, 154.6, 136.1, 131.8, 129.9, 128.7, 128.5, 128.2, 123.5, 121.6, 119.7, 118.8, 82.2, 67.5, 54.2, 53.2, 49.3, 41.4, 40.2, 36.8, 28.2, 25.4, 25.0, 23.4, 23.1, 21.9, 21.5. ESI‐MS: *m/z*=745.39 [M+H]^+^.

***tert*** **‐Butyl(*S*)‐3‐((*S*)‐2‐(((benzyloxy)carbonyl)amino)‐4‐methylpentanamido)‐4‐(((*S*)‐1‐((2,4‐dime‐thyl‐5‐phenoxyphenyl)amino)‐5‐methyl‐1,2‐dioxohexan‐3‐yl)amino)‐4‐oxobutanoate (21)** Cbz‐Leu‐Asp(O*t*Bu)‐Leu‐al (250 mg, 0.468 mmol, 1.0 equiv), 1‐isocyano‐2,4‐dimethyl‐5‐phenoxybenzene (157 mg, 0.702 mmol, 1.5 equiv), trifluoroacetic acid (72 μL, 107 mg, 0.936 mmol, 2.0 equiv) and IBX (262 mg, 0.936 mmol, 2.0 equiv) were reacted according to GP i to afford 21 (207 mg, 0.263 mmol, 56 %) as a pale yellow amorphous solid. R_*f*_=0.27 (cyclohexane/AcOEt=2 : 1). HPLC (254 nm, VWD): t_R_=9.34 min (97.86 %). ^1^H‐NMR (500 MHz, CDCl_3_): *δ*=8.61 (s, 1H), 7.77 (s, 1H), 7.42 (d, *J*=8.2 Hz, 1H), 7.36–7.31 (m, 5H), 7.30–7.25 (m, 3H), 7.08 (s, 1H), 7.03–6.99 (m, 1H), 6.89–6.86 (m, 2H), 5.42–5.35 (m, 1H), 5.32 (d, *J*=7.2 Hz, 1H), 5.12 (s, 2H), 4.82–4.75 (m, 1H), 4.24–4.19 (m, 1H), 2.95–2.87 (m, 1H), 2.61–2.52 (m, 1H), 2.27 (s, 3H), 2.16 (s, 3H), 1.80–1.64 (m, 4H), 1.58–1.49 (m, 2H), 1.45 (s, 9H), 1.00–0.97 (m, 3H), 0.96–0.92 (m, 9H). ^13^C‐NMR (126 MHz, CDCl_3_): *δ*=196.6, 172.2, 171.5, 170.3, 158.1, 156.4, 152.3, 136.1, 133.2, 133.0, 129.7, 128.6, 128.3, 128.1, 127.7, 124.2, 122.2, 116.6, 114.0, 82.0, 67.3, 54.1, 53.0, 49.2, 41.3, 40.0, 36.7, 28.1, 25.3, 24.9, 23.3, 23.0, 21.8, 21.4, 16.9, 15.8. ESI‐MS: *m/z*=773.41 [M+H]^+^.

**(*S*)‐4‐Methyl‐*N*‐((*S*)‐4‐methyl‐1‐(((*S*)‐4‐methyl‐1‐oxopentan‐2‐yl)amino)‐1‐oxopentan‐2‐yl)‐2‐(2‐(4‐oxoquinazolin‐3(4*H*)‐yl)acetamido)pentanamide (22)** (*S*)‐*N*‐((*S*)‐1‐Hydroxy‐4‐methylpentan‐2‐yl)‐4‐methyl‐2‐((*S*)‐4‐methyl‐2‐(2‐(4‐oxoquinazolin‐3(4*H*)yl)acet‐amido)pen‐tanamido)pentanamide (130 mg, 0.245 mmol, 1.0 equiv) and IBX (103 mg, 0.368 mmol, 1.5 equiv) in 6 mL of DMSO were reacted according to GP ii to afford 22 (109 mg, 0.207 mmol, 84 %) as a colorless amorphous solid. R_*f*_=0.13 (cyclohexane/AcOEt=1 : 5). HPLC (254 nm, VWD): t_R_=4.97 min (97.80 %). ^1^H‐NMR (500 MHz, DMSO‐*d_6_*): *δ*=9.32 (s, 1H), 8.61 (d, *J*=7.8 Hz, 1H), 8.29 (s, 1H), 8.11 (dd, *J*=8.0, 1.5 Hz, 1H), 8.03 (d, *J*=7.3 Hz, 1H), 7.98 (d, *J*=8.1 Hz, 1H), 7.84 (ddd, *J*=8.5, 7.1, 1.6 Hz, 1H), 7.70 (dd, *J*=8.2, 1.0 Hz, 1H), 7.56 (ddd, *J*=8.1, 7.2, 1.2 Hz, 1H), 4.71 (s, 2H), 4.36–4.26 (m, 2H), 4.04–3.98 (m, 1H), 1.69–1.58 (m, 2H), 1.57–1.41 (m, 6H), 1.36–1.29 (m, 1H), 0.91–0.89 (m, 6H), 0.87–0.84 (m, 6H), 0.80–0.78 (m, 3H), 0.75–0.73 (m, 3H). ^13^C‐NMR (126 MHz, DMSO‐*d_6_*): *δ*=201.2, 172.3, 171.4, 166.9, 160.3, 148.5, 148.1, 134.4, 127.2, 127.0, 125.9, 121.4, 56.4, 51.4, 51.1, 48.2, 40.8, 40.4, 36.2, 24.2, 24.1, 23.8, 23.0, 22.9, 22.8, 21.7, 21.1. ESI‐MS: *m/z*=528.33 [M+H]^+^.

***N*** **‐((*S*)‐4‐Methyl‐1‐(((*S*)‐4‐methyl‐1‐(((*S*)‐4‐methyl‐1‐oxopentan‐2‐yl)amino)‐1‐oxopentan‐2‐yl)amino)‐1‐oxopentan‐2‐yl)pyrazine‐2‐carboxamide (23)** *N*‐((*S*)‐1‐(((*S*)‐1‐(((*S*)‐1‐Hydroxy‐4‐methylpentan‐2‐yl)amino)‐4‐methyl‐1‐oxopentan‐2‐yl)amino)‐4‐methyl‐1‐oxopentan‐2‐yl)pyrazine‐2‐carboxamide (120 mg, 0.267 mmol, 1.0 equiv) and IBX (112 mg, 0.400 mmol, 1.5 equiv) in 6 mL of DMSO were reacted according to GP ii to afford 23 (107 mg, 0.239 mmol, 90 %) as a colorless amorphous solid. R_*f*_=0.34 (cyclohexane/AcOEt=1 : 5). HPLC (254 nm, VWD): t_R_=4.62 min (98.99 %). ^1^H‐NMR (500 MHz, CDCl_3_): *δ*=9.54 (s, 1H), 9.37 (d, *J*=1.5 Hz, 1H), 8.77 (d, *J*=2.5 Hz, 1H), 8.55–8.53 (m, 1H), 8.15 (d, *J*=8.2 Hz, 1H), 6.77–6.69 (m, 2H), 4.69–4.61 (m, 1H), 4.50–4.45 (m, 2H), 1.80–1.65 (m, 6H), 1.63–1.43 (m, 3H), 0.98–0.92 (m, 12H), 0.90–0.86 (m, 3H), 0.86–0.83 (m, 3H). ^13^C‐NMR (126 MHz, CDCl_3_): *δ*=199.5, 172.0, 171.9, 163.5, 147.8, 144.6, 144.0, 142.9, 57.4, 52.2, 52.1, 41.1, 40.6, 37.9, 25.0, 24.9, 24.9, 23.2, 23.0, 22.9, 22.2, 22.0. ESI‐MS: *m/z*=482.24 [M+H]^+^.

**2,5‐Dichloro‐*N*‐((*S*)‐4‐methyl‐1‐(((*S*)‐4‐methyl‐1‐(((*S*)‐4‐methyl‐1‐oxopentan‐2‐yl)‐amino)‐1‐oxo‐pentan‐2‐yl)amino)‐1‐oxopentan‐2‐yl)benzamide (24)** 2,5‐Dichloro‐*N*‐((*S*)‐1‐(((*S*)‐1‐(((*S*)‐1‐hydroxy‐4‐methylpentan‐2‐yl)amino)‐4‐methyl‐1‐oxopentan‐2‐yl)amino)‐4‐methyl‐1‐oxopentan‐2‐yl)benzamide (820 mg, 1.59 mmol, 1.0 equiv) and IBX (667 mg, 2.38 mmol, 1.5 equiv) in 20 mL of DMSO were reacted according to GP ii to afford 24 (722 mg, 1.40 mmol, 88 %) as a colorless amorphous solid. R_*f*_=0.60 (cyclohexane/AcOEt=1 : 2). HPLC (205 nm, VWD): t_R_=7.20 min (97.85 %). ^1^H‐NMR (500 MHz, CDCl_3_): *δ*=9.50 (s, 1H), 7.47 (t, *J*=1.4 Hz, 1H), 7.30 (d, *J*=1.5 Hz, 2H), 7.15–7.10 (m, 2H), 7.01 (d, *J*=7.5 Hz, 1H), 4.82–4.73 (m, 1H), 4.67–4.59 (m, 1H), 4.39–4.32 (m, 1H), 1.78–1.54 (m, 8H), 1.44–1.37 (m, 1H), 0.98–0.85 (m, 18H). ^13^C‐NMR (126 MHz, CDCl_3_): *δ*=199.6, 172.2, 171.7, 165.4, 136.1, 133.3, 131.5, 129.9, 129.3, 57.4, 52.6, 51.9, 41.4, 41.2, 37.6, 25.1, 25.0, 24.8, 23.2, 23.0, 22.9, 22.5, 21.9. ESI‐MS: *m/z*=514.23 [M+H]^+^.

***tert*** **‐Butyl (*S*)‐3‐((*S*)‐2‐(2,5‐dichlorobenzamido)‐4‐methylpentanamido)‐4‐(((*S*)‐4‐methyl‐1‐oxopen‐tan‐2‐yl)amino)‐4‐oxobutanoate (25)** *tert*‐Butyl (*S*)‐3‐((*S*)‐2‐(2,5‐dichlorobenzamido)‐4‐methylpentanamido)‐4‐(((*S*)‐1‐hydroxy‐4‐methylpentan‐2‐yl)amino)‐4‐oxobutanoate (700 mg, 1.22 mmol, 1.0 equiv) and IBX (512 mg, 1.83 mmol, 1.5 equiv) in 8 mL of DMSO were reacted according to GP ii to afford 25 (537 mg, 0.938 mmol, 77 %) as a colorless amorphous solid. R_*f*_=0.15 (cyclohexane/AcOEt=2 : 1). HPLC (205 nm, VWD): t_R_=7.53 min (96.63 %). ^1^H‐NMR (500 MHz, CDCl_3_): *δ*=9.53 (s, 1H), 7.75–7.74 (m, 1H), 7.58 (d, *J*=8.3 Hz, 1H), 7.37–7.36 (m, 2H), 7.18 (d, *J*=7.7 Hz, 1H), 6.58 (d, *J*=6.4 Hz, 1H), 4.84–4.78 (m, 1H), 4.59–4.54 (m, 1H), 4.44–4.39 (m, 1H), 3.05 (dd, *J*=17.3, 3.9 Hz, 1H), 2.58 (dd, *J*=17.2, 6.2 Hz, 1H), 1.87–1.75 (m, 2H), 1.72–1.59 (m, 4H), 1.44 (s, 9H), 1.03–0.99 (m, 6H), 0.91–0.89 (m, 3H), 0.86 (d, *J*=6.4 Hz, 3H). ^13^C‐NMR (126 MHz, CDCl_3_): *δ*=199.9, 172.0, 171.2, 170.7, 166.2, 135.7, 133.6, 131.9, 131.6, 130.4, 129.0, 82.6, 57.6, 53.7, 49.6, 40.9, 37.5, 36.5, 28.2, 25.2, 24.7, 23.2, 21.7. ESI‐MS: *m/z*=572.23 [M+H]^+^.

**2,5‐Dichloro‐*N*‐((*S*)‐1‐(((*S*)‐1‐(((*S*)‐1‐((2,4‐dimethylphenyl)amino)‐5‐methyl‐1,2‐dioxohexan‐3‐yl)amino)‐4‐methyl‐1‐oxopentan‐2‐yl)amino)‐4‐methyl‐1‐oxopentan‐2‐yl)‐benzamide (26)** Compound 24 (425 mg, 0.826 mmol, 1.0 equiv), 1‐isocyano‐2,4‐dimethylbenzene (163 mg, 1.24 mmol, 1.5 equiv), trifluoroacetic acid (127 μL, 188 mg, 1.65 mmol, 2.0 equiv) in 10 mL of CH_2_Cl_2_ and IBX (462 mg, 1.65 mmol, 2.0 equiv) in 5 mL of DMSO were reacted according to GP 4 to afford 26 (184 mg, 0.268 mmol, 17 %) as a colorless amorphous solid. R_*f*_=0.32 (cyclohexane/AcOEt=2 : 1). HPLC (254 nm, VWD): t_R_=8.43 min (97.54 %). ^1^H‐NMR (500 MHz, CDCl_3_): *δ*=9.95 (s, 1H), 8.69 (d, *J*=8.0 Hz, 1H), 8.25 (d, *J*=7.0 Hz, 1H), 7.94 (d, *J*=8.3 Hz, 1H), 7.53 (s, 2H), 7.43 (s, 1H), 7.22 (d, *J*=8.0 Hz, 1H), 7.06 (s, 1H), 7.00 (d, *J*=8.1 Hz, 1H), 5.16–5.07 (m, 1H), 4.51–4.40 (m, 2H), 2.26 (s, 3H), 2.15 (s, 3H), 1.75–1.62 (m, 3H), 1.61–1.43 (m, 6H), 0.96–0.83 (m, 18H). ^13^C‐NMR (126 MHz, CDCl_3_): *δ*=197.1, 172.0, 171.1, 164.8, 159.6, 138.0, 135.4, 132.5, 132.0, 131.4, 131.3, 130.8, 130.5, 128.7, 128.5, 126.5, 125.3, 52.1, 51.7, 50.5, 41.0, 39.9, 38.2, 24.5, 24.2, 24.0, 23.0, 23.0, 21.7, 21.5, 21.0, 20.5, 17.5. ESI‐MS: *m/z*=661.30[M+H]^+^.

**2,5‐Dichloro‐*N*‐((*S*)‐1‐(((*S*)‐1‐(((*S*)‐1‐((2,4‐dimethyl‐5‐phenoxyphenyl)amino)‐5‐methyl‐1,2‐dioxo‐hexan‐3‐yl)amino)‐4‐methyl‐1‐oxopentan‐2‐yl)amino)‐4‐methyl‐1‐oxopentan‐2‐yl)benzamide (27)** Compound 24 (294 mg, 0.572 mmol, 1.0 equiv), 1‐isocyano‐2,4‐dimethyl‐5‐phenoxybenzene (200 mg, 0.857 mmol, 1.5 equiv), trifluoroacetic acid (127 μL, 188 mg, 1.65 mmol, 2.0 equiv) in 8 mL of CH_2_Cl_2_ and IBX (462 mg, 1.65 mmol, 2.0 equiv) in 5 mL of DMSO were reacted according to GP i to afford 27 (136 mg, 0.180 mmol, 31 %) as a pale yellow amorphous solid. R_*f*_=0.33 (cyclohexane/AcOEt=2 : 1). HPLC (254 nm, VWD): t_R_=9.27 min (99.05 %). ^1^H‐NMR (500 MHz, CDCl_3_): *δ*=8.56 (s, 1H), 7.69 (s, 1H), 7.50 (t, *J*=1.5 Hz, 1H), 7.30–7.26 (m, 5H), 7.08 (s, 1H), 7.03–6.99 (m, 2H), 6.96 (d, *J*=7.5 Hz, 1H), 6.89–6.86 (m, 2H), 5.32–5.26 (m, 1H), 4.79–4.72 (m, 1H), 4.63–4.56 (m, 1H), 2.27 (s, 3H), 2.16 (s, 3H), 1.77–1.60 (m, 7H), 1.58–1.50 (m, 1H), 1.49–1.41 (m, 1H), 0.98–0.87 (m, 18H). ^13^C‐NMR (126 MHz, CDCl_3_): *δ*=196.7, 165.4, 158.1, 156.6, 152.5, 136.0, 133.3, 133.2, 133.0, 131.5, 130.1, 129.8, 129.2, 127.9, 124.4, 122.3, 116.8, 114.1, 53.1, 52.6, 51.7, 41.3, 41.1, 40.1, 25.4, 25.0, 24.9, 23.3, 23.0, 22.9, 22.4, 22.4, 21.5, 17.0, 15.9. ESI‐MS: *m/z*=753.33 [M+H]^+^.

***tert*** **‐Butyl (*S*)‐3‐((*S*)‐2‐(2,5‐dichlorobenzamido)‐4‐methylpentanamido)‐4‐(((*S*)‐1‐((2,4‐dimethyl‐phenyl)amino)‐5‐methyl‐1,2‐dioxohexan‐3‐yl)amino)‐4‐oxobutanoate (28)** Compound 25 (250 mg, 0.437 mmol, 1.0 equiv), 1‐isocyano‐2,4‐dimethylbenzene (86 mg, 0.655 mmol, 1.5 equiv), trifluoroacetic acid (67 μL, 100 mg, 0.874 mmol, 2.0 equiv) in 8 mL of CH_2_Cl_2_ and IBX in 5 mL of DMSO (245 mg, 0.874 mmol, 2.0 equiv) were reacted according to GP i to afford 28 (138 mg, 0.192 mmol, 44 %) as a pale yellow amorphous solid. R_*f*_=0.19 (cyclohexane/AcOEt=2 : 1). HPLC (254 nm, VWD): t_R_=8.64 min (97.46 %). ^1^H‐NMR (500 MHz, CDCl_3_): *δ*=8.58 (s, 1H), 7.87 (d, *J*=8.0 Hz, 1H), 7.68 (t, *J*=1.5 Hz, 1H), 7.58 (d, *J*=8.3 Hz, 1H), 7.32 (d, *J*=1.4 Hz, 2H), 7.30 (d, *J*=7.9 Hz, 1H), 7.01–6.97 (m, 2H), 6.79 (d, *J*=6.9 Hz, 1H), 5.51–5.41 (m, 1H), 4.82–4.77 (m, 1H), 4.69–4.60 (m, 1H), 2.97 (dd, *J*=17.2, 4.1 Hz, 1H), 2.58 (dd, *J*=17.2, 6.5 Hz, 1H), 2.27 (s, 3H), 2.23 (s, 3H), 1.82–1.72 (m, 4H), 1.72–1.64 (m, 1H), 1.56–1.48 (m, 1H), 1.43 (s, 9H), 1.00–0.97 (m, 9H), 0.88–0.86 (m, 3H). ^13^C‐NMR (126 MHz, CDCl_3_): *δ*=196.7, 171.7, 171.3, 170.2, 165.9, 156.6, 135.9, 135.5, 133.4, 131.8, 131.6, 131.4, 131.4, 130.2, 129.0, 128.6, 127.5, 121.6, 82.2, 53.3, 53.2, 49.4, 41.0, 40.2, 36.7, 28.1, 25.3, 25.1, 23.3, 23.1, 21.8, 21.3, 21.0, 17.5. ESI‐MS: *m/z*=719.30 [M+H]^+^.

***tert*** **‐Butyl (*S*)‐3‐((*S*)‐2‐(2,5‐dichlorobenzamido)‐4‐methylpentanamido)‐4‐(((*S*)‐1‐((2,4‐dimethyl‐5‐phenoxyphenyl)amino)‐5‐methyl‐1,2‐dioxohexan‐3‐yl)amino)‐4‐oxobuta‐noate (29)** Compound 25 (260 mg, 0.454 mmol, 1.0 equiv), 1‐isocyano‐2,4‐dimethyl‐5‐phenoxybenzene (152 mg, 0.681 mmol, 1.5 equiv), trifluoroacetic acid (70 μL, 104 mg, 0.908 mmol, 2.0 equiv) in 8 mL of CH_2_Cl_2_ and IBX (254 mg, 0.908 mmol, 2.0 equiv) in 5 mL of DMSO were reacted according to GP i to afford 29 (184 mg, 0.227 mmol, 50 %) as a pale yellow amorphous solid. R_*f*_=0.21 (cyclohexane/AcOEt=2 : 1). HPLC (254 nm, VWD): t_R_=9.49 min (99.28 %).

^1^H‐NMR (500 MHz, CDCl_3_): *δ*=8.64 (s, 1H), 7.76 (s, 1H), 7.71–7.69 (m, 1H), 7.61 (d, *J*=8.3 Hz, 1H), 7.34 (d, *J*=1.4 Hz, 2H), 7.32–7.26 (m, 3H), 7.08 (s, 1H), 7.03–6.98 (m, 1H), 6.90–6.86 (m, 2H), 6.84 (d, *J*=6.9 Hz, 1H), 5.46–5.39 (m, 1H), 4.84–4.78 (m, 1H), 4.70–4.63 (m, 1H), 2.97 (dd, *J*=17.2, 4.2 Hz, 1H), 2.59 (dd, *J*=17.2, 6.5 Hz, 1H), 2.27 (s, 3H), 2.16 (s, 3H), 1.84–1.77 (m, 2H), 1.77–1.67 (m, 3H), 1.55–1.49 (m, 1H), 1.45 (s, 9H), 1.02–0.96 (m, 9H), 0.88–0.85 (m, 3H). ^13^C‐NMR (126 MHz, CDCl_3_): *δ*=196.6, 171.6, 171.3, 170.1, 165.9, 158.1, 156.4, 152.3, 135.9, 133.3, 133.1, 133.0, 131.6, 131.4, 130.1, 129.7, 129.0, 127.7, 124.3, 122.1, 116.6, 114.0, 82.2, 53.3, 53.0, 49.4, 41.0, 40.0, 36.6, 28.0, 25.2, 25.0, 23.2, 23.1, 21.7, 21.3, 16.9, 15.8. ESI‐MS: *m/z*=811.32 [M+H]^+^.

**(*S*)‐3‐((*S*)‐2‐((*S*)‐2‐(2‐(4‐(2,8‐Diethyl‐5,5‐difluoro‐1,3,7,9‐tetramethyl‐5*H*‐4λ^4^,5λ^4^‐dipyrrolo[1,2‐*c*:2′,1′‐*f*][1,3,2]diazaborinin‐10‐yl)phenoxy)acetamido)‐4‐methylpentanami‐do)‐4‐methylpentanamido)‐5‐methyl‐2‐oxo‐*N*‐(3‐phenoxyphenyl)hexanamide (32)** (3 *S*)‐3‐((*S*)‐2‐((*S*)‐2‐Amino‐4‐methylpentanamido)‐4‐methylpentanamido)‐2‐hydroxy‐5‐methyl‐*N*‐(3‐phenoxyphenyl)hexanamide (87 mg, 0.159 mmol, 1.1 equiv), 2‐(4‐(2,8‐diethyl‐5,5‐difluoro‐1,3,7,9‐tetramethyl‐5*H*‐4λ^4^,5λ^4^‐dipyrrolo[1,2‐*c*:2′,1′‐*f*][1,3,2]diazaborinin‐10‐yl)phe‐noxy)acetic acid (66 mg, 0.145 mmol, 1.0 equiv), HATU (60 mg, 0.159 mmol, 1.1 equiv), and DIPEA (72 μL, 54 mg, 0.421 mmol, 2.9 equiv) in 10 mL of DMF were reacted according to GP iii. The crude coupling product was directly taken up in 4 mL of DMSO und oxidized with IBX (81 mg, 0.290 mmol, 2.0 equiv). Column chromatography of the residue on silica (cyclohexane/AcOEt 2 : 1) afforded 32 (48 mg, 0.0485 mmol, 33 %) as a purple amorphous solid. R_*f*_=0.47 (cyclohexane/AcOEt=1 : 1). HPLC (360 nm, VWD): t_R_=10.59 min (99.29 %). ^1^H‐NMR (500 MHz, CDCl_3_): *δ*=8.65 (s, 1H), 7.39–7.34 (m, 2H), 7.34–7.31 (m, 2H), 7.30–7.27 (m, 1H), 7.25–7.22 (m, 2H), 7.15–7.10 (m, 1H), 7.05 (d, *J*=8.6 Hz, 2H), 7.03–6.98 (m, 3H), 6.83–6.78 (m, 1H), 6.64 (d, *J*=7.4 Hz, 1H), 6.53 (d, *J*=8.0 Hz, 1H), 5.39–5.32 (m, 1H), 4.60–4.53 (m, 3H), 4.51–4.43 (m, 1H), 2.53 (s, 6H), 2.30 (q, *J*=7.6 Hz, 4H), 1.82–1.67 (m, 5H), 1.67–1.58 (m, 2H), 1.57–1.49 (m, 2H), 1.31 (s, 6H), 1.03–0.88 (m, 24H). ^13^C‐NMR (126 MHz, CDCl_3_): *δ*=196.5, 171.7, 171.6, 168.4, 168.2, 158.3, 157.5, 156.8, 154.0, 139.5, 138.3, 137.6, 133.0, 131.2, 130.4, 130.1, 130.0, 123.9, 119.3, 119.3, 115.7, 115.4, 114.7, 110.6, 67.3, 53.1, 51.8, 51.6, 41.1, 40.7, 40.4, 25.6, 25.5, 25.1, 25.0, 24.9, 23.3, 23.1, 23.0, 22.9, 22.3, 22.3, 22.2, 22.1, 21.6, 17.2, 14.8, 12.7, 12.0. ^19^F NMR (471 MHz, CDCl_3_): *δ*=‐145.81 (dd, *J*=66.5, 32.1 Hz). ESI‐MS: *m/z*=969.53 [M−F]^+^.

**(*S*)‐3‐((*S*)‐2‐((*S*)‐2‐(2‐(2‐(4‐(2,8‐diethyl‐5,5‐difluoro‐1,3,7,9‐tetramethyl‐5*H*‐4λ^4^,5λ^4^‐dipyrrolo[1,2‐*c*:2′,1′‐*f*][1,3,2]diazaborinin‐10‐yl)phenoxy)acetamido)acetamido)‐4‐methyl‐pentanamido)‐4‐methylpentan‐amido)‐5‐methyl‐2‐oxo‐*N*‐(3‐phenoxyphenyl)hexanamide (33)** (3 *S*)‐3‐((*S*)‐2‐((*S*)‐2‐Amino‐4‐methylpentan‐amido)‐4‐methylpentanamido)‐2‐hydroxy‐5‐methyl‐*N*‐(3‐phenoxyphenyl)hexanamide (207 mg, 0.373 mmol, 1.5 equiv), (2‐(4‐(2,8‐Diethyl‐5,5‐difluoro‐1,3,7,9‐tetramethyl‐5*H*‐4λ^4^,5λ^4^‐dipyrrolo[1,2‐*c*:2′,1′‐*f*][1,3,2]diazabo‐rinin‐10‐yl)phenoxy)acetyl)glycine (127 mg, 0.249 mmol, 1.0 equiv), HATU (104 mg, 0.274 mmol, 1.1 equiv), and DIPEA (123 μL, 93 mg, 0.721 mmol, 2.9 equiv) in 10 mL of DMF were reacted according to GP iii. The crude coupling product was directly taken up in 10 mL of DMSO und oxidized with IBX (139 mg, 0.498 mmol, 2.0 equiv). Column chromatography of the residue on silica (cyclohexane/AcOEt 1 : 1) afforded 33 (122 mg, 0.117 mmol, 47 %) as a purple amorphous solid. R_*f*_=0.31 (cyclohexane/AcOEt=1 : 2). HPLC (360 nm, VWD): t_R_=10.20 min (99.48 %). ^1^H‐NMR (500 MHz, DMSO‐*d_6_*): *δ*=10.68–10.55 (m, 1H), 8.37–8.29 (m, 1H), 8.28–8.20 (m, 1H), 8.09–8.02 (m, 1H), 7.94 (d, *J*=8.2 Hz, 1H), 7.60–7.55 (m, 1H), 7.53 (t, *J*=2.3 Hz, 1H), 7.44–7.35 (m, 2H), 7.35–7.30 (m, 1H), 7.28–7.20 (m, 2H), 7.18–7.12 (m, 3H), 7.03–6.98 (m, 2H), 6.79–6.74 (m, 1H), 5.09–4.91 (m, 1H), 4.63–4.57 (m, 2H), 4.36–4.30 (m, 2H), 3.87–3.78 (m, 2H), 2.43 (s, 6H), 2.35–2.23 (m, 4H), 1.74–1.64 (m, 1H), 1.62–1.49 (m, 4H), 1.49–1.37 (m, 4H), 1.32–1.28 (m, 6H), 0.97–0.91 (m, 6H), 0.91–0.81 (m, 14H), 0.81–0.76 (m, 4H). ^13^C‐NMR (126 MHz, DMSO‐*d_6_*): *δ*=196.8, 172.1, 171.6, 168.3, 167.7, 159.7, 158.1, 156.9, 156.3, 152.9, 140.4, 139.0, 138.1, 132.4, 130.3, 130.0, 129.3, 127.4, 123.6, 118.7, 115.4, 115.2, 114.4, 110.4, 66.9, 52.1, 51.0, 50.5, 41.7, 40.9, 40.7, 38.1, 24.4, 24.1, 24.0, 23.0, 22.7, 21.8, 21.6, 21.0, 16.4, 14.5, 12.2, 11.5. ^19^F NMR (471 MHz, DMSO‐*d_6_*): *δ*=‐143.01 (dd, *J*=66.5, 28.6 Hz). ^11^B NMR (160 MHz, DMSO‐*d_6_*): *δ=*3.69 (t, *J*=33.4 Hz). ESI‐MS: *m/z*=1026.56 [M−F]^+^.

**(*S*)‐3‐((*S*)‐2‐((*S*)‐2‐(3‐(2‐(4‐(2,8‐diethyl‐5,5‐difluoro‐1,3,7,9‐tetramethyl‐5*H*‐4λ^4^,5λ^4^‐dipyrrolo[1,2‐*c*:2′,1′‐*f*][1,3,2]diazaborinin‐10‐yl)phenoxy)acetamido)propanamido)‐4‐methylpentanamido)‐4‐methylpen‐tan‐amido)‐5‐methyl‐2‐oxo‐*N*‐(3‐phenoxyphenyl)hexan‐amide (34)** (3 *S*)‐3‐((*S*)‐2‐((*S*)‐2‐Amino‐4‐methylpen‐tanamido)‐4‐methylpentanamido)‐2‐hydroxy‐5‐methyl‐*N*‐(3‐phenoxyphenyl)hexanamide (207 mg, 0.373 mmol, 1.5 equiv), 3‐(2‐(4‐(2,8‐Diethyl‐5,5‐difluoro‐1,3,7,9‐tetramethyl‐5*H*‐4λ^4^,5λ^4^‐dipyrrolo[1,2‐*c*:2′,1′‐*f*][1,3,2]diaza‐borinin10‐yl)phenoxy)acetamido)propanoic acid (130 mg, 0.249 mmol, 1.0, equiv), HATU (104 mg, 0.274 mmol, 1.1 equiv), and DIPEA (123 μL, 93 mg, 0.721 mmol, 2.9 equiv) in 10 mL of DMF were reacted according to GP iii. The crude coupling product was directly taken up in 10 mL of DMSO und oxidized with IBX (139 mg, 0.498 mmol, 2.0 equiv). Column chromatography of the residue on silica (cyclohexane/AcOEt 1 : 2) afforded 34 (75 mg, 0.0707 mmol, 28 %) as a purple amorphous solid. R_*f*_=0.29 (cyclohexane/AcOEt=1 : 5). HPLC (360 nm, VWD): t_R_=10.16 min (98.54 %). ^1^H‐NMR (500 MHz, DMSO‐*d_6_*): *δ*=10.63 (s, 1H), 8.31–8.26 (m, 1H), 8.14–8.08 (m, 1H), 8.03 (d, *J*=8.0 Hz, 1H), 7.90–7.81 (m, 1H), 7.59–7.56 (m, 1H), 7.55–7.51 (m, 1H), 7.39 (t, *J*=8.0 Hz, 2H), 7.35–7.30 (m, 1H), 7.27–7.23 (m, 2H), 7.17–7.11 (m, 3H), 7.01 (d, *J*=8.0 Hz, 2H), 6.79–6.74 (m, 1H), 5.03–4.92 (m, 1H), 4.52 (s, 2H), 4.40–4.26 (m, 2H), 3.39–3.33 (m, 2H), 2.43 (s, 6H), 2.38–2.32 (m, 2H), 2.28 (q, *J*=7.5 Hz, 4H), 1.73–1.65 (m, 1H), 1.61–1.49 (m, 4H), 1.47–1.37 (m, 4H), 1.30 (s, 6H), 0.93 (t, *J*=7.5 Hz, 6H), 0.91–0.85 (m, 5H), 0.85–0.77 (m, 13H). ^13^C‐NMR (126 MHz, DMSO‐*d_6_*): *δ*=196.8, 172.3, 172.2, 171.8, 171.7, 170.5, 170.4, 167.1, 159.7, 159.5, 158.0, 156.9, 156.3, 152.9, 140.4, 139.0, 138.1, 132.4, 130.3, 130.0, 129.3, 127.4, 123.5, 118.7, 115.4, 115.2, 114.4, 110.4, 67.0, 52.1, 51.0, 50.5, 41.1, 40.8, 40.7, 40.5, 38.1, 35.2, 35.0, 24.4, 24.2, 24.0, 23.0, 23.0, 22.8, 21.7, 21.6, 21.0, 20.8, 16.4, 14.5, 12.2, 11.5. ^19^F NMR (471 MHz, DMSO‐*d_6_*): *δ*=‐143.02 (dd, *J*=66.5, 28.6 Hz). ^11^B NMR (160 MHz, DMSO‐*d_6_*): *δ=*3.68 (t, *J*=33.8 Hz). ESI‐MS: *m/z*=1040.57 [M−F]^+^.

### Biology

***Proteasome inhibition assay***. Bortezomib (Selleckchem) and carfilzomib (MedChemExpress) were purchased and used without further purification. The 20 S Proteasome Assay Kit for Drug Discovery was used according to the supplier's protocol using Suc‐LLVY‐AMC (β5), Bz‐VGR‐AMC (β2) or Z‐LLE‐AMC (β1) as substrates (Enzo Life Sciences). Single point measurements of residual activity of proteasome activity were performed at a final concentration of 100 nM (n=3, β5c, β5i) or 1 μM (n=1, β1c, β2c, β5c, β5i, SI chapter 2a, Table S1). For key compounds **7**, **9**, **27** and carfilzomib **3** in this study half‐maximal inhibitory values were determined using inhibitor concentrations spanning four orders of magnitude (β5c, β5i; 5 nM to 10 μM Figure S1). Inhibitors were prepared in 2‐fold concentration (c_final_=100 nM) and added to proteasomes (m_final_=200 ng/well). After 30 min incubation, substrate was added (c_final_=100 μM), incubated for 60 minutes and fluorescence was read using a Tecan M1000 microplate reader. Dose‐response curves were fitted to the equation Y=100/(1+10^((L^°^gIC50−X)*HillSl^°^pe)^) using GraphPad Prism 7.02. IC_50_ values were determined from technical duplicates as mean value of three independent experiments.

***Cell lines***. MV4‐11 (ACC 102), Jurkat (ACC 282) and THP‐1 (ACC 16) cells were obtained from DSMZ and maintained at 37 °C and 5 % CO_2_ at densities between 0.2–1×10^6^ cells per mL in RMPI‐1640 medium supplemented with 10 % FBS, L‐glutamine (2 mM) and penicillin/streptomycin.

***Cell viability endpoint assay***. For the determination of cytotoxicity of proteasome inhibitors 50.000 cells per well were plated in 90 μl RPMI‐1640 medium supplemented with 0.1 % FBS and incubated overnight at 37 °C and 5 % CO_2_. Inhibitors were serially diluted in RPMI‐1640 medium as 10‐fold stocks and added to cells giving the final concentration (1 nM to 10 μM, Figure S2–S4) and incubated for 72 h at 37 °C and 5 % CO_2_. Cell viability was determined using the Celltiter Blue assay (Promega) by adding 20 μl of reagent, incubation for 120 minutes at 37 °C and 5 % CO_2_ and fluorescent read‐out using a Tecan M1000 microplate reader. Dose‐response curves were fitted to the equation “Y=Bottom+(Top‐Bottom)/(1+10^(X−L^°^gIC50)^)” using GraphPad Prism 7.02. IC_50_ values are determined from technical triplicates as mean value of two independent experiments. Standard deviations (Table S1) as well as dose response curves (SI chapter 2b) are given in the supplementary information.

***In‐cell determination of proteasome IC***_***50***._ Cellular inhibition of conversion of proteasome substrate **30** was determined by plating 25.000 cells per well in RPMI‐1640 medium supplemented with 0.1 % FBS. Inhibitors were serially diluted in RPMI‐1640 medium as 10‐fold stocks, added to cells giving final concentrations and incubated for 0 h–13 h at 37 °C and 5 % CO_2_ (Figure S5–S8). Substrate was added to give a final concentration of 30 μM and substrate conversion was detected by fluorescence measurement over 100 min (assay validation) or 60 min (inhibition assays) using a Tecan M1000 microplate reader. Rate of fluorescence change over time was calculated, rates for negative (no‐cell) control was subtracted and normalized to positive control (no inhibitor). Dose‐response curves were fitted to the equation “Y=100/(1+10^((L^°^gIC50−X)*HillSl^°^pe)^)” using GraphPad Prism 7.02 (SI chapter 2b, Figure S2–S4).

***Time‐dependent determination of cell viability***. For the determination of time‐dependent cytotoxic activity 5.000 cells per well were plated in a white 384 well‐plate in RPMI‐1640 medium supplemented with 0.1 % FBS. Real‐Time Glo MT cell viability assay (Promega) and inhibitor were added to the final concentration and luminescence was determined using a Tecan M1000 microplate reader at the time points indicated (SI chapter 2b, Figure S9–S17, Table S2).

***Danio rerio embryo toxicity assay***. Fish keeping protocols are approved by the Darmstadt administrative authority and documented. All fish were treated humanely. Inhibitors **7**, **9**, **27** as well as bortezomib and carfilzomib were prepared at 25 μM, 10 μM, 5 μM and 1 μM inhibitor in E3 medium. For better solubility of the compounds in the media, the wells contain 1 % v/v DMSO. Embryos were decollated into separate wells containing a total volume of 200 μL and the inhibitor at the given concentration.

***Danio rerio escape response assay***. 24 hours past fertilization embryos were dechorionated and transferred into 96‐well plates containing 198 μL of E3 media at 26 °C. Embryos were treated at 24 h, 48 h or 72 h with bortezomib or compound **27** by adding 2 μL 100‐fold concentrated proteasome inhibitor in DMSO to give a final concentration of 25 μM or 50 μM. Incubation times were 24 h, 48 h and 72 h. Survival of the embryos was determined by visual analysis of heart‐beat. For recording the touch evoked escape response, embryos were transferred to a microscopy slide with cavity that is placed on a SL‐300 LED Soft Light (Dörr, Neu‐Ulm, Germany) under a MotionBlitz EoSens mini1‐1 MC1370 high speed camera (Mikrotron, Unterschleißheim, Germany, Figure S18). An escape response was triggered by a touch stimulus at the trunk of embryos using a small sewing needle, which is slightly blunted. For each embryo, a maximum of 50 stimuli was applied. Two (in case where only 2 of 4 embryos survived) or three embryos per incubation time and concentration were assayed. No escape response was determined if only one embryo was alive at 96 hpf. Movement was recorded at 96 hpf at 500 frames per second for 3 seconds using the MotionBlitzDirector 2 software. Determination of the amplitude α defined by the body angle spanned between the head, yolk and tail using a MATLAB‐based software tool and plotted using GraphPad Prism 7.02. Statistical analysis was performed using the two‐sided, unpaired t‐test (Parametric test, α=0.05, 95 % CI) under the Null hypothesis (H^0^) that there is no difference in means after treatment and the alternate hypothesis that there is a difference (H^1^) using GraphPad Prism 7.02. These data were used to obtain the amplitude and length of the C‐bend, total number of movements and response duration. Detailed statistical parameters including 95 % confidence intervals and R^2^ values are given in the supporting information (Tables S4, S5; Figures S19–S23).

***Fluorescence characterization of BODIPY‐labeled ketoamide proteasome inhibitors***. Absorption and fluorescence emission spectra have been determined in acetonitrile at 10 μM using a Tecan M1000 Pro microplate reader (Figure S24).

***Imaging of embryos treated with BODIPY‐conjugated PI***. To analyze the location, where fluorescent proteasome inhibitors are mounted and metabolized within the zebrafish embryo, dechorionated embryos were decollated into 96‐well‐plates containing concentrations of 25 μM inhibitor in E3 media. 96 hours post fertilization and 72 hours post the start of embryo‘s incubation in the presence of fluorescent inhibitor, the embryos were placed under an AxioScope A1 fluorescence microscope equipped with a Nuance Fx multispectral imaging system. Untreated embryos were investigated using the same exposure time to evaluate the pictures. For a clear detection under the microscope, the embryos were anaesthetized with tricaine for 3 minutes (Figure S25).

## Supporting Information

Synthesis procedures, compound characterization data, NMR spectra, dose‐response curves, characterization of BODIPY‐conjugated ketoamides, *Danio rerio* toxicity and escape response assay raw data and statistics, molecular modeling details.

## Author Contributions

The manuscript was written through contributions of all authors. All authors have given approval to the final version of the manuscript.

## Abbreviations

CP: 20 S proteasome core particle
yCP: yeast proteasome core particle
cCP: constitutive proteasome core particle
iCP: immunoproteasome core particle
PI: proteasome inhibitor
MOE: Molecular Operating Environment

## Conflict of interest

The authors declare no conflict of interest.

## Supporting information

As a service to our authors and readers, this journal provides supporting information supplied by the authors. Such materials are peer reviewed and may be re‐organized for online delivery, but are not copy‐edited or typeset. Technical support issues arising from supporting information (other than missing files) should be addressed to the authors.

Supplementary
