# Supplementary material for: Cell‐Based Optimization of Covalent Reversible Ketoamide Inhibitors Bridging the Unprimed to the Primed Site of the Proteasome β5 Subunit
Source: ChemMedChem. 2019 Nov 12;14(23):2005–22. doi: 10.1002/cmdc.201900472 (PMC6916368; doi:10.1002/cmdc.201900472)
Supplement: Supplementary file 1 — Supplementary [file CMDC-14-2005-s001.pdf]

CHEMMEDCHEM

Supporting Information

© Copyright Wiley-VCH Verlag GmbH & Co. KGaA, 69451 Weinheim, 2019

Cell-Based Optimization of Covalent Reversible Ketoamide Inhibitors Bridging the Unprimed to the Primed Site of the Proteasome β5 Subunit

Daniel Stubba, Dennis Bensinger, Janika Steinbacher, Lilia Proskurjakov, Álvaro Salcedo Gómez, Uwe Schmidt, Stefan Roth, Katja Schmitz, and Boris Schmidt*© 2019 The Authors. Published by Wiley-VCH Verlag GmbH & Co. KGaA. This is an open access article under the terms of the Creative Commons Attribution License, which permits use, distribution and reproduction in any medium, provided the original work is properly cited.

Supporting Information

Content of Supporting Information

Content of Supporting Information S1

1. Organic Chemistry

a. General procedures S2
b. Synthesis of phenoxy-substituted aromatic isocyanides (**47**–**49**) S5
c. Synthesis of methyl-substituted phenoxy-isocyanides (**74**–**78**) S10
d. Synthesis of intermediates for *N*-protected peptide aldehydes (**22**–**25**) S22
e. Synthesis of intermediates for BODIPY labeled α-ketoamides (**30**–**32**) S26
f. Compound purity as determined by HPLC S31

2. Biochemistry

a. Summary of proteasome and cytotoxicity assays S32
b. Dose-response curves S33
c. Danio rerio toxicity assay S42
d. Escape response assay S43
e. BODIPY characterization S54

3. Molecular Modelling

a. Structural analysis of BSc4999 (**7**) and covalent redocking using Docktite S55
b. Energy-minimized binding poses of phenoxy and P4-substituted ketoamides S57
c. Covalent docking of BSc5376 (**27**) using Docktite S60

4. Appendix

Spectra S63
References S92

1. Organic chemistry

a. General procedures

Synthesis of diphenyl ethers via aromatic nucleophilic substitution (GP1). Fluorobenzene (1.0 equiv) and phenol (1.0 equiv) were dissolved in DMSO and K_2CO_3 (2.0 equiv) was added. The mixture was heated in a sealed tube for 24 h at 100°C. Completion of the reaction was monitored by HPLC and TLC. Then water was added and the mixture was extracted with MTBE (3x). The combined organic extracts were dried over Na_2SO_4 and the solvent was evaporated under reduced pressure.

Reduction of aromatic nitro-compounds to amines or Cbz-deprotection (GP2). A solution of the corresponding nitro-compound in MeOH was added to 10 wt% 10% Pd/C. The mixture was stirred overnight under an atmosphere of hydrogen. After completion of the reaction, the mixture was filtered through a pad of celite and the solvent was removed under reduced pressure.

Synthesis of aromatic formamides (GP3). The corresponding aromatic amine (1.0 equiv) was dissolved in PhMe and HCOOH (2.0 equiv) was added. The mixture was heated in a sealed tube for 24 h at 110°C. Completion of the reaction was monitored by TLC and HPLC. After completion AcOEt was added and the organic layer was washed with 0.1 N aq HCl, sat. aq. $NaHCO_3$ and sat. aq. NaCl. The organic extract was dried over Na_2SO_4 and the solvent was evaporated under reduced pressure.

Synthesis of aromatic isocyanides (GP4). The corresponding aromatic formamide (1.0 equiv) and NEt_3 (10 equiv) were dissolved in CH_2Cl_2 and cooled to 0°C. At this temperature $POCl_3$ (2.1 equiv) was added and the mixtures was stirred for 24 h at rt. Completion of the reaction was monitored by TLC and HPLC. Due to the instability of the product, the workup was done fast and with cooled solvents. After completion CH_2Cl_2 was added and the organic layer was washed with sat. aq. $NaHCO_3$ (2x). The organic layer was dried over Na_2SO_4 and the solvent was evaporated under reduced pressure. Purification was done by column chromatography of the residue on silica gel (cyclohexane / AcOEt 1:1) and the obtained isocyanides were directly used in the next step due to fast hydrolysis on air. It should be mentioned that all actions should be performed in a well-ventilated hood due to obnoxious odor and high toxicity of the compounds.

Synthesis of α-Ketophenylamides (GP5). The corresponding peptidic aldehyde (1.0 equiv) and aromatic isocyanide (1.5 equiv) were dissolved in a minimal amount of CH_2Cl_2 and cooled to 0°C under an atmosphere of argon. A mixture of trifluoroacetic acid (2.0 equiv) in CH_2Cl_2 was added dropwise and the mixture was stirred for

2 h at 0°C and 24 h at room temperature. Completion of the reaction was monitored by HPLC and TLC. CH_2Cl_2 was added and the organic layer was washed with 0.1 N aq. HCl (3x), sat. aq. $NaHCO_3$ (3x) and sat. aq. NaCl. The organic extract was dried over Na_2SO_4 and the solvent was evaporated under reduced pressure. The resulting solid was dissolved in DMSO and IBX (2.0 equiv) was added. The reaction mixture was stirred 24 h at rt. After completion CH_2Cl_2 was added and the mixture was washed with water (3x), sat. aq. $NaHCO_3$ (3x) and sat. aq. NaCl. The organic extract was dried over Na_2SO_4 and the solvent was evaporated under reduced pressure. Purification was done by column chromatography of the residue on silica gel (cyclohexane / AcOEt 2:1).

Nitration of anilines (GP6). The corresponding aromatic amine (1.0 equiv) was dissolved in conc. sulphuric acid and fuming nitric acid (1.3 equiv) was added dropwise at 0°C. The mixture was warmed to rt, stirred for 12 h, poured onto crushed ice and filtered. The filter cake was washed with sat. aq. $NaHCO_3$ solution and extracted with AcOEt (3x). The combined organic extracts were dried over Na_2SO_4 and the solvent was evaporated under reduced pressure.

Arylation of phenols with Ph_2ICl (GP7). Potassium *t*-butoxide was suspended in dry THF at 0°C and the corresponding phenol (1.0 equiv) was added. The mixture was stirred 15 min. and Ph_2ICl (1.2 equiv) was added and stirred overnight at rt. Completion of the reaction was monitored by HPLC and TLC. The mixture was cooled to 0°C and quenched with water. The aqueous layer was extracted with CH_2Cl_2 (3x), the combined organic extracts were washed with water, dried over Na_2SO_4 and the solvent was evaporated under reduced pressure. The crude product was purified by column chromatography of the residue on silica gel (cyclohexane / AcOEt 100:1).

Synthesis of phenols from anilines (GP8). The corresponding aniline (1.0 equiv) was suspended in water, cooled to 0°C and conc. sulphuric acid (13 mL) was added. $NaNO_3$ (1.3 equiv) was dissolved in water and added to the reaction mixture. In a separate flask 10 mL of water and 1.1 mL of conc. sulphuric acid were mixed and heated to reflux. The aniline solution was added dropwise to the refluxing mixture and stirred for 15 min. The reaction mixture was cooled to rt and extracted with MTBE (3x 35 mL). The combined organic extracts were extracted with 10% aq. NaOH (3x 35 mL) and the combined aqueous extracts were acidified with aq. HCl and extracted with MTBE (3x 35 mL). The combined organic extracts were dried over Na_2SO_4 and the solvent was evaporated under reduced pressure. If necessary, purification was done by column chromatography of the residue on silica gel (cyclohexane / AcOEt).

Peptide synthesis via HATU (GP9). The carboxylic acid (1.0 equiv) was dissolved in DMF and HATU (1.1 equiv) was added. The mixture was stirred for 20 min. at rt before the amine (1.0 equiv) and DIPEA (2.9 equiv) were added. The reaction mixture was stirred overnight at rt. Completion of the reaction was monitored by HPLC and TLC. CH_2Cl_2 was added and the organic layer was washed with 0.1 N aq. HCl (5x), 0.1 N aq. NaOH (3x) and sat. aq. NaCl. The organic extract was dried over Na_2SO_4 and the solvent was evaporated under reduced pressure. If necessary, purification was done by column chromatography of the residue on silica gel.

Peptide synthesis via EDC and HOBt (GP10). The carboxylic acid (1.0 equiv) was dissolved in CH_2Cl_2 and EDC • HCl (1.0 equiv) and HOBt • H_2O (1.2 equiv) were added. The mixture was stirred for 20 min. at rt before the amine (1.0 equiv) and NEt_3 (1.5 equiv) were added. The reaction mixture was stirred overnight at rt. Completion of the reaction was monitored by HPLC and TLC. CH_2Cl_2 was added and the organic layer was washed with 0.1 N aq. HCl (5x), 0.1 N aq. NaOH (3x) and sat. aq. NaCl. The organic extract was dried over Na_2SO_4 and the solvent was evaporated under reduced pressure. If necessary, purification was done by column chromatography of the residue on silica gel.

Oxidation of alcohols via IBX (GP11). The corresponding peptidic alcohol (1.0 equiv) was dissolved in DMSO and IBX (1.5 equiv) was added. The mixture was stirred overnight at rt. Completion of the reaction was monitored by HPLC and TLC. CH_2Cl_2 was added and the organic layer was washed with water (3x), sat. aq. $NaHCO_3$ (3x) and sat. aq. NaCl. The organic extract was dried over Na_2SO_4 and the solvent was evaporated under reduced pressure.

b. Synthesis of phenoxy-substituted aromatic isocyanides (47 – 49)

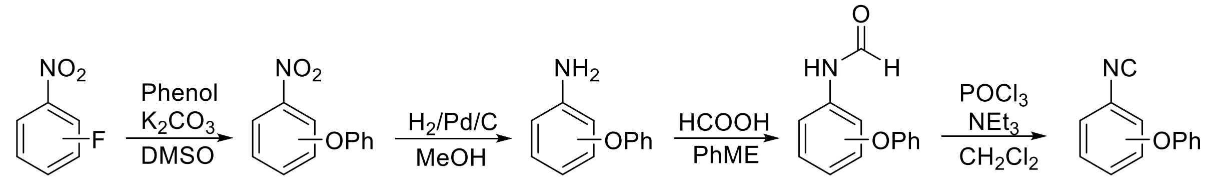

35 2-subst **38** 2-subst **41** 2-subst **44** 2-subst **47** 2-subst
36 3-subst **39** 3-subst **42** 3-subst **45** 3-subst **48** 3-subst
37 4-subst **40** 4-subst **43** 4-subst **46** 4-subst **49** 4-subst

Scheme S1: Synthesis of phenoxy-substituted isocyanides.

1-Nitro-2-phenoxybenzene (38)

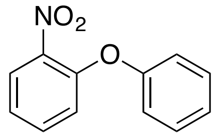

1-Fluoro-2-nitrobenzene (748 µL, 1.00 g, 7.08 mmol, 1.0 equiv), phenol (666 mg, 7.08 mmol, 1.0 equiv) and K_2CO_3 (1.96 g, 14.2 mmol, 2.0 equiv) were reacted according to GP1 to afford 1-nitro-2-phenoxybenzene **38** (1.50 g, 6.97 mmol, 98%) as a pale-yellow oil.

R*f*= 0.69 (cyclohexane / AcOEt = 5:1).

HPLC (254 nm, VWD): t_R = 6.91 min.

^{1}H-NMR (300 MHz, $CDCl_3$): δ = 7.95 (dd, J = 8.1, 1.7 Hz, 1H), 7.55 – 7.46 (m, 1H), 7.42 – 7.34 (m, 2H), 7.23 – 7.15 (m, 2H), 7.10 – 6.99 (m, 3H).

^{13}C-NMR (75 MHz, $CDCl_3$): δ = 155.9, 150.9, 134.2, 130.2, 125.9, 124.7, 123.2, 120.6, 119.4.

1-Nitro-3-phenoxybenzene (39)

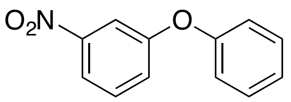

1-Fluoro-3-nitrobenzene (1.51 mL, 2.00 g, 14.1 mmol, 1.0 equiv), phenol (1.33 g, 14.1 mmol, 1.0 equiv) and K_2CO_3 (3.92 g, 28.3 mmol, 2.0 equiv) were reacted according to GP1, at 150°C, to afford 1-nitro-3-phenoxybenzene **39** (2.96 g, 13.7 mmol, 97%) as a brown oil.

R*f*= 0.47 (cyclohexane / AcOEt = 5:1).

HPLC (254 nm, VWD): t_R = 7.47 min.

^{1}H-NMR (300 MHz, $CDCl_3$): δ = 7.93 (ddd, J = 8.2, 2.2, 1.0 Hz, 1H), 7.79 (t, J = 2.2 Hz, 1H), 7.48 (t, J = 8.2 Hz, 1H), 7.45 – 7.37 (m, 2H), 7.32 (ddd, J = 8.3, 2.5, 1.0 Hz, 1H), 7.25 – 7.18 (m, 1H), 7.11 – 7.04 (m, 2H).

13C-NMR (75 MHz, $CDCl_3$): δ = 158.7, 155.7, 149.5, 130.4, 130.4, 125.0, 124.3, 119.9, 117.8, 113.0.

1-Nitro-4-phenoxybenzene (40)

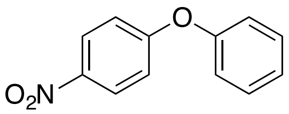

1-Fluoro-4-nitrobenzene (0.75 mL, 1.00 g, 7.09 mmol, 1.0 equiv), phenol (0.67 g, 7.09 mmol, 1.0 equiv) and K_2CO_3 (0.98 g, 14.2 mmol, 2.0 equiv) were reacted according to GP1 to afford 1-nitro-4-phenoxybenzene **40** (1.45 g, 6.72 mmol, 95%) as a pale yellow oil.

R_f = 0.52 (cyclohexane / AcOEt = 5:1).

HPLC (254 nm, VWD): t_R = 7.34 min.

1H-NMR (500 MHz, $CDCl_3$): δ = 8.24 – 8.18 (m, 2H), 7.48 – 7.41 (m, 2H), 7.28 – 7.25 (m, 1H), 7.12 – 7.08 (m, 2H), 7.04 – 7.00 (m, 2H).

13C-NMR (126 MHz, $CDCl_3$): 163.5, 154.9, 142.8, 130.5, 126.1, 125.5, 120.7, 117.3.

2-Phenoxyaniline (41)

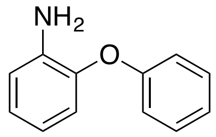

1-Nitro-2-phenoxybenzene (2.90 g, 13.5 mmol, 1.0 equiv) in 40 mL of MeOH and 10% Pd/C (290 mg, 10 wt%) were reacted according to GP2 to afford 2-Phenoxyaniline **41** (2.35 g, 12.7 mmol, 94%) as a pale brown oil.

R_f = 0.46 (cyclohexane / AcOEt = 5:1).

HPLC (254 nm, VWD): t_R = 2.30 min.

1H-NMR (500 MHz, $CDCl_3$): δ = 7.34 – 7.29 (m, 2H), 7.06 (m, 1H), 7.02 – 6.97 (m, 3H), 6.89 (dd, J = 8.0, 1.4 Hz, 1H), 6.84 (dd, J = 7.9, 1.6 Hz, 1H), 6.76 – 6.71 (m, 1H), 3.69 (s, 2H).

13C-NMR (75 MHz, $CDCl_3$): δ = 157.6, 143.3, 138.7, 129.8, 125.0, 122.8, 120.4, 119.0, 117.3, 116.7.

3-Phenoxyaniline (42)

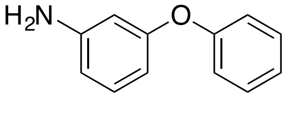

1-Nitro-3-phenoxybenzene (2.82 g, 13.1 mmol, 1.0 equiv) in 100 mL of MeOH and 10% Pd/C (282 mg, 10 wt%) were reacted according to GP2 to afford 3-phenoxyaniline **42** (2.44 g, 13.1 mmol, quant.) as a pale brown oil.

R_f = 0.20 (cyclohexane / AcOEt = 5:1).

HPLC (254 nm, VWD): t_R = 1.80 min.

^{1}H NMR (300 MHz, $CDCl_3$): δ = 7.38 – 7.30 (m, 2H), 7.17 – 6.97 (m, 4H), 6.45 – 6.39 (m, 2H), 6.34 (t, *J* = 2.2 Hz, 1H), 3.63 (s, 2H).

^{13}C NMR (75 MHz, $CDCl_3$): δ = 158.6, 157.3, 148.1, 130.5, 129.8, 123.3, 119.2, 110.2, 109.0, 105.7.

4-Phenoxyaniline (43)

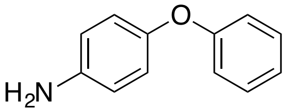

1-Nitro-4-phenoxybenzene (1.32 g, 6.15 mmol, 1.0 equiv) in 50 mL of MeOH and 10% Pd/C (132 mg, 10 wt%) were reacted according to GP2 to afford 4-phenoxyaniline **43** (976 mg, 5.27 mmol, 86%) as a pale brown solid.

R_f = 0.14 (cyclohexane / AcOEt = 5:1).

HPLC (254 nm, VWD): t_R = 1.92 min.

^{1}H NMR (500 MHz, $CDCl_3$): = δ 7.28 (t, *J* = 8.0 Hz, 2H), 7.02 (t, *J* = 7.4 Hz, 1H), 6.94 (d, *J* = 8.0 Hz, 2H), 6.88 (d, *J* = 8.7 Hz, 2H), 6.68 (d, *J* = 8.7 Hz, 2H), 3.52 (s, 2H).

^{13}C NMR (126 MHz, $CDCl_3$): δ 159.0, 148.8, 142.8, 129.7, 122.2, 121.2, 117.4, 116.4.

***N*-(2-phenoxyphenyl)formamide (44)**

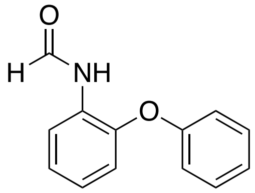

2-Phenoxyaniline (598 mg, 3.23 mmol, 1.0 equiv) in 5 mL of PhMe and formic acid (243 µL, 297 mg, 6.46 mmol, 2.0 equiv) were reacted according to GP3 to afford *N*-(2-phenoxyphenyl)formamide **44** (629 mg, 2.95 mmol, 91%) as a pale brown oil that crystallizes upon standing.

R_f = 0.20 (cyclohexane / AcOEt = 5:1).

HPLC (254 nm, VWD): t_R = 5.44 min.

^{1}H-NMR (300 MHz, $CDCl_3$): δ = 8.76 – 8.80 (d, 1H, H_{trans}, J = 11.5 Hz), 8.48 (d, 1H, H_{cis}, J = 1.5 Hz), 8.44 (br s, 1H), 7.84 (br s, 1H), 7.29 – 7.39 (m, 2H), 6.85 – 7.17 (m, 6H) (1:1.5 mixture of rotamers).

^{13}C-NMR (75 MHz, $CDCl_3$): δ = 161.5, 159.0, 156.4, 145.5, 130.1, 124.7, 124.3, 124.1, 121.5, 118.6, 118.0 (1:1.5 mixture of rotamers).

***N*-(3-phenoxyphenyl)formamide (45)**

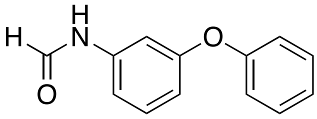

3-Phenoxyaniline (765 mg, 4.13 mmol, 1.0 equiv) in 5 mL of PhMe and formic acid (309 µL, 377 mg, 8.26 mmol, 2.0 equiv) were reacted according to GP3 to afford *N*-(3-phenoxyphenyl)formamide **45** (843 mg, 3.95 mmol, 97%) as a pale brown oil that crystallizes upon standing.

R_f = 0.12 (cyclohexane / AcOEt = 3:1).

HPLC (254 nm, VWD): t_R = 5.34 min.

^{1}H-NMR (300 MHz, $CDCl_3$): δ = 8.62 – 8.66 (d, 1H, H_{trans}, J = 11.2 Hz), 8.37 (m, 1H), 8.30 (d, 1H, H_{cis}, J = 1.6 Hz, 7.56 (br s, 1H), 6.71 – 7.37 ppm (m, 9H) (1:1 mixture of rotamers).
^{13}C-NMR (75 MHz, $CDCl_3$): δ = 162.6, 159.2, 158.9, 158.1, 156.8, 156.4, 138.3, 131.0, 130.3, 130.1, 129.9, 124.1, 123.7, 119.5, 119.3, 115.0, 114.8, 113.1, 110.6, 108.9 (1:1 mixture of rotamers).

***N*-(4-phenoxyphenyl)formamide (46)**

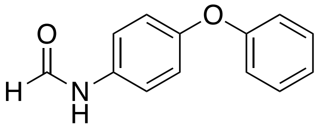

4-Phenoxyaniline (942 mg, 5.08 mmol, 1.0 equiv) in 5 mL of PhMe and formic acid (384 µL, 468 mg, 10.2 mmol, 2.0 equiv) were reacted according to GP3 to afford *N*-(4-phenoxyphenyl)formamide **46** (998 mg, 4.68 mmol, 92%) as a pale brown oil.

R_f = 0.14 (cyclohexane / AcOEt = 3:1).

HPLC (254 nm, VWD): t_R = 5.25 min.

^{1}H-NMR (300 MHz, $CDCl_3$): δ = 8.58 – 8.62 (d, 1H, H_{trans}, J = 11.5 Hz), 8.38 (br s, 1H), 8.35 ppm (d, 1H, H_{cis}, J =1.7 Hz), 7.57 (br s, 1H), 7.49 – 7.52 (m, 1H), 7.30 – 7.37 (m, 2H), 6.97 – 7.02 (m, 4H) (1:1 mixture of rotamers).

^{13}C-NMR (75 MHz, $CDCl_3$): δ = 163.0, 159.1, 157.5, 157.2, 155.1, 154.1, 132.4, 132.1, 130.0, 129.9, 123.6, 123.3, 121.9, 121.2, 120.2, 119.7, 118.8, 118.7 (1:1 mixture of rotamers).

1-isocyano-2-phenoxybenzene (47)

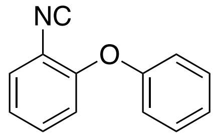

N-(2-phenoxyphenyl)formamide (500 mg, 2.34 mmol, 1.0 equiv) in 20 mL of CH_2Cl_2, NEt_3 (3.24 mL, 2.38 g, 23.4 mmol, 10 equiv) and $POCl_3$ (454 µL, 753 mg, 4.91 mmol, 2.1 equiv) were reacted according to GP4 to afford 1-isocyano-2-phenoxybenzene **47** (388 mg, 1.98 mmol, 85%) as a pale yellow oil with a very intense smell. The product was directly used in the next step without further characterization.

R*f* = 0.73 (cyclohexane / AcOEt = 1:1).

HPLC (254 nm, VWD): t_R = 6.93 min.

1-isocyano-3-phenoxybenzene (48)

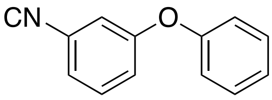

N-(3-phenoxyphenyl)formamide (819 mg, 3.84 mmol, 1.0 equiv) in 50 mL of CH_2Cl_2, NEt_3 (5.32 mL, 3.89 g, 38.4 mmol, 10 equiv) and $POCl_3$ (746 µL, 1.25 g, 8.06 mmol, 2.1 equiv) were reacted according to GP4 to afford 1-isocyano-3-phenoxybenzene **48** (672 mg, 3.44 mmol, 90%) as a pale yellow oil with a very intense smell. The product was directly used in the next step without further characterization.

R*f* = 0.70 (cyclohexane / AcOEt = 1:1).

HPLC (254 nm, VWD): t_R = 7.08 min.

1-isocyano-4-phenoxybenzene (49)

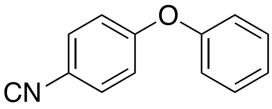

N-(4-phenoxyphenyl)formamide (1.00 g, 4.69 mmol, 1.0 equiv) in 50 mL of CH_2Cl_2, NEt_3 (6.50 mL, 4.75 g, 46.9 mmol, 10 equiv) and $POCl_3$ (921 µL, 1.53 g, 9.85 mmol, 2.1 equiv) were reacted according to GP4 to afford 1-isocyano-2-phenoxybenze **49** (782 mg, 4.01 mmol, 85%) as a pale yellow oil with a very intense smell. The product was directly used in the next step without further characterization.

R*f* = 0.78 (cyclohexane / AcOEt = 1:1).

HPLC (254 nm, VWD): t_R = 7.07 min.

c. Synthesis of methyl-substituted phenoxy-isocyanides (74 – 78)

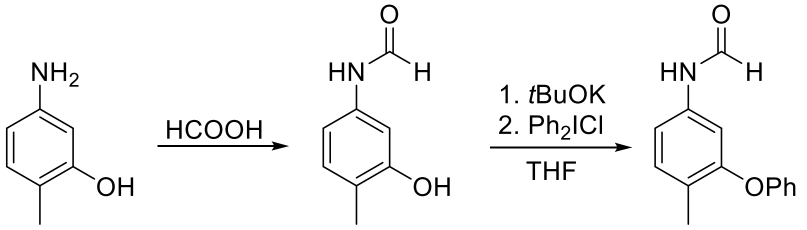

50 **51** **52**

Scheme S2: Synthesis of *N*-(4-Methyl-3-phenoxyphenyl)formamide.

***N*-(3-Hydroxy-4-methylphenyl)formamide (51)**

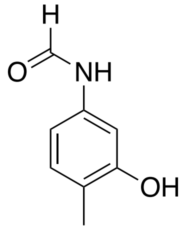

5-Amino-2-methylphenol (2.00 g, 16.2 mmol, 1.0 equiv) and formic acid (920 µL 1.12 g, 24.3 mmol, 1.5 equiv) were stirred in a capped microwave vial at 80 °C overnight. After completion of the reaction AcOEt (40 mL) was added and the organic layer was washed with sat. aq. $NaHCO_3$ (2 x 20 mL) and sat. aq. NaCl (2 x 20 mL). The organic extract was dried over Na_2SO_4 and the solvent was evaporated under reduced pressure to afford *N*-(3-hydroxy-4-methylphenyl)formamide **51** (1.85 g, 12.3 mmol, 76%) as a brown solid.

R*f*= 0.29 (cyclohexane / AcOEt = 1:2).

HPLC (254 nm, VWD): t_R = 1.45 min.

^{1}H-NMR (500 MHz, DMSO-*d6*): δ = 9.92 (s, 1H), 9.41 – 9.25 (m, 1H), 8.19 – 8.17 (m, 1H), 7.23 – 7.19 (m, 1H), 6.98 – 6.93 (m, 1H), 6.84 – 6.81 (m, 1H), 2.05 (s, 3H) (1:1 mixture of rotamers).

^{13}C-NMR (126 MHz, DMSO-*d6*): δ = 162.2, 159.1, 155.9, 155.3, 136.8, 131.0, 130.3, 119.5, 119.1, 109.7, 108.0, 106.0, 104.8, 15.4 (1:1 mixture of rotamers).

***N*-(4-Methyl-3-phenoxyphenyl)formamide (52)**

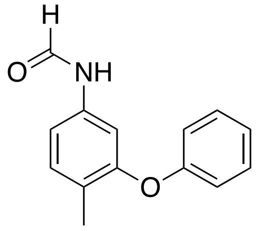

N-(3-Hydroxy-4-methylphenyl)formamide (500 mg, 3.31 mmol, 1.0 equiv), potassium *tert*-butoxide (409 mg, 3.64 mmol, 1.1 equiv) and Ph_2ICl (1.26 g, 3.97 mmol, 1.2 equiv) in 13 mL of THF were reacted according to GP7.

Instead mentioned in GP7 a mixture of cyclohexane / AcOEt (2:1) was used for purification by column chromatography to afford *N*-(4-methyl-3-phenoxyphenyl)formamide **52** (499 mg, 2.20 mmol, 66%) as a brown oil.
R*f*= 0.21 (cyclohexane / AcOEt = 2:1).

HPLC (254 nm, VWD): t_R = 5.65 min.

^{1}H-NMR (500 MHz, $CDCl_3$): δ = 8.55 (d, J = 11.4 Hz, 0.5H), 8.29 (d, J = 1.8 Hz, 0.5H), 7.72 – 7.65 (m, 0.5H), 7.36 – 7.27 (m, 2.5H), 7.20 (dd, J = 10.0, 8.2 Hz, 1H), 7.16 (s, 0.5H), 7.12 – 7.08 (m, 0.5H), 7.08 – 7.03 (m, 1H), 6.95 – 6.89 (m, 2H), 6.77 (dd, J = 8.1, 2.3 Hz, 0.5H), 6.59 (d, J = 2.2 Hz, 0.5H), 2.23 (s, 1.5H), 2.21 (s, 1.5H) (1:1 mixture of rotamers).

^{13}C-NMR (126 MHz, $CDCl_3$): δ = 162.3, 158.8, 157.6, 157.2, 155.8, 154.9, 135.9, 135.7, 132.5, 131.8, 130.0, 129.9, 126.9, 126.6, 123.3, 122.9, 118.0, 117.7, 115.7, 114.4, 111.6, 110.2, 15.8 (1:1 mixture of rotamers).

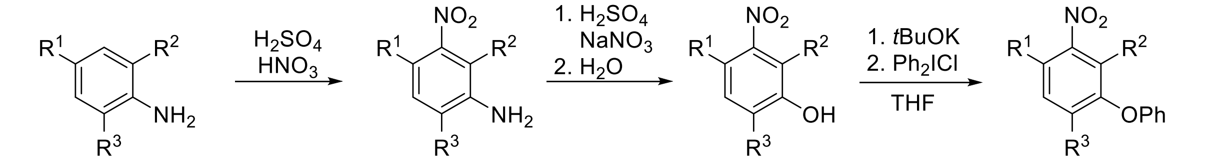

53 R^2 = H; R^1,R^3 = Me
54 R^1 = H; R^2,R^3 = Me

55 R^2 = H; R^1,R^3 = Me
56 R^1 = H; R^2,R^3 = Me
57 R^1,R^3 = H; R^2 = Me
58 R^2,R^3 = H; R^1 = Me

59 R^2 = H; R^1,R^3 = Me
60 R^1 = H; R^2,R^3 = Me
61 R^1,R^3 = H; R^2 = Me
62 R^2,R^3 = H; R^1 = Me

63 R^2 = H; R^1,R^3 = Me
64 R^1 = H; R^2,R^3 = Me
65 R^1,R^3 = H; R^2 = Me
66 R^2,R^3 = H; R^1 = Me

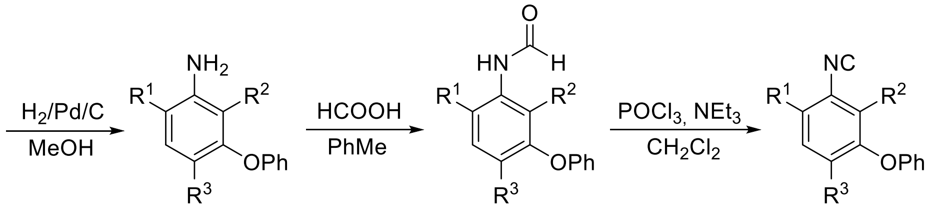

67 R^2 = H; R^1,R^3 = Me
68 R^1 = H; R^2,R^3 = Me
69 R^1,R^3 = H; R^2 = Me
70 R^2,R^3 = H; R^1 = Me

71 R^2 = H; R^1,R^3 = Me
72 R^1 = H; R^2,R^3 = Me
73 R^1,R^3 = H; R^2 = Me
74 R^2,R^3 = H; R^1 = Me

75 R^2 = H; R^1,R^3 = Me
76 R^1 = H; R^2,R^3 = Me
77 R^1,R^2 = H; R^3 = Me
78 R^1,R^3 = H; R^2 = Me
79 R^2,R^3 = H; R^1 = Me

Scheme S3: Synthesis of methyl-phenoxy-substituted isocyanides.

2,4-Dimethyl-5-nitroaniline (55)

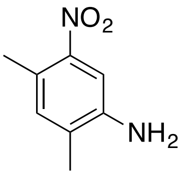

2,4-Dimethylaniline (4.85 g, 40.0 mmol, 1.0 equiv) in 20 mL of conc. H_2SO_4 and fuming nitric acid (2.40 mL, 3.20 g, 50.6 mmol, 1.3 equiv) were reacted according to GP6 to afford 2,4-dimethyl-5-nitroaniline **55** (5.73 g, 34.5 mmol, 86%) as an orange solid.

R*f* = 0.11 (cyclohexane / AcOEt = 5:1).

HPLC (254 nm, VWD): t_R = 2.34 min.

^{1}H-NMR (500 MHz, $CDCl_3$): δ = 7.33 (s, 1H), 6.97 (s, 1H), 3.73 (s, 3H), 2.46 (s, 3H), 2.18 (s, 3H).

^{13}C-NMR (126 MHz, $CDCl_3$): δ = 143.4, 134.5, 128.7, 123.4, 116.5, 110.4, 19.9, 17.4.

2,6-Dimethyl-3-nitroaniline (56)

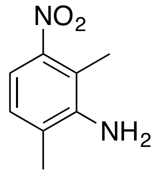

2,6-Dimethylaniline (4.85 g, 40.0 mmol, 1.0 equiv) in 20 mL of conc. H_2SO_4 and fuming nitric acid (2.40 mL, 3.20 g, 50.6 mmol, 1.3 equiv) were reacted according to GP6 to afford 2,6-dimethyl-3-nitroaniline **56** (5.37 g, 32.3 mmol, 81%) as a yellow solid.

R*f* = 0.17 (cyclohexane / AcOEt = 5:1).

HPLC (254 nm, VWD): t_R = 4.12 min.

^{1}H-NMR (500 MHz, $CDCl_3$): δ = 7.16 (d, J = 8.2 Hz, 1H), 7.01 (d, J = 8.2 Hz, 1H), 3.86 (br s, 2H), 2.28 (s, 3H), 2.23 (s, 3H).

2,4-Dimethyl-5-nitrophenol (59)

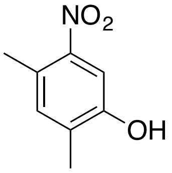

2,4-Dimethyl-5-nitroaniline (2.85 g, 17.2 mmol, 1.0 equiv) and $NaNO_3$ (1.54 g, 22.3 mmol, 1.3 equiv) were reacted according to GP8 to afford 2,4-dimethyl-5-nitrophenol **59** (1.34 g, 8.03 mmol, 47%) as a yellow solid.

R_f = 0.26 (cyclohexane / AcOEt = 5:1).

HPLC (254 nm, VWD): t_R = 4.58 min.

^{1}H-NMR (500 MHz, DMSO-*d_6*): δ = 10.04 (s, 1H), 7.42 (s, 1H), 7.17 (s, 1H), 2.39 (s, 3H), 2.17 (s, 3H).

^{13}C-NMR (126 MHz, DMSO-*d_6*): δ = 153.8, 146.3, 134.4, 131.5, 123.2, 109.7, 19.2, 15.7.

2,6-Dimethyl-3-nitrophenol (60)

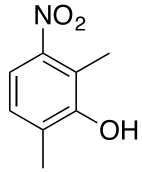

2,6-Dimethyl-3-nitroaniline (2.85 g, 17.2 mmol, 1.0 equiv) and $NaNO_3$ (1.54 g, 22.4 mmol, 1.3 equiv) were reacted according to GP8 to afford 2,6-dimethyl-3-nitrophenol **60** (1.53 g, 9.15 mmol, 53%) as a yellow solid.

R_f = 0.18 (cyclohexane / AcOEt = 5:1).

HPLC (254 nm, VWD): t_R = 4.49 min.

^{1}H-NMR (500 MHz, DMSO-*d_6*): δ = 9.13 (s, 1H), 7.31 (d, J = 8.3 Hz, 1H), 7.14 (d, J = 8.3 Hz, 1H), 2.29 (s, 3H), 2.25 (s, 3H).

^{13}C-NMR (126 MHz, DMSO-*d_6*): δ = 154.0, 148.8, 130.6, 127.9, 119.4, 114.9, 17.0, 12.1.

2-Methyl-3-nitrophenol (61)

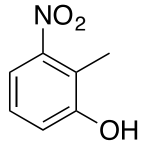

2-Methyl-3-nitroaniline (3.62 g, 23.8 mmol, 1.0 equiv) and $NaNO_3$ (2.13 g, 31.0 mmol, 1.3 equiv) were reacted according to GP8 to afford 2-methyl-3-nitrophenol **61** (2.46 g, 16.1 mmol, 67%) as a brown solid.

R_f = 0.35 (cyclohexane / AcOEt = 2:1).

HPLC (254 nm, VWD): t_R = 3.79 min.

^{1}H-NMR (500 MHz, DMSO-*d_6*): δ = 10.29 (s, 1H), 7.30 (dd, J = 8.1, 1.2 Hz, 1H), 7.22 (t, J = 8.1 Hz, 1H), 7.12 (dd, J = 8.1, 1.3 Hz, 1H), 2.22 (s, 3H).

4-Methyl-3-nitrophenol (62)

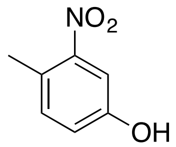

4-Methyl-3-nitroaniline (3.62 g, 23.8 mmol, 1.0 equiv) and $NaNO_3$ (2.13 g, 31.0 mmol, 1.3 equiv) were reacted according to GP8 to afford 4-methyl-3-nitrophenol **62** (1.51 g, 9.88 mmol, 42%) as a brown solid.

R*f*= 0.36 (cyclohexane / AcOEt = 2:1).

HPLC (254 nm, VWD): t_R = 3.58 min.

^{1}H-NMR (500 MHz, $CDCl_3$): δ= 7.48 (d, J = 2.7 Hz, 1H), 7.20 (d, J = 8.3 Hz, 1H), 7.01 (dd, J = 8.3, 2.7 Hz, 1H), 5.55 (s, 1H), 2.51 (s, 3H).

^{13}C-NMR (126 MHz, $CDCl_3$): δ= 154.3, 149.4, 133.9, 125.9, 120.9, 111.6, 19.8.

1,5-Dimethyl-2-nitro-4-phenoxybenzene (63)

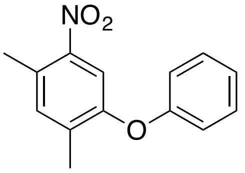

2,4-Dimethyl-5-nitrophenol (500 mg, 2.99 mmol, 1.0 equiv) in 13 mL of THF, potassium *tert*-butoxide (369 mg, 3.29 mmol, 1.1 equiv) and Ph_2ICl (1.14 g, 3.59 mmol, 1.2 equiv) were reacted according to GP7 to afford 1,5-dimethyl-2-nitro-4-phenoxybenzene **63** (665 mg, 2.73 mmol, 91%) as a yellow oil.
R*f*= 0.33 (cyclohexane / AcOEt = 50:1).
HPLC (254 nm, VWD): t_R = 7.87 min.

^{1}H-NMR (500 MHz, $CDCl_3$): δ= 7.52 (s, 1H), 7.38 – 7.33 (m, 2H), 7.20 (s, 1H), 7.16 – 7.11 (m, 1H), 6.97 – 6.94 (m, 2H), 2.57 (s, 3H), 2.32 (s, 3H).

^{13}C-NMR (126 MHz, $CDCl_3$): δ= 156.8, 153.5, 147.4, 136.1, 135.4, 130.2, 128.9, 123.9, 118.3, 114.9, 20.2, 16.4.

1,3-Dimethyl-4-nitro-2-phenoxybenzene (64)

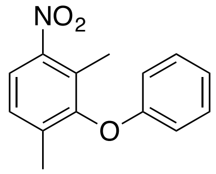

2,6-Dimethyl-3-nitrophenol (500 mg, 2.99 mmol, 1.0 equiv) in 13 mL of THF, potassium *tert*-butoxide (369 mg, 3.29 mmol, 1.1 equiv) and Ph_2ICl (1.14 g, 3.59 mmol, 1.2 equiv) were reacted according to GP7 to afford 1,3-dimethyl-4-nitro-2-phenoxybenzene **64** (605 mg, 2.50 mmol, 83%) as a colorless solid.

R_f= 0.19 (cyclohexane / AcOEt = 50:1).

HPLC (254 nm, VWD): t_R = 7.77 min.

^{1}H-NMR (500 MHz, $CDCl_3$): δ = 7.76 (d, J = 8.4 Hz, 1H), 7.30 – 7.27 (m, 2H), 7.23 (d, J = 8.4 Hz, 1H), 7.05 – 7.01 (m, 1H), 6.76 – 6.72 (m, 2H), 2.35 (s, 3H), 2.20 (s, 3H).

^{13}C-NMR (126 MHz, $CDCl_3$): δ = 157.2, 151.9, 149.3, 138.2, 130.1, 128.9, 128.1, 122.4, 121.4, 114.7, 17.2, 12.9.

2-Methyl-1-nitro-3-phenoxybenzene (65)

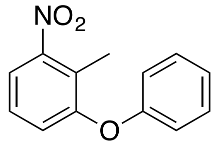

2-Methyl-3-nitrophenol (500 mg, 3.26 mmol, 1.0 equiv) in 13 mL of THF, potassium *tert*-butoxide (403 mg, 3.59 mmol, 1.1 equiv) and Ph_2ICl (1.24 g, 3.92 mmol, 1.2 equiv) were reacted according to GP7 to afford 2-methyl-1-nitro-3-phenoxybenzene **65** (575 mg, 2.51 mmol, 77%) as a yellow oil.

R_f= 0.22 (cyclohexane / AcOEt = 50:1).

HPLC (254 nm, VWD): t_R = 7.55 min.

^{1}H-NMR (500 MHz, DMSO-d_6): δ = 7.63 (dd, J = 8.1, 1.2 Hz, 1H), 7.38 – 7.34 (m, 2H), 7.28 – 7.24 (m, 1H), 7.16 – 7.12 (m, 1H), 7.10 (dd, J = 8.1, 1.2 Hz, 1H), 6.96 – 6.93 (m, 2H), 2.46 (s, 3H).

^{13}C-NMR (126 MHz, $CDCl_3$): δ = 157.0, 156.3, 151.5, 130.2, 127.0, 125.4, 123.9, 123.1, 119.4, 118.3, 12.2.

1-Methyl-2-nitro-4-phenoxybenzene (66)

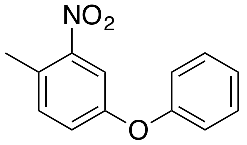

4-Methyl-3-nitrophenol (500 mg, 3.26 mmol, 1.0 equiv), potassium *tert*-butoxide (403 mg, 3.59 mmol, 1.1 equiv) and Ph_2ICl (1.24 g, 3.92 mmol, 1.2 equiv) in 13 mL of THF were reacted according to GP7 to afford 1-methyl-2-nitro-4-phenoxybenzene **66** (614 mg, 2.68 mmol, 82%) as a yellow oil.

R_f= 0.20 (cyclohexane / AcOEt = 50:1).

HPLC (254 nm, VWD): t_R = 7.52 min.

^{1}**H-NMR** (500 MHz, $CDCl_3$): δ = 7.58 (d, J = 2.6 Hz, 1H), 7.40 – 7.36 (m, 2H), 7.29 – 7.27 (m, 1H), 7.20 – 7.14 (m, 2H), 7.05 – 7.02 (m, 2H), 2.56 (s, 3H).

^{13}**C-NMR** (126 MHz, $CDCl_3$): δ = 156.3, 156.2, 149.8, 133.9, 130.3, 127.9, 124.6, 123.2, 119.5, 114.3, 19.9.

2,4-Dimethyl-5-phenoxyaniline (67)

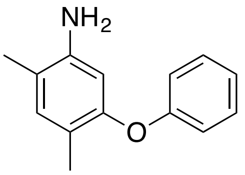

1,5-Dimethyl-2-nitro-4-phenoxybenzene (420 mg, 1.73 mmol, 1.0 equiv) in 40 mL of MeOH and 10% Pd/C (42 mg, 10 wt%) were reacted according to GP2 to afford 2,4-dimethyl-5-phenoxyaniline **67** (343 mg, 1.61 mmol, 93%) as a colorless oil.

$\mathbf{R}_f$ = 0.10 (cyclohexane / AcOEt = 10:1).

HPLC (254 nm, VWD): t_R = 3.24 min.

^{1}**H-NMR** (500 MHz, $CDCl_3$): δ = 7.30 – 7.26 (m, 2H), 7.03 – 6.99 (m, 1H), 6.92 – 6.88 (m, 3H), 6.30 (s, 1H), 3.65 – 3.30 (m, 2H), 2.14 (s, 3H), 2.09 (s, 3H).

^{13}**C-NMR** (126 MHz, $CDCl_3$): δ = 158.7, 153.2, 143.7, 133.4, 129.9, 122.3, 120.0, 118.9, 117.3, 107.6, 17.1, 15.6.

2,4-Dimethyl-3-phenoxyaniline (68)

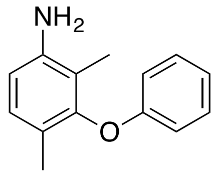

1,3-Dimethyl-4-nitro-2-phenoxybenzene (593 mg, 2.44 mmol, 1.0 equiv) in 50 mL of MeOH and 10% Pd/C (59 mg, 10 wt%) were reacted according to GP2 to afford 2,4-dimethyl-3-phenoxyaniline **68** (472 mg, 2.21 mmol, 91%) as a pale yellow oil.

$\mathbf{R}_f$ = 0.14 (cyclohexane / AcOEt = 10:1).

HPLC (254 nm, VWD): t_R = 3.05 min.

^{1}**H-NMR** (500 MHz, $CDCl_3$): δ = 7.26 – 7.22 (m, 2H), 6.97 – 6.93 (m, 1H), 6.91 (d, J = 8.1 Hz, 1H), 6.79 – 6.75 (m, 2H), 6.54 (d, J = 8.1 Hz, 1H), 3.54 (br s, 2H), 2.02 (s, 3H), 1.96 (s, 3H).

^{13}**C-NMR** (126 MHz, $CDCl_3$): δ = 158.3, 151.4, 144.2, 129.7, 128.7, 121.3, 116.5, 114.9, 112.2, 16.0, 10.3.

2-Methyl-3-phenoxyaniline (69)

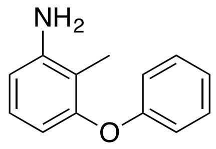

2-Methyl-1-nitro-3-phenoxybenzen (563 mg, 2.46 mmol, 1.0 equiv) in 50 mL of MeOH and 10% Pd/C (56 mg, 10 wt%) were reacted according to GP2 to afford 2-methyl-3-phenoxyaniline **69** (462 mg, 2.32 mmol, 94%) as a pale yellow oil.

R*f*= 0.14 (cyclohexane / AcOEt = 10:1).

HPLC (254 nm, VWD): t_R = 2.74 min.

^{1}H-NMR (500 MHz, DMSO-d_6): δ = 7.31 – 7.26 (m, 2H), 7.04 – 7.00 (m, 1H), 6.98 (t, J = 8.0 Hz, 1H), 6.93 – 6.89 (m, 2H), 6.52 (dd, J = 8.0, 1.1 Hz, 1H), 6.39 (dd, J = 8.0, 1.1 Hz, 1H), 3.70 (s, 2H), 2.06 (s, 3H).

^{13}C-NMR (126 MHz, $CDCl_3$): δ = 158.4, 155.0, 146.5, 129.7, 127.1, 122.2, 117.4, 114.7, 111.1, 110.5, 9.8.

2-Methyl-5-phenoxyaniline (70)

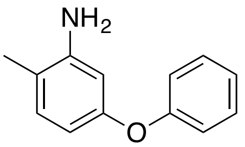

1-Methyl-2-nitro-4-phenoxybenzene (822 mg, 3.58 mmol, 1.0 equiv) in 50 mL of MeOH and 10% Pd/C (82 mg, 10 wt%) were reacted according to GP2 to afford 2-methyl-5-phenoxyaniline **70** (699 mg, 3.51 mmol, 98%) as a colorless solid.

R*f*= 0.20 (cyclohexane / AcOEt = 10:1).

HPLC (254 nm, VWD): t_R = 2.85 min.

^{1}H-NMR (500 MHz, $CDCl_3$): δ = 7.33 – 7.29 (m, 2H), 7.09 – 7.04 (m, 1H), 7.02 – 6.97 (m, 3H), 6.39 – 6.35 (m, 2H), 3.62 (s, 2H), 2.14 (s, 3H).

^{13}C-NMR (126 MHz, $CDCl_3$): δ = 157.9, 156.4, 145.9, 131.4, 129.7, 122.9, 118.7, 117.5, 109.3, 105.8, 16.8.

***N*-(2,4-Dimethyl-5-phenoxyphenyl)formamide (71)**

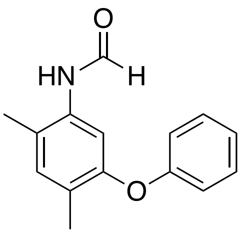

2,4-Dimethyl-5-phenoxyaniline (522 mg, 2.45 mmol, 1.0 equiv) in 5 mL of PhMe and formic acid (185 µL, 225 mg, 2.90 mmol, 2.0 equiv) were reacted according to GP3 to afford *N*-(2,4-dimethyl-5-phenoxyphenyl)-formamide **71** (557 mg, 2.31 mmol, 94%) as a brown oil.

R*f* = 0.18 (cyclohexane / AcOEt = 2:1).

HPLC (254 nm, VWD): t_R = 5.29 min.

^{1}H-NMR (500 MHz, $CDCl_3$): δ = 8.38 (d, *J* = 11.3 Hz, 0.5H), 8.36 (d, *J* = 1.8 Hz, 0.5H), 7.52 (s, 1H), 7.33 – 7.27 (m, 2H), 7.10 (s, 0.5H), 7.09 – 7.05 (m, 1H), 7.04 – 6.99 (m, 0.5H), 6.97 (s, 0.5H), 6.92 – 6.88 (m, 2H), 6.71 (s, 0.5H), 2.25 – 2.23(m, 3H), 2.19 (s, 1.5H), 2.17 (s, 1.5H). (1:1 mixture of rotamers).

^{13}C-NMR (126 MHz, $CDCl_3$): δ = 163.0, 158.9, 158.0, 157.7, 153.3, 152.6, 133.9, 133.6, 133.1, 129.8, 127.8, 127.5, 125.0, 124.4, 122.9, 122.4, 117.4, 117.1, 115.2, 112.4, 17.3, 17.1, 15.9, 15.7. (1:1 mixture of rotamers).

***N*-(2,4-Dimethyl-3-phenoxyphenyl)formamide (72)**

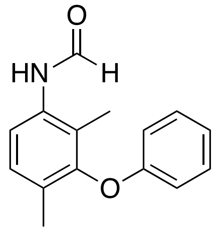

2,4-Dimethyl-3-phenoxyaniline (475 mg, 2.23 mmol, 1.0 equiv) in 5 mL of PhMe and formic acid (168 µL, 205 mg, 4.45 mmol, 2 equiv) were reacted according to GP3 to afford *N*-(2,4-dimethyl-3-phenoxyphenyl)-formamide **72** (530 mg, 2.20 mmol, 99%) as a brown solid.

R*f* = 0.21 (cyclohexane / AcOEt = 2:1).

HPLC (254 nm, VWD): t_R = 5.30 min.

^{1}H-NMR (500 MHz, $CDCl_3$): δ = 8.51 (d, *J* = 11.3 Hz, 0.5H), 8.44 (d, *J* = 1.8 Hz, 0.5H), 7.70 (d, *J* = 8.3 Hz, 0.5H), 7.53 (d, *J* = 9.5 Hz, 0.5H), 7.29 – 7.23 (m, 2H), 7.12 (d, *J* = 8.3 Hz, 1H), 7.04 – 6.95 (m, 2H), 6.77 – 6.72 (m, 2H), 2.13 – 2.09 (m, 3H), 2.08 – 2.05 (m, 3H) (1:1 mixture of rotamers).

^{13}**C-NMR** (126 MHz, $CDCl_3$): δ = 163.2, 159.1, 157.9, 157.7, 151.9, 151.2, 134.4, 133.8, 129.9, 129.9, 129.2, 129.0, 124.4, 123.3, 121.9, 121.7, 120.4, 118.2, 114.8, 114.7, 16.4, 11.1, 11.0 (1:1 mixture of rotamers).

***N*-(4-Methyl-3-phenoxyphenyl)formamide (73)**

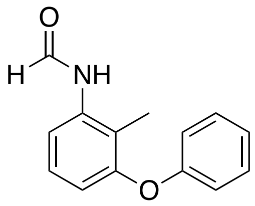

2-Methyl-3-phenoxyaniline (456 mg, 2.29 mmol, 1.0 equiv) in 5 mL of PhMe and formic acid (173 µL, 211 mg, 4.58 mmol, 2.0 equiv) were reacted according to GP3 to afford *N*-(4-methyl-3-phenoxyphenyl)formamide **73** (486 mg, 2.14 mmol, 93%) as a brown solid.

$\mathbf{R}_f$ = 0.20 (cyclohexane / AcOEt = 2:1).

HPLC (254 nm, VWD): t_R = 5.38 min.

^{1}**H-NMR** (500 MHz, $CDCl_3$): δ = 8.59 (d, J = 11.2 Hz, 0.5H), 8.48 (d, J = 1.8 Hz, 0.5H), 7.77 – 7.72 (m, 1H), 7.36 – 7.28 (m, 2H), 7.21 – 7.13 (m, 1H), 7.12 – 7.04 (m, 1H), 6.96 (d, J = 8.0 Hz, 1H), 6.94 – 6.89 (m, 2H), 6.81 – 6.75 (m, 1H), 2.23 – 2.17 (m, 3H) (1:1 mixture of rotamers).

^{13}**C-NMR** (126 MHz, $CDCl_3$): δ = 163.2, 159.1, 157.5, 155.9, 155.0, 136.7, 136.2, 130.0, 129.9, 127.4, 127.2, 123.2, 122.9, 121.8, 121.2, 118.9, 118.0, 117.7, 117.0, 116.1, 10.6 (1:1 mixture of rotamers).

***N*-(2-Methyl-5-phenoxyphenyl)formamide (74)**

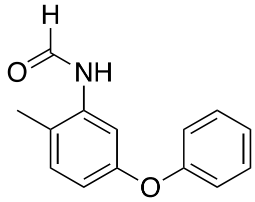

2-Methyl-5-phenoxyaniline (685 mg, 3.44 mmol, 1 equiv) in 7 mL of PhMe and formic acid (259 µL, 316 mg, 6.88 mmol, 2 equiv) were reacted according to GP3 to afford *N*-(2-methyl-5-phenoxyphenyl)formamide **74** (724 mg, 3.19 mmol, 93%) as a brown solid.

$\mathbf{R}_f$ = 0.22 (cyclohexane / AcOEt = 2:1).

HPLC (254 nm, VWD): t_R = 5.10 min.

^{1}**H-NMR** (500 MHz, $CDCl_3$): δ = 8.51 (d, J = 11.2 Hz, 0.5H), 8.42 (d, J = 1.8 Hz, 0.5H), 7.72 (d, J = 2.6 Hz, 0.5H), 7.69 – 7.62 (m, 0.5H), 7.38 – 7.28 (m, 2H), 7.19 – 6.97 (m, 4H), 6.85 – 6.73 (m, 2H)), 2.27 – 2.24 (m, 3H) (1:1 mixture of rotamers).

^{13}**C-NMR** (126 MHz, $CDCl_3$): δ = 162.9, 158.9, 157.4, 157.0, 156.5, 155.9, 136.1, 135.8, 132.3, 131.4, 130.0, 129.8, 123.6, 123.3, 122.8, 119.1, 118.8, 116.0, 116.0, 113.8, 110.7, 17.1 (1:1 mixture of rotamers).

1-Isocyano-2,4-Dimethyl-5-phenoxybenzene (75)

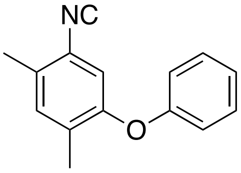

N-(2,4-Methyl-5-phenoxyphenyl)formamide (330 mg, 1.37 mmol, 1.0 equiv) in 20 mL of CH_2Cl_2, NEt_3 (1.90 mL, 1.37 g, 13.7 mmol, 10 equiv) and $POCl_3$ (316 µL, 531 mg, 3.30 mmol, 2.1 equiv) were reacted according to GP4 to afford 1-isocyano-2,4-dimethyl-5-phenoxybenzene **75** (260 mg, 1.16 mmol, 85%) as a pale yellow oil with a very intense smell. The product was directly used in the next step without further characterization.

R*f* = 0.72 (cyclohexane / AcOEt = 1:1).

HPLC (254 nm, VWD): t_R = 7.83 min.

1-Isocyano-2,4-Dimethyl-3-phenoxybenzene (76)

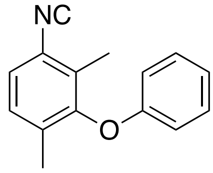

N-(2,4-Dimethyl-3-phenoxyphenyl)formamide (330 mg, 1.37 mmol, 1.0 equiv) in 20 mL of CH_2Cl_2, NEt_3 (1.90 mL, 1.37 g, 13.7 mmol, 10 equiv) and $POCl_3$ (316 µL, 531 mg, 3.42 mmol, 2.1 equiv) were reacted according to GP4 to afford 1-isocyano-2,4-dimethyl-3-phenoxybenzene **76** (282 mg, 1.26 mmol, 92%) as a pale green oil with a very intense smell. The product was directly used in the next step without further characterization.

R*f* = 0.68 (cyclohexane / AcOEt = 1:1).

HPLC (254 nm, VWD): t_R = 7.74 min.

4-Isocyano-1-methyl-2-phenoxybenzene (77)

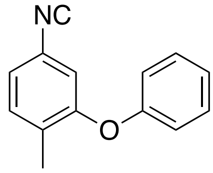

N-(4-methyl-3-phenoxyphenyl)formamide (300 mg, 1.32 mmol, 1.0 equiv) in 20 mL of CH_2Cl_2, NEt_3 (1.83 mL, 1.36 g, 13.2 mmol, 10 equiv) and $POCl_3$ (305 µL, 513 mg, 3.30 mmol, 2.1 equiv) were reacted according to GP4 to afford **4**-isocyano-1-methyl-2-phenoxybenzene **77** (238 mg, 1.14 mmol, 86%) as a pale green oil with a very strong smell. The product was directly used in the next step without further characterization.

R_f= 0.68 (cyclohexane / AcOEt = 1:1).

HPLC (254 nm, VWD): t_R = 7.39 min.

1-Isocyano-2-methyl-3-phenoxybenzene (78)

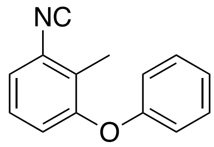

N-(4-Methyl-3-phenoxyphenyl)formamide (227 mg, 1.00 mmol, 1.0 equiv) in 15 mL of CH_2Cl_2, NEt_3 (1.38 mL, 1.01 g, 10.0 mmol, 10 equiv) and $POCl_3$ (194 µL, 326 mg, 2.10 mmol, 2.1 equiv) were reacted according to GP4 to afford **1**-isocyano-2-methyl-3-phenoxybenzene **78** (165 mg, 0.789 mmol, 79%) as a pale yellow oil with a very intense smell. The product was directly used in the next step without further characterization.

R_f= 0.67 (cyclohexane / AcOEt = 1:1).

HPLC (254 nm, VWD): t_R = 7.48 min.

2-Isocyano-1-methyl-4-phenoxybenzene (79)

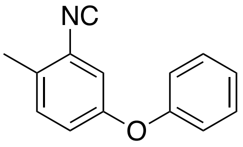

N-(2-Methyl-5-phenoxyphenyl)formamide (226 mg, 0.994 mmol, 1.0 equiv) in 15 mL of CH_2Cl_2, NEt_3 (1.38 mL, 1.01 g, 9.94 mmol, 10 equiv) and $POCl_3$ (193 µL, 324 mg, 2.09 mmol, 2.1 equiv) were reacted according to GP4 to afford 2-isocyano-1-methyl-4-phenoxybenzene **79** (167 mg, 0.798 mmol, 80%) as a pale yellow oil with a very intense smell. The product was directly used in the next step without further characterization.

R_f= 0.62 (cyclohexane / AcOEt = 1:1).

HPLC (254 nm, VWD): t_R = 7.44 min.

d. Synthesis of intermediates for *N*-substituted tripeptide aldehydes (22 – 25)

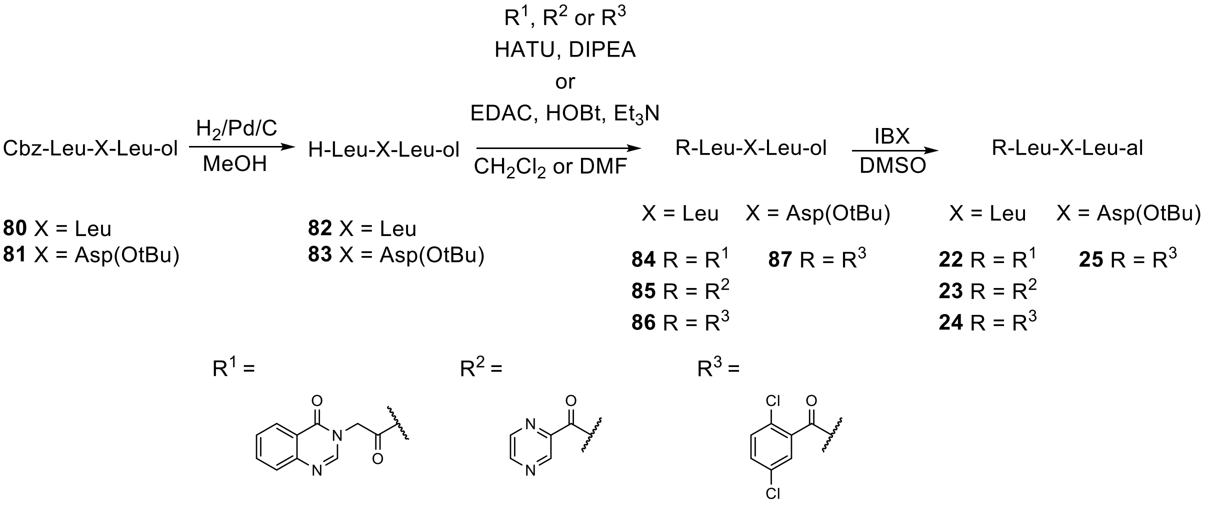

Scheme S4: Synthesis of *N*-substituted tripeptide aldehydes.

Cbz-Leu-Leu-Leu-ol **80**, Cbz-Leu-Asp(O*t*Bu)-Leu-ol **81** and 2-(4-oxoquinazolin-3(4*H*)-yl)acetic acid were synthesized as described before.[1-2]

H_2N-Leu-Leu-Leu-ol (82)

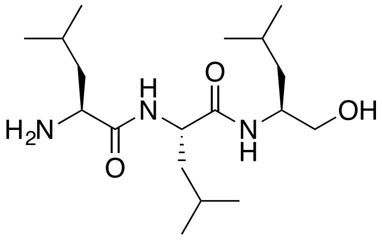

Cbz-Leu-Leu-Leu-ol (1.01 g, 2.30 mmol, 1.0 equiv) in 50 mL of MeOH and 10% Pd/C (101 mg, 10 wt%) were reacted according to GP2 to afford H_2N-Leu-Leu-Leu-ol **82** (750 mg, 2.18 mmol, 95%) as a colorless solid.

R_f= 0.32 (CH_2Cl_2 / MeOH = 10:1).

HPLC (205 nm, VWD): t_R = 1.48 min.

^{1}H-NMR (500 MHz, $CDCl_3$): δ = 7.68 (d, J = 8.3 Hz, 1H), 6.66 (d, J = 8.0 Hz, 1H), 4.41 – 4.33 (m, 1H), 4.00 – 3.93 (m, 1H), 3.65 (dd, J = 11.1, 3.5 Hz, 1H), 3.53 (dd, J = 11.1, 5.6 Hz, 1H), 3.38 (dd, J = 9.8, 3.9 Hz, 1H), 1.76 – 1.53 (m, 6H), 1.42 – 1.29 (m, 4H), 0.97 – 0.86 (m, 18H).

^{13}C-NMR (126 MHz, $CDCl_3$): δ = 176.4, 172.7, 66.0, 53.6, 51.8, 50.4, 44.1, 40.5, 40.1, 25.0, 23.5, 23.1, 22.4, 22.3, 21.5.

H_2N-Leu-Asp(O-*t*Bu)-Leu-ol (83)

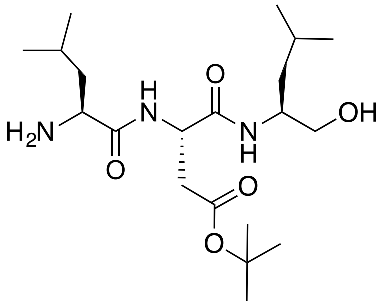

Cbz-Leu-Asp(O*t*Bu)-Leu-ol (1.00 g, 1.87 mmol, 1.0 equiv) in 50 mL of MeOH and 10% Pd/C (100 mg, 10 wt%) were reacted according to GP2 to afford H_2N-Leu-Asp(O*t*Bu)-Leu-ol **83** (716 mg, 1.78 mmol, 95%) as a colorless solid.

R_f= 0.07 (CH_2Cl_2 / MeOH = 20:1).

HPLC (205 nm, VWD): t_R = 1.87 min.

^{1}H-NMR (500 MHz, $CDCl_3$): δ = 8.17 (d, J = 8.3 Hz, 1H), 6.60 (d, J = 8.2 Hz, 1H), 4.71 – 4.65 (m, 1H), 4.00 – 3.92 (m, 1H), 3.64 (dd, J = 11.2, 3.6 Hz, 1H), 3.50 – 3.45 (m, 1H), 3.39 (dd, J = 9.8, 4.0 Hz, 1H), 2.76 (dd, J = 16.6, 5.0 Hz, 1H), 2.67 (dd, J = 16.6, 7.2 Hz, 1H), 1.77 – 1.55 (m, 4H), 1.43 (s, 9H), 1.39 – 1.30 (m, 2H), 0.97 – 0.87 (m, 12H).

^{13}C-NMR (126 MHz, $CDCl_3$): δ = 176.3, 171.2, 81.9, 65.8, 53.6, 50.6, 49.6, 44.2, 40.1, 37.3, 28.2, 25.0, 23.5, 23.2, 22.3, 21.5.

(*S*)-*N*-((*S*)-1-Hydroxy-4-methylpentan-2-yl)-4-methyl-2-((*S*)-4-methyl-2-(2-(4-oxoquinazolin-3(4*H*)-yl)acetamido)pentanamido)pentanamide (84)

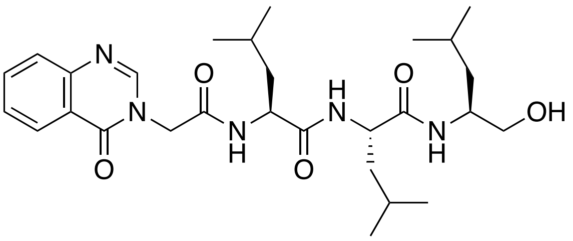

2-(4-Oxoquinazolin-3(4*H*)-yl)acetic acid (94 mg, 0.460 mmol, 1.0 equiv), H_2N-Leu-Leu-Leu-ol (158 mg, 0.460 mmol, 1.0 equiv), HATU (192 mg, 0.506 mmol, 1.1 equiv), and DIPEA (227 µL, 172 mg, 1.33 mmol, 2.9 equiv) in 6 mL of DMF were reacted according to GP9. The crude product was purified by column chromatography of the residue on silica gel (CH_2Cl_2 / MeOH = 20:1) to afford the title compound **84** (161 mg, 0.304 mmol, 66%) as a colorless solid.

R_f= 0.16 (CH_2Cl_2 / MeOH = 20:1).

HPLC (254 nm, VWD): t_R = 4.87 min.

^{1}H-NMR (500 MHz, DMSO-*d$_6$*): δ = 8.58 (d, *J* = 7.9 Hz, 1H), 8.28 (s, 1H), 8.12 (dd, *J* = 8.0, 1.5 Hz, 1H), 7.93 (d, *J* = 8.3 Hz, 1H), 7.84 (ddd, *J* = 8.5, 7.1, 1.6 Hz, 1H), 7.70 (dd, *J* = 8.2, 1.0 Hz, 1H), 7.56 (ddd, *J* = 8.2, 7.1, 1.2 Hz, 1H), 7.23 (d, *J* = 8.8 Hz, 1H), 4.70 (s, 2H), 4.50 (s, 1H), 4.34 – 4.28 (m, 1H), 4.25 – 4.19 (m, 1H), 3.77 – 3.68 (m, 1H), 3.28 – 3.24 (m, 1H), 3.17 – 3.10 (m, 1H), 1.68 – 1.61 (m, 1H), 1.61 – 1.55 (m, 1H), 1.52 – 1.42 (m, 5H), 1.28 – 1.21 (m, 1H), 1.18 – 1.11 (m, 1H), 0.91 – 0.87 (m, 6H), 0.86 – 0.83 (m, 6H), 0.76 – 0.74 (m, 3H), 0.73 – 0.71 (m, 3H).

^{13}C-NMR (126 MHz, DMSO-*d$_6$*): δ = 171.3, 171.1, 166.7, 160.3, 148.5, 148.1, 134.4, 127.2, 127.0, 126.0, 121.4, 63.8, 51.4, 48.5, 48.2, 40.9, 40.5, 24.2, 24.1, 24.0, 23.3, 23.0, 22.9, 21.8, 21.6.

***N*-((*S*)-1-(((*S*)-1-(((*S*)-1-Hydroxy-4-methylpentan-2-yl)amino)-4-methyl-1-oxopentan-2-yl)amino)-4-methyl-1-oxopentan-2-yl)pyrazine-2-carboxamide (85)**

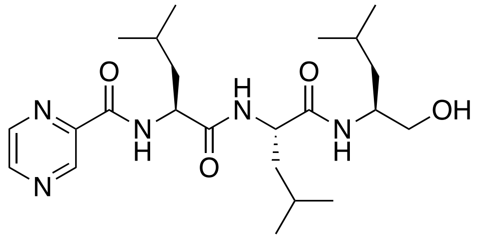

Pyrazine-2-carboxylic acid (57 mg, 0.460 mmol, 1.0 equiv), H$_2$N-Leu-Leu-Leu-ol (158 mg, 0.460 mmol, 1.0 equiv), HATU (192 mg, 0.506 mmol, 1.1 equiv), and DIPEA (227 µL, 172 mg, 1.33 mmol, 2.9 equiv) in 6 mL of DMF were reacted according to GP9. The crude product was purified by column chromatography of the residue on silica gel (CH_2Cl_2 / MeOH = 50:1) to afford the title compound **85** (147 mg, 0.324 mmol, 71%) as a colorless solid.

R*$_f$* = 0.24 (CH_2Cl_2 / MeOH = 20:1).

HPLC (254 nm, VWD): t_R = 4.46 min.

^{1}H-NMR (500 MHz, $CDCl_3$): δ = 9.38 (d, *J* = 1.3 Hz, 1H), 8.78 (d, *J* = 2.3 Hz, 1H), 8.56 – 8.53 (m, 1H), 8.18 (d, *J* = 7.9 Hz, 1H), 6.85 (d, *J* = 7.8 Hz, 1H), 6.47 (d, *J* = 8.2 Hz, 1H), 4.69 – 4.62 (m, 1H), 4.43 – 4.37 (m, 1H), 4.05 – 3.99 (m, 1H), 3.65 (dd, *J* = 11.1, 3.8 Hz, 1H), 3.56 (dd, *J* = 11.1, 5.6 Hz, 1H), 1.81 – 1.68 (m, 4H), 1.64 – 1.50 (m, 3H), 1.45 – 1.34 (m, 2H), 1.27 – 1.24 (m, 1H), 0.98 – 0.93 (m, 6H), 0.92 – 0.89 (m, 6H), 0.88 – 0.86 (m, 3H), 0.85 (d, *J* = 6.3 Hz, 3H).

^{13}C-NMR (126 MHz, $CDCl_3$): δ = 172.13, 171.91, 163.58, 147.87, 144.61, 143.93, 142.90, 65.87, 52.56, 52.41, 50.38, 41.21, 40.75, 40.14, 25.08, 25.02, 23.22, 23.05, 22.94, 22.29, 22.19.

2,5-Dichloro-*N*-((*S*)-1-(((*S*)-1-(((*S*)-1-hydroxy-4-methylpentan-2-yl)amino)-4-methyl-1-oxopentan-2-yl)amino)-4-methyl-1-oxopentan-2-yl)benzamide (86)

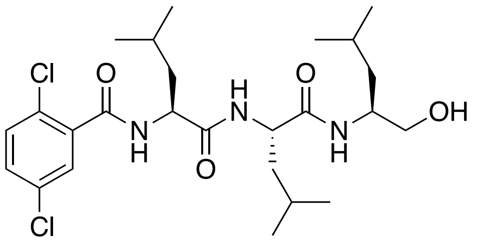

2,5-Dichlorobenzoic acid (414 mg, 2.17 mmol, 1.0 equiv), H_2N-Leu-Leu-Leu-ol (744 mg, 2.17 mmol, 1.0 equiv), EDC x HCl (416 mg, 2.17 mmol, 1.0 equiv), HOBt x H_2O (399 mg, 2.60 mmol, 1.2 equiv) and NEt_3 (451 µL, 329 mg, 3.26 mmol, 1.5 equiv) in 30 mL of CH_2Cl_2 were reacted according to GP10 to afford the title compound **86** (888 mg, 1.72 mmol, 79%) as a colorless solid.

R*f* = 0.24 (cyclohexane / AcOEt = 1:2).

HPLC (254 nm, VWD): t_R = 6.35 min.

^{1}H-NMR (500 MHz, $CDCl_3$): δ = 7.49 (t, J = 1.5 Hz, 1H), 7.30 (d, J = 1.4 Hz, 2H), 7.08 (d, J = 7.8 Hz, 2H), 6.59 (d, J = 8.3 Hz, 1H), 4.76 – 4.69 (m, 1H), 4.54 – 4.45 (m, 1H), 4.01 – 3.93 (m, 1H), 3.66 – 3.59 (m, 1H), 3.55 – 3.49 (m, 1H), 2.84 (s, 1H), 1.78 – 1.54 (m, 6H), 1.42 – 1.24 (m, 3H), 0.99 – 0.85 (m, 18H).

^{13}C-NMR (126 MHz, $CDCl_3$): δ = 172.1, 171.7, 165.6, 135.9, 133.4, 131.6, 131.5, 130.0, 129.2, 66.0, 52.9, 52.5, 50.3, 41.2, 41.0, 40.0, 25.1, 25.0, 23.2, 23.0, 22.4, 22.3.

***tert*-Butyl (*S*)-3-((*S*)-2-(2,5-dichlorobenzamido)-4-methylpentanamido)-4-(((*S*)-1-hydroxy-4-methylpentan-2-yl)amino)-4-oxobutanoate (87)**

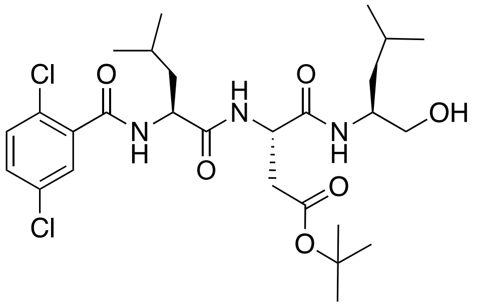

2,5-Dichlorobenzoic acid (327 mg, 1.71 mmol, 1.0 equiv), H_2N-Leu-Asp(O*t*Bu)-Leu-ol (685 mg, 1.71 mmol, 1.0 equiv), EDC x HCl (328 mg, 1.71 mmol, 1.0 equiv), HOBt x H_2O (314 mg, 2.05 mmol, 1.2 equiv) and NEt_3 (356 µL, 260 mg, 2.57 mmol, 1.5 equiv) in 30 mL of CH_2Cl_2 were reacted according to GP10 to afford the title compound **87** (721 mg, 1.25 mmol, 73%) as a colorless solid.

R*f* = 0.17 (cyclohexane / AcOEt = 1:2).

HPLC (254 nm, VWD): t_R = 6.76 min.

^{1}H-NMR (500 MHz, $CDCl_3$): δ= 7.74 – 7.72 (m, 1H), 7.67 (d, J = 8.2 Hz, 1H), 7.36 – 7.35 (m, 2H), 6.78 – 6.72 (m, 2H), 4.70 – 4.63 (m, 1H), 4.57 – 4.50 (m, 1H), 4.05 – 3.98 (m, 1H), 3.67 – 3.62 (m, 1H), 3.53 – 3.47 (m, 1H), 2.99 (dd, J = 17.0, 4.1 Hz, 1H), 2.82 (s, 1H), 2.60 (dd, J = 17.0, 6.3 Hz, 1H), 1.79 – 1.77 (m, 4H), 1.70 – 1.65 (m, 1H), 1.61 – 1.54 (m, 1H), 1.42 (s, 9H), 1.02 – 0.97 (m, 6H), 0.89 – 0.83 (m, 6H).

^{13}C-NMR (126 MHz, $CDCl_3$): δ= 171.9, 171.5, 170.4, 166.4, 135.6, 133.6, 131.9, 131.5, 130.4, 129.1, 82.4, 65.9, 53.9, 50.7, 50.2, 40.8, 39.9, 36.4, 28.1, 25.2, 24.8, 23.2, 22.1, 21.7.

e. Synthesis of intermediates for BODIPY labeled *α*-ketoamides (30 – 32)

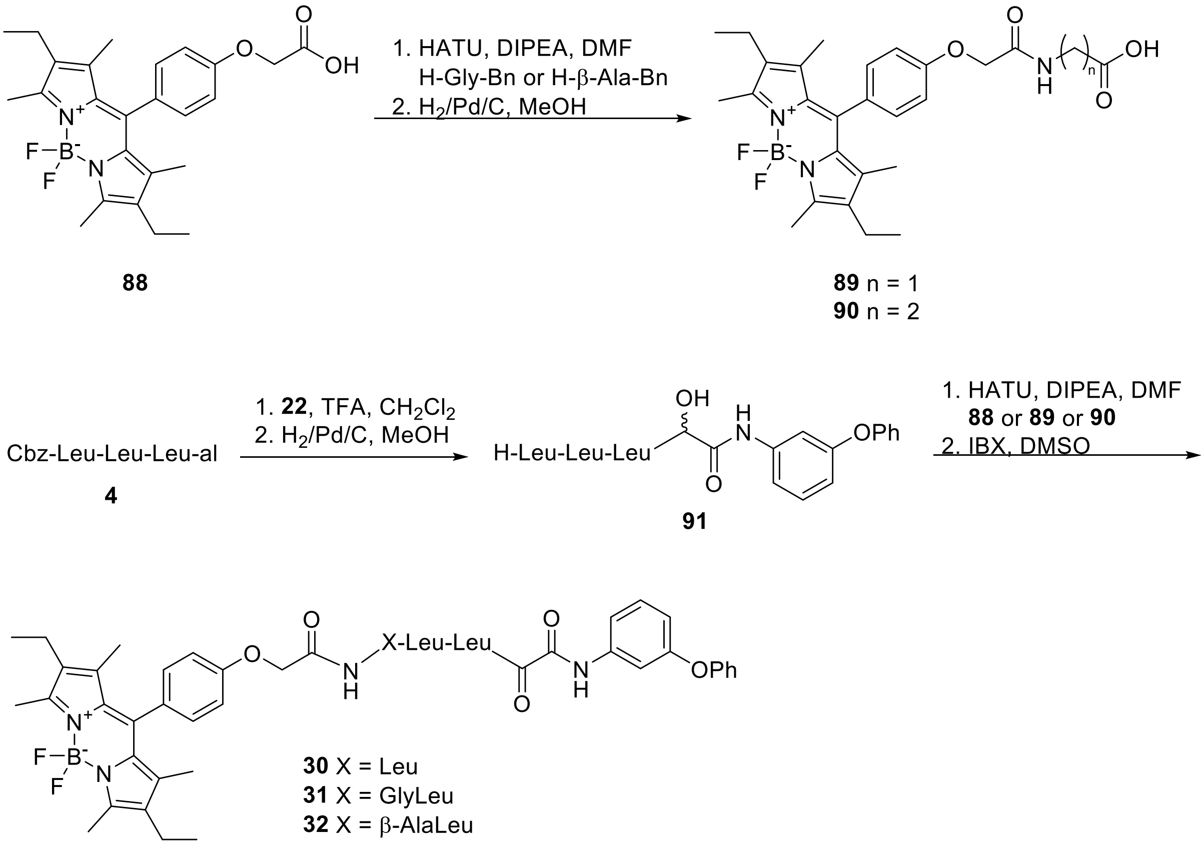

Scheme S5: Synthesis of BODIPY labeled α-ketoamides.

2-(4-(2,8-diethyl-5,5-difluoro-1,3,7,9-tetramethyl-5*H*-4λ^4,5λ^4-dipyrrolo[1,2-*c*:2',1'-*f*][1,3,2]diazaborinin-10-yl)phenoxy)acetic acid **88** was synthesized as described before.[3]

Benzyl(2-(4-(2,8-diethyl-5,5-difluoro-1,3,7,9-tetramethyl-5*H*-4λ^4,5λ^4-dipyrrolo[1,2-*c*:2',1'-*f*][1,3,2]diaza-borinin-10-yl)phenoxy)acetyl)glycinate

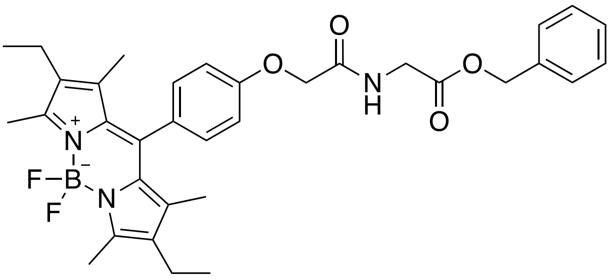

Compound **88** (300 mg, 0.660 mmol, 1.0 equiv), glycine benzyl ester *p*-toluenesulfonate (445 mg, 1.32 mmol, 2.0 equiv), HATU (277 mg, 0.729 mmol, 1.1 equiv), and DIPEA (324 µL, 247 mg, 1.91 mmol, 2.9 equiv) in 50 mL of DMF were reacted according to GP9 to afford the title compound (368 mg, 0.655 mmol, 97%) as a purple solid.

R*f* = 0.88 (cyclohexane / AcOEt = 1:2).

HPLC (254 nm, VWD): t_R = 9.00 min.

^{1}H-NMR (500 MHz, $CDCl_3$): *δ* = 7.33 – 7.26 (m, 5H), 7.15 (d, *J* = 8.7 Hz, 2H), 6.96 (d, *J* = 8.7 Hz, 2H), 5.14 (s, 2H), 4.52 (s, 2H), 4.13 (s, 2H), 2.65 (s, 1H), 2.46 (s, 6H), 2.23 (q, *J* = 7.6 Hz, 4H), 1.24 (s, 6H), 0.91 (t, *J* = 7.5 Hz, 6H).

^{13}C-NMR (126 MHz, $CDCl_3$): *δ* = 169.4, 168.1, 157.6, 153.9, 139.6, 138.3, 135.1, 132.9, 131.1, 130.1, 129.7, 128.8, 128.8, 128.5, 115.4, 67.5, 67.3, 41.0, 38.7, 17.2, 14.7, 12.6, 11.9.

(2-(4-(2,8-Diethyl-5,5-difluoro-1,3,7,9-tetramethyl-5*H*-4λ^4,5λ^4-dipyrrolo[1,2-*c*:2',1'-*f*][1,3,2]diazaborinin-10-yl)phenoxy)acetyl)glycine (89)

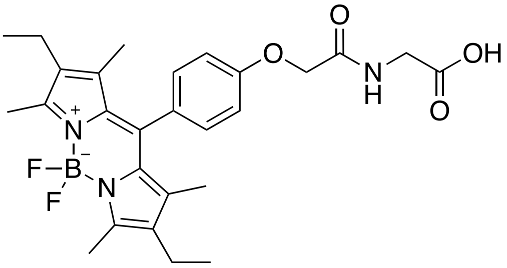

Benzyl(2-(4-(2,8-diethyl-5,5-difluoro-1,3,7,9-tetramethyl-5*H*-4λ^4,5λ^4-dipyrrolo[1,2-*c*:2',1'-*f*][1,3,2]diazaborinin-10-yl)phenoxy)acetyl)glycinate (386 mg, 0.655 mmol, 1.0 equiv) in 25 mL of MeOH and 10% Pd/C (39 mg, 10 wt%) were reacted according to GP2 to afford the title compound **89** (290 mg, 0.567 mmol, 85%) as a purple solid.

R*f* = 0.53 (CH_2Cl_2 / MeOH = 10:1).

HPLC (254 nm, VWD): t_R = 7.32 min.

^{1}H-NMR (500 MHz, $CDCl_3$): *δ* = 8.34 (t, *J* = 5.9 Hz, 1H), 7.26 (d, *J* = 8.1 Hz, 2H), 7.16 (d, *J* = 8.2 Hz, 2H), 4.61 (s, 2H), 3.81 (d, *J* = 5.7 Hz, 2H), 2.43 (s, 6H), 2.29 (q, *J* = 7.5 Hz, 4H), 1.31 (s, 6H), 0.94 (t, *J* = 7.5 Hz, 6H).

^{13}C-NMR (126 MHz, $CDCl_3$): δ = 171.0, 167.5, 158.1, 153.0, 140.5, 138.1, 132.5, 130.3, 129.3, 127.4, 115.5, 66.9, 40.8, 16.4, 14.5, 12.2, 11.5.

^{19}F NMR (471 MHz, DMSO-d_6): δ = -143.00 (dd, J = 66.3, 29.4 Hz).

^{11}B NMR (160 MHz, DMSO-d_6): δ = (160 MHz, DMSO-d6) δ 3.69 (t, J = 33.5 Hz).

Benzyl-3-(2-(4-(2,8-diethyl-5,5-difluoro-1,3,7,9-tetramethyl-5*H*-4λ^4,5λ^4-dipyrrolo[1,2-*c*:2',1'-*f*][1,3,2]diaza-borinin-10-yl)phenoxy)acetamido)propanoate

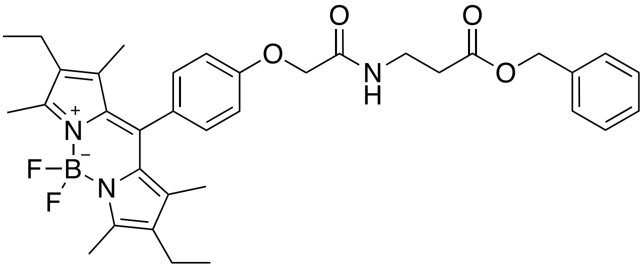

Compound **88** (300 mg, 0.660 mmol, 1.0 equiv), β-alanine benzyl ester *p*-toluenesulfonate (464 mg, 1.32 mmol, 2.0 equiv), HATU (277 mg, 0.729 mmol, 1.1 equiv), and DIPEA (324 µL, 247 mg, 1.91 mmol, 2.9 equiv) in 50 mL of DMF were reacted according to GP9 to afford the title compound (284 mg, 0.461 mmol, 70%) as a purple solid.

R_f = 0.62 (cyclohexane / AcOEt = 1:2).

HPLC (254 nm, VWD): t_R = 8.98 min.

^{1}H-NMR (500 MHz, $CDCl_3$): δ = 7.31 – 7.37 (m, 5H), 7.21 (d, J = 8.7 Hz, 2H), 7.00 (d, J = 8.7 Hz, 2H), 5.29 (s, 1H), 5.16 (s, 2H), 4.52 (s, 2H), 3.67 (q, J = 6.0 Hz, 2H), 2.66 (t, J = 6.0 Hz, 2H), 2.52 (s, 6H), 2.29 (q, J = 7.6 Hz, 4H), 1.30 (s, 6H), 0.98 (t, J = 7.5 Hz, 6H).

^{13}C-NMR (126 MHz, $CDCl_3$): δ = 172.0, 167.6, 157.4, 153.8, 139.4, 138.1, 135.5, 132.7, 131.0, 129.9, 129.4, 128.6, 128.4, 128.2, 115.2, 67.2, 66.6, 38.6, 34.4, 34.0, 17.0, 14.6, 12.3, 11.8.

3-(2-(4-(2,8-Diethyl-5,5-difluoro-1,3,7,9-tetramethyl-5*H*-4λ[4],5λ[4]-dipyrrolo[1,2-*c*:2',1'-*f*][1,3,2]diaza-borinin10-yl)phenoxy)acetamido)propanoic acid (90)

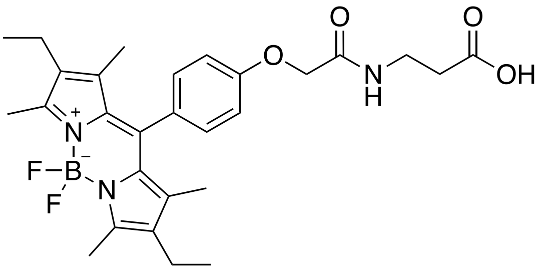

Benzyl 3-(2-(4-(2,8-diethyl-5,5-difluoro-1,3,7,9-tetramethyl-5*H*-4λ[4],5λ[4]-dipyrrolo[1,2-*c*:2',1'-*f*][1,3,2]diazaborinin-10-yl)phenoxy)acetamido)propanoate (284 mg, 0.461 mmol, 1.0 equiv) in 50 mL of MeOH and 10% Pd/C (29 mg, 10 wt%) were reacted according to GP2 to afford the title compound **90** (164 mg, 0.312 mmol, 47%) as a purple solid.

$\mathbf{R}_f$ = 0.86 (CH_2Cl_2 / MeOH = 40:1).

HPLC (254 nm, VWD): t_R = 7.15 min.
^{1}H-NMR (500 MHz, $CDCl_3$): δ = 8.17 (t, J = 5.8 Hz, 1H), 7.26 (d, J = 8.1 Hz, 2H), 7.13 (d, J = 8.1 Hz, 2H), 4.54 (s, 2H), 3.39 – 3.33 (m, 4H), 2.43 (s, 6H), 2.29 (q, J = 7.5 Hz, 4H), 1.30 (s, 6H), 0.94 (t, J = 7.5 Hz, 6H).

^{13}C-NMR (126 MHz, $CDCl_3$): δ = 172.8, 167.2, 158.1, 152.9, 140.5, 138.1, 132.5, 130.3, 129.3, 127.4, 115.4, 67.0, 34.5, 33.7, 16.4, 14.5, 12.2, 11.5.

^{19}F NMR (471 MHz, DMSO-d_6): δ = -143.00 (dd, J = 66.1, 30.0 Hz).

^{11}B NMR (160 MHz, DMSO-d_6): δ = 3.68 (t, J = 33.6 Hz).

(3*S*)-3-((*S*)-2-((*S*)-2-Amino-4-methylpentanamido)-4-methylpentanamido)-2-hydroxy-5-methyl-*N*-(3-phenoxyphenyl)hexanamide (91)

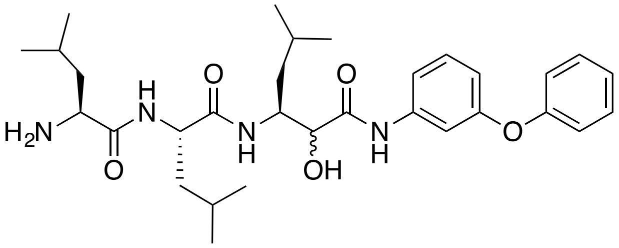

Cbz-Leu-Leu-Leu-al (957 mg, 2.01 mmol, 1.0 equiv) and 1-isocyano-3-phenoxybenzene (586 mg, 3.02 mmol, 1.5 equiv) were dissolved in 25 mL of CH_2Cl_2 and cooled to 0°C under an atmosphere of argon. A mixture of trifluoroacetic acid (310 µL, 458 mg, 4.02 mmol, 2.0 equiv) in 1 mL of CH_2Cl_2 was added dropwise and the mixture was stirred for 2 h at 0°C and 24 h at room temperature. Completion of the reaction was monitored by HPLC and TLC. CH_2Cl_2 (100 mL) were added and the organic layer was washed with 0.1 N aq. HCl (3x 80 mL), sat. aq. $NaHCO_3$ (3x 80 mL) and sat. aq. NaCl (1x 80 mL). The organic layer was dried over Na_2SO_4 and the solvent was evaporated under reduced pressure to afford the crude hydroxyamide. 200 mg of the obtained solid were dissolved in 25 mL of MeOH and added to 10% Pd/C (20 mg, 10 wt%). The mixture was stirred 15 min. under an atmosphere of hydrogen. After completion of the reaction, the mixture was filtered through a pad of celite and the solvent was removed under reduced pressure. The crude amine was purified by column chromatography on silica (CH_2Cl_2 / MeOH = 20:1) to afford the free amine (87 mg, 0.159 mmol) as a colorless solid and as a mixture of two diastereomers.

R*f*= 0.26 (CH_2Cl_2 / MeOH = 20:1).

HPLC (254 nm, VWD): t_R = 5.47 min (diastereomer 1), 5.91 min (diastereomer 2).

ESI-MS: *m/z* = 555.37 $[M+H]^+$.

f. Compound purity as determined by HPLC

Compound	BSc	t_r / min	purity / %	λ / nm
8	5321	9.062	98.0256	254
9	5322	9.906	97.8340	205
10	5323	9.225	97.8486	205
18	5324	9.266	99.1150	254
19	5325	9.118	99.0789	254
20	5326	9.052	97.5287	254
16	5327	8.267	98.5202	254
17	5328	9.472	97.3843	254
15	5356	9.006	98.4421	254
14	5357	8.977	97.8081	254
13	5358	9.090	98.0169	254
11	5359	9.127	97.8065	254
12	5360	9.093	96.1759	254
22	5372	4.967	97.8043	254
24	5373	7.198	97.8479	205
23	5374	4.619	98.9923	254
26	5375	8.430	97.5408	254
27	5376	9.273	99.0460	254
29	5377	9.492	99.2761	254
28	5378	8.636	97.4556	254
21	5379	9.337	97.8595	254
25	5380	7.529	96.6261	205
30	5390	10.589	99.2857	360
31	5391	10.204	99.4795	360
32	5392	10.159	98.5419	360

2. Biochemistry

a. Summary of proteasome and cytotoxicity assays

Table S1: Inhibition of catalytic subunits β5c, β2c, β1c and β5i in isolated human 20S proteasome by compounds used in this study. n.a. = not available.

ID	Inhibition cCP / %				Inhibition iCP / %	
	β5		β2	β1	β5	
	c = 100 nM	c = 1 µM			c = 100 nM	c = 1 µM
**1**	89	98	5	80	85	98
**3**	86	96	37	-18	43	91
**4**	43	82	21	27	6	72
**5**	54	90	4	16	11	72
**7**	64	92	9	-26	33	74
**8**	12	32	-4	-7	8	7
**9**	56	81	9	0	23	31
**10**	25	63	2	-3	5	23
**18**	28	59	2	-12	18	7
**19**	53	84	9	-15	43	31
**20**	41	38	-7	-24	40	22
**16**	57	91	-8	12	48	76
**17**	43	88	-10	11	36	69
**15**	26	75	9	-2	19	48
**14**	41	78	5	-10	29	30
**13**	49	69	4	-4	28	15
**11**	46	59	10	-10	12	10
**12**	20	53	4	-33	9	16
**21**	33	52	11	-4	4	17
**23**	30	75	49	19	4	64
**24**	76	94	26	13	60	89
**22**	6	43	-10	28	11	28
**25**	64	90	10	9	38	75
**26**	25	51	17	-38	4	24
**27**	83	91	6	-48	58	68
**29**	26	53	-4	-19	8	-1
**28**	26	81	6	-10	35	52

b. Dose-Response Curves

a. IC_{50} determination of purified proteasomes β5 subunit

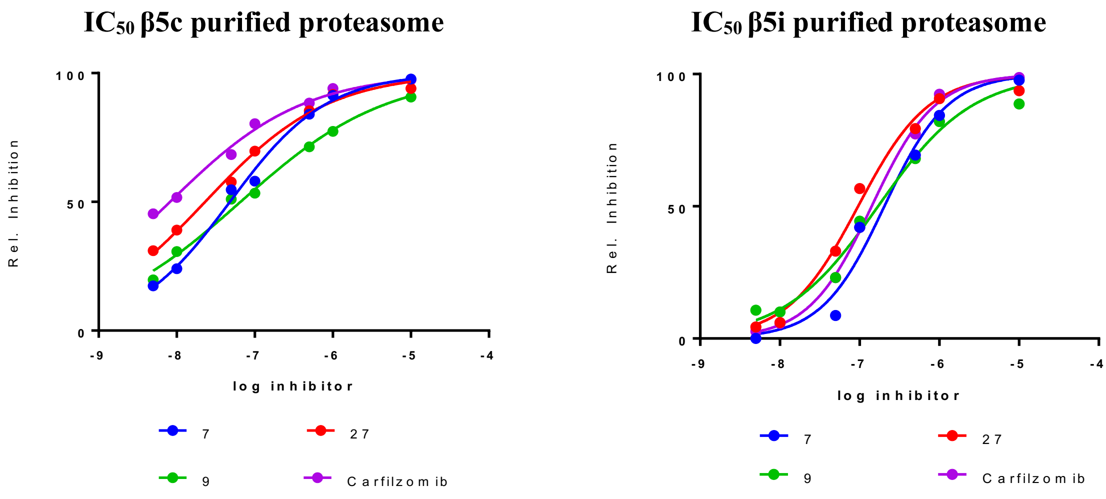

Figure S1: Dose-response curves for the inhibition of β5c and β5i by key compounds used in this study.

IC$_{50}$ determination of cytotoxicity in MV4-11 cells

Replicate 1

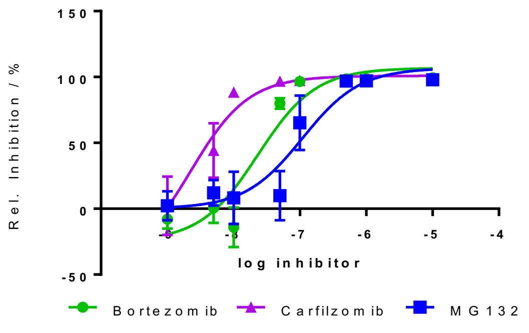

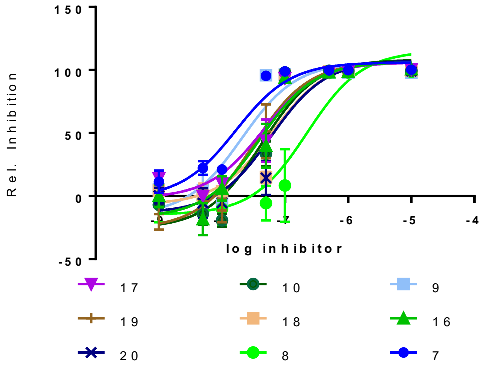

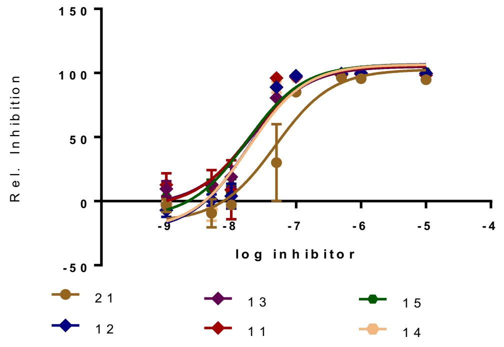

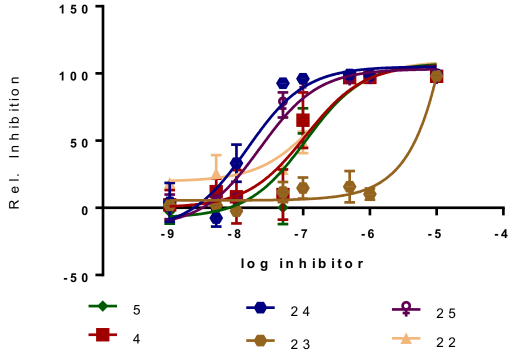

Replicate 2

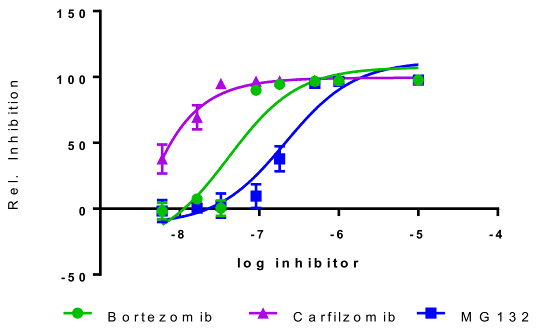

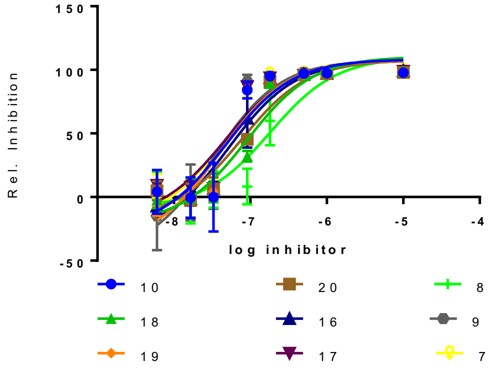

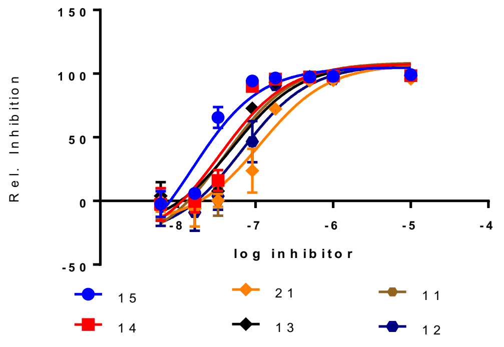

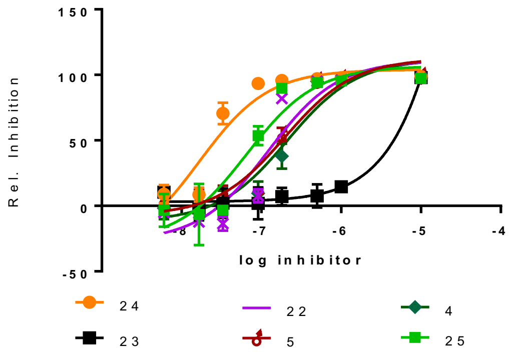

(Table continues on next page)

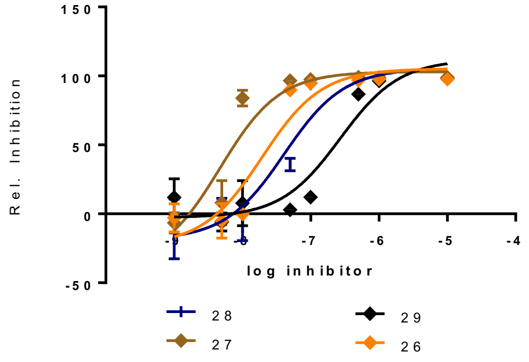

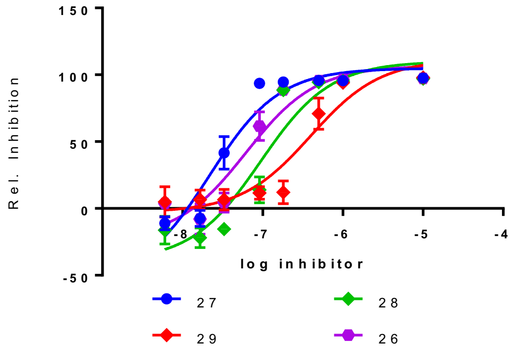

Figure S2: Dose-response curves for the determination of cytotoxic activity in MV4-11 cells by compounds used in this study.

IC$_{50}$ determination of cytotoxicity in THP1 cells

Replicate 1

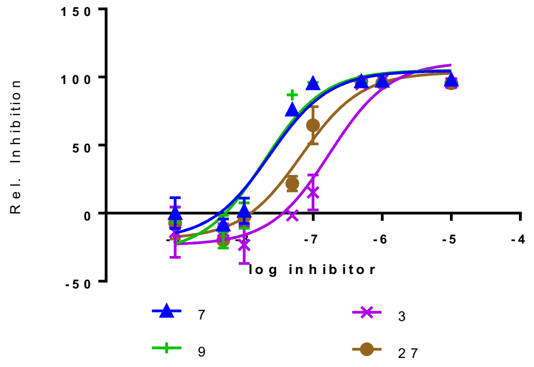

Replicate 2

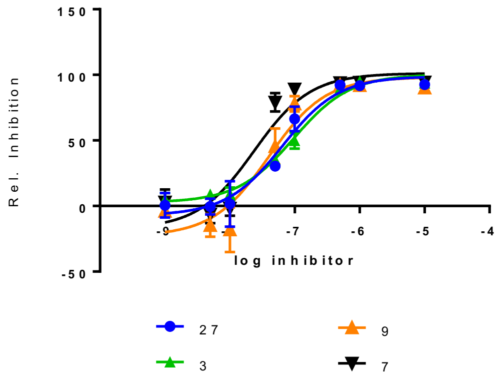

Figure S3: Dose-response curves for the determination of cytotoxic activity in THP-1 cells by key compounds used in this study.

IC$_{50}$ determination of cytotoxicity in Jurkat cells

Replicate 1

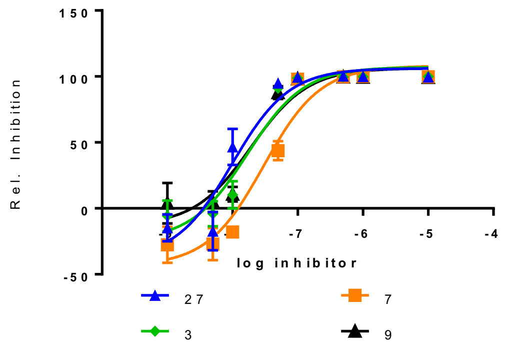

Replicate 2

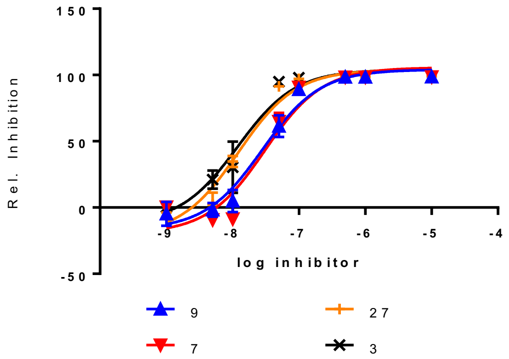

Figure S4: Dose-response curves for the determination of cytotoxic activity in Jurkat cells by key compounds used in this study.

Assay validation: Cellular conversion of substrate 33

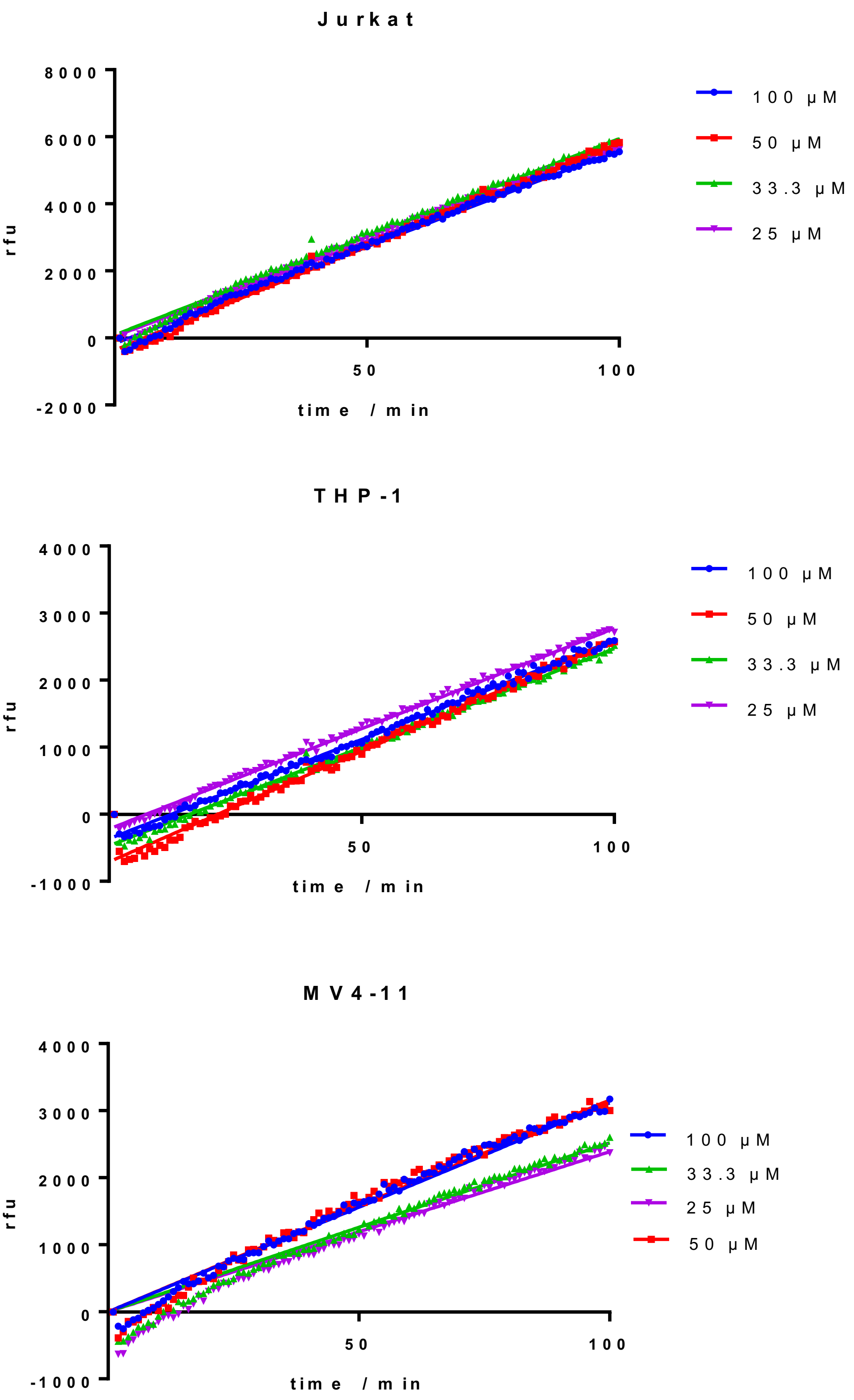

Figure S5: Cellular conversion of Z-LD(OtBu)A-AMC using 25 µM tp 100 µM substrate, 60 min pre-incubation of substrate and cells and 100 min fluorescence detection.

Inhibition of cellular Z-LD(OtBu)A-AMC Substrate conversion

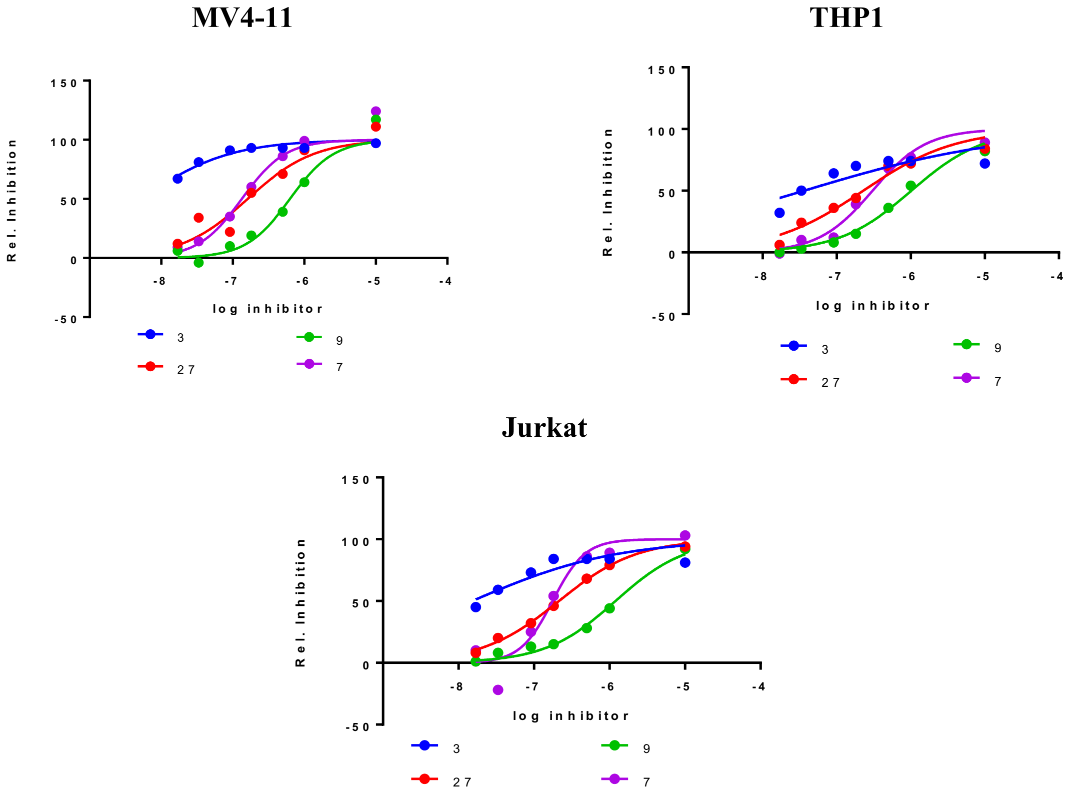

Figure S6: Dose-response curves for the inhibition of Z-L-D(OtBu)A-AMC proteolysis by key compounds used in this study (0 h pre-incubation).

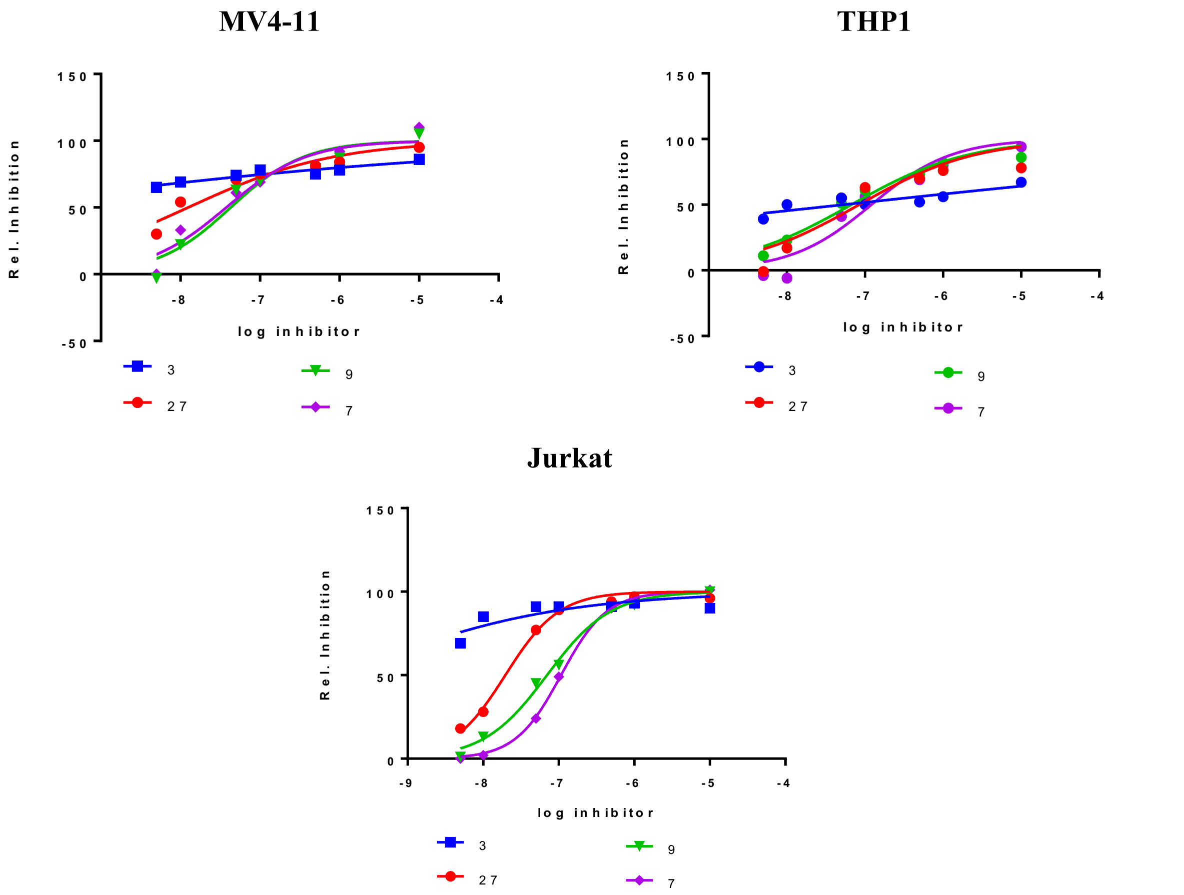

Figure S7: Dose-response curves for the inhibition of Z-L-D(OtBu)A-AMC proteolysis by key compounds used in this study (1 h pre-incubation).

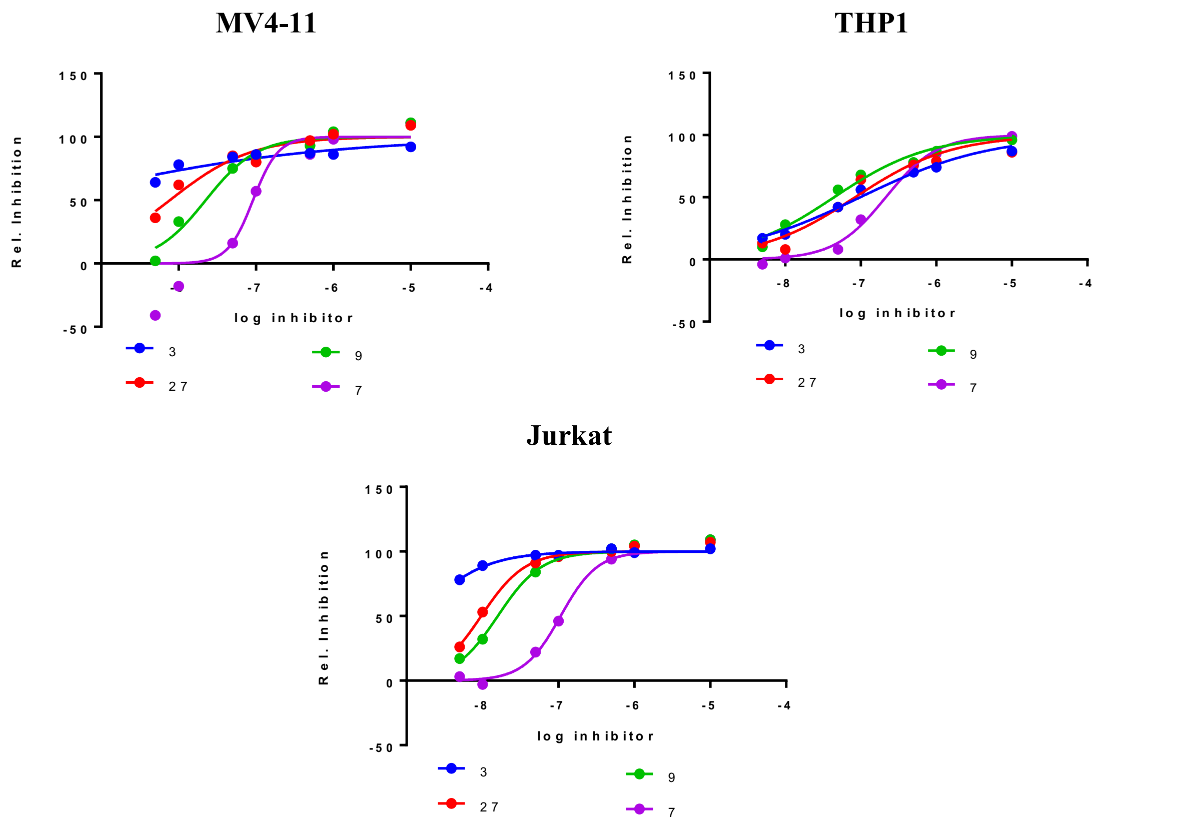

Figure S8: Dose-response curves for the inhibition of Z-L-D(OtBu)A-AMC proteolysis by key compounds used in this study (13 h pre-incubation).

Cytotoxicity of 27 in MV4-11 Cells at different time points

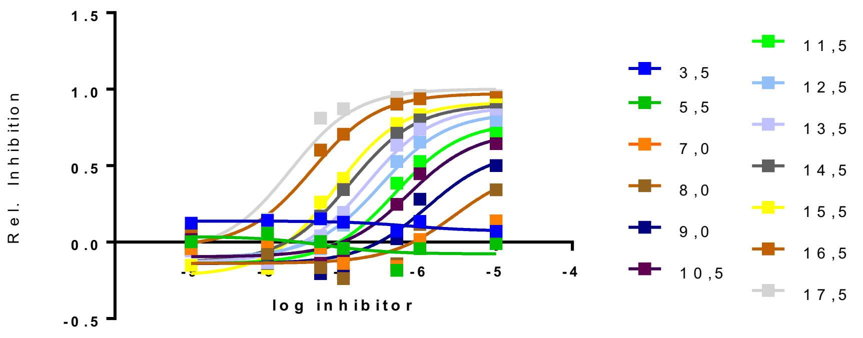

Figure S9: Dose-response curves for the continuous determination of cytotoxic activity in MV4-11 cells by Compound **27** over 17.5 h.

Cytotoxicity of 3 in MV4-11 Cells at different time points

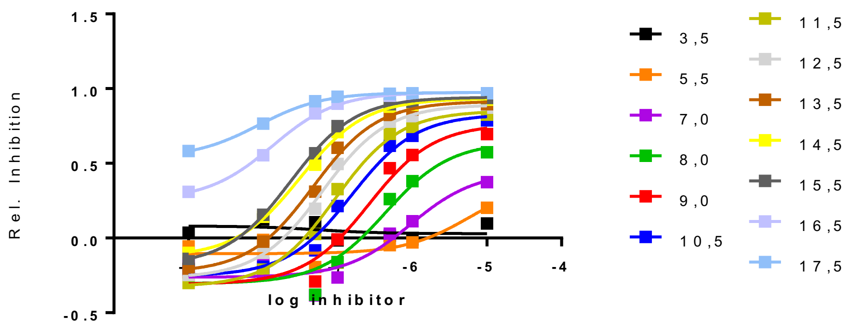

Figure S10: Dose-response curves for the continuous determination of cytotoxic activity in MV4-11 cells by compound **3** over 17.5 h.

Comparison of cytotoxicity in MV4-11 Cells at different time points

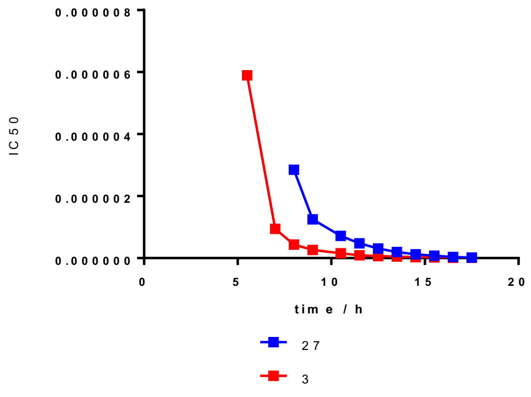

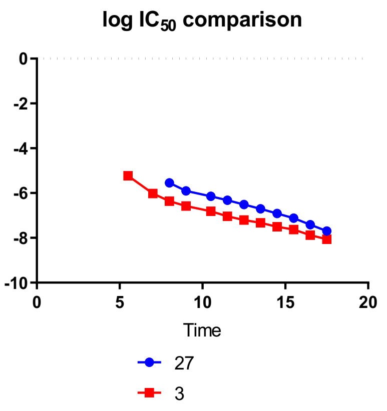

Figure S11: Comparison of time-dependent cytotoxic activity in MV4-11 cells by compounds **27** and **3** over 17.5 h.

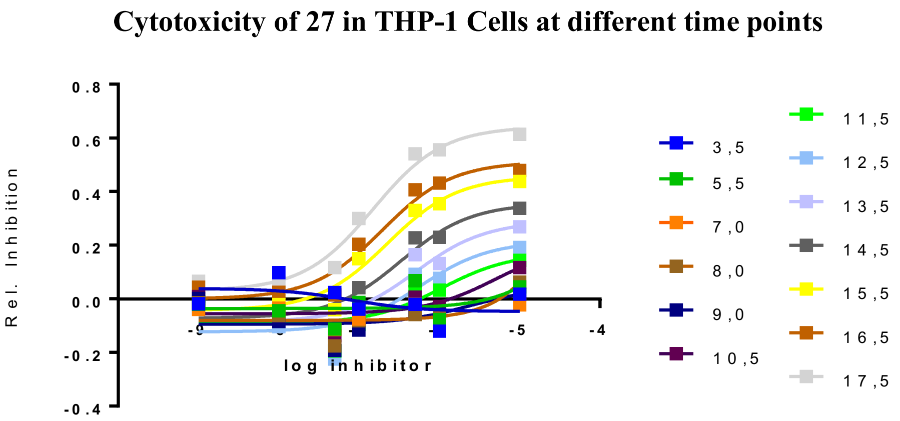

Figure S12: Dose-response curves for the continuous determination of cytotoxic activity in THP-1 cells by Compound **27** over 17.5 h.

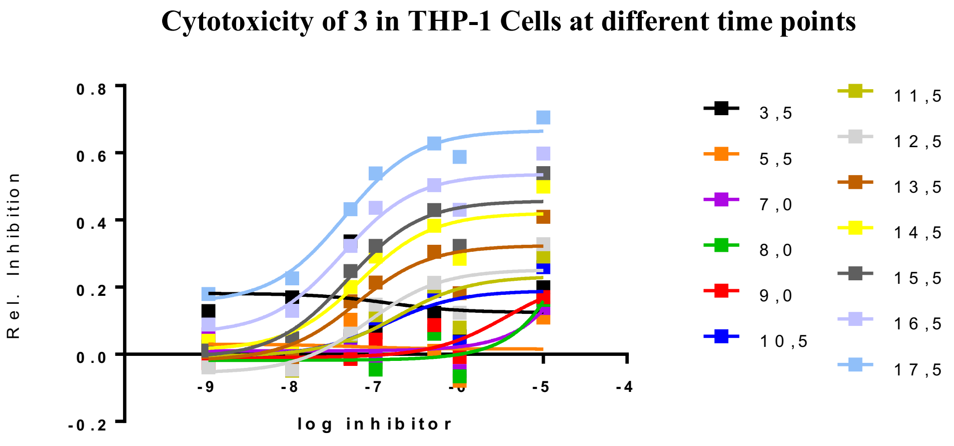

Figure S13: Dose-response curves for the continuous determination of cytotoxic activity in THP-1 cells by Compound **3** over 17.5 h.

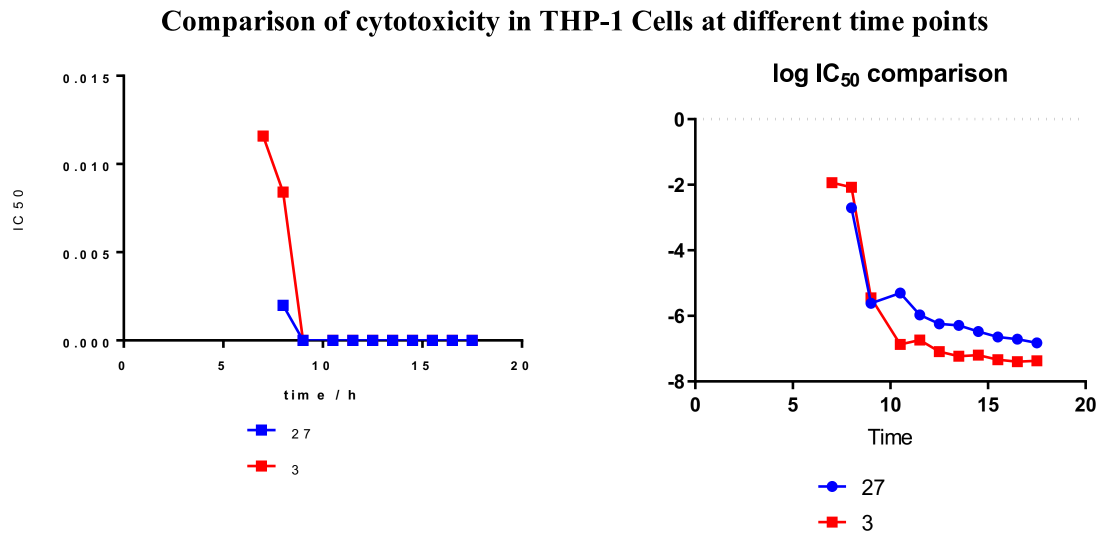

Figure S14: Comparison of time-dependent cytotoxic activity in THP-1 cells by compounds **27** and **3** over 17.5 h.

Cytotoxicity of 27 in Jurkat Cells at different time points

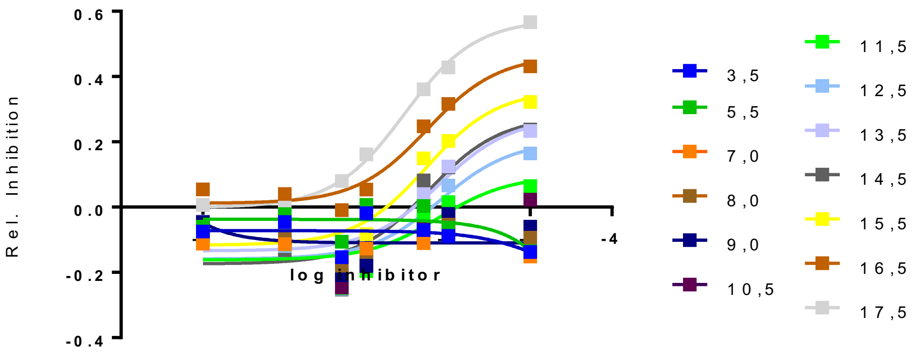

Figure S15: Dose-response curves for the continuous determination of cytotoxic activity in Jurkat cells by Compound **27** over 17.5 h.

Cytotoxicity of 3 in Jurkat Cells at different time points

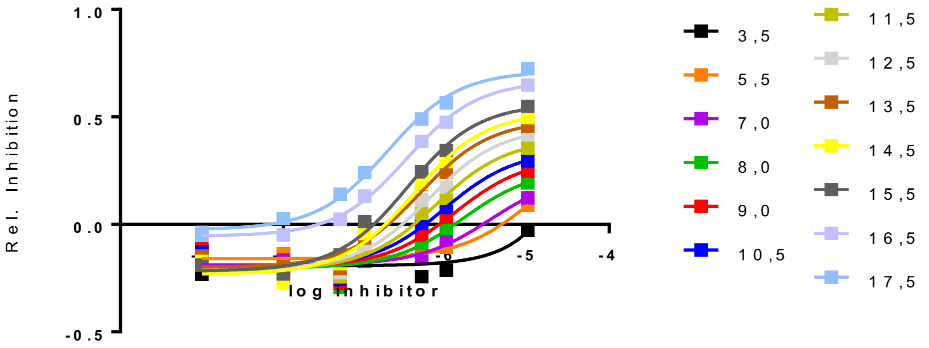

Figure S16: Dose-response curves for the continuous determination of cytotoxic activity in Jurkat cells by Compound **3** over 17.5 h.

Comparison of Cytotoxicity in Jurkat Cells at different time points

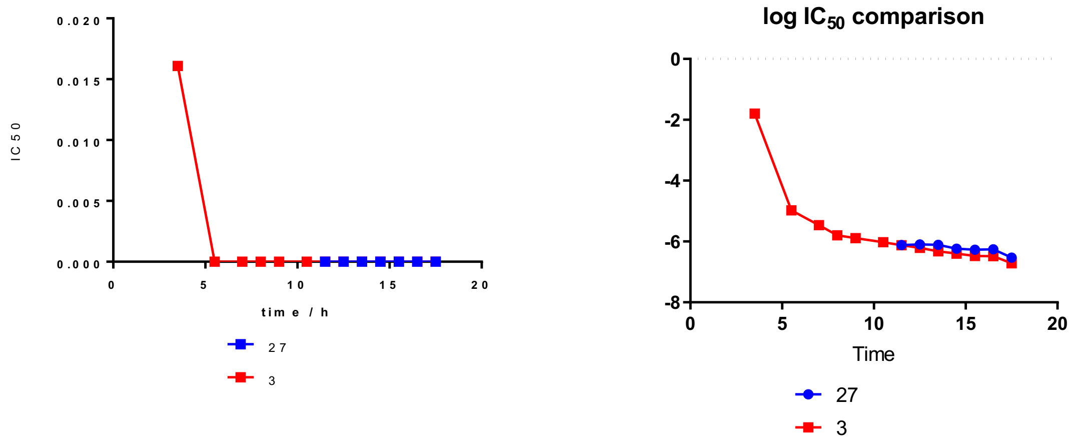

Figure S17: Comparison of time-dependent cytotoxic activity in Jurkat cells by compounds **27** and **3** over 17.5 h.

Summary of time-dependent cytotoxicity

Table S2: Comparison of time-dependent cytotoxic activity (IC50) and relative IC_{50} values in MV4-11, THP-1 and Jurkat cells by compounds **27** and **3** over 17.5 h.

	MV4-11			Jurkat			THP-1		
	IC_{50} / nM			IC_{50} / nM			IC_{50} / nM		
**Time / h**	**27**	**3**	**Fold-IC_{50}**	**2**	**3**	**Fold-IC_{50}**	**27**	**3**	**Fold-IC_{50}**
**3,5**					1,61E-02				
**5,5**		5,89E-06			1,06E-05				
**7**		9,44E-07			3,46E-06			1,16E-02	
**8**	2,85E-06	4,37E-07	6,5		1,61E-06		1,99E-03	8,41E-03	0,2
**9**	1,25E-06	2,63E-07	4,8		1,28E-06		2,43E-06	3,56E-06	0,7
**10,5**	7,15E-07	1,54E-07	4,7		9,54E-07		4,96E-06	1,34E-07	37,0
**11,5**	4,78E-07	9,25E-08	5,2	7,67E-07	7,51E-07	1,0	1,07E-06	1,82E-07	5,8
**12,5**	3,08E-07	6,21E-08	5,0	8,05E-07	6,15E-07	1,3	5,70E-07	8,19E-08	7,0
**13,5**	1,99E-07	4,67E-08	4,3	7,80E-07	4,73E-07	1,6	5,09E-07	5,91E-08	8,6
**14,5**	1,21E-07	3,15E-08	3,9	5,80E-07	4,01E-07	1,4	3,35E-07	6,34E-08	5,3
**15,5**	7,63E-08	2,34E-08	3,3	5,40E-07	3,33E-07	1,6	2,26E-07	4,61E-08	4,9
**16,5**	3,84E-08	1,33E-08	2,9	5,53E-07	3,28E-07	1,7	1,94E-07	4,01E-08	4,8
**17,5**	2,00E-08	8,67E-09	2,3	2,94E-07	1,93E-07	1,5	1,51E-07	4,24E-08	3,6

c. Danio rerio toxicity assay

Table S3: Toxicity in Danio rerio embryos (not decorionated) by key compounds used in this study.

Compound	Concentration [µM]	0 h		24 h		48 h		72 h		96 h	
		n	%	n	%	n	%	n	%	n	%
1	1	5	100%	5	100%	5	100%	5	100%	5	100%
	5	5	100%	5	100%	5	100%	5	100%	4	80%
	10	5	100%	5	100%	5	100%	5	100%	5	100%
	25	5	100%	5	100%	5	100%	5	100%	4	80%
3	1	5	100%	5	100%	5	100%	5	100%	5	100%
	5	5	100%	5	100%	5	100%	5	100%	5	100%
	10	5	100%	5	100%	5	100%	5	100%	5	100%
	25	5	100%	5	100%	5	100%	5	100%	5	100%
7	1	5	100%	5	100%	5	100%	5	100%	5	100%
	5	5	100%	5	100%	5	100%	5	100%	5	100%
	10	5	100%	5	100%	5	100%	5	100%	5	100%
	25	5	100%	4	80%	4	80%	4	80%	4	80%
9	1	5	100%	5	100%	5	100%	5	100%	5	100%
	5	5	100%	5	100%	5	100%	5	100%	5	100%
	10	5	100%	5	100%	5	100%	5	100%	5	100%
	25	5	100%	5	100%	5	100%	5	100%	5	100%
27	1	5	100%	5	100%	5	100%	5	100%	5	100%
	5	5	100%	5	100%	5	100%	5	100%	5	100%
	10	5	100%	5	100%	5	100%	5	100%	5	100%
	25	5	100%	5	100%	5	100%	5	100%	5	100%
Control	-	5	100%	5	100%	5	100%	5	100%	5	100%

d. Escape response assay

a. Setup and Image processing

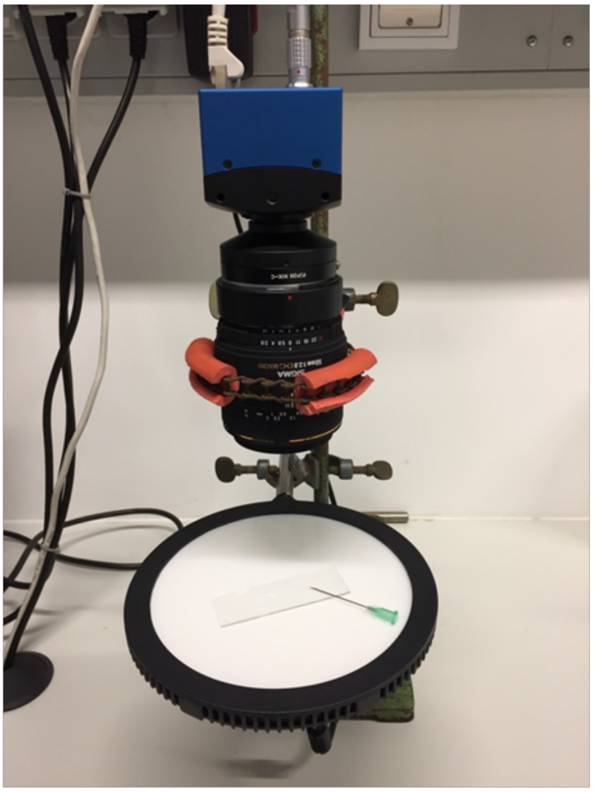

Figure S18: Imaging setup for motion tracking of Danio rerio embryos. High-Speed Digital Camera MotionBlitz EoSens mini1; KIPON Nikon C adapter; 20 mm extension ring; Sigma 50 mm F2.8 EX DG macro objective (55 mm); SL-300 LED Soft Light.

b. Summary –detailed statistics of kinematic parameters

Table S4: Overview of assayed compounds and concentrations, sample size, number of reflexes, stimuli and relative number of reflexes per stimuli.

Groups tested in Neurotoxicity Assay									
	c	time	Origin	Survived	Assayed	# stimuli	# reflexes	Relative reflexes / Stimuli	Mean
**Control**	-	-	4	4	3	19	9	0,474	0,47
	25 µM	24 h	4	4	3	100	8	0,080	
**1**	25 µM	48 h	4	3	3	150	3	0,020	0,03
	50 µM	24 h	4	4	3	150	0	0,000	
	25 µM	24 h	4	2	2	6	6	1,000	
**27**	50 µM	24 h	4	3	3	9	9	1,000	0,81
	25 µM	48 h	4	2	2	11	6	0,545	
								Fold-response	29

Table S5: Detailed statistics for analysis of kinematic parameters in the touch-evoked escape response assay.

		c	time	Mean	Difference between means	95% CI	p	Interpretation	R^2
C-Bend Amplitude	Control			129 ± 13,26, n=9	n.a.	n.a.	n.a.	n.a.	n.a.
	1	25 µM	24 h	131,6 ± 21,3, n=9	2,556 ± 25,09	-50,64 to 55,75	0,9201	n.s.	0,0006479
		25 µM	48 h	**6,889 ± 5,549, n=9**	**-122,1 ± 14,37**	**-152,6 to -91,64**	**<0,0001**	********	**0,8185**
		50 µM	24 h	**0 ± 0, n=9**	**-129 ± 13,26**	**-157,1 to -100,9**	**<0,0001**	********	**0,8554**
	27	25 µM	24 h	126,2 ± 14,05, n=6	-2,833 ± 19,93	-45,88 to 40,21	0,8891	n.s.	0,001553
		50 µM	24 h	121,7 ± 7,399, n=9	-7,333 ± 15,18	-39,52 to 24,86	0,6357	n.s.	0,01437
		25 µM	48 h	119,8 ± 6,848, n=6	-9,167 ± 17,34	-46,62 to 28,29	0,6059	n.s.	0,02105
C-Bend duration	Control			19,67 ± 1,374, n=9	n.a.	n.a.	n.a.	n.a.	n.a.
	1	25 µM	24 h	32,67 ± 6,489, n=9	13 ± 6,633	-1,062 to 27,06	0,0677	n.s.	0,1936
		25 µM	48 h	8,222 ± 6,654, n=9	-11,44 ± 6,794	-25,85 to 2,959	0,1115	n.s.	0,1506
		50 µM	24 h	**0 ± 0, n=9**	**-19,67 ± 1,374**	**-22,58 to -16,75**	**<0,0001**	********	**0,9275**
	27	25 µM	24 h	**14 ± 0,8165, n=6**	**-5,667 ± 1,826**	**-9,611 to -1,722**	**0,0084**	******	**0,4256**
		50 µM	24 h	19,17 ± 2,212, n=6	-0,5 ± 2,458	-5,811 to 4,811	0,842	n.s.	0,003172
		25 µM	48 h	**15,56 ± 0,5031, n=9**	**-4,111 ± 1,464**	**-7,214 to -1,009**	**0,0126**	*****	**0,3303**
Reflex Duration	Control			390,7 ± 50,72, n=9	n.a.	n.a.	n.a.	n.a.	n.a.
	1	25 µM	24 h	540,2 ± 136,6, n=9	149,6 ± 145,7	-159,3 to 458,4	0,32	n.s.	0,06178
		25 µM	48 h	289,3 ± 198,9, n=9	-101,3 ± 205,3	-536,6 to 333,9	0,6283	n.s.	0,015
		50 µM	24 h	**0 ± 0, n=9**	**-390,7 ± 50,72**	**-498,2 to -283,1**	**<0,0001**	********	**0,7876**
	27	25 µM	24 h	295,3 ± 28,53, n=6	-95,33 ± 66,93	-239,9 to 49,27	0,1779	n.s.	0,135
		50 µM	24 h	**799 ± 91,81, n=6**	**408,3 ± 96,75**	**199,3 to 617,4**	**0,001**	******	**0,5781**
		25 µM	48 h	403,6 ± 30,92, n=9	12,89 ± 59,4	-113 to 138,8	0,831	n.s.	0,002934
Number of total movements	Control				n.a.	n.a.	n.a.	n.a.	n.a.
	1	25 µM	24 h	11,56 ± 3,33, n=9	-2,556 ± 4,37	-11,82 to 6,709	0,5669	n.s.	0,02092
		25 µM	48 h	**3,222 ± 2,259, n=9**	**-10,89 ± 3,622**	**-18,57 to -3,211**	**0,0084**	******	**0,361**
		50 µM	24 h	**0 ± 0, n=9**	**-14,11 ± 2,831**	**-20,11 to -8,11**	**0,0001**	*******	**0,6083**
	27	25 µM	24 h	11,5 ± 0,9916, n=6	-2,611 ± 3,6	-10,39 to 5,165	0,4811	n.s.	0,0389
		50 µM	24 h	**30,67 ± 3,252, n=6**	**16,56 ± 4,371**	**7,112 to 26**	**0,0023**	******	**0,5246**
		25 µM	48 h	16 ± 1,384, n=9	1,889 ± 3,151	-4,791 to 8,569	0,5573	n.s.	0,02197
Number of movements per 100 ms	Control			3,475 ± 0,3192, n=9	n.a.	n.a.	n.a.	n.a.	n.a.
	1	25 µM	24 h	**1,916 ± 0,3504, n=9**	**-1,559 ± 0,4739**	**-2,563 to -0,554**	**0,0046**	******	**0,4034**
		25 µM	48 h	**0,2435 ± 0,1615, n=9**	**-3,231 ± 0,3577**	**-3,989 to -2,473**	**<0,0001**	********	**0,8361**
		50 µM	24 h	**0 ± 0, n=9**	**-3,475 ± 0,3192**	**-4,151 to -2,798**	**<0,0001**	********	**0,8811**
	27	25 µM	24 h	3,951 ± 0,246, n=6	0,4767 ± 0,4422	-0,4786 to 1,432	0,3006	n.s.	0,08205
		50 µM	24 h	3,877 ± 0,2249, n=6	0,4019 ± 0,4349	-0,5376 to 1,341	0,3722	n.s.	0,06165
		25 µM	48 h	4,02 ± 0,2617, n=9	0,545 ± 0,4127	-0,3299 to 1,42	0,2052	n.s.	0,09828

c. **Measurement of movement amplitude after touch-evoked escape response**

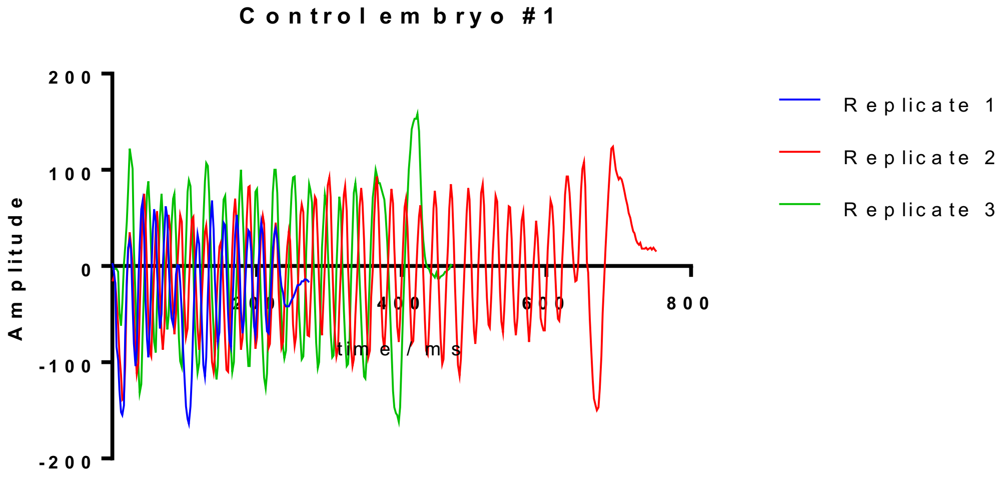

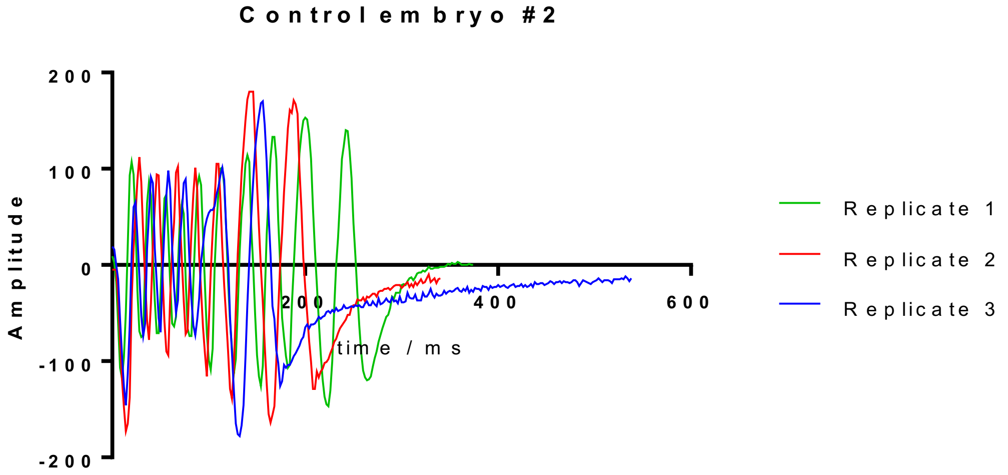

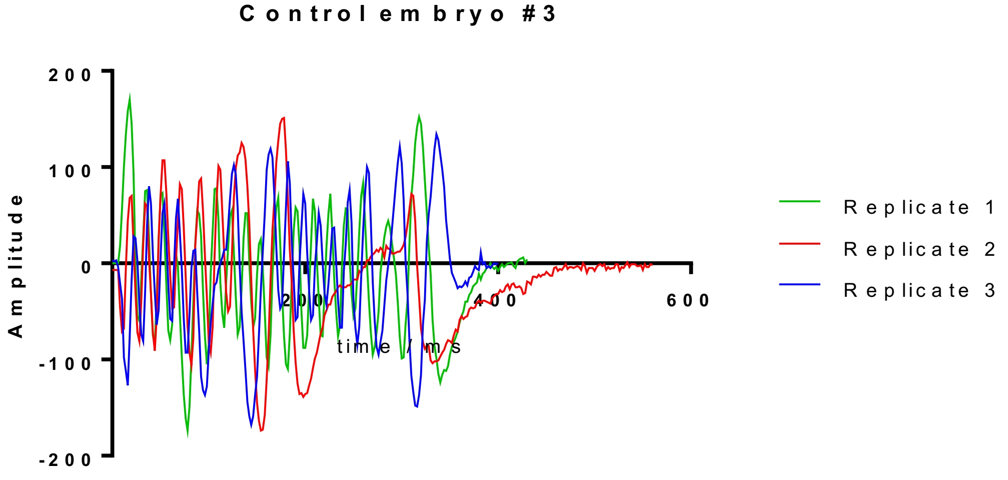

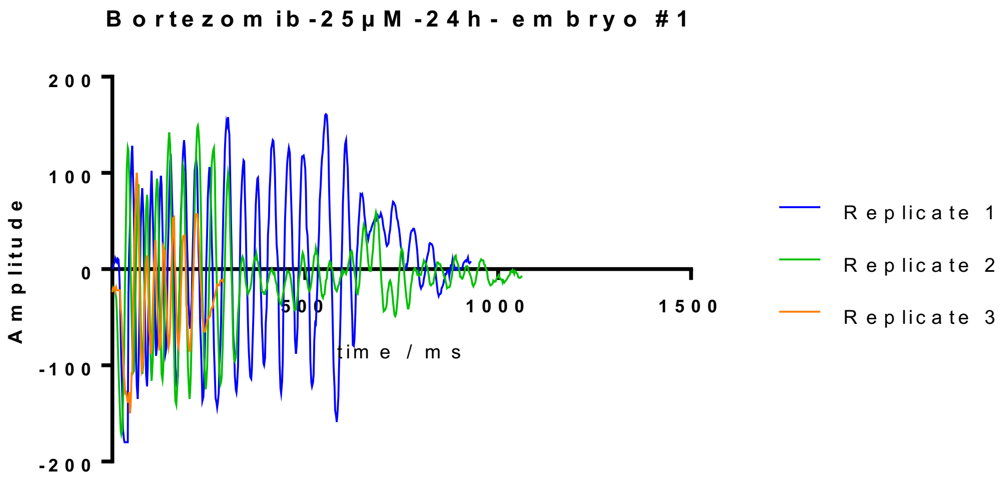
Bortezomib-25µM-24h-embryo #1
200
100
0
-100
-200
Amplitude
500
1000
1500
time / ms
Replicate 1
Replicate 2
Replicate 3

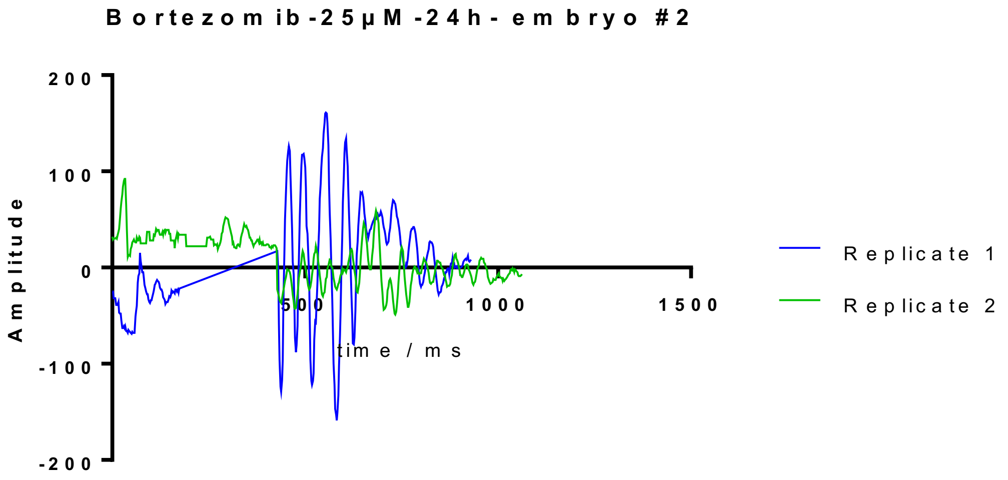
Bortezomib-25µM-24h-embryo #2
200
100
0
-100
-200
Amplitude
500
1000
1500
time / ms
Replicate 1
Replicate 2

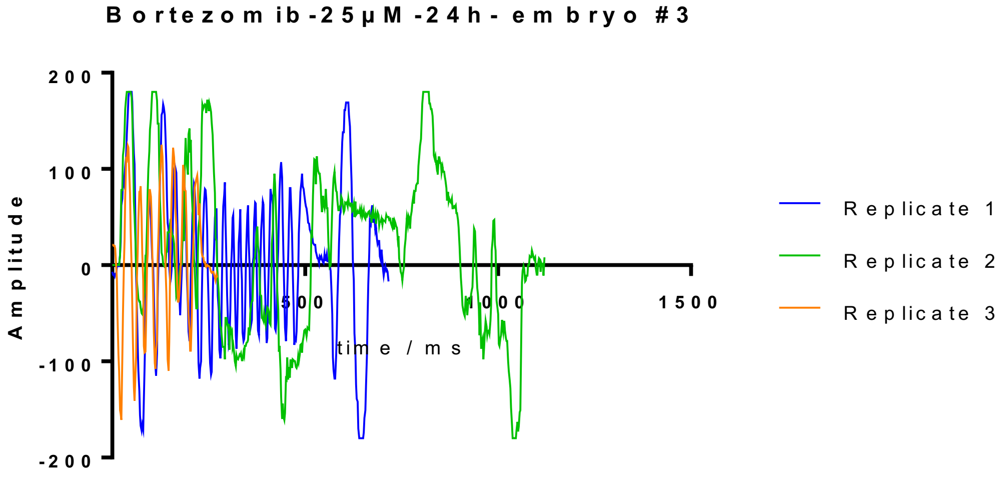
Bortezomib-25µM-24h-embryo #3
200
100
0
-100
-200
Amplitude
500
1000
1500
time / ms
Replicate 1
Replicate 2
Replicate 3

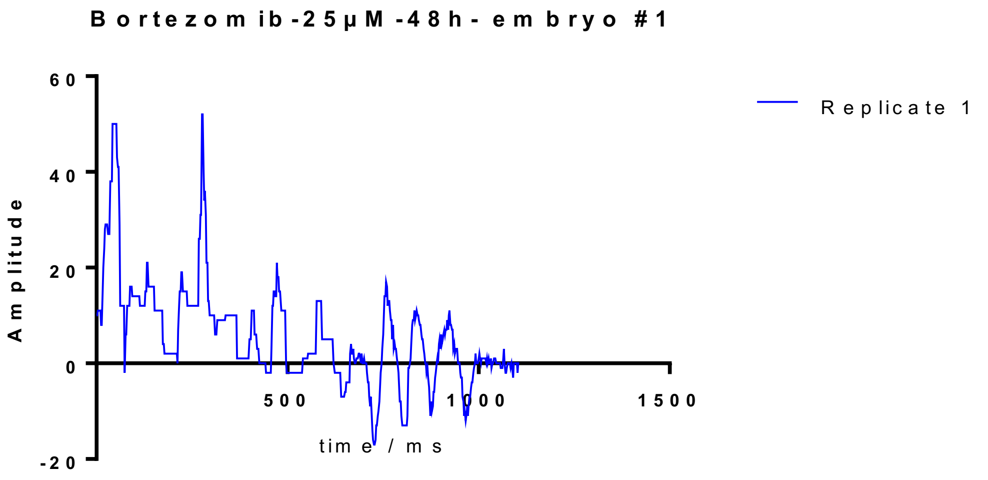
Bortezomib-25µM-48h-embryo #1
Amplitude
time / ms
60
40
20
0
-20
500
1000
1500
Replicate 1

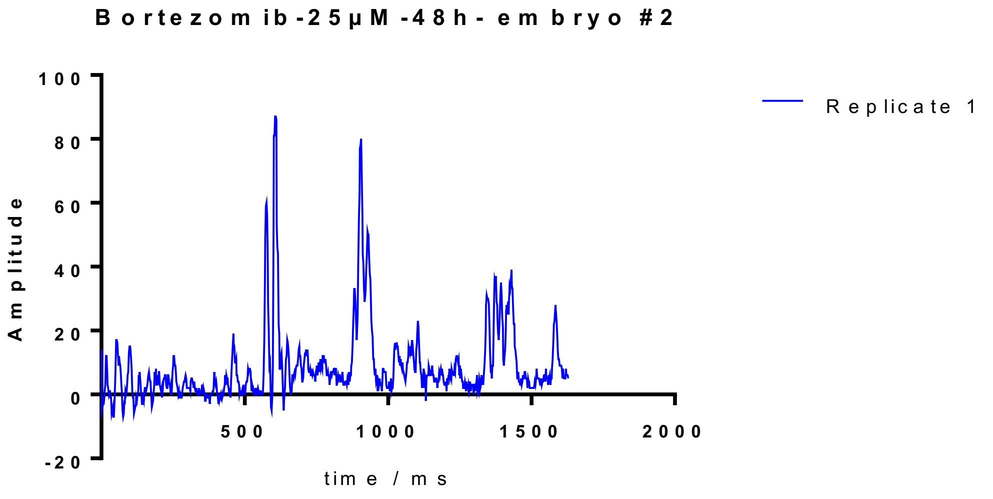
Bortezomib-25µM-48h-embryo #2
Amplitude
time / ms
100
80
60
40
20
0
-20
500
1000
1500
2000
Replicate 1

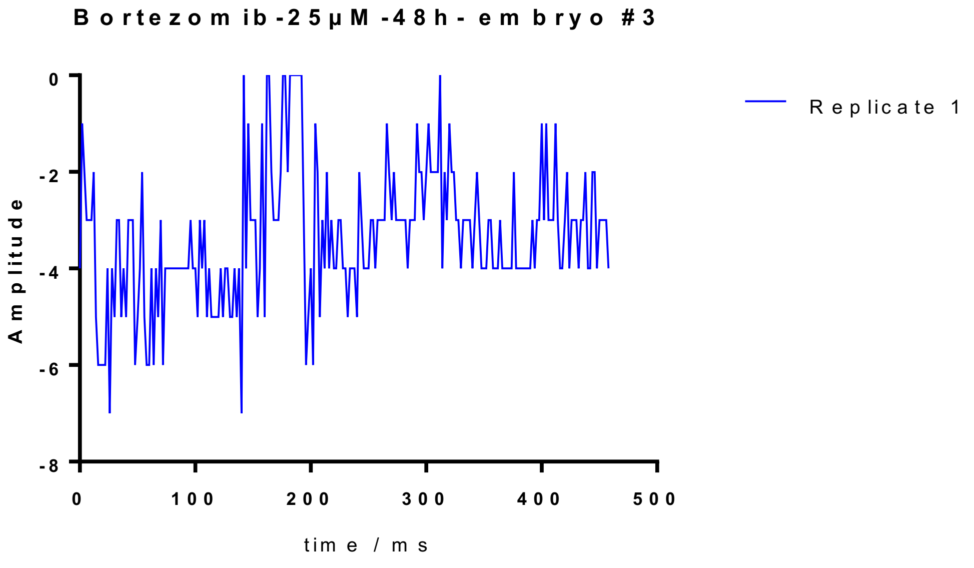
Bortezomib-25µM-48h-embryo #3
Amplitude
time / ms
0
-2
-4
-6
-8
0
100
200
300
400
500
Replicate 1

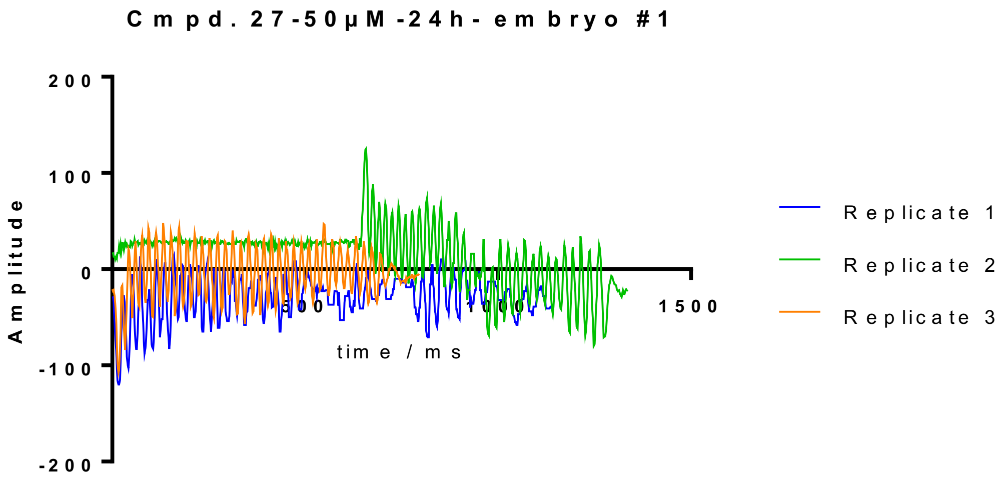
Cmpd. 27-50µM-24h- embryo #1
200
100
0
-100
-200
Amplitude
500
1000
1500
time / ms
Replicate 1
Replicate 2
Replicate 3

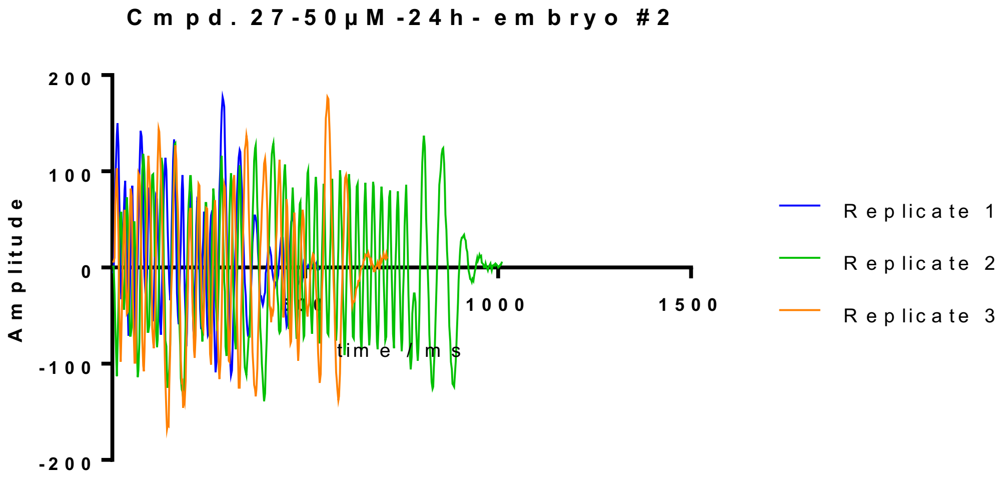
Cmpd. 27-50µM-24h- embryo #2
200
100
0
-100
-200
Amplitude
500
1000
1500
time / ms
Replicate 1
Replicate 2
Replicate 3

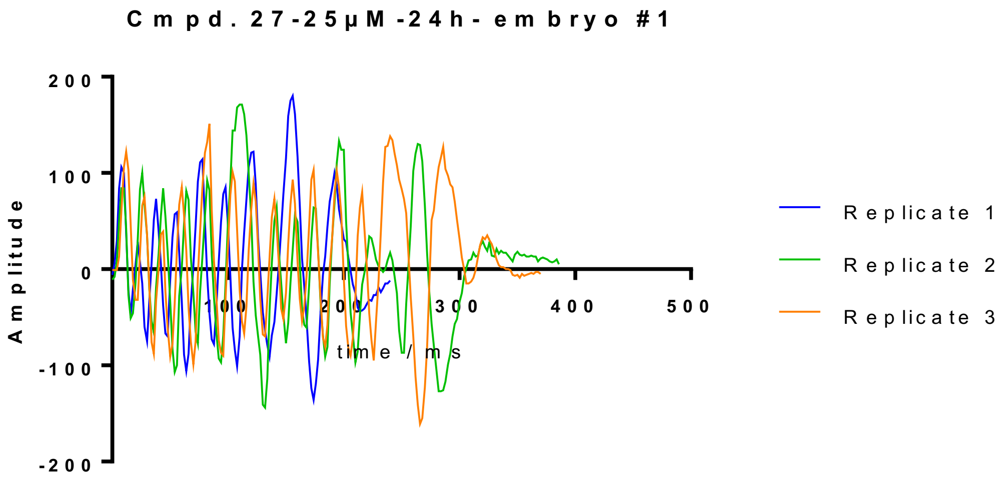
Cmpd. 27-25µM-24h- embryo #1
200
100
0
-100
-200
Amplitude
100
200
300
400
500
time / ms
Replicate 1
Replicate 2
Replicate 3

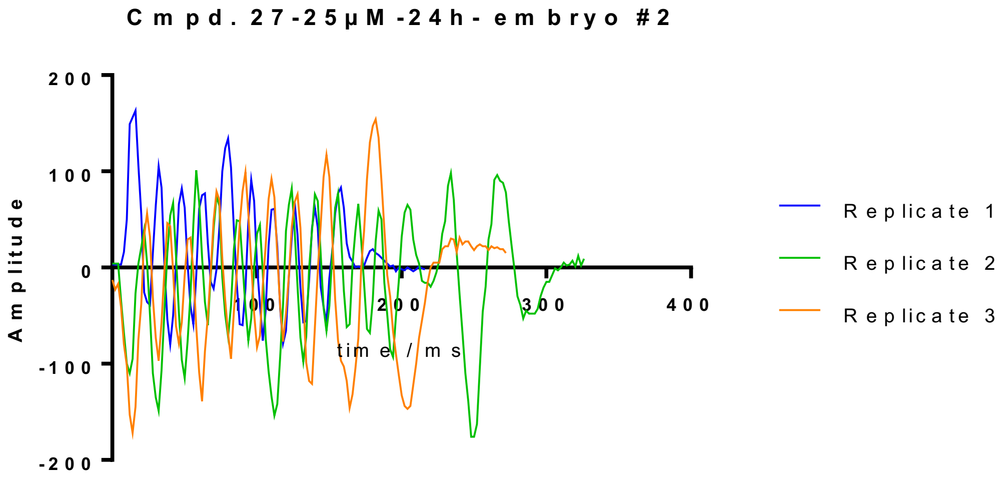
Cmpd. 27-25µM-24h- embryo #2
200
100
0
-100
-200
Amplitude
100
200
300
400
time / ms
Replicate 1
Replicate 2
Replicate 3

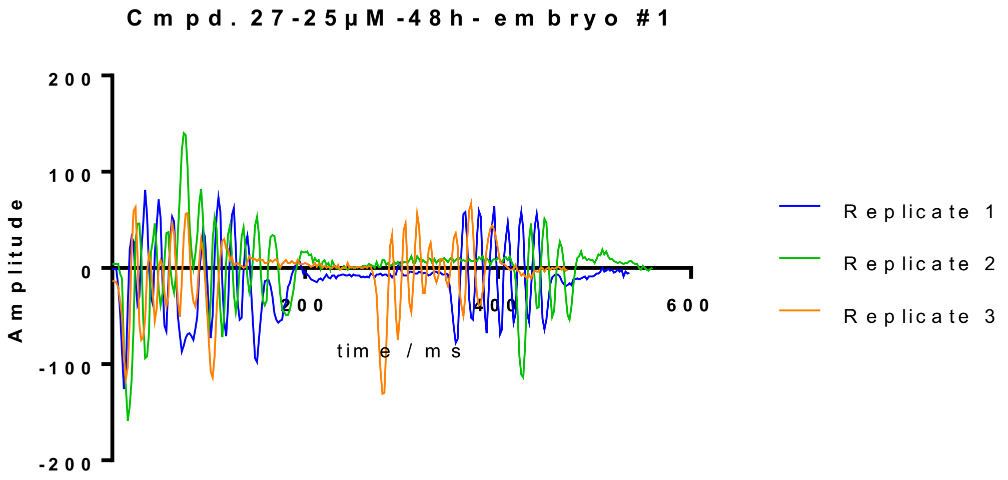
Cmpd. 27-25µM-48h-embryo #1
200
100
0
-100
-200
Amplitude
200
400
600
time / ms
Replicate 1
Replicate 2
Replicate 3

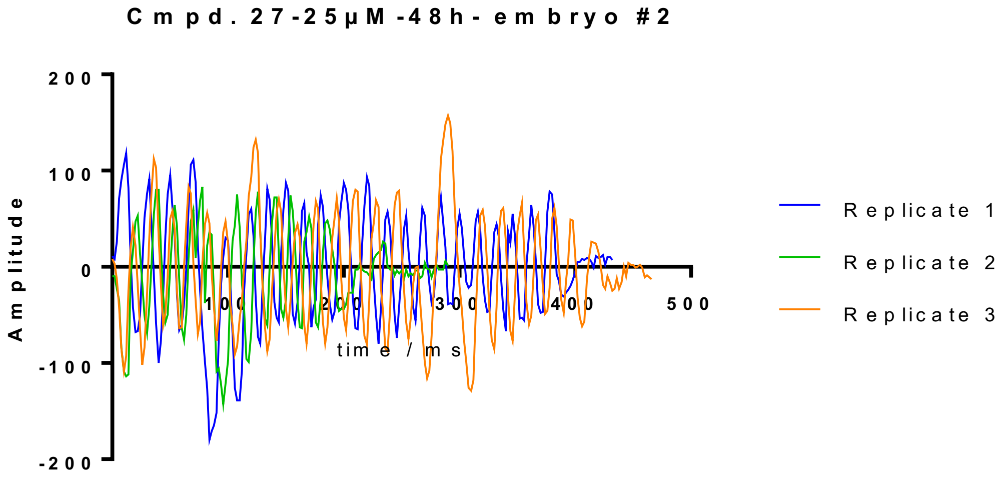
Cmpd. 27-25µM-48h-embryo #2
200
100
0
-100
-200
Amplitude
100
200
300
400
500
time / ms
Replicate 1
Replicate 2
Replicate 3

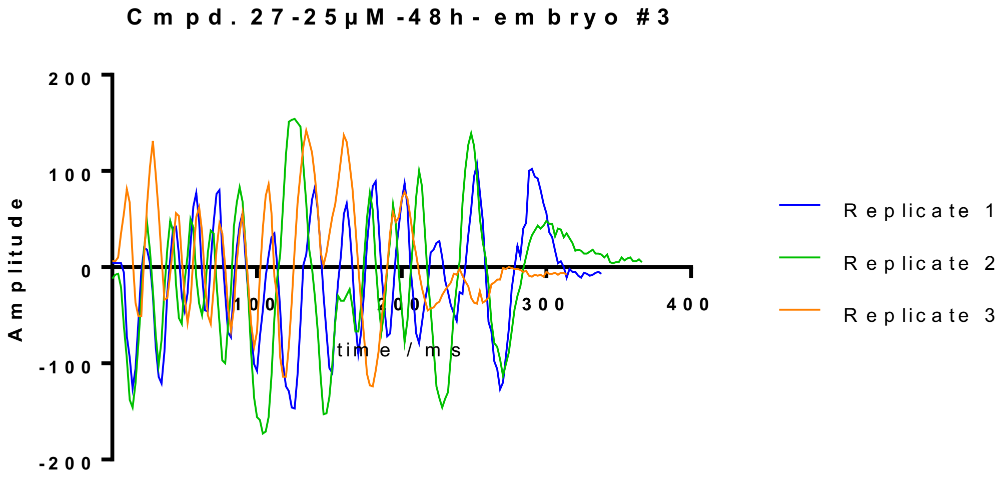
Cmpd. 27-25µM-48h-embryo #3
200
100
0
-100
-200
Amplitude
100
200
300
400
time / ms
Replicate 1
Replicate 2
Replicate 3

C-Bend Amplitude

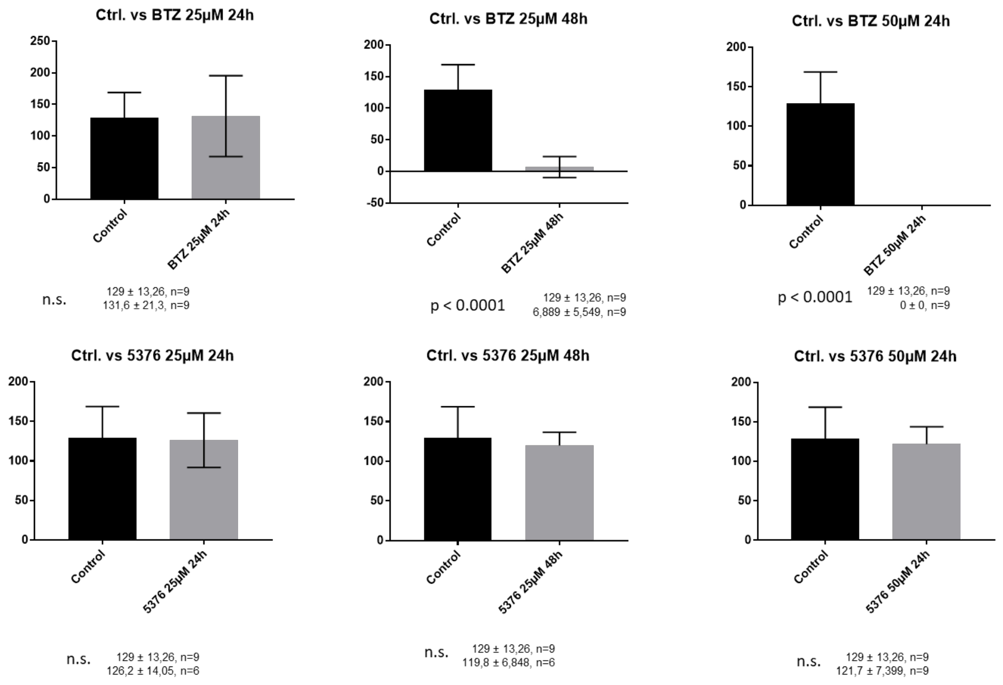

Figure S19: Comparison of the c-bend amplitude in degrees.

C-Bend Duration

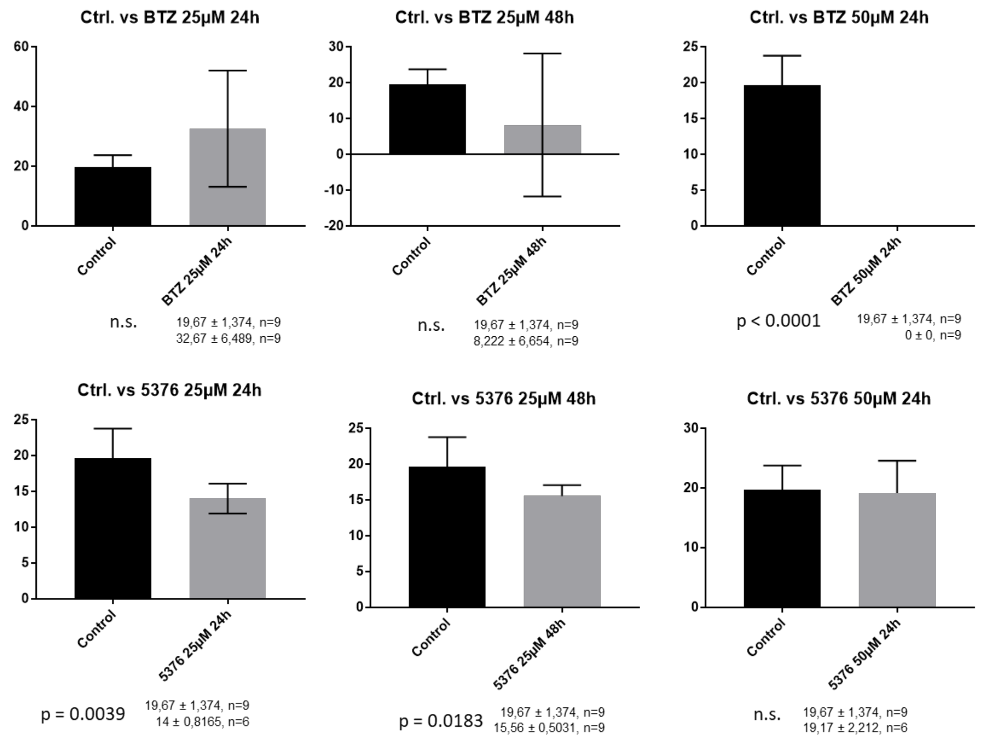

Figure S20: Comparison of the c-bend duration in milliseconds.

Reflex Duration

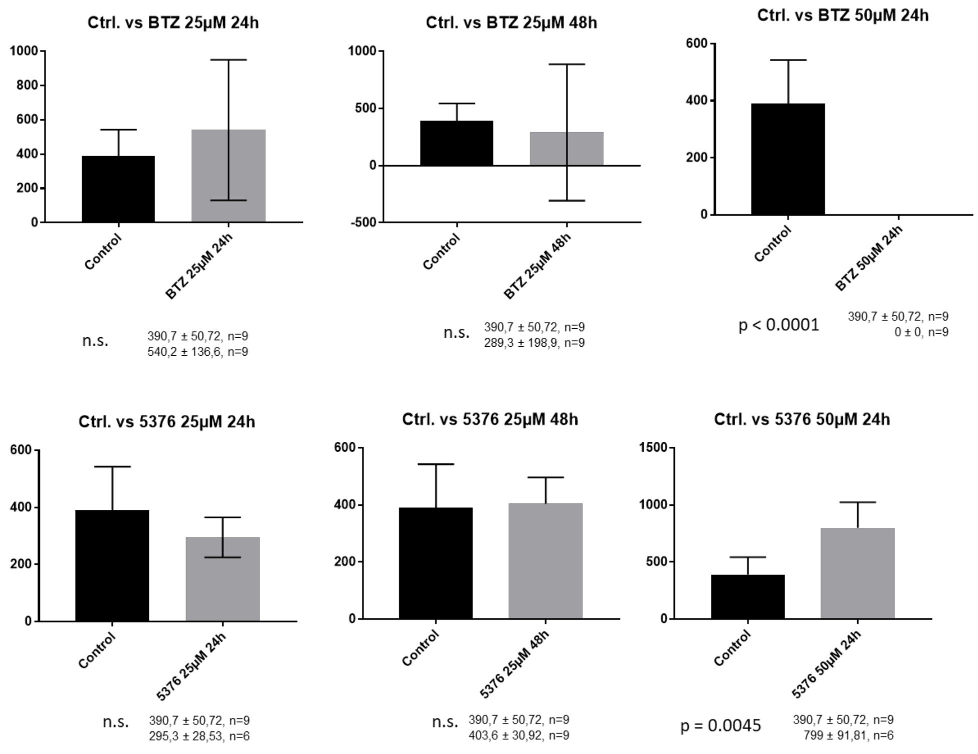

Figure S21 Comparison of the absolute reflex duration in milliseconds.

No of movements

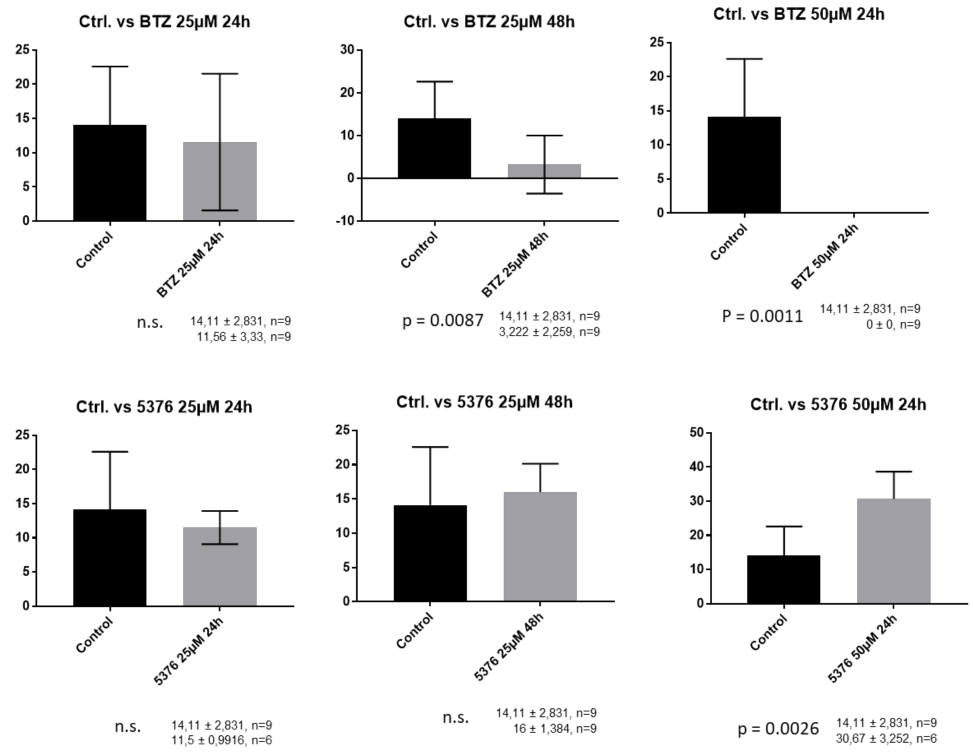

Figure S22: Comparison of the total number of movements per reflex.

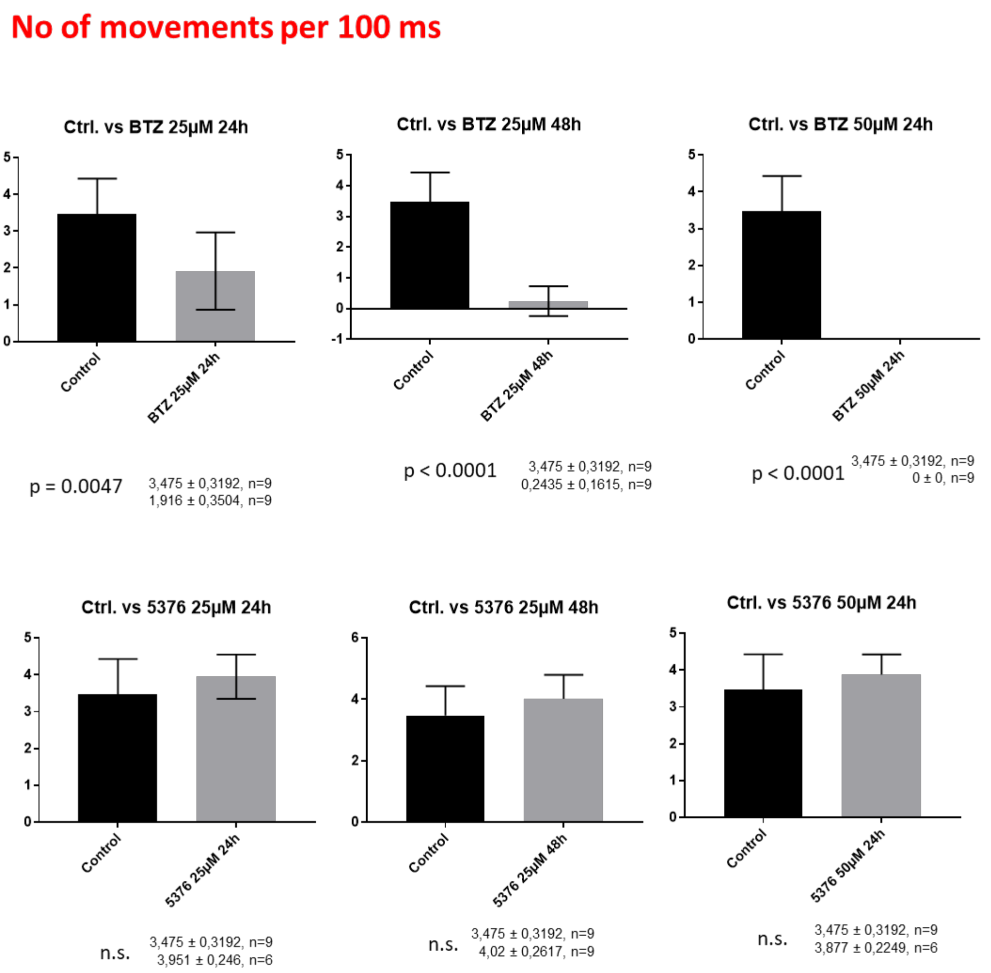

Figure S23: Comparison of the number of movements per 100 ms.

e. BODIPY Characterization

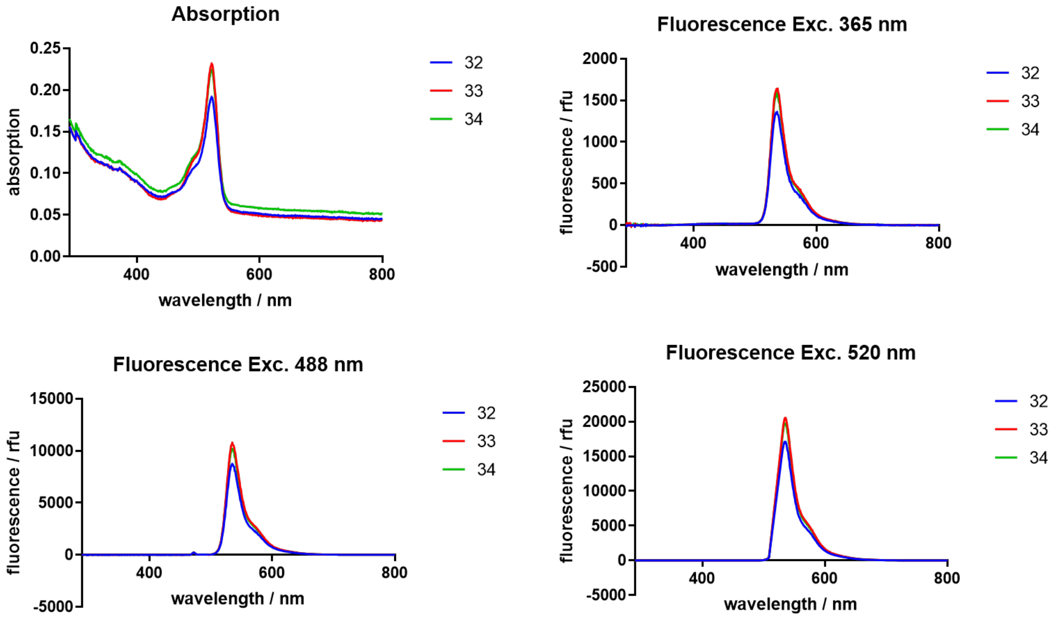

Figure S24: Absorption and fluorescence emission spectra of compounds **32** – **34**.

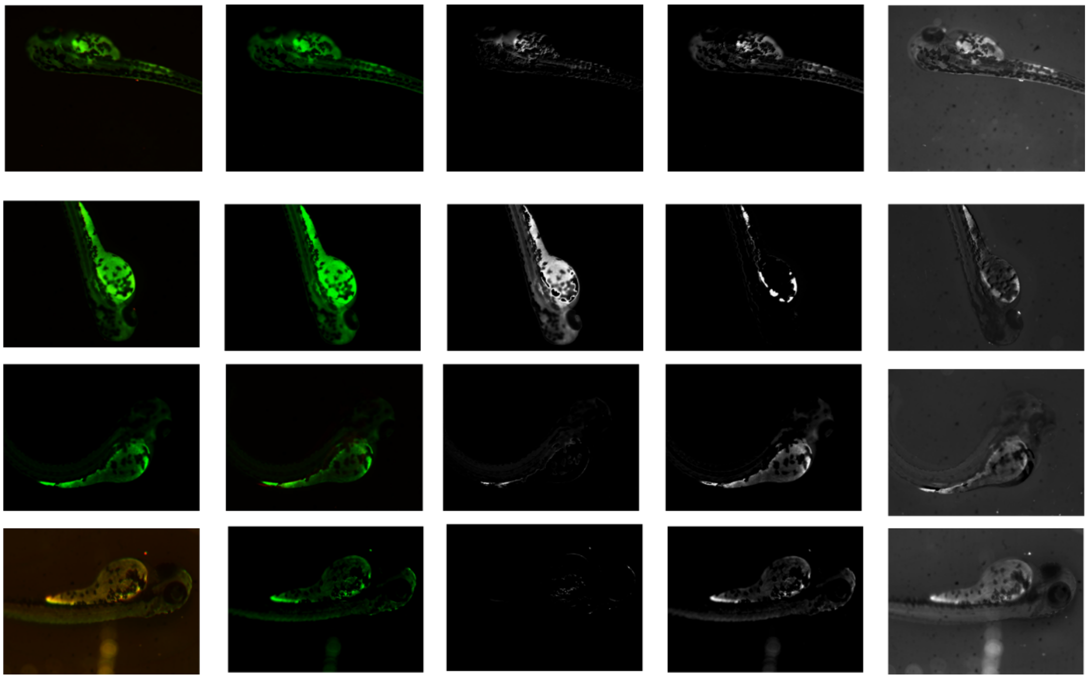

Figure S25: Imaging of Danio rerio embryos treated with BODIPY-conjugated fluorophores **32** (top), **33** (2nd row) and **34** (3rd row) as well as control embryo (bottom row). Images are recorded using the Nuance Fx Imaging system (3225.5 ms exposure time, Filter AF488, 5x magnification). Left column: Mixed RGB image; 2nd row: unmixed image; 3rd row: Signal 1; 4th row: Signal 2; right row: background.

3. Molecular Modelling

a. Structural analysis of BSc4999 (7) and covalent redocking using Docktite

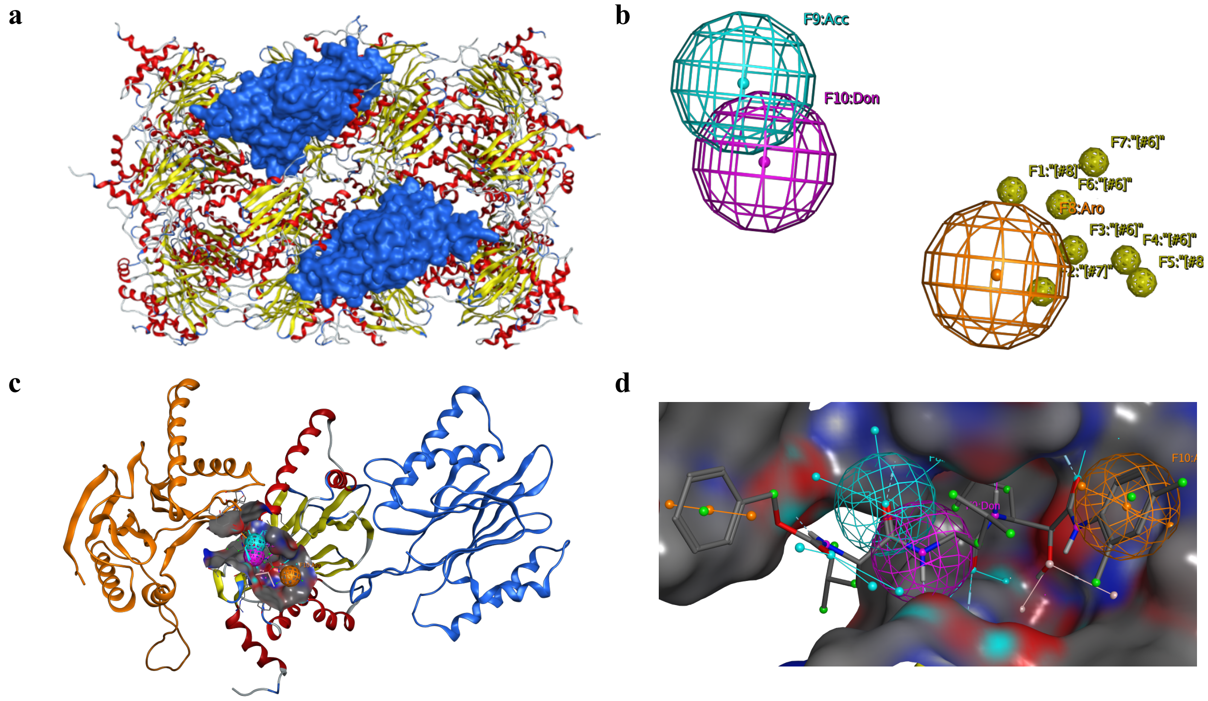

Figure S26: (a) Overview of proteasome architecture and location of the β5 subunit (blue). (b) Pharmacophore features defined using the pose obtained from cocrystallized structure (PDB: 4r02, R = 2.0 Å for donor, acceptor and aromatic features; R = 0.4 Å for atom pharmacophores of Thr1 (yellow)).

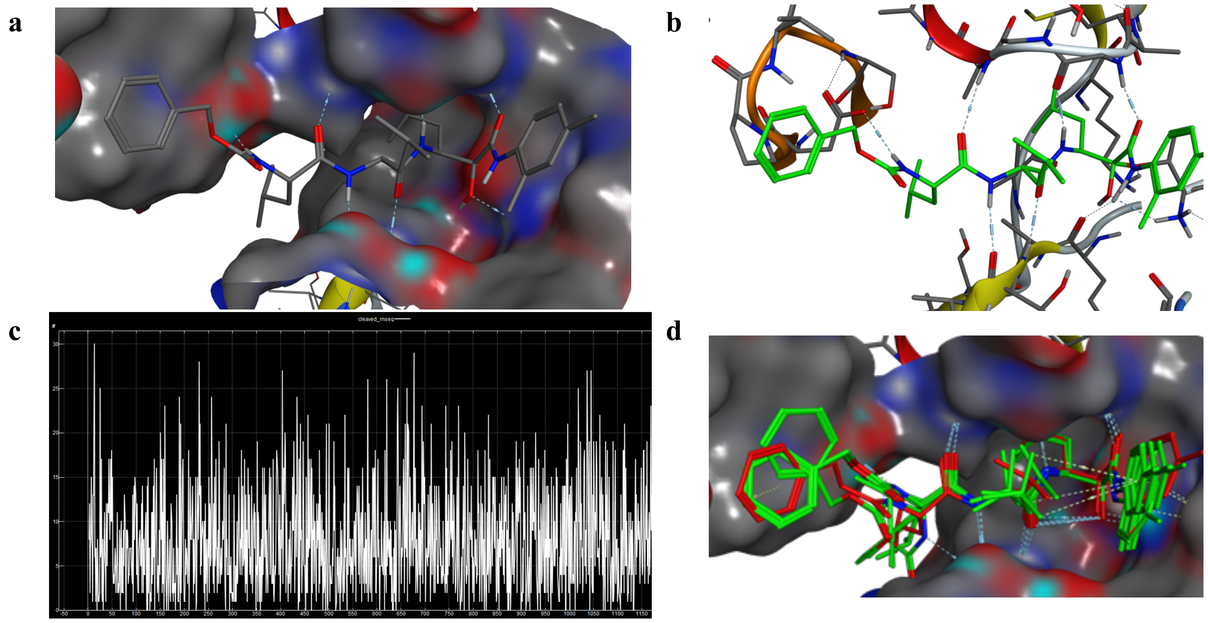

Figure S27: (a) Overview of binding of **7** highlighting the molecular surface of the receptor or (b) highlighting important interactions in the yCP β5 subunit (obtained from PDB: 4r02). (c) Distribution of number of output poses per input conformation for redocking of **7**. (d) Redocked poses of **7** in β5c (RMSD < 2.0 Å, 7 poses in Top1% scored poses).

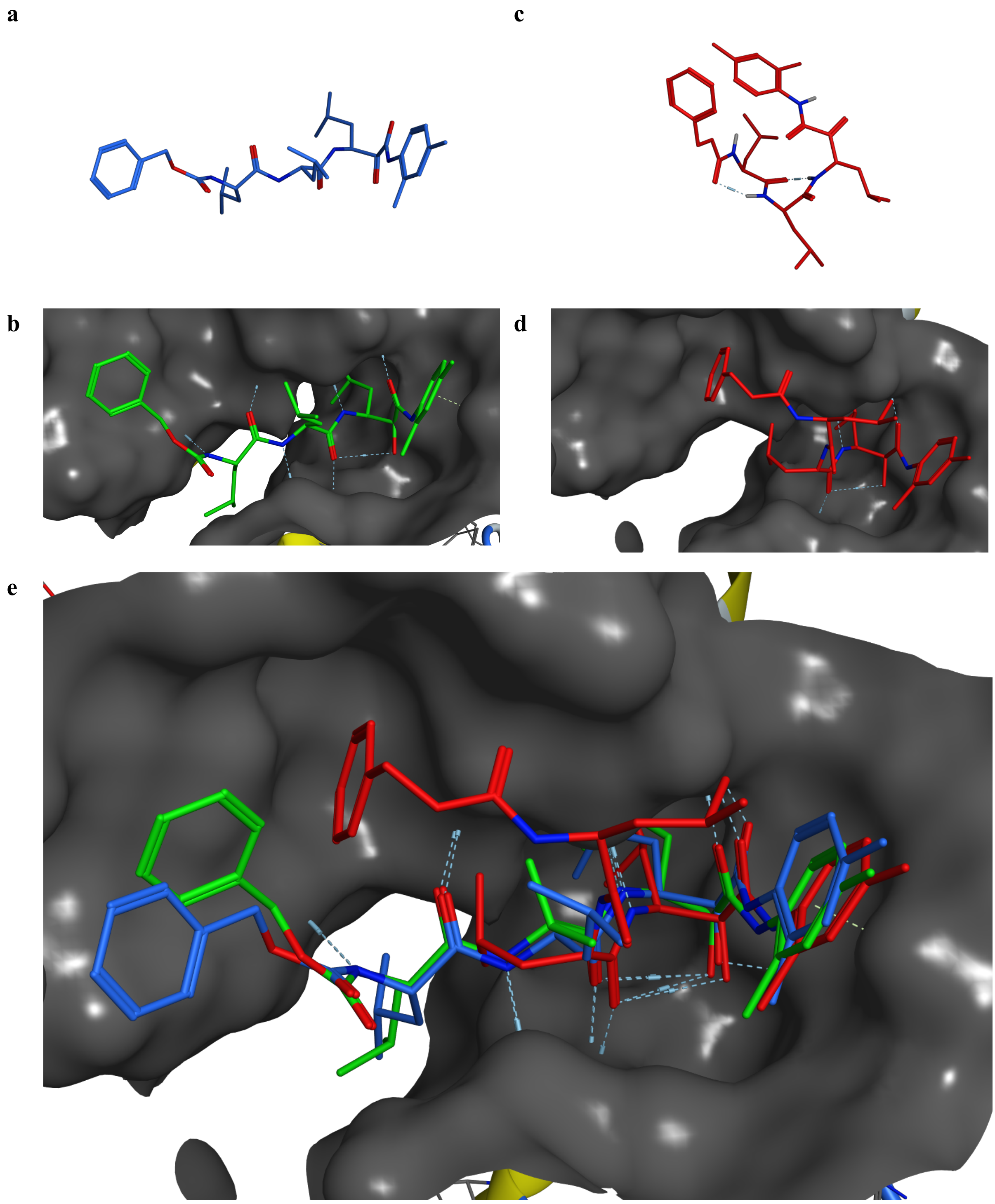

Figure S28: Conformations of input structures of **7**: (a) native and (b) random conformation. (c) Lowest energy pose obtained from redocking of native pose (London dG, RMSD =1.3 Å). (d) Lowest energy pose obtained from redocking of random pose (London dG, RMSD =5.2 Å). (e) Superposition of cocrystallized structure (blue) of **7** as well as top-scored pose obtained from redocking using native input conformation (green) and a random input conformation (red).

b. Energy-minimized binding poses of phenoxy and P4-substituted ketoamides

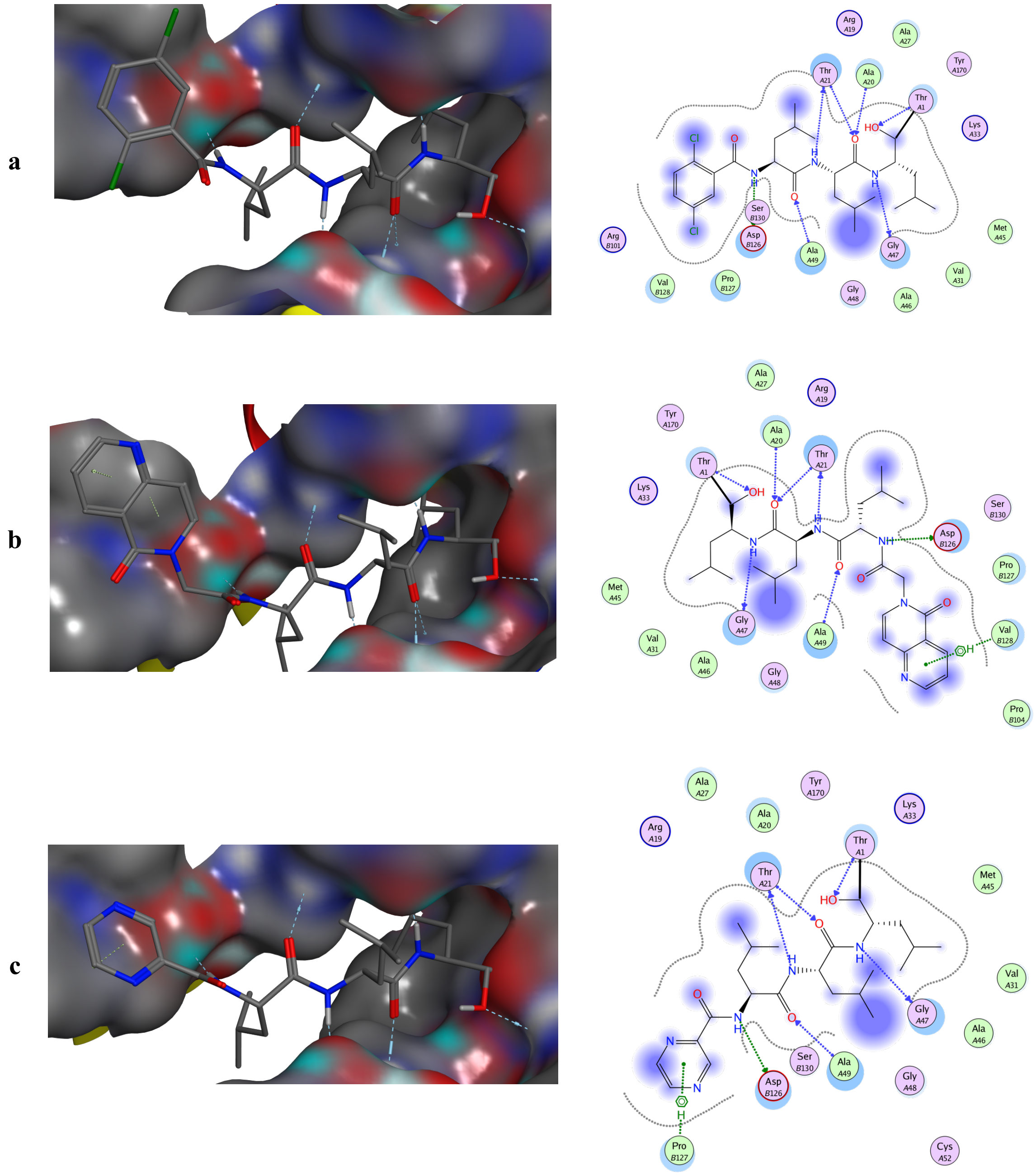

Figure S29: Energy-minimized poses and ligand interaction of aldehydes (a) **24**, (b) **22**, (c) **23** binding in the β5c subunit.

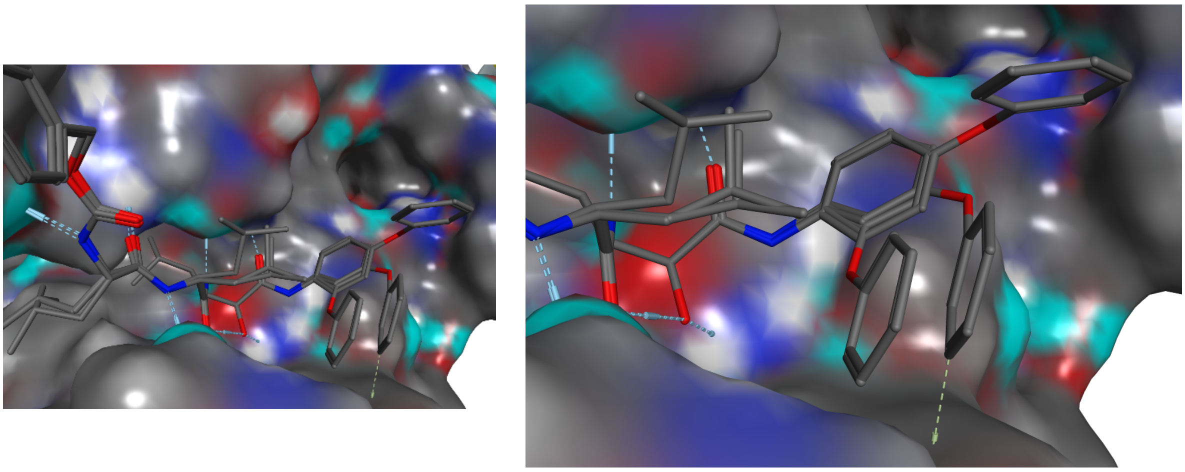

Figure S30: Superposition of energy-minimized poses of 2-, 3- and 4-phenoxy substituted Ketoamides **8**, **9** and **10**.

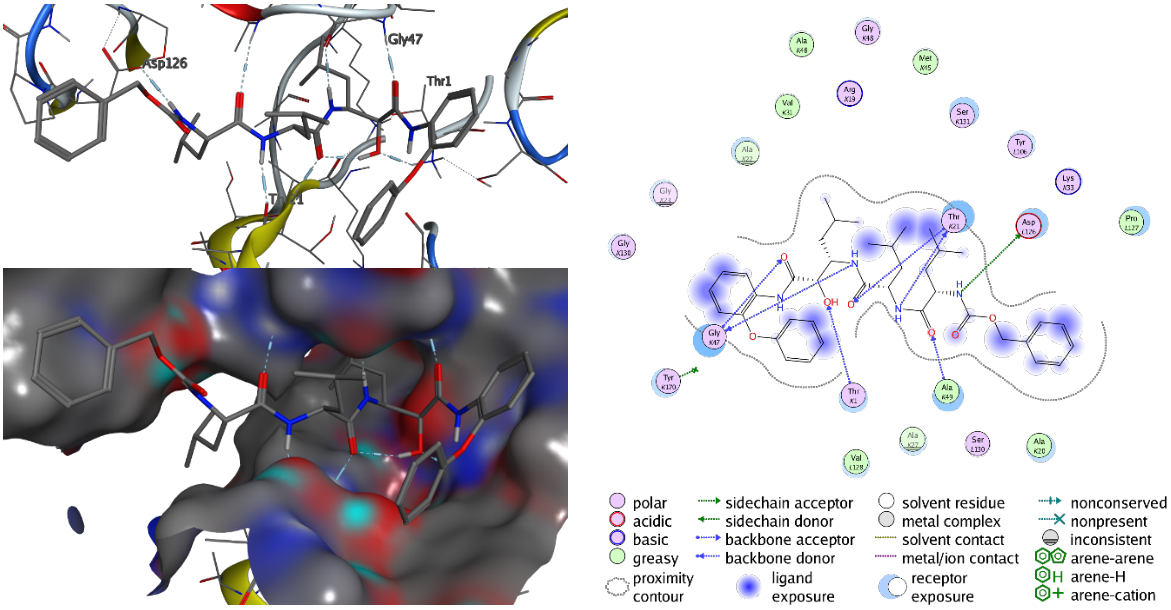

Figure S31: Energy-minimized pose of **8** highlighting key hydrogen bond interactions, molecular surface of the receptor and ligand interactions.

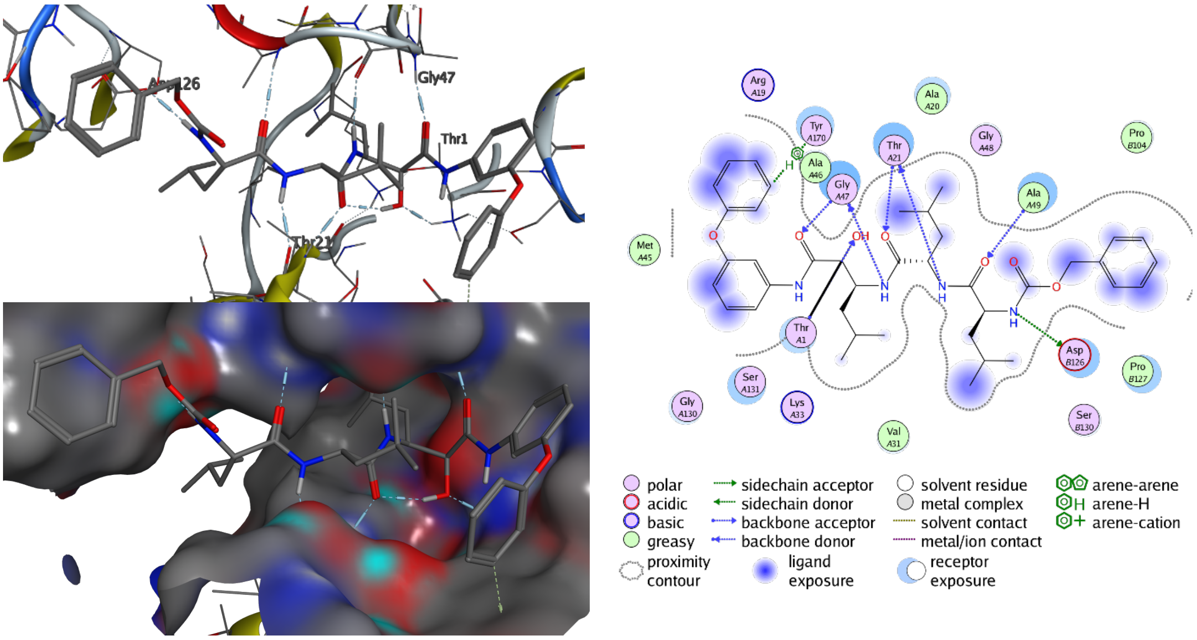

Figure S32: Energy-minimized pose of **9** highlighting key hydrogen bond interactions, molecular surface of the receptor and ligand interactions.

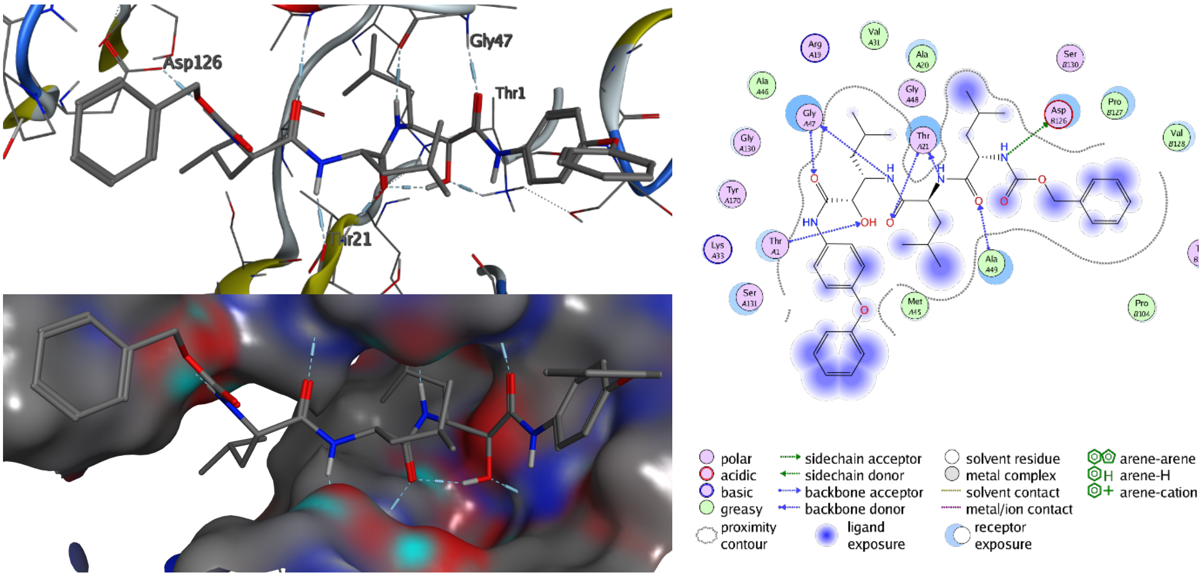

Figure S33: Energy-minimized pose of **10** highlighting key hydrogen bond interactions, molecular surface of the receptor and ligand interactions.

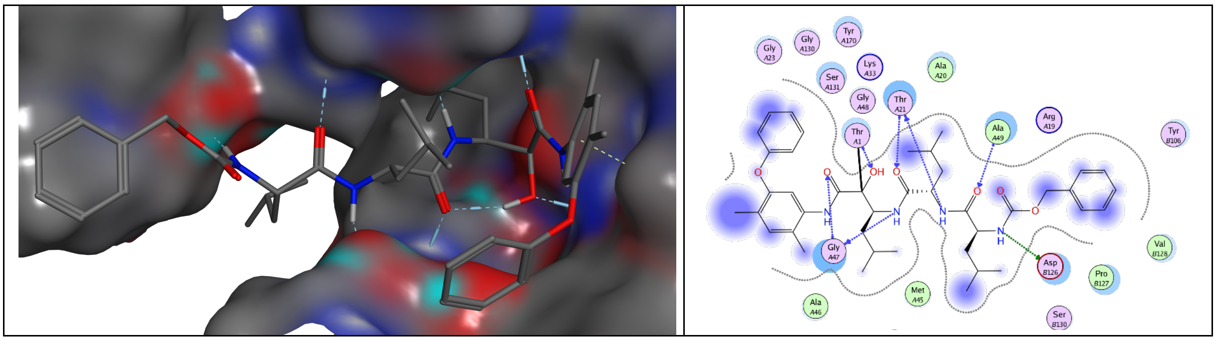

Figure S34: Energy-minimized pose of **11.**

c. Covalent docking of BSc5376 (27) using Docktite

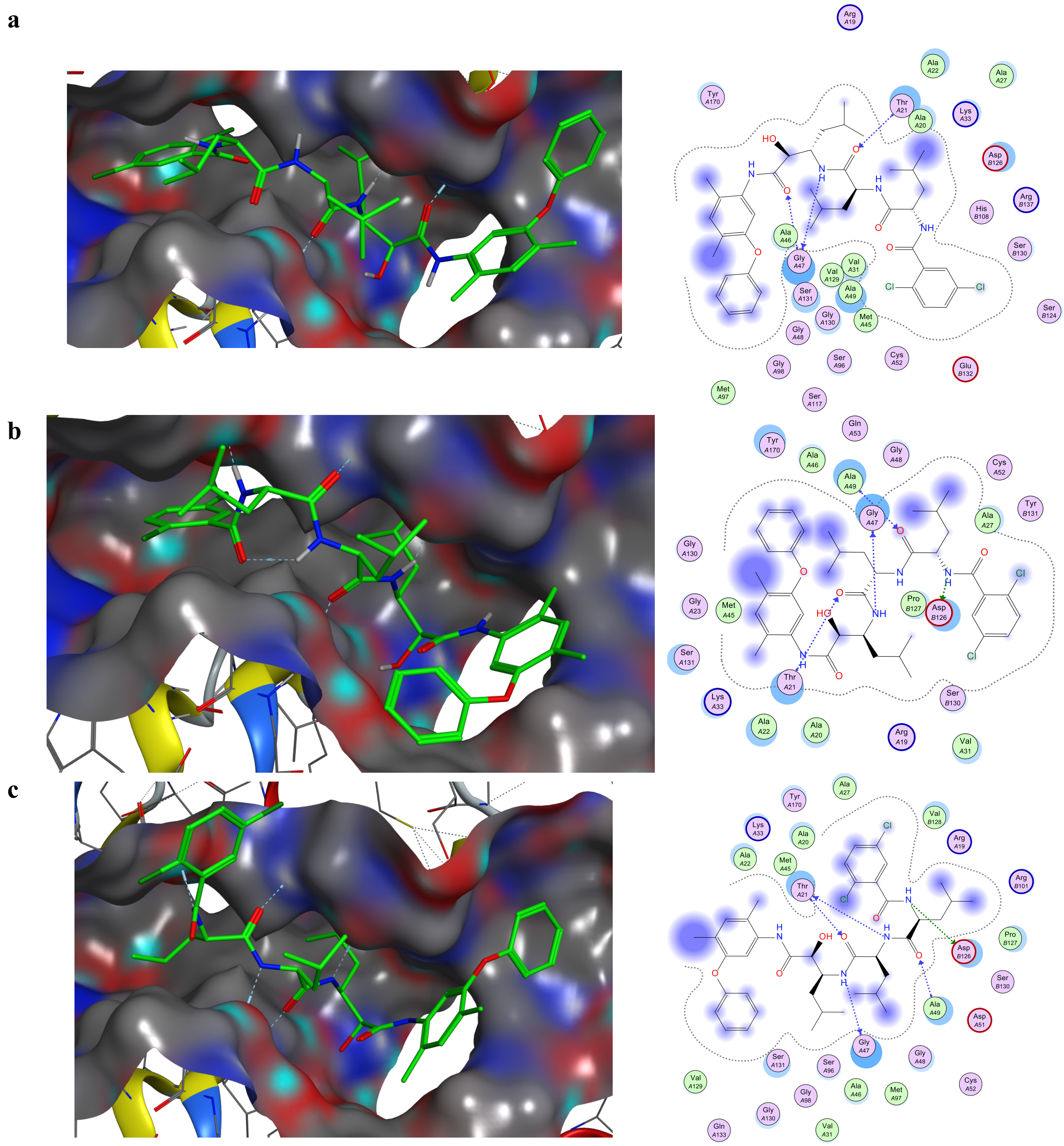

Figure S35: Overview of ligand interactions of top-scored poses of (a) ensemble 1, (b) ensemble 2a, (c) ensemble 2b.

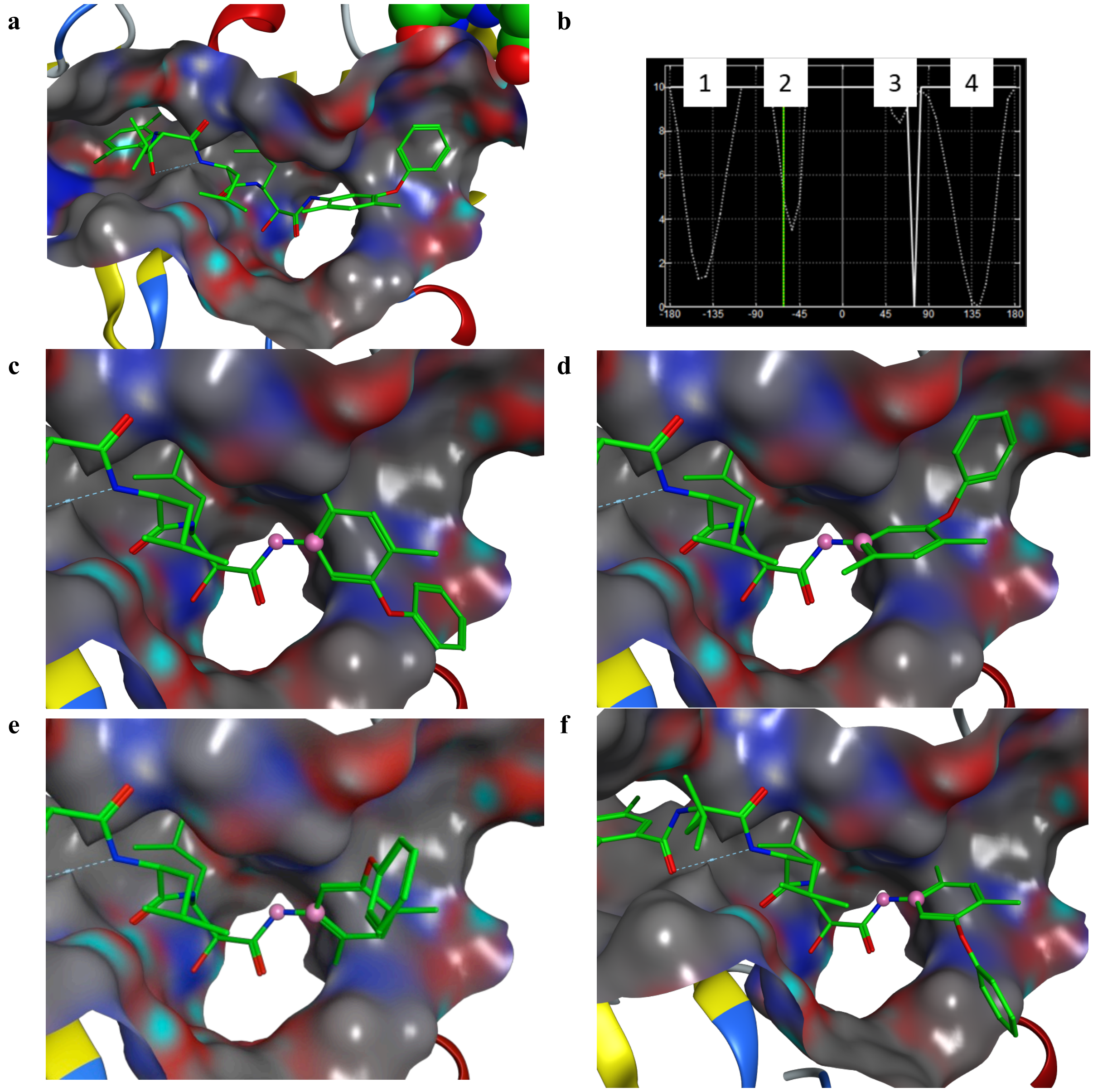

Figure S36: (a) Overview of top-scored docked pose of **27**. (b) Dihedral energy profile of C-N bond between phenyl ring and the amide group. (c) **1** (d) **2** (e) **3** and (f) **4**.

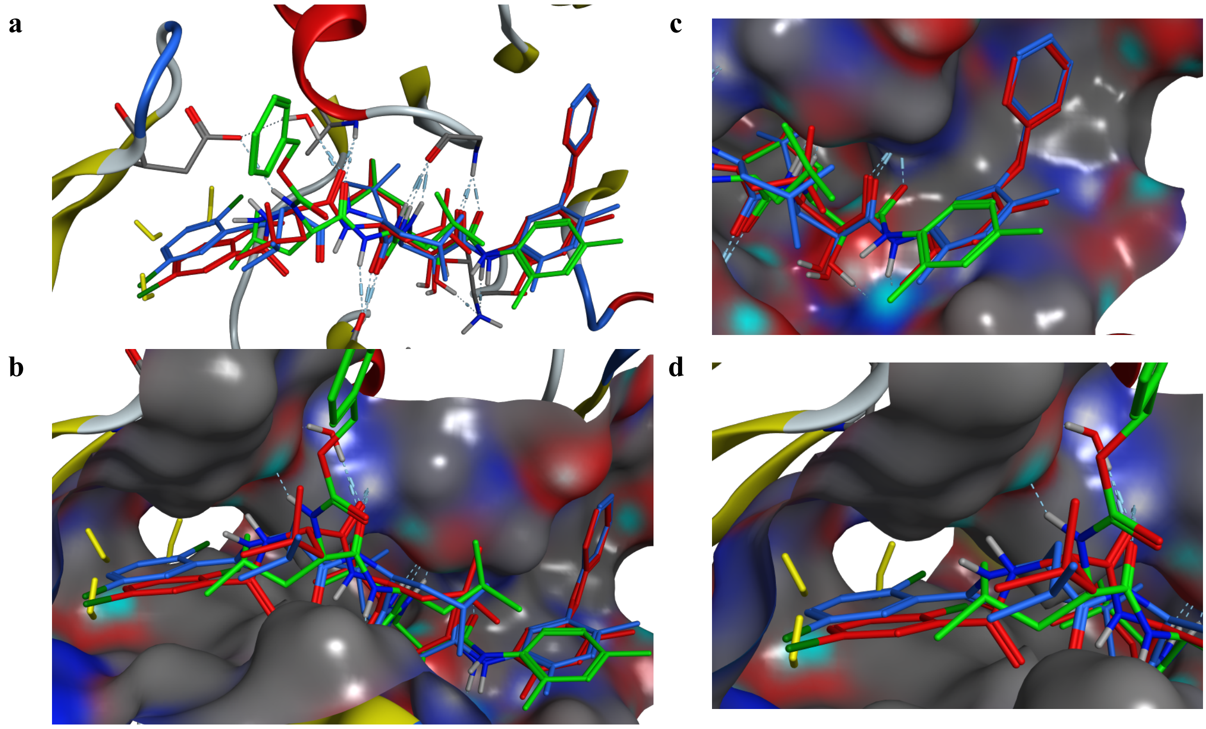

Figure S37: (a) Compound **7** superposed with top-ranked and fourth-ranked pose of **27** (2 possible amide orientations). (b) Similar conformations of **27** (pose 1, pose 4) targeting the primed site. (c) Altered orientation of P3 group (d) close-up of 3 displaced water molecules in P3 pocket

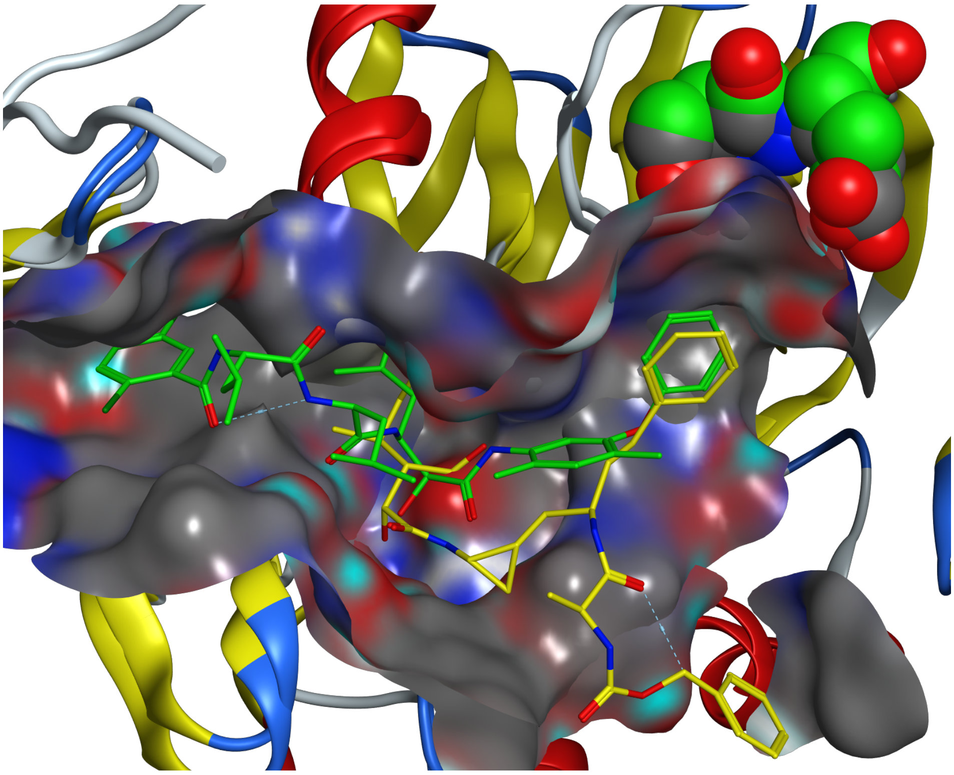

Figure S38: Superposition of top-scored docking pose of **27** (green) and homobelactosin C cocrystal structure (grey, PDB: 4J70) highlighting the non-conserved residues in the S1' site (Ser117, Asp118 in yCP).

4. Appendix

NMR-Spectra

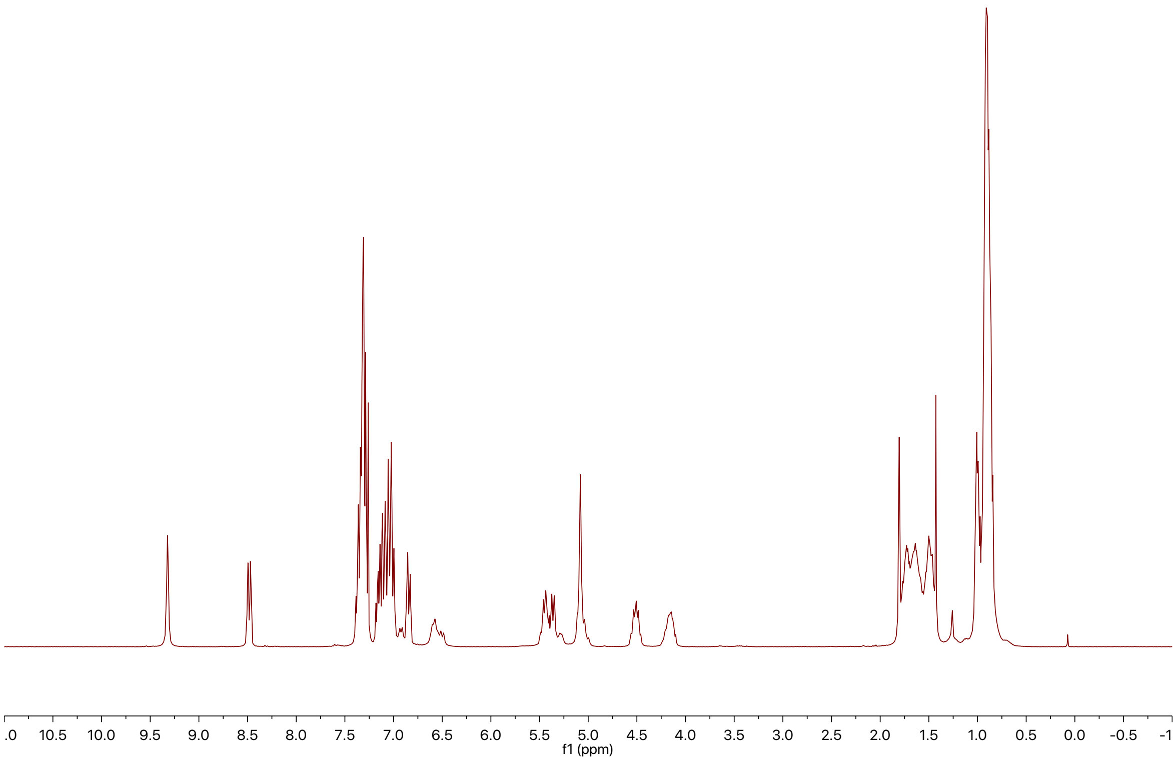

^{1}H-NMR (300 MHz, $CDCl_3$) of compound 8.

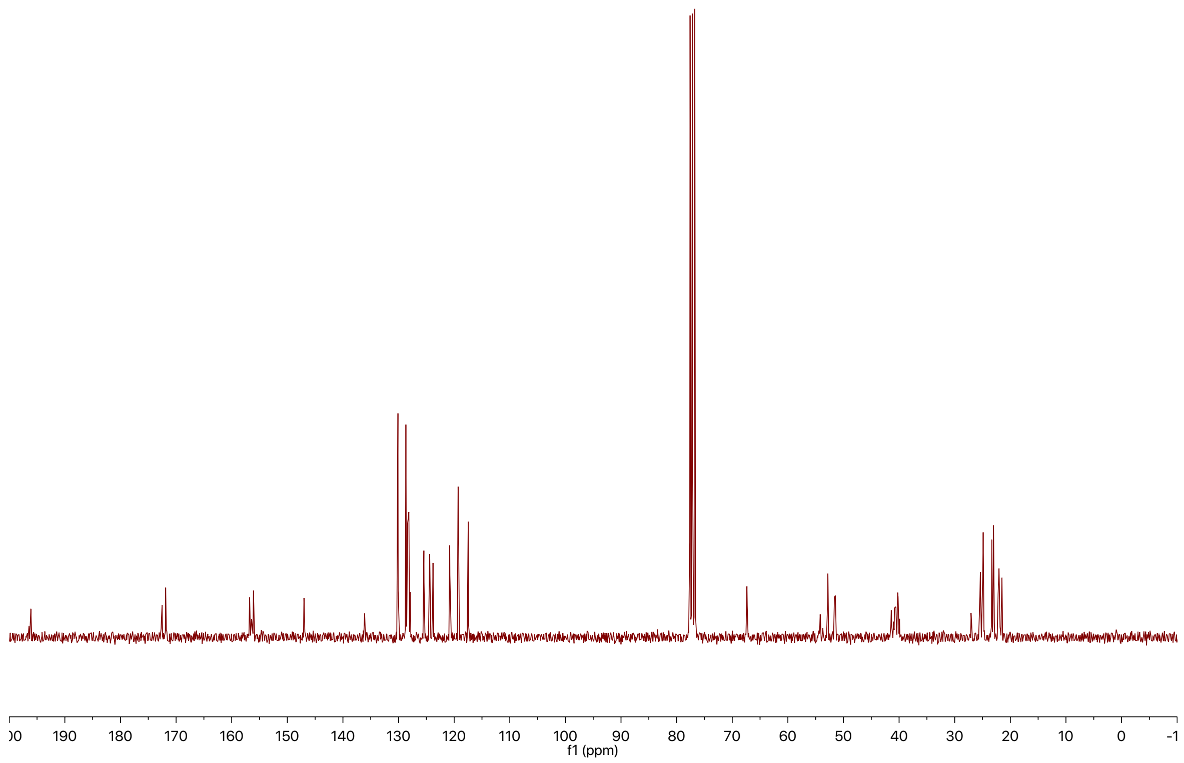

^{13}C-NMR (75 MHz, $CDCl_3$) of compound 8.

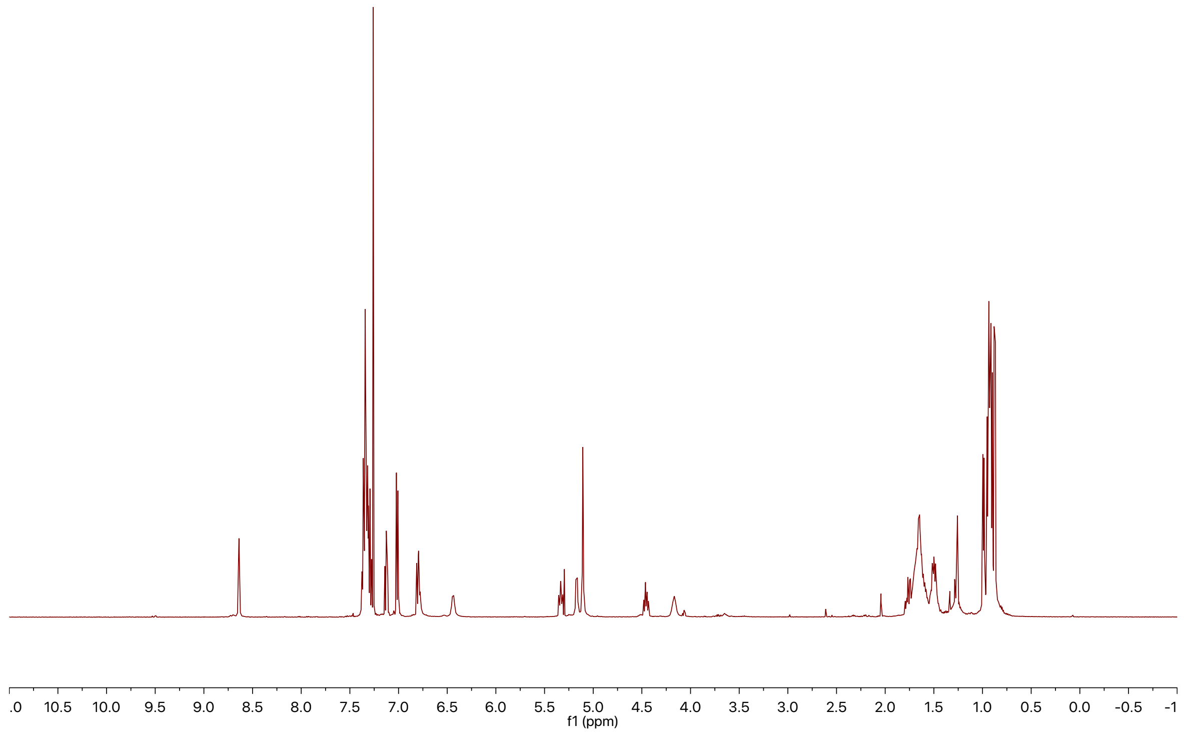

^{1}H-NMR (500 MHz, $CDCl_3$) of compound 9.

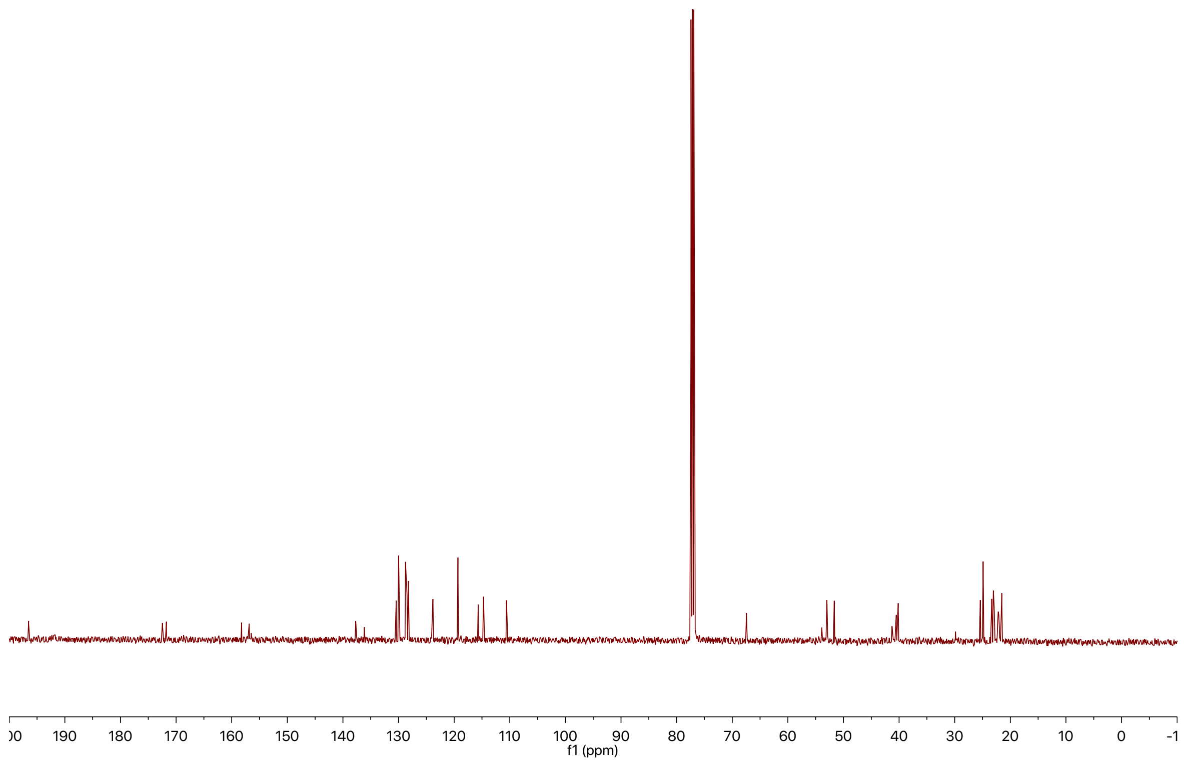

^{13}C-NMR (126 MHz, $CDCl_3$) of compound 9.

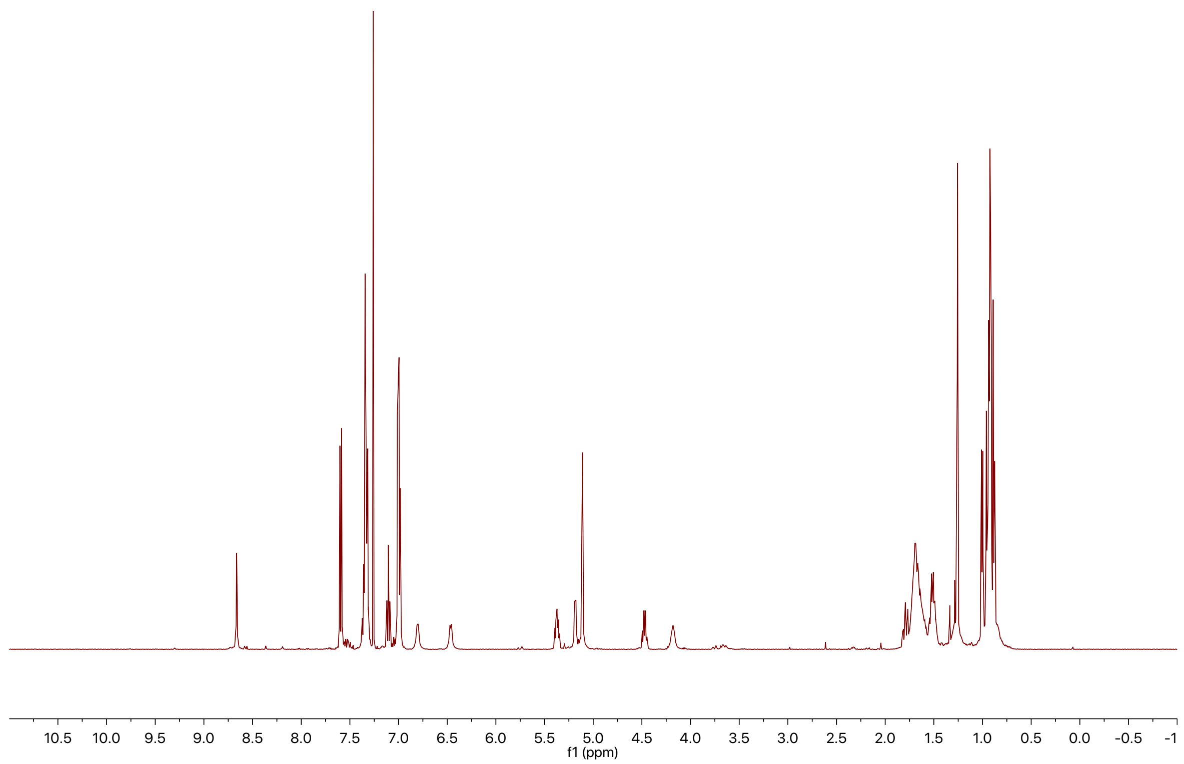

^{1}H-NMR (500 MHz, $CDCl_3$) of compound 10.

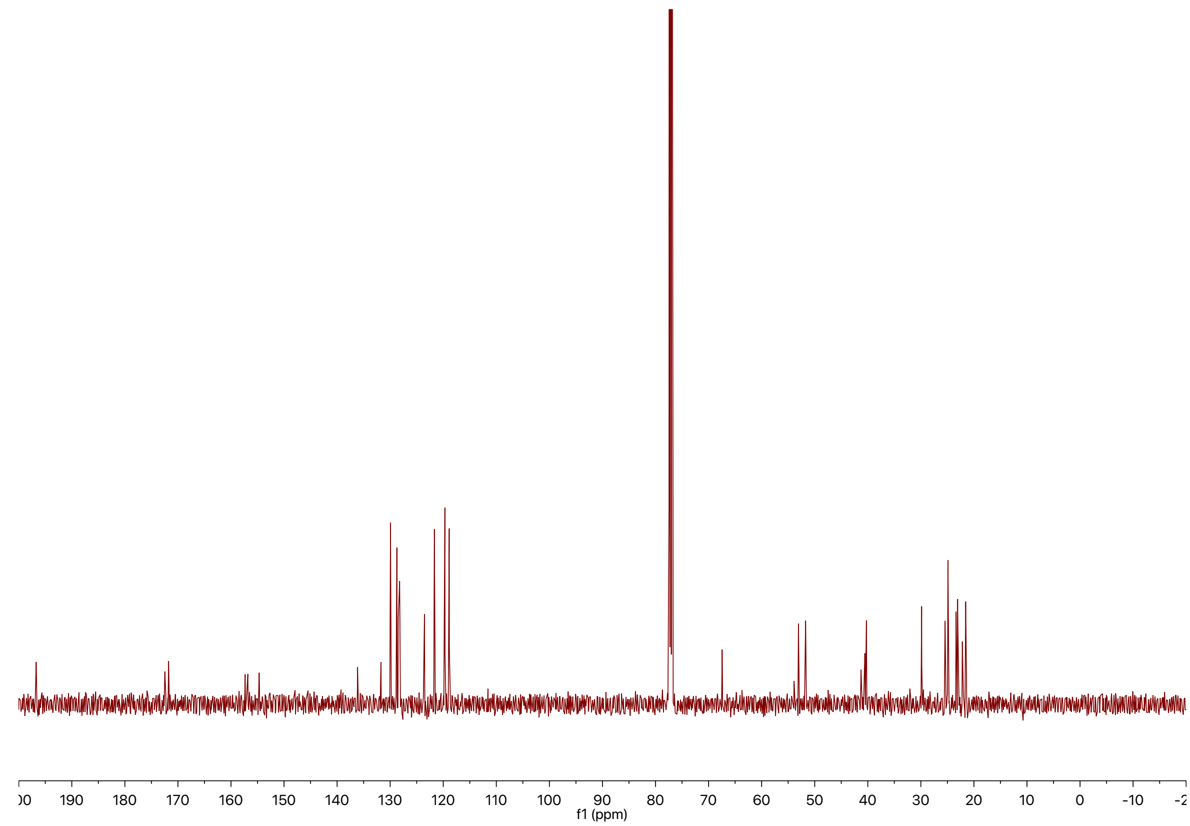

^{13}C-NMR (126 MHz, $CDCl_3$) of compound 10.

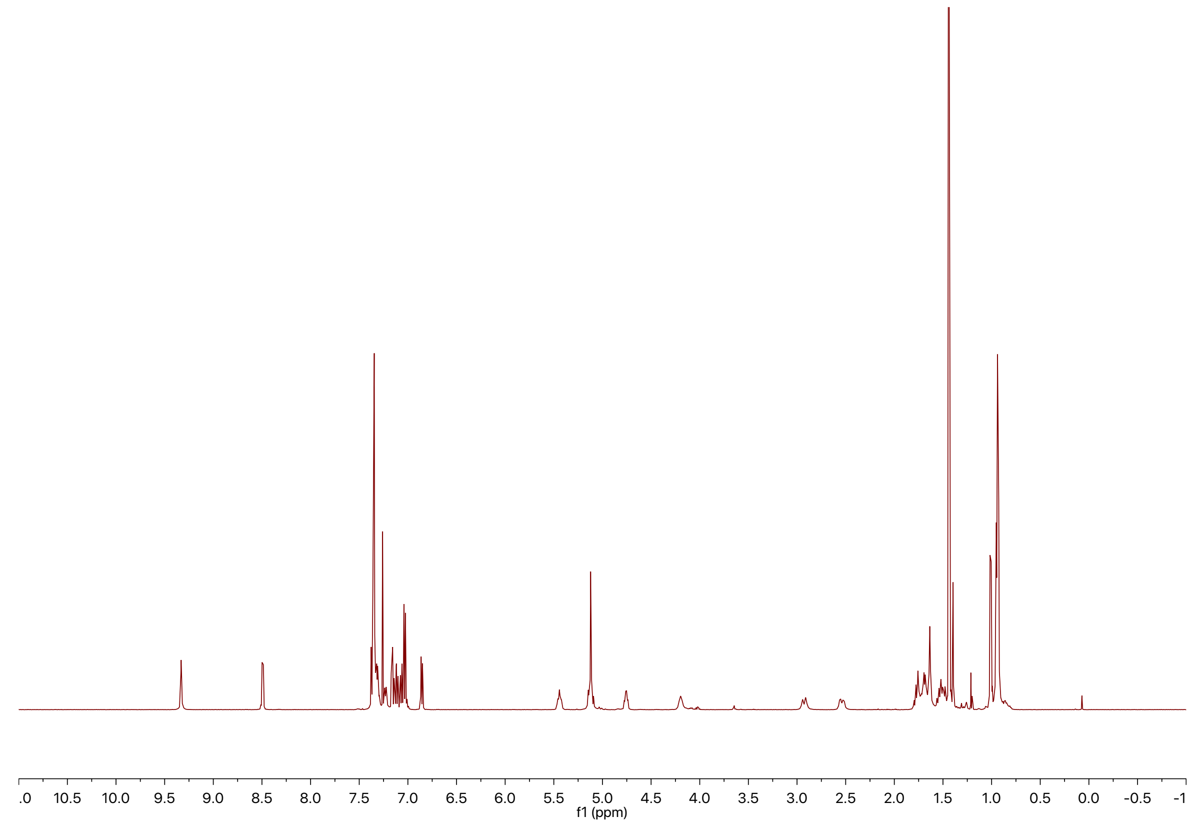

^{1}H-NMR (500 MHz, $CDCl_3$) of compound 18.

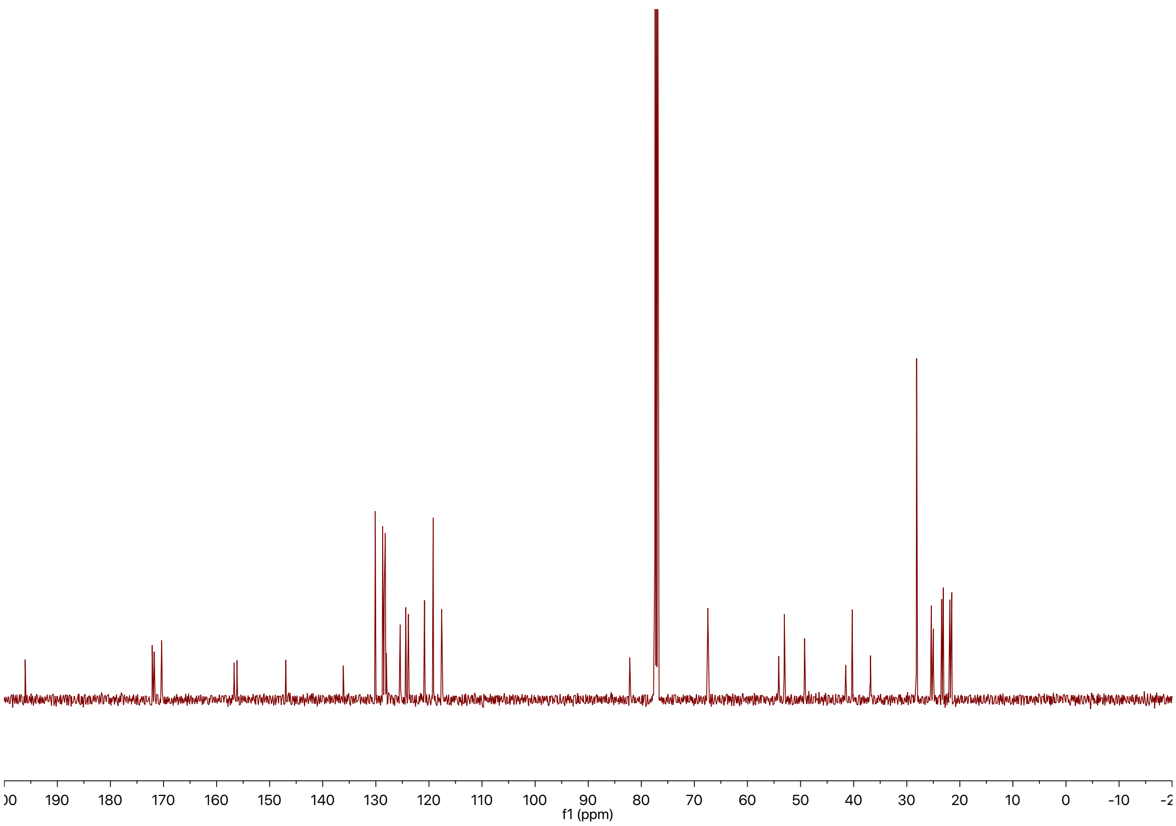

^{13}C-NMR (126 MHz, $CDCl_3$) of compound 18.

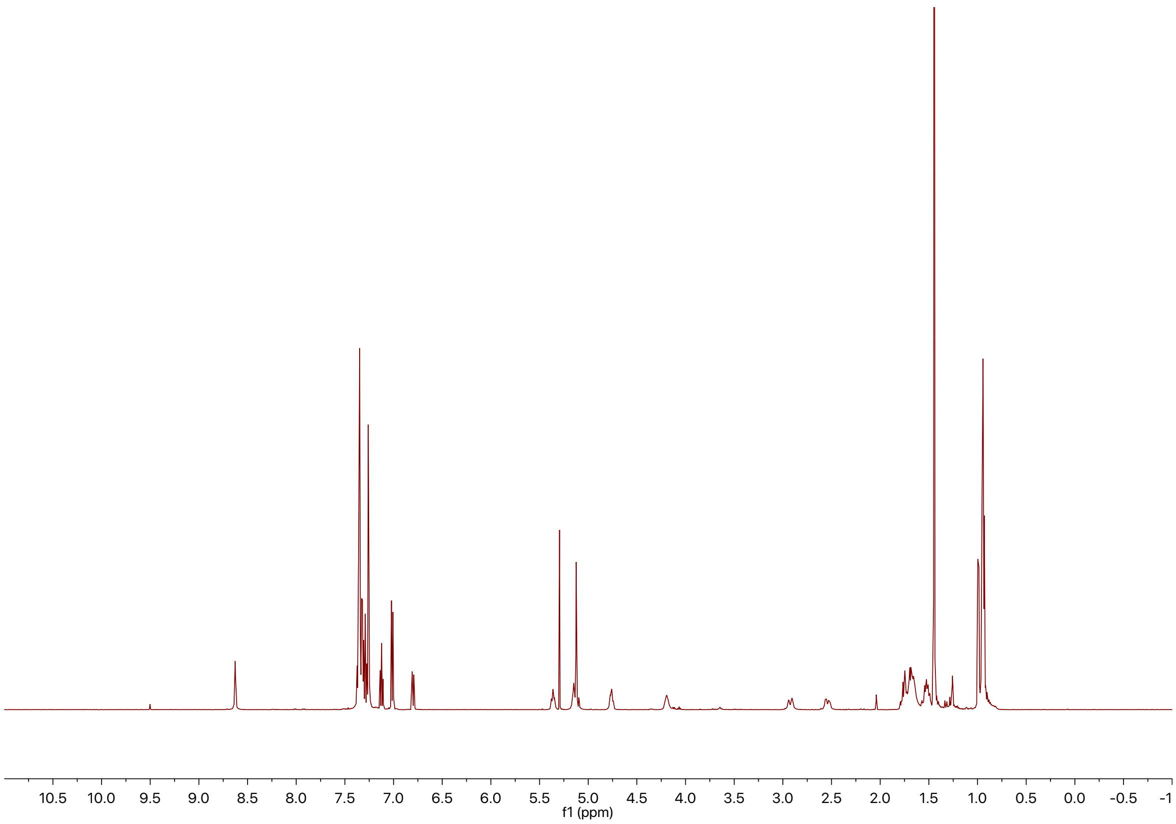

^{1}H-NMR (500 MHz, $CDCl_3$) of compound 19.

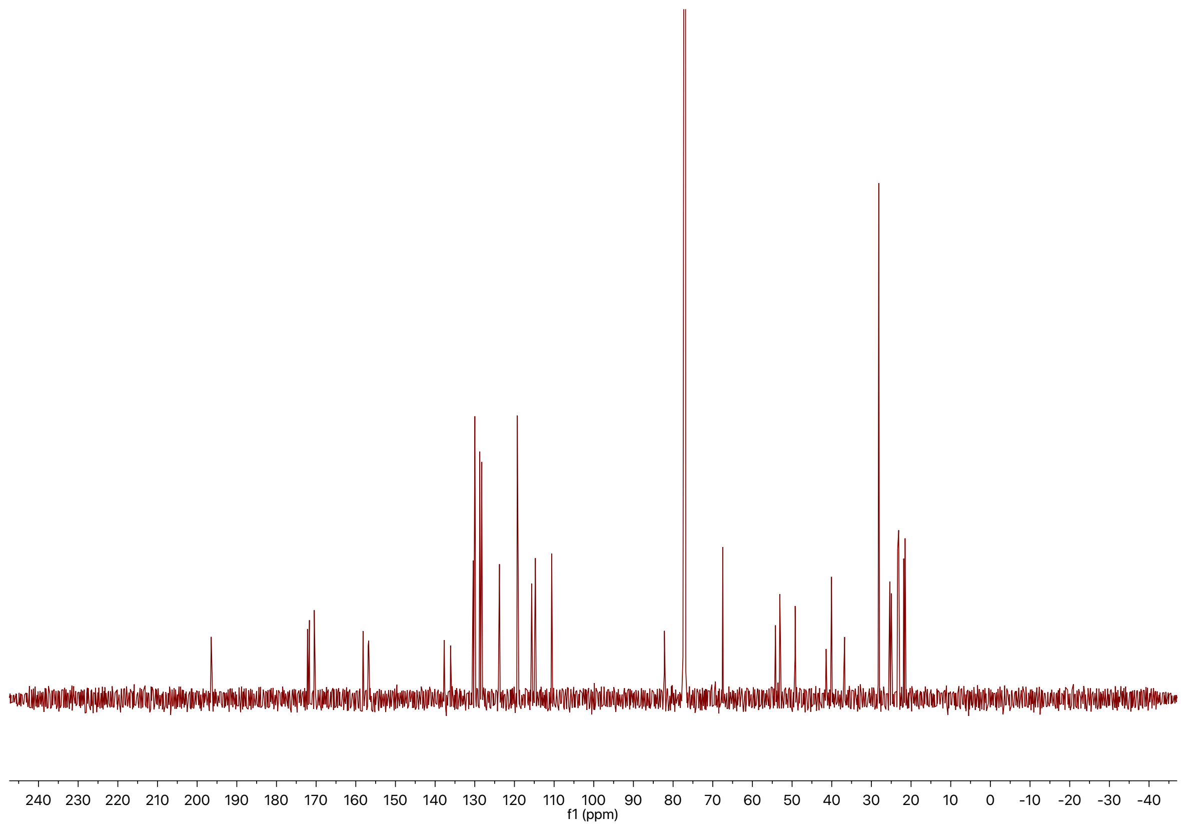

^{13}C-NMR (126 MHz, $CDCl_3$) of compound 19.

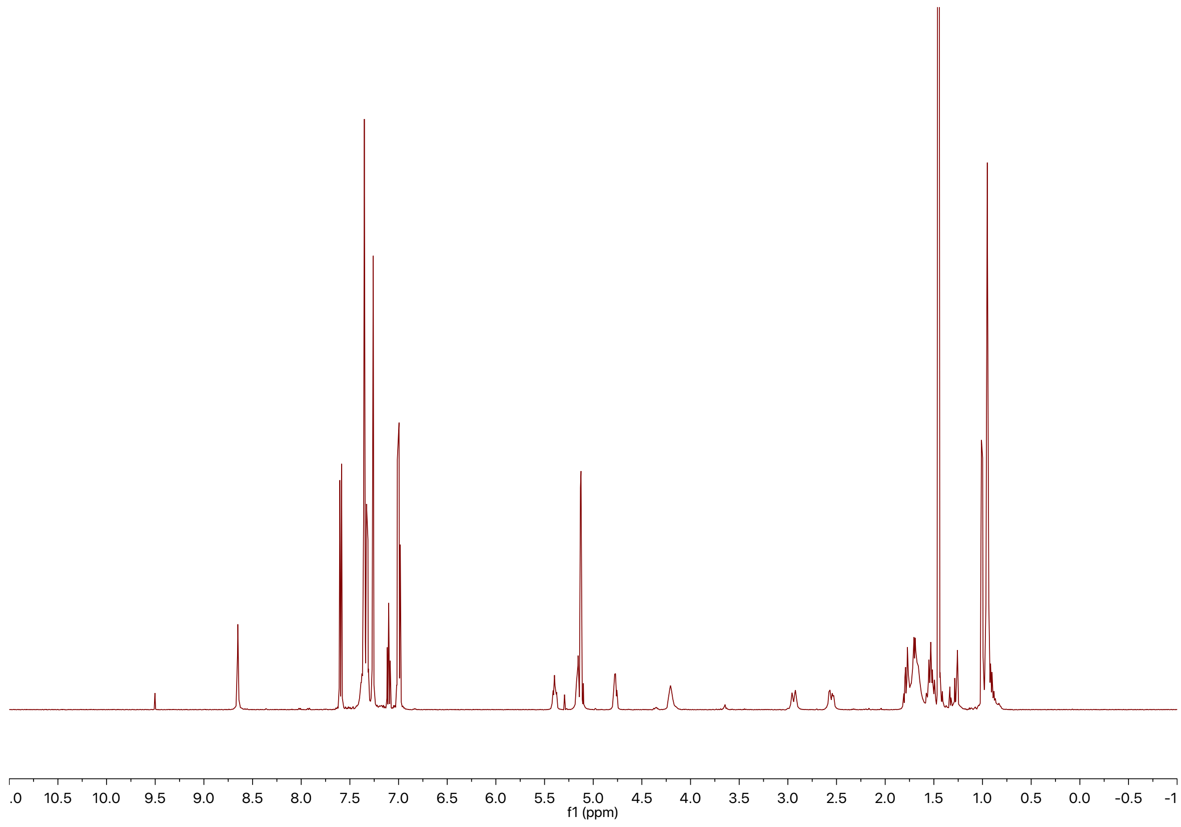

^{1}H-NMR (500 MHz, $CDCl_3$) of compound 20.

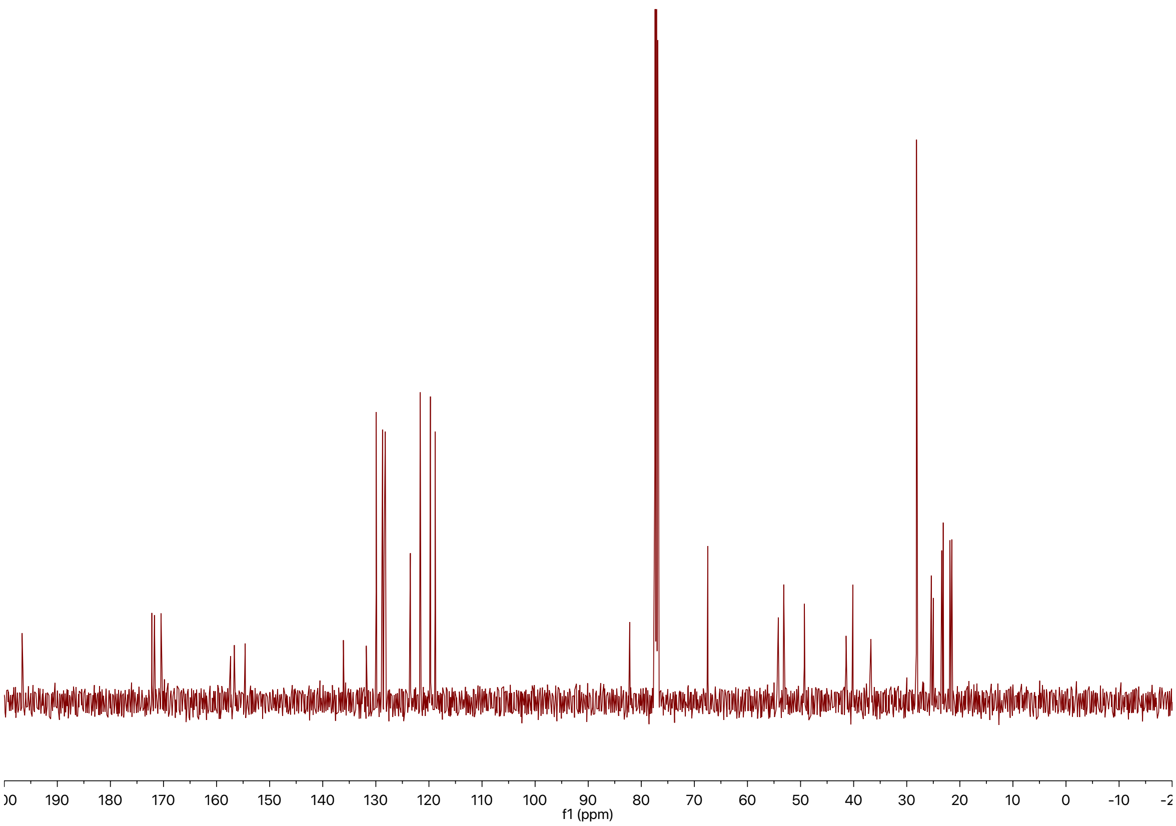

^{13}C-NMR (126 MHz, $CDCl_3$) of compound 20.

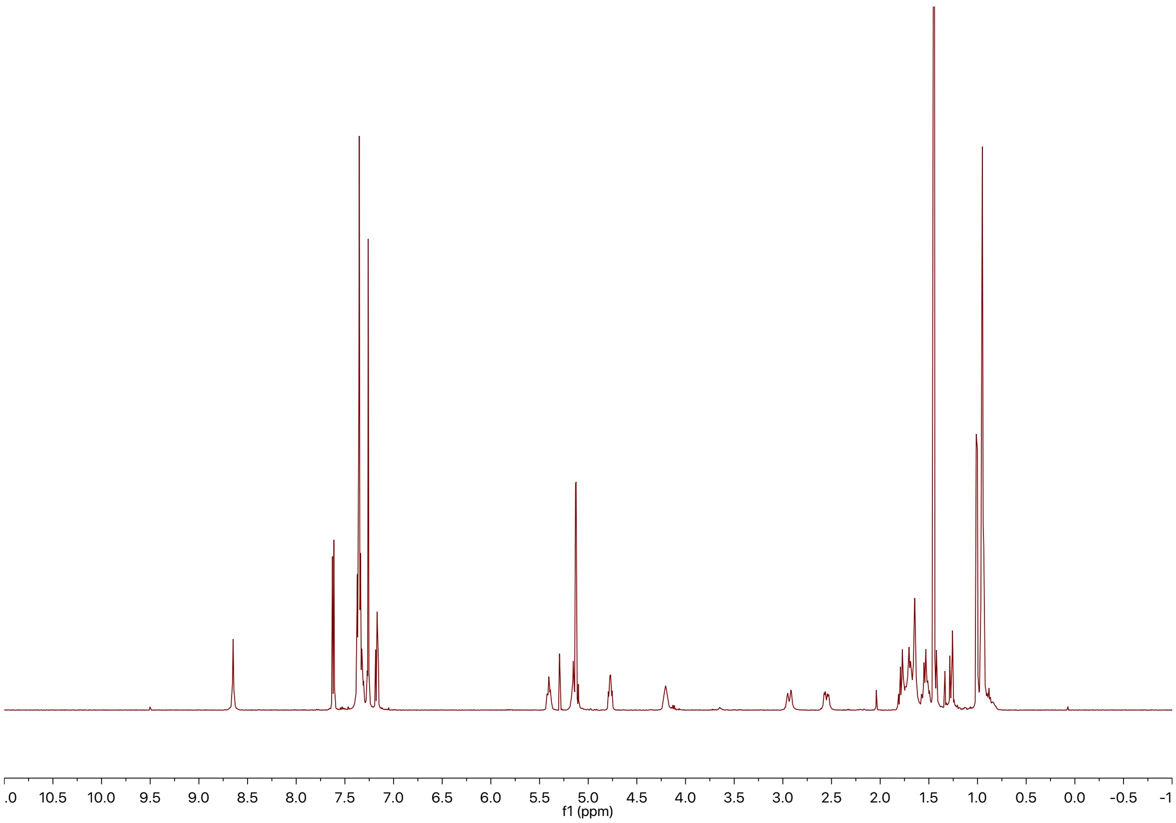

^{1}H-NMR (500 MHz, $CDCl_3$) of compound 16.

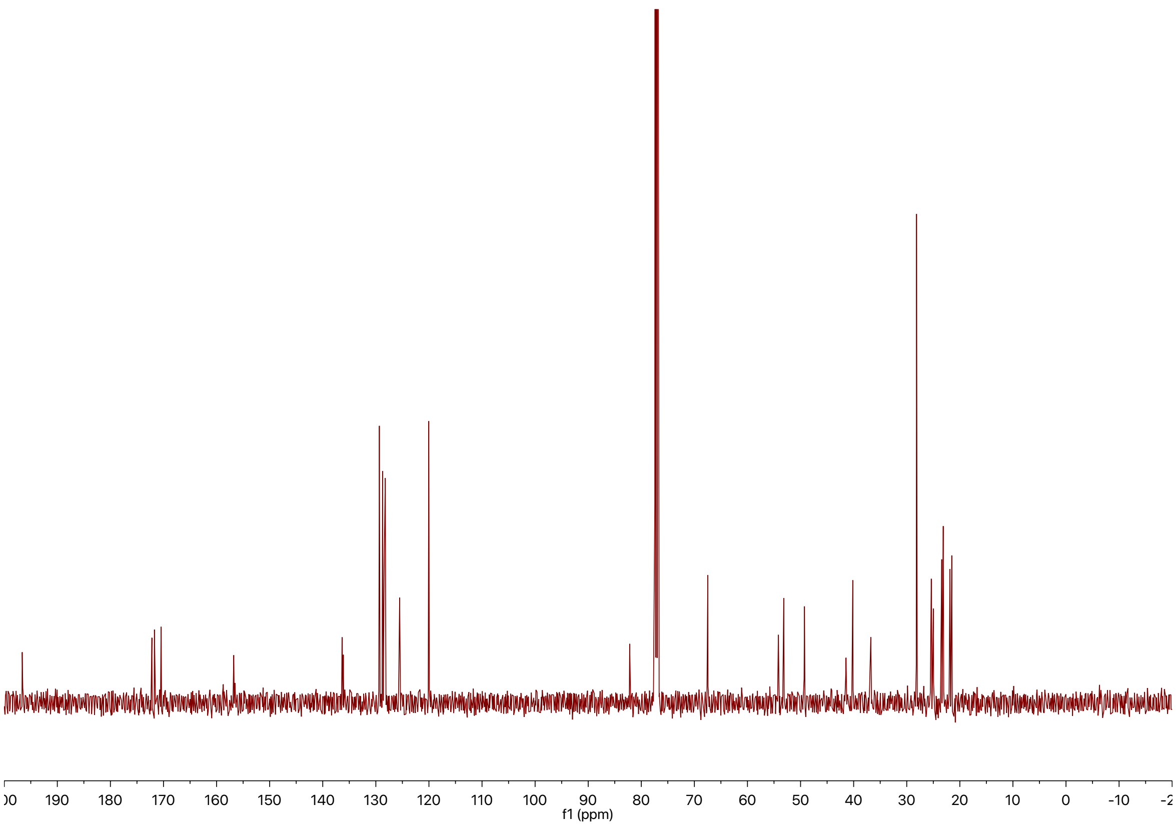

^{13}C-NMR (126 MHz, $CDCl_3$) of compound 16.

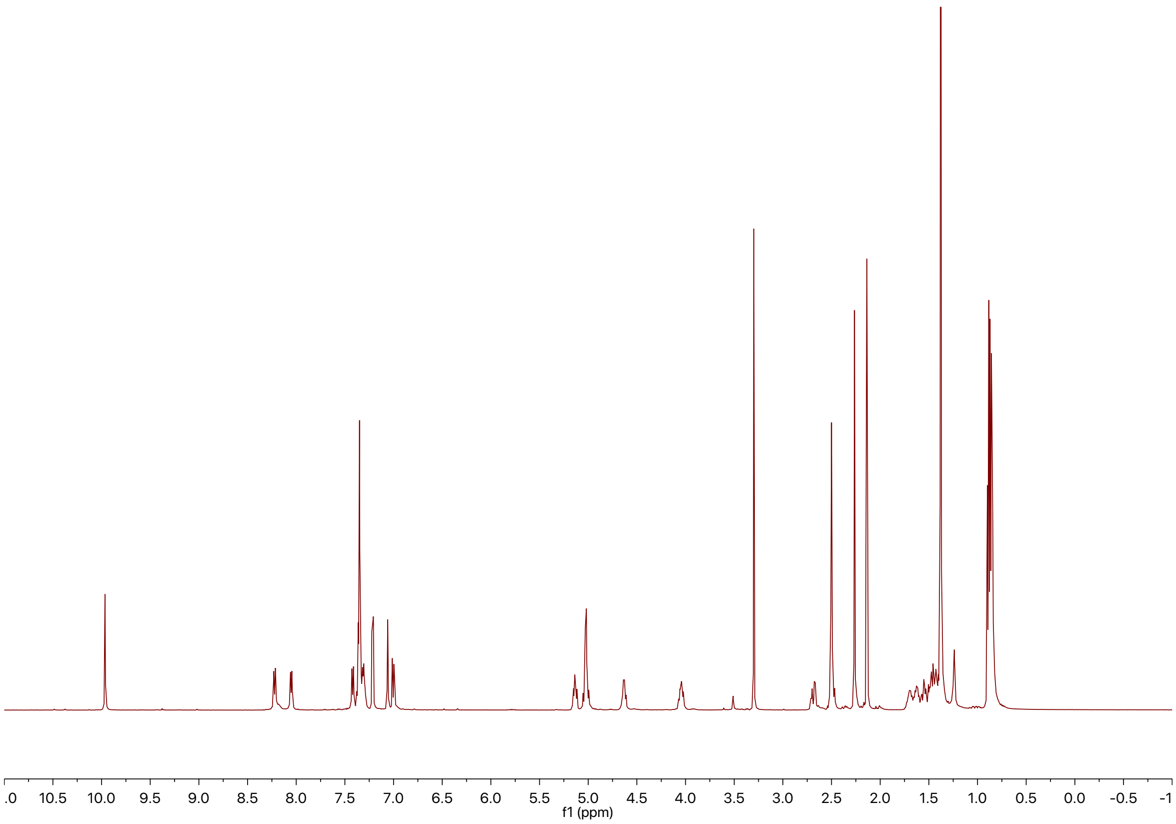

^{1}H-NMR (500 MHz, DMSO-d_6) of compound 17.

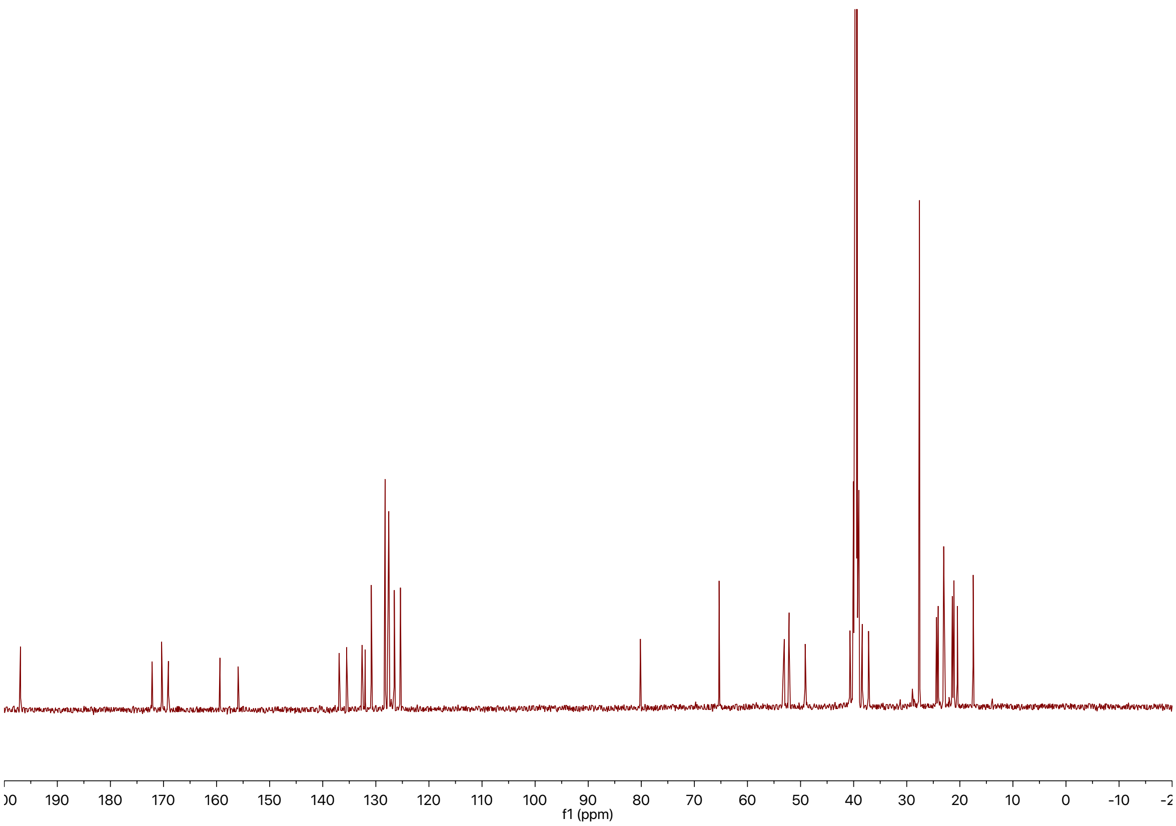

^{13}C-NMR (126 MHz, DMSO-d$_6$) of compound 17.

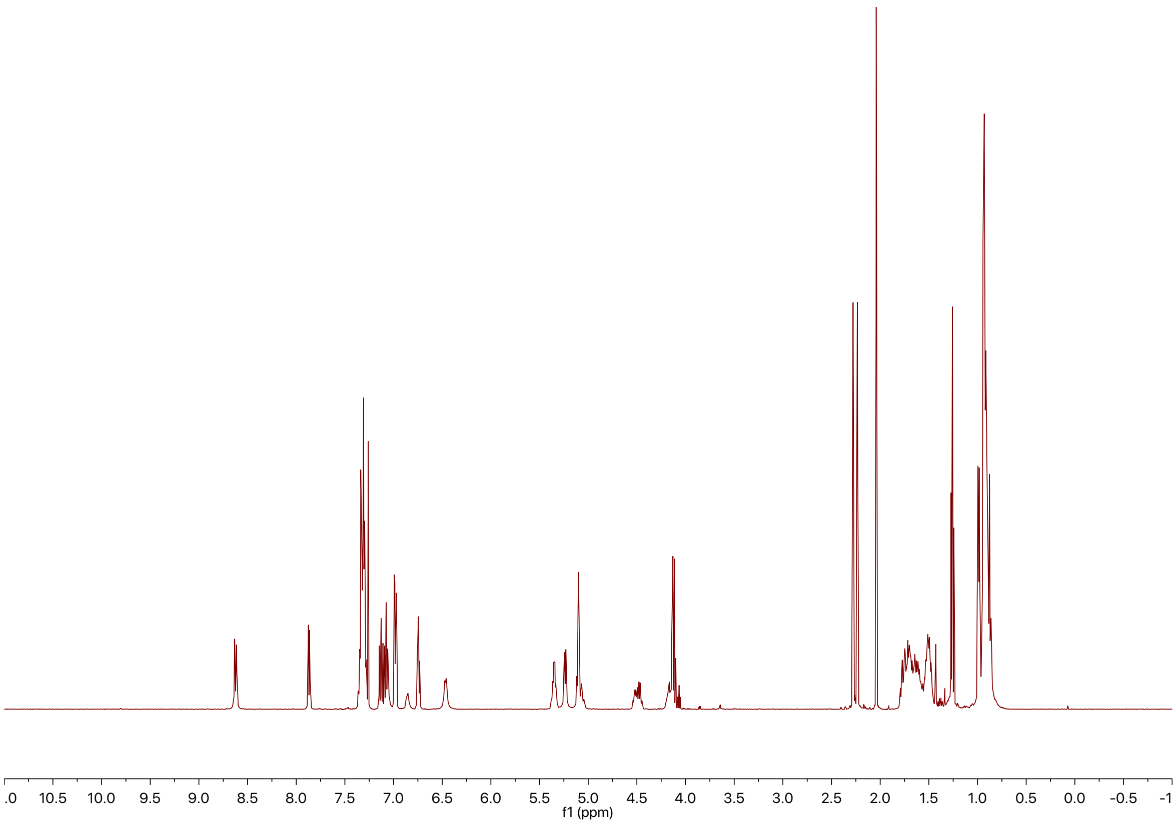

^{1}H-NMR (500 MHz, CDCl$_3$) of compound 15.

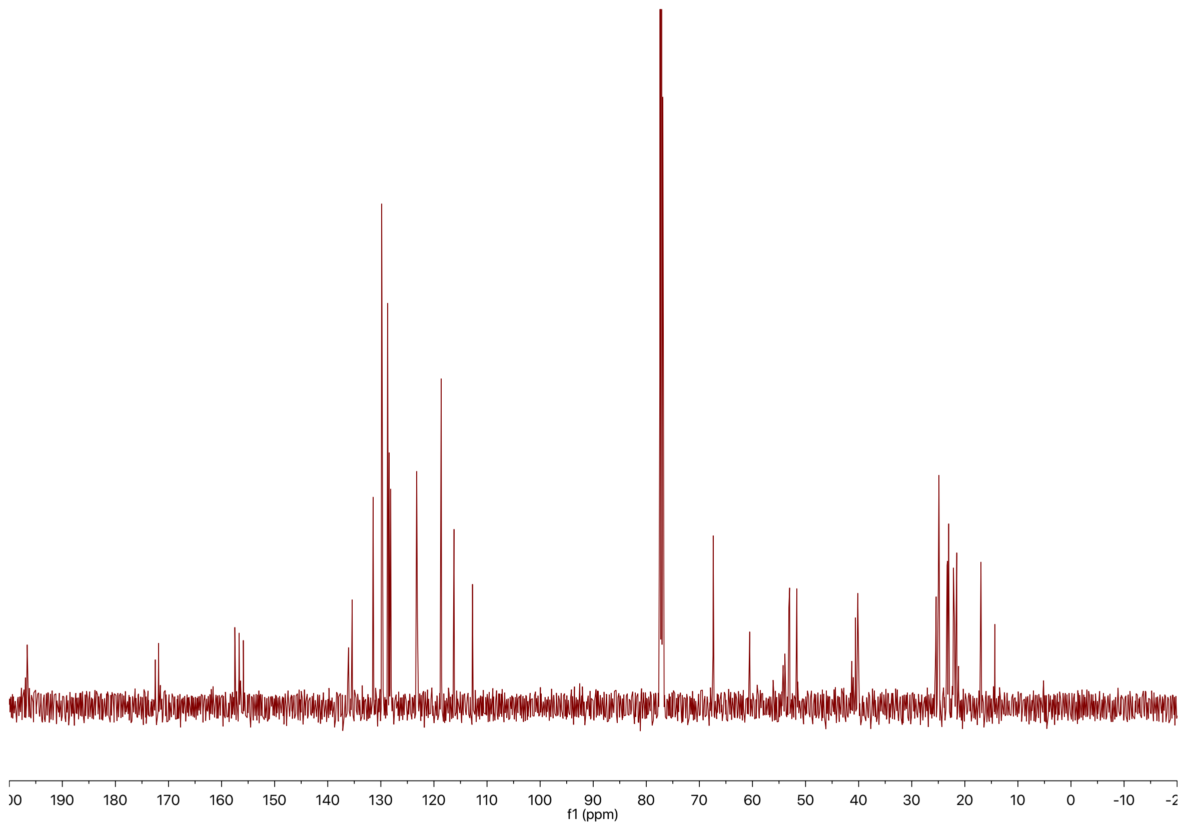

^{13}C-NMR (126 MHz, CDCl$_3$) of compound 15.

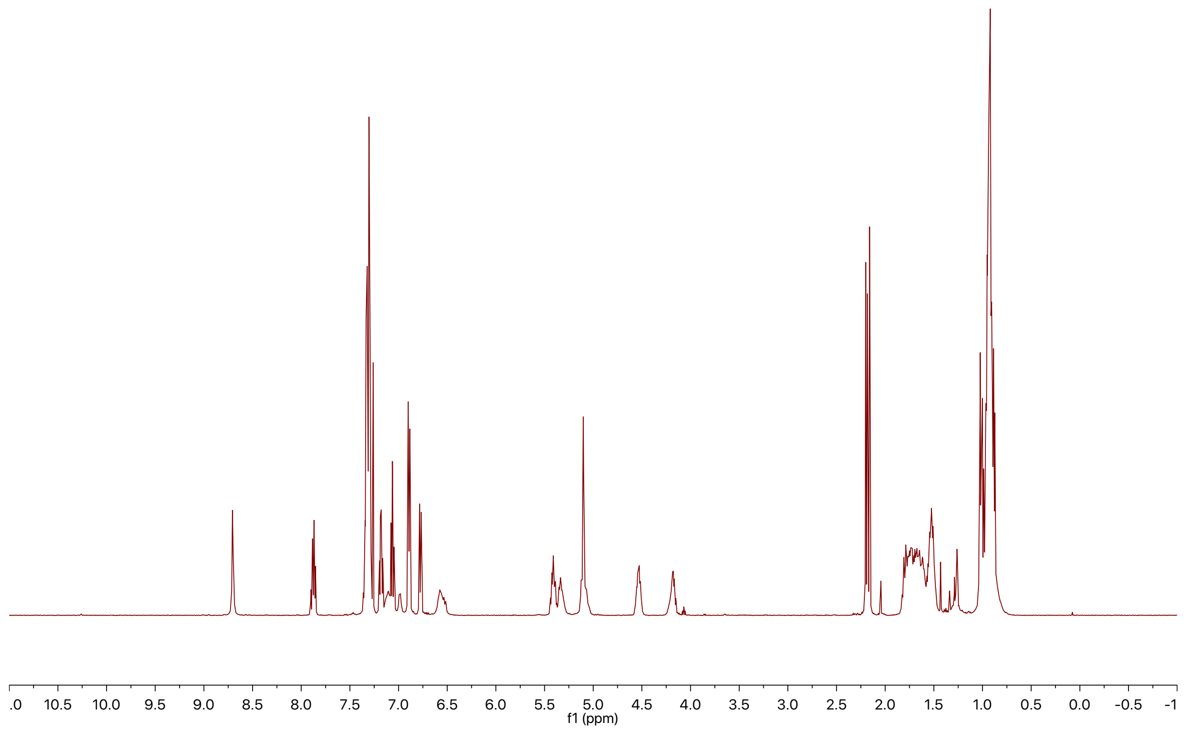

^{1}H-NMR (500 MHz, CDCl$_3$) of compound 14.

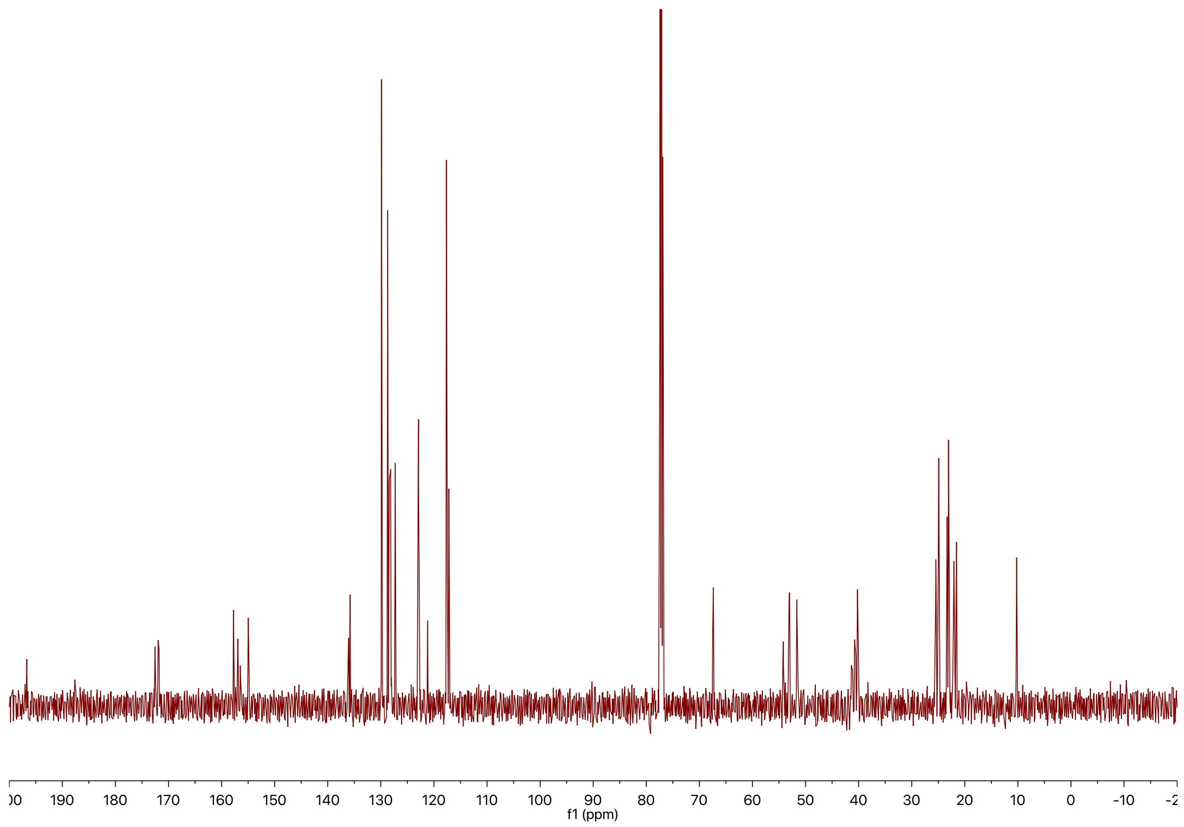

^{13}C-NMR (126 MHz, $CDCl_3$) of compound 14.

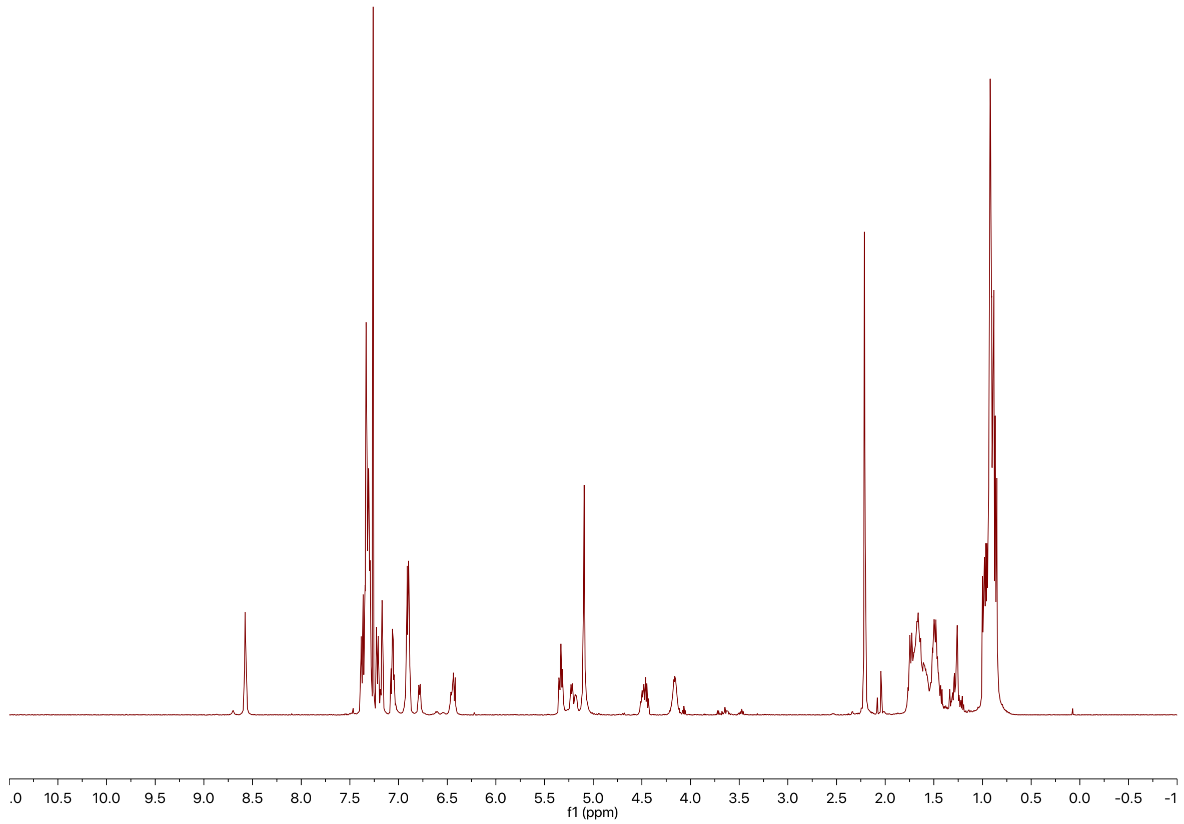

^{1}H-NMR (500 MHz, $CDCl_3$) of compound 13.

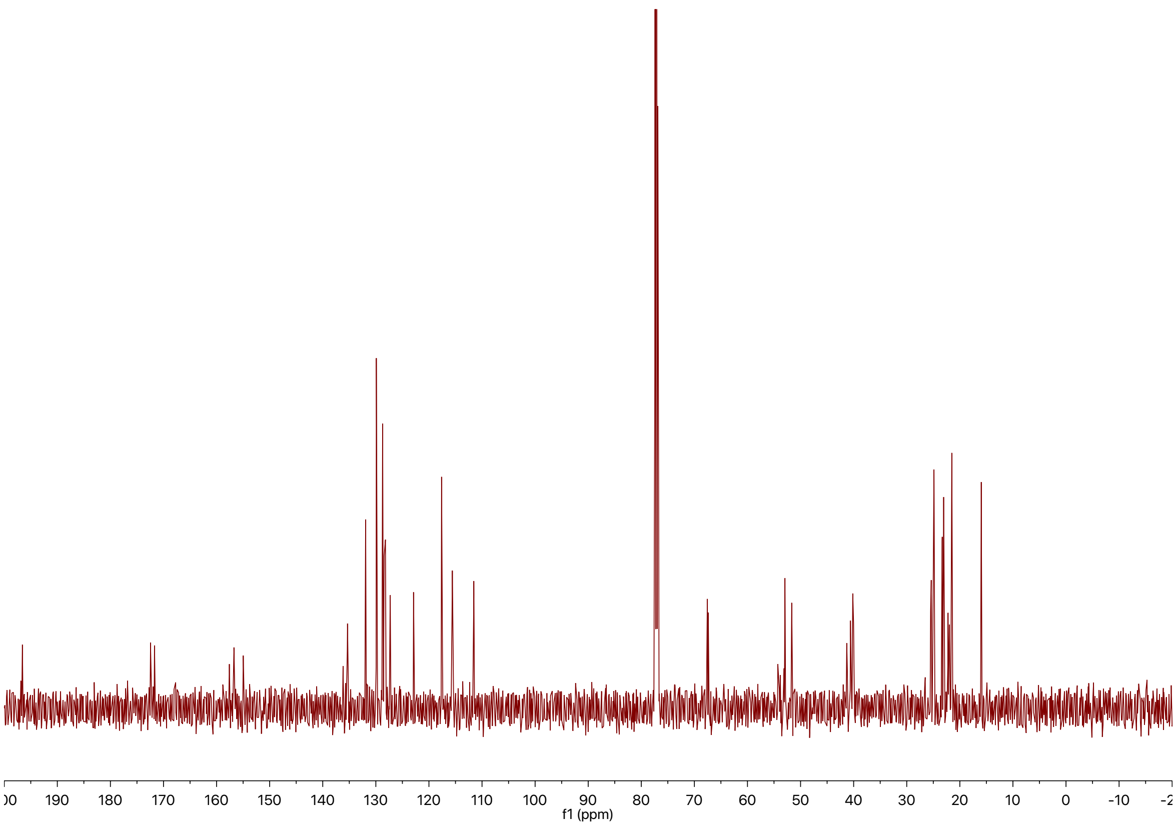

^{13}C-NMR (126 MHz, CDCl$_3$) of compound 13.

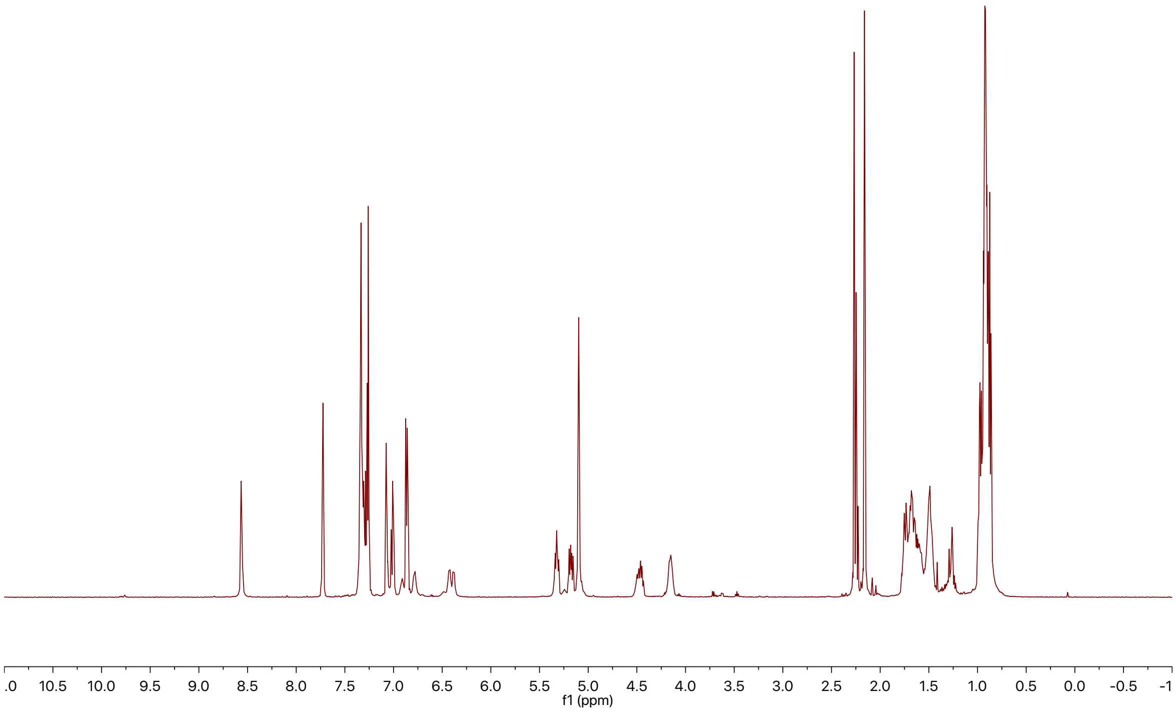

^{1}H-NMR (500 MHz, CDCl$_3$) of compound 11.

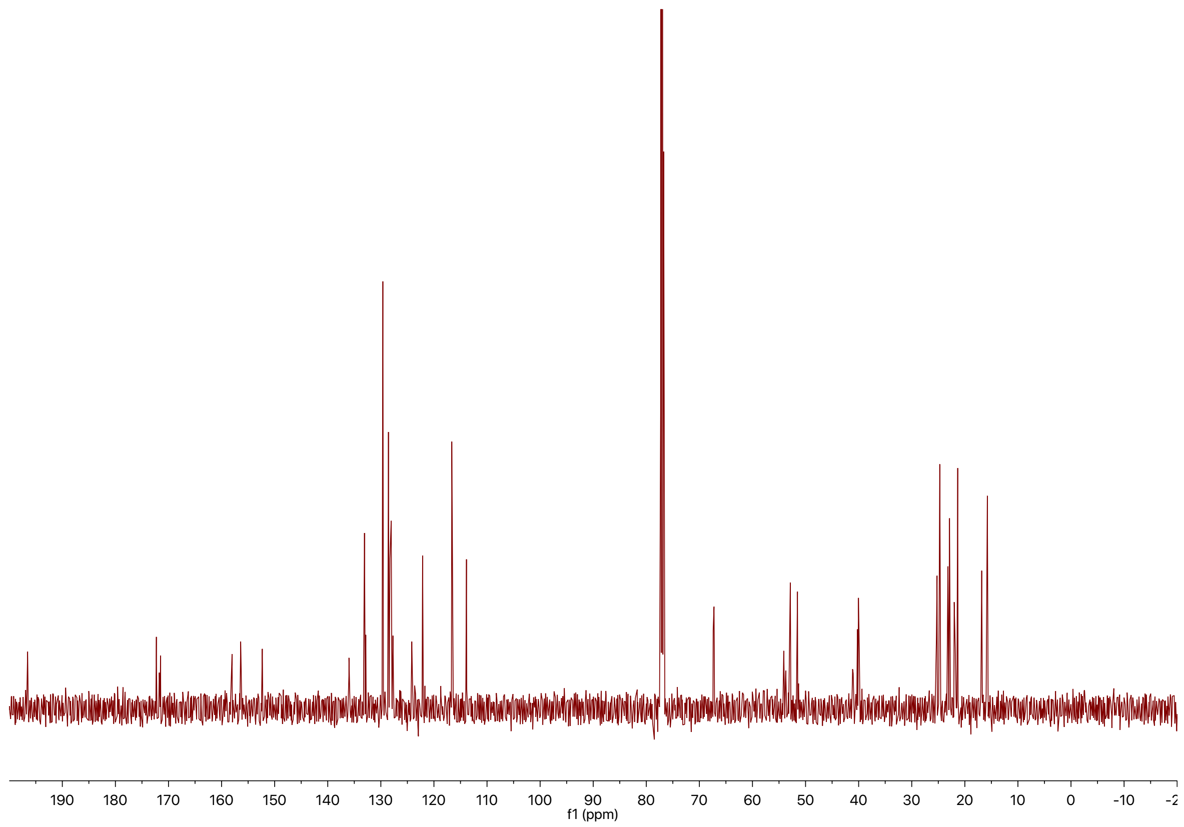

^{13}C-NMR (126 MHz, $CDCl_3$) of compound 11.

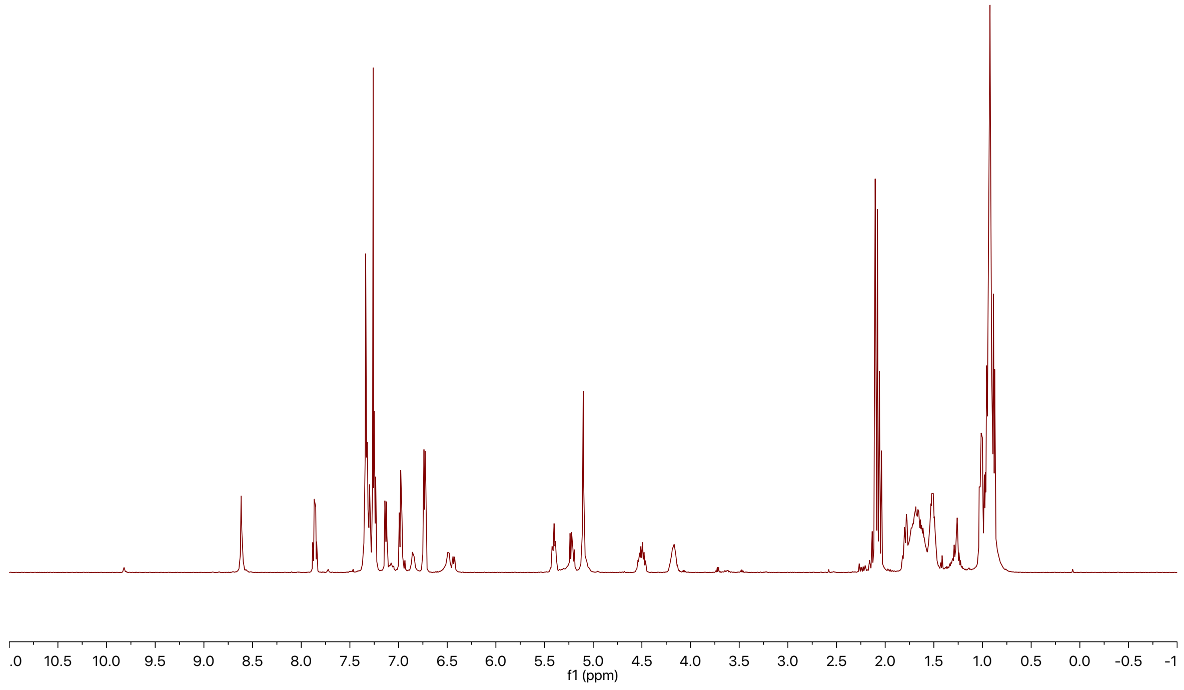

^{1}H-NMR (500 MHz, $CDCl_3$) of compound 12.

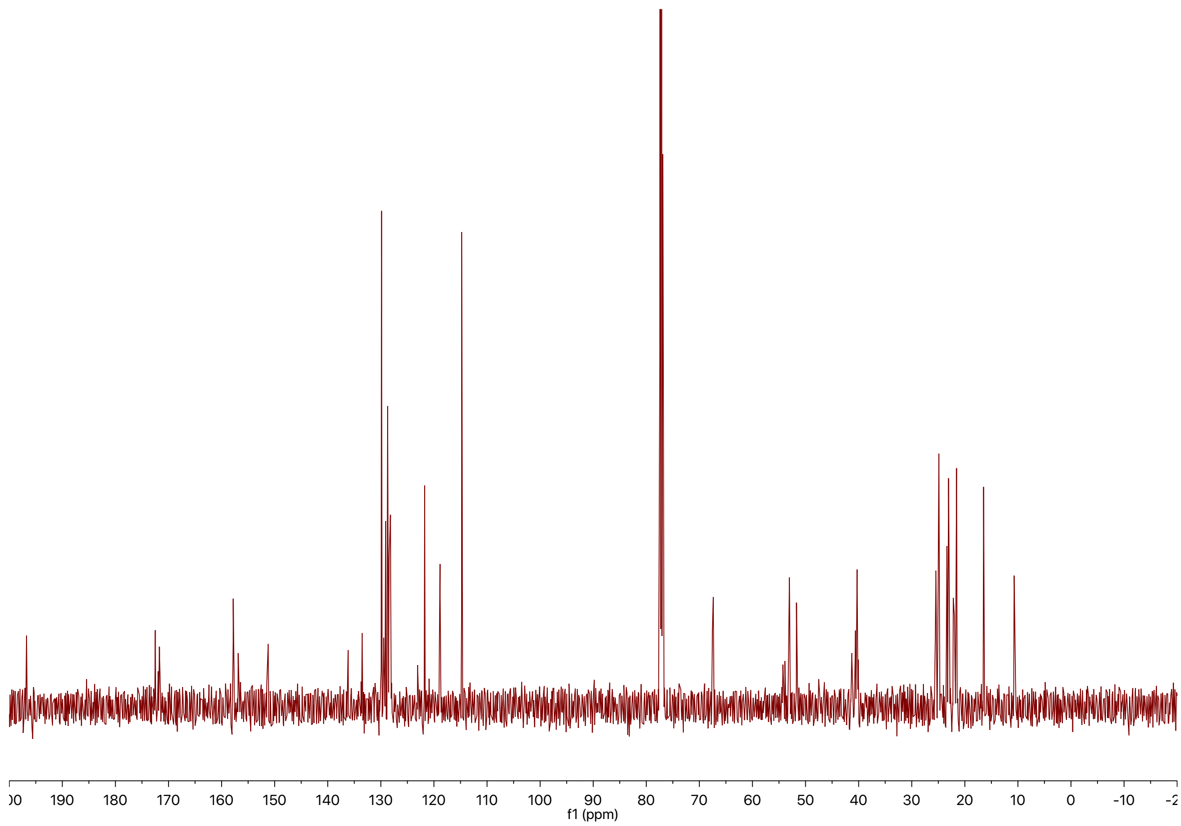

^{13}C-NMR (126 MHz, CDCl₃) of compound 12.

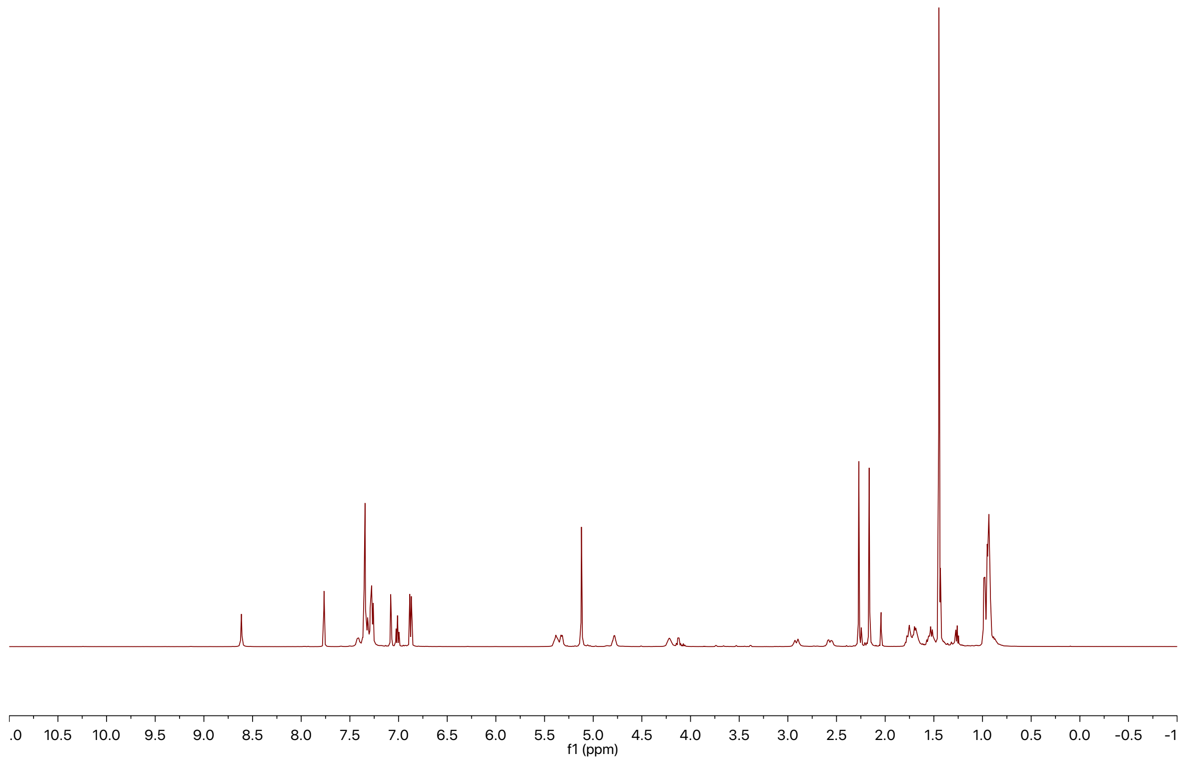

^{1}H-NMR (500 MHz, CDCl₃) of compound 21.

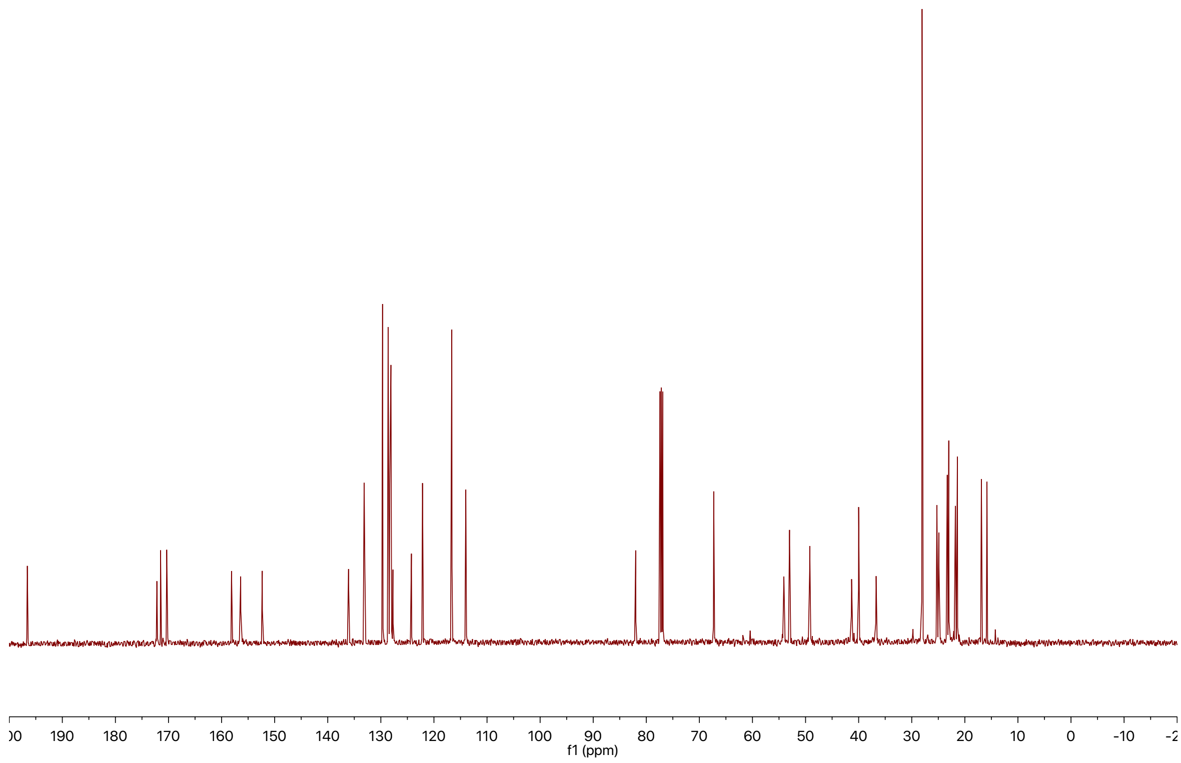

^{13}C-NMR (126 MHz, CDCl₃) of compound 21.

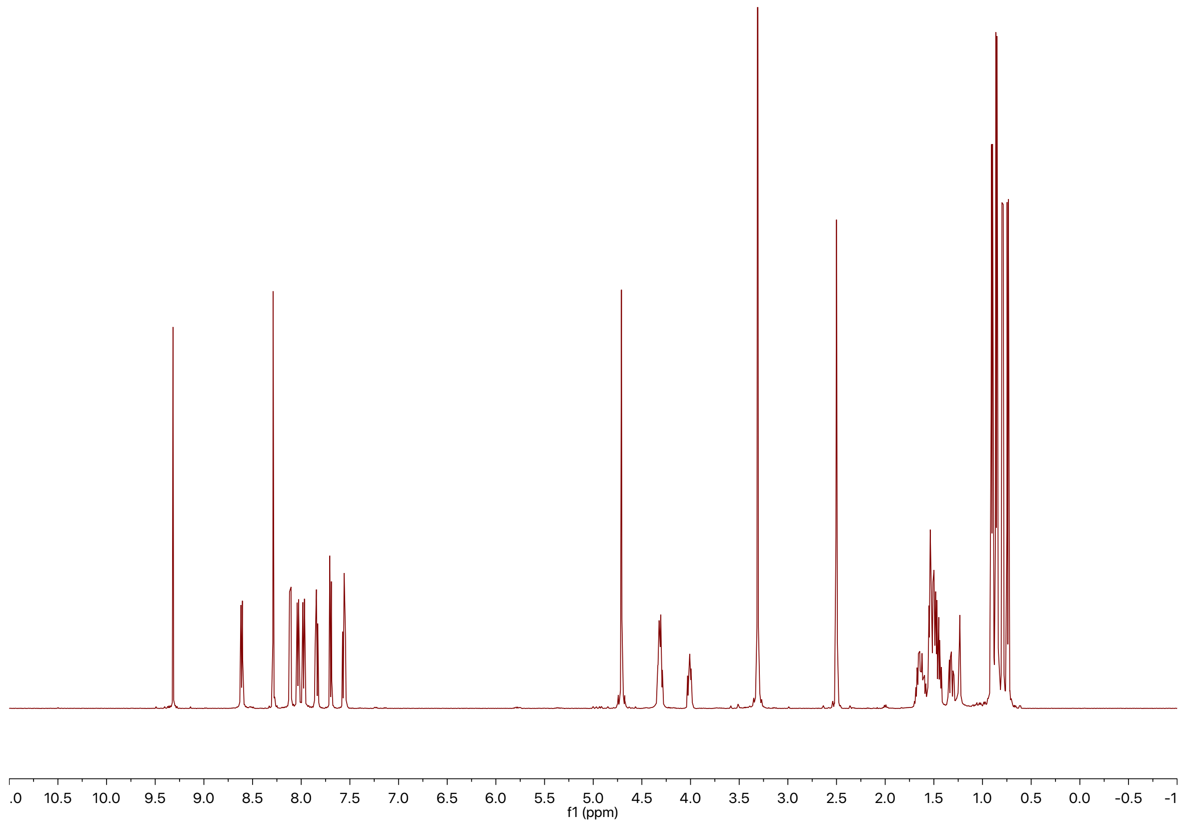

^{1}H-NMR (500 MHz, DMSO-d₆) of compound 22.

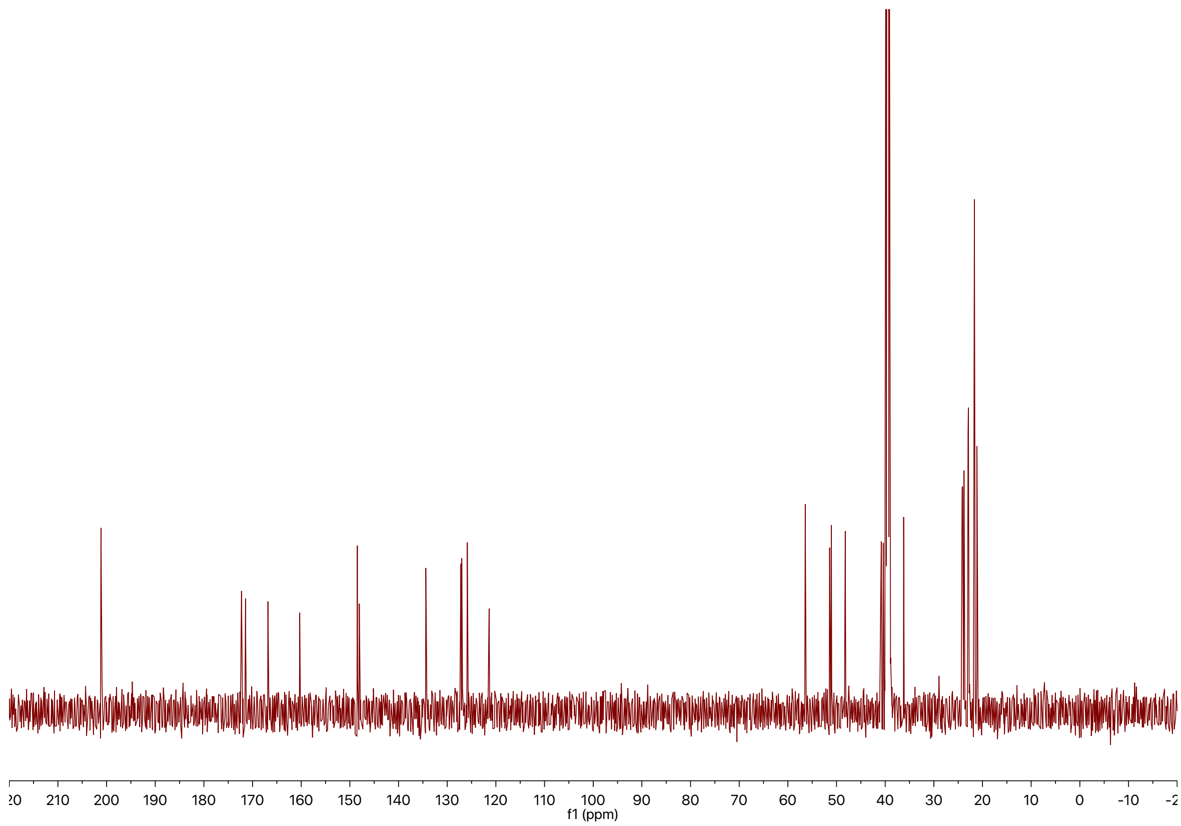

^{13}C-NMR (126 MHz, DMSO-d$_{6]}$) of compound 22.

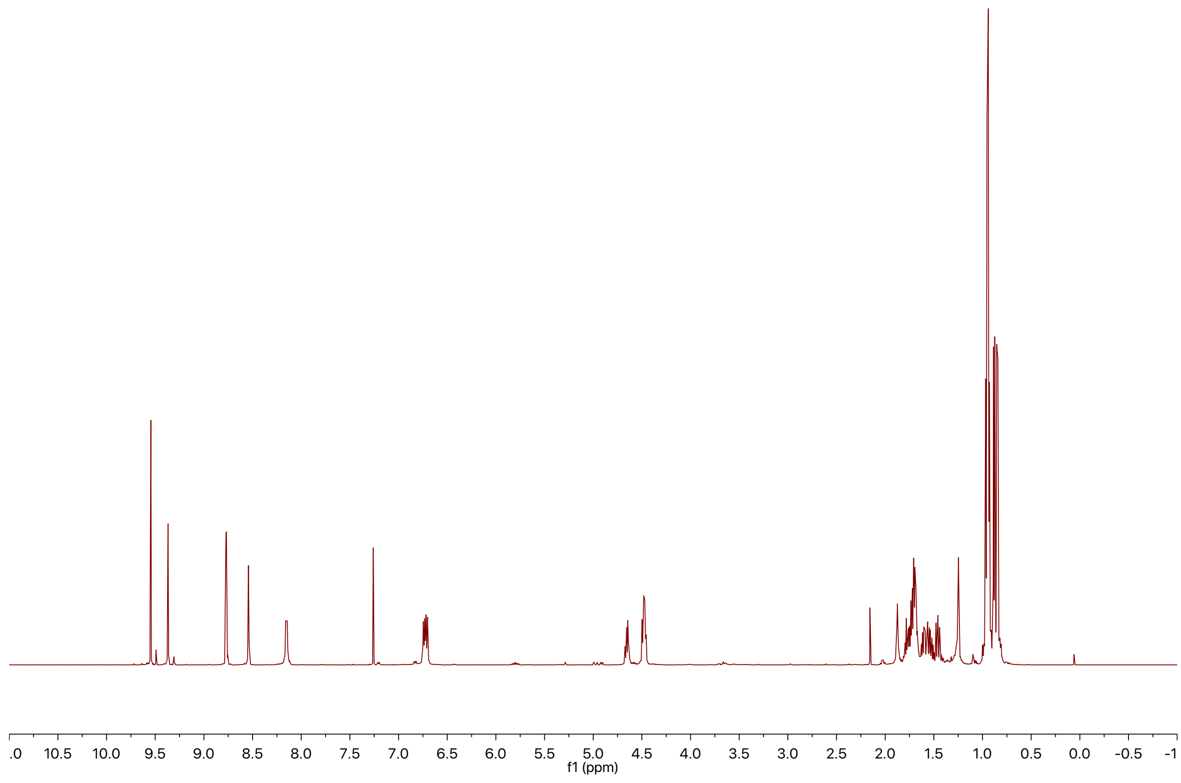

^{1}H-NMR (500 MHz, CDCl$_3$) of compound 23.

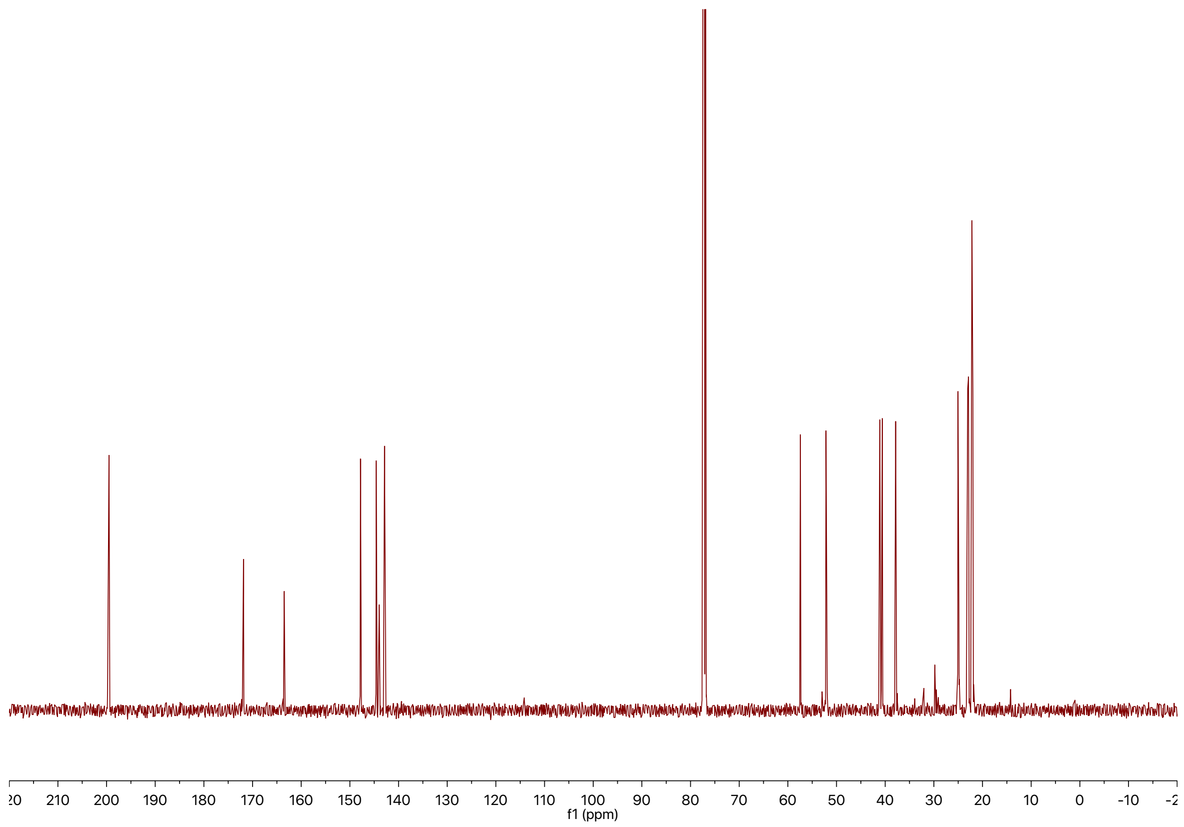

^{13}C-NMR (126 MHz, $CDCl_3$) of compound 23.

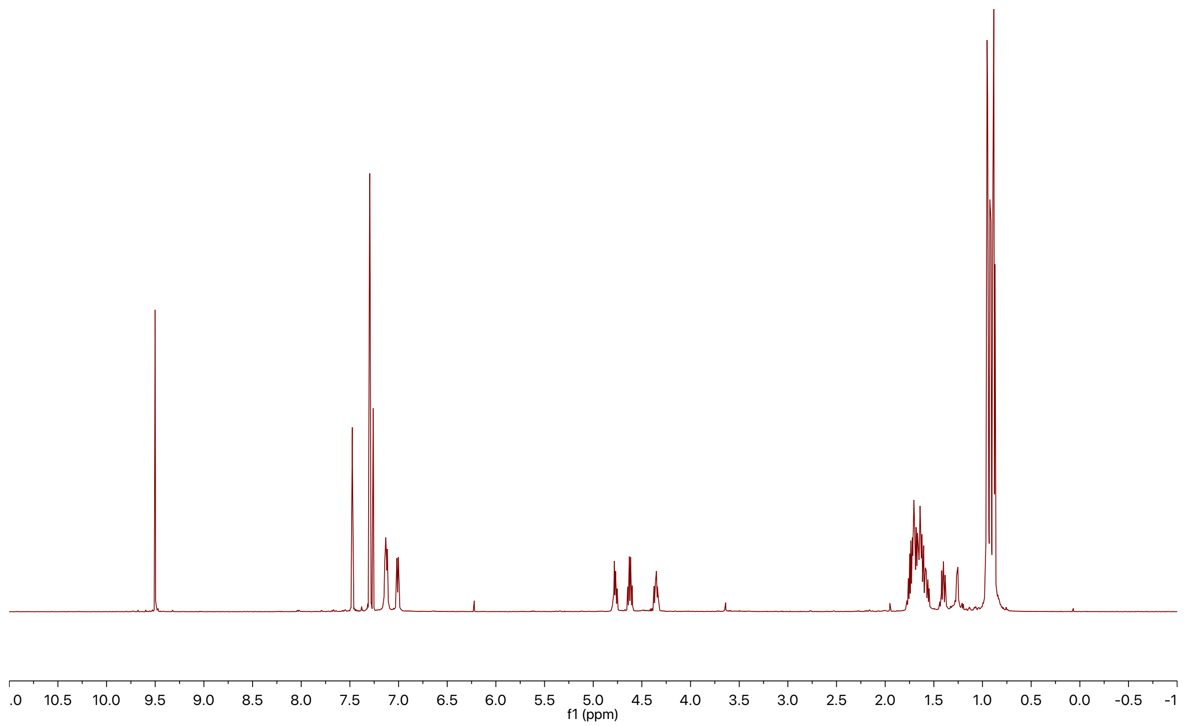

^{1}H-NMR (500 MHz, $CDCl_3$) of compound 24.

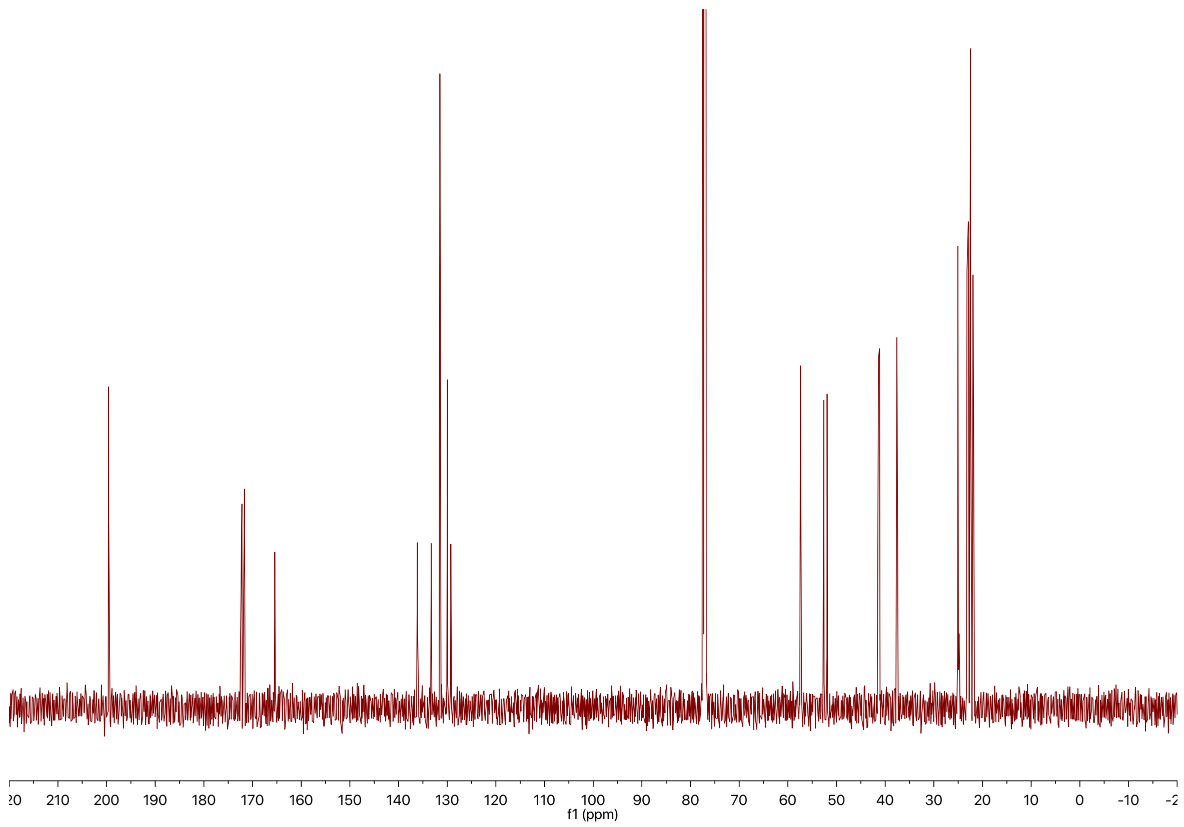

^{13}C-NMR (126 MHz, $CDCl_3$) of compound 24.

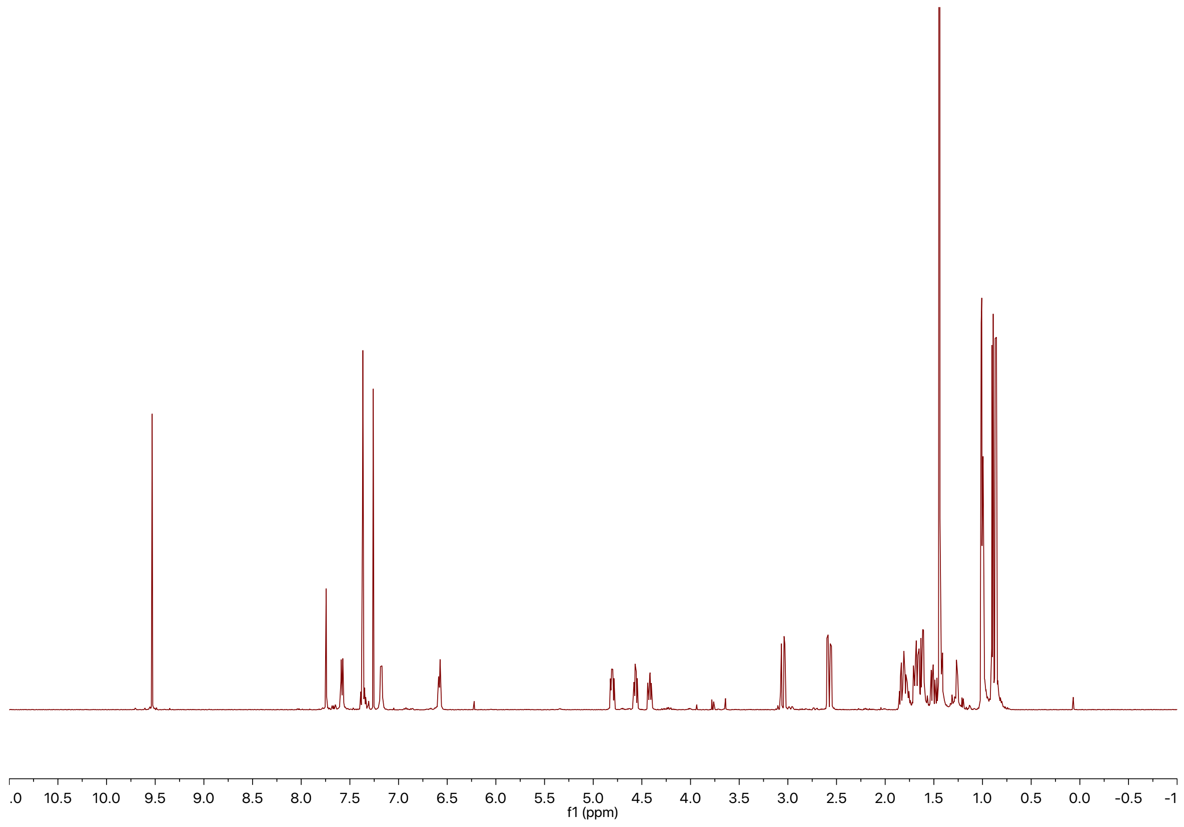

^{1}H-NMR (500 MHz, $CDCl_3$) of compound 25.

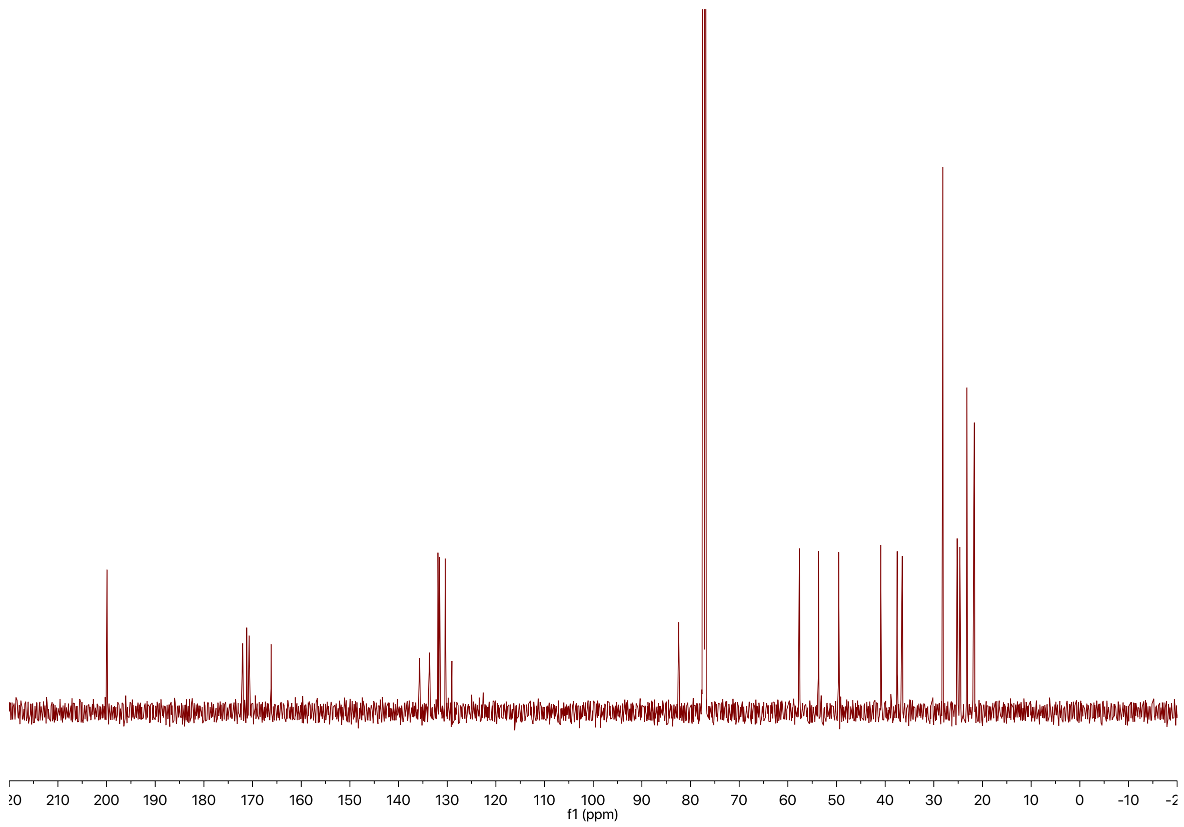

^{13}C-NMR (126 MHz, $CDCl_3$) of compound 25.

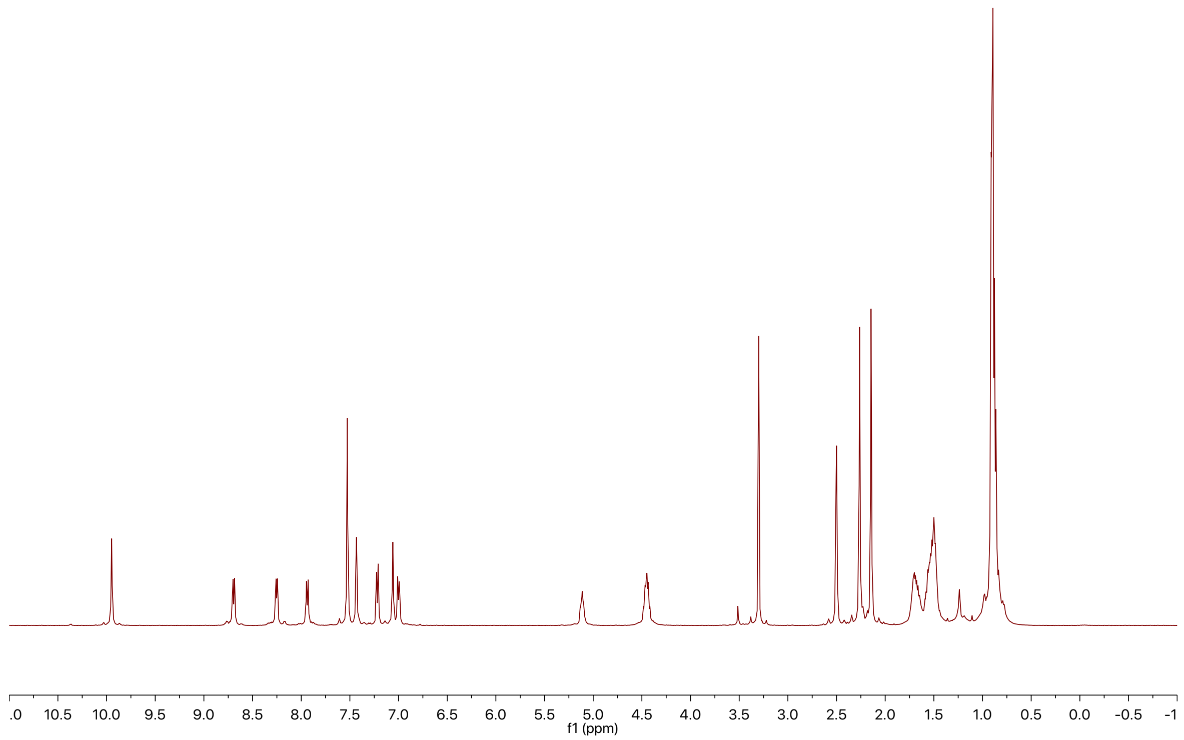

^{1}H-NMR (500 MHz, DMSO-d_6) of compound 26.

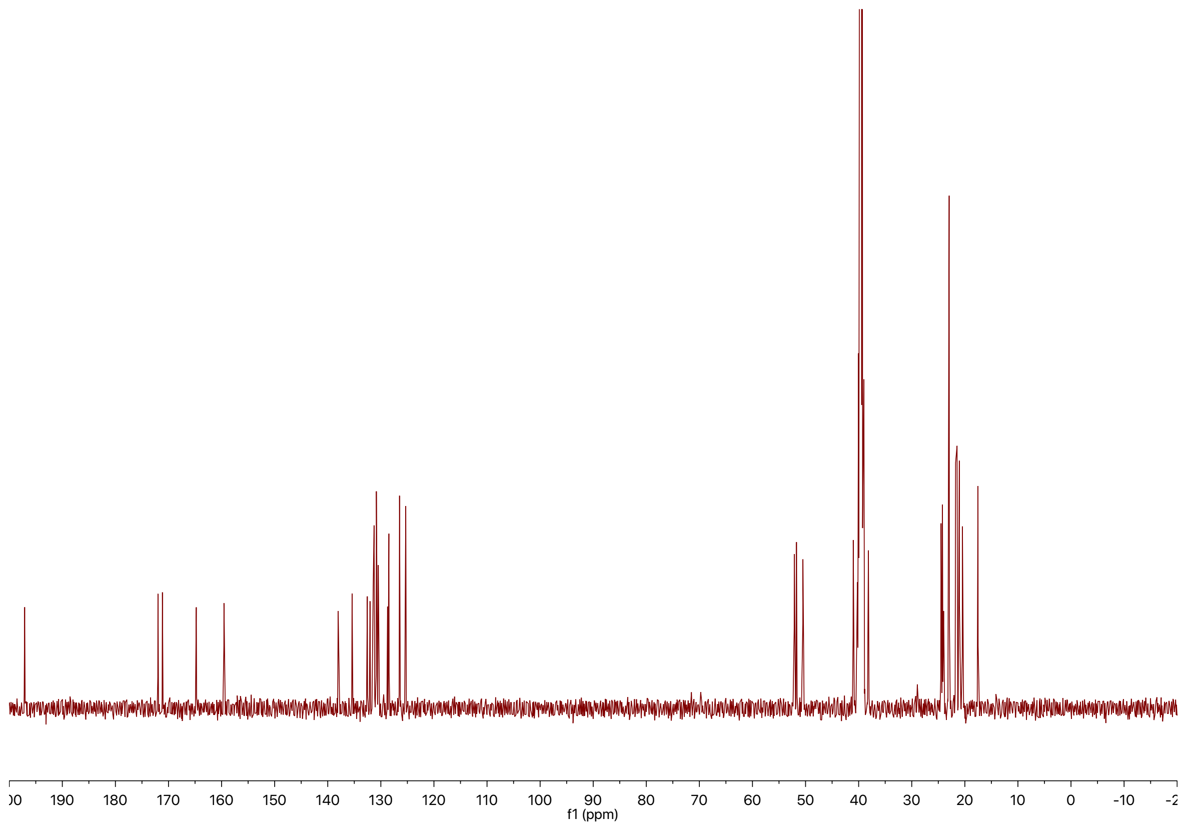

^{13}C-NMR (126 MHz, DMSO-d_6) of compound 26.

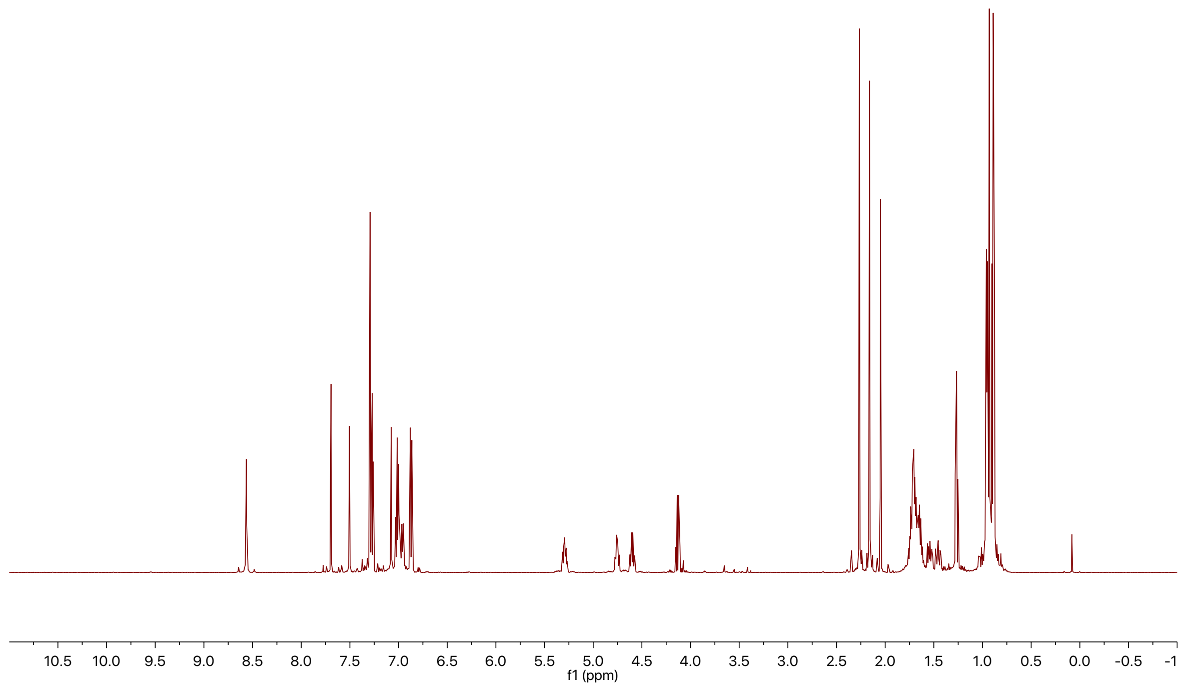

^{1}H-NMR (500 MHz, $CDCl_3$) of compound 27.

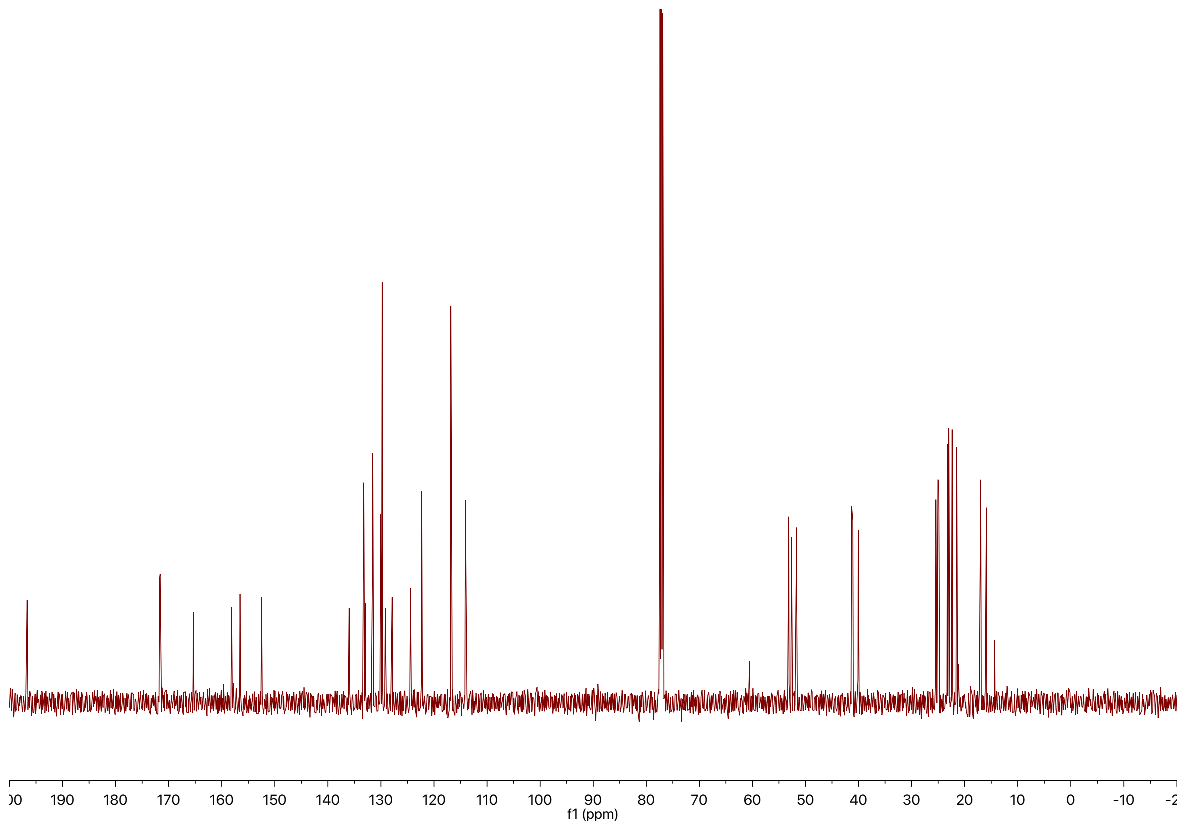

^{13}C-NMR (126 MHz, $CDCl_3$) of compound 27.

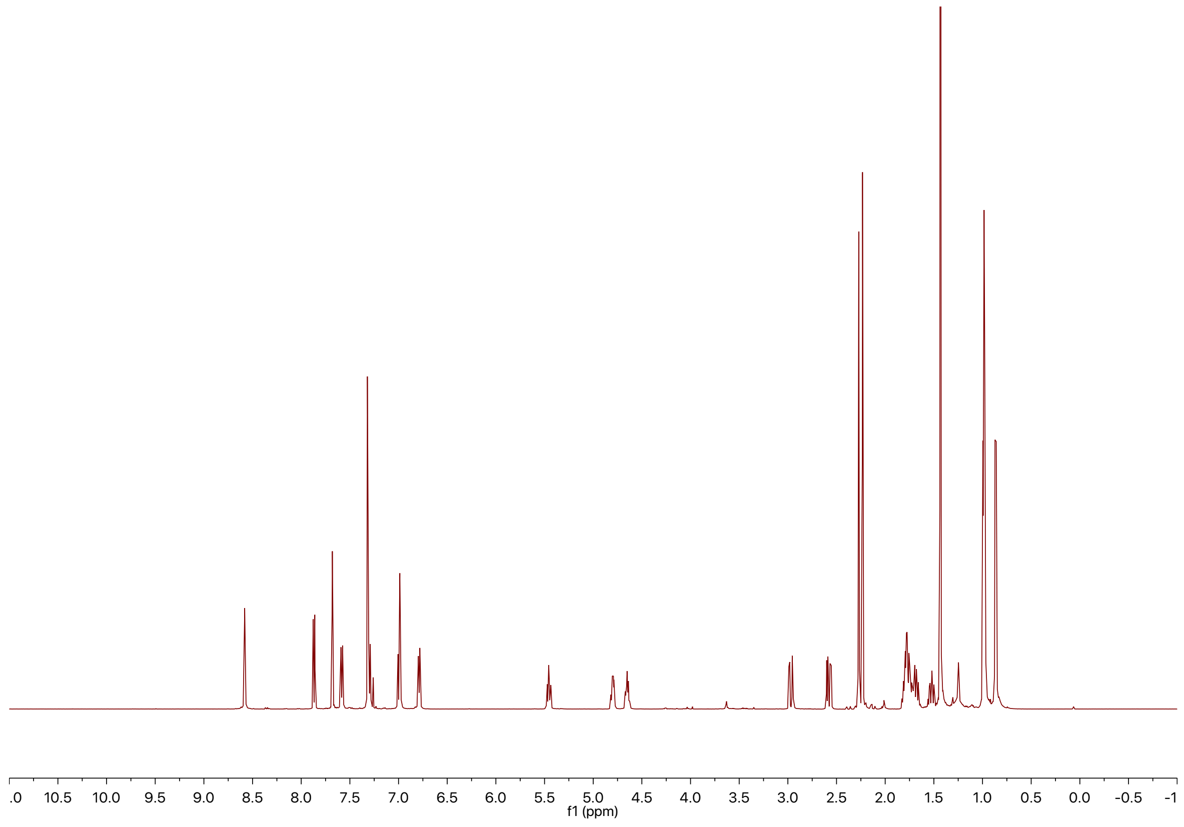

^{1}H-NMR (500 MHz, $CDCl_3$) of compound 28.

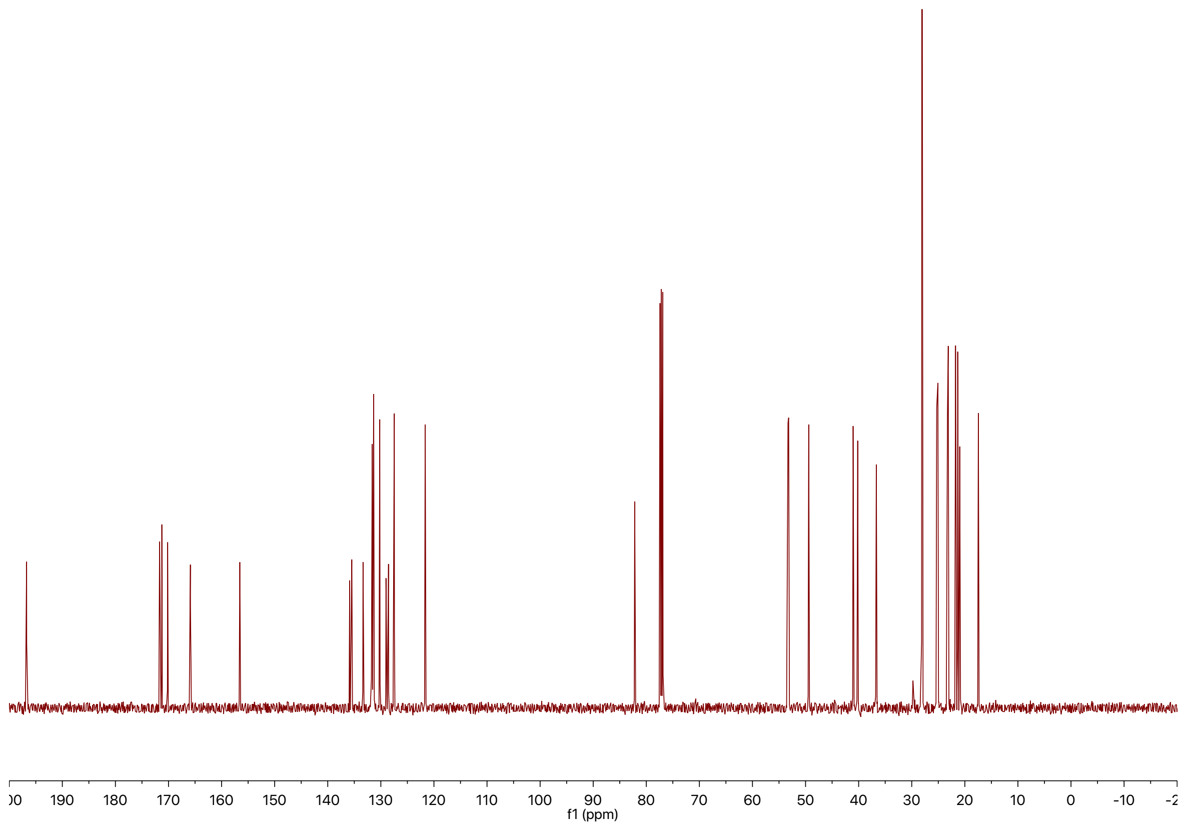

^{13}C-NMR (126 MHz, CDCl$_3$) of compound 28.

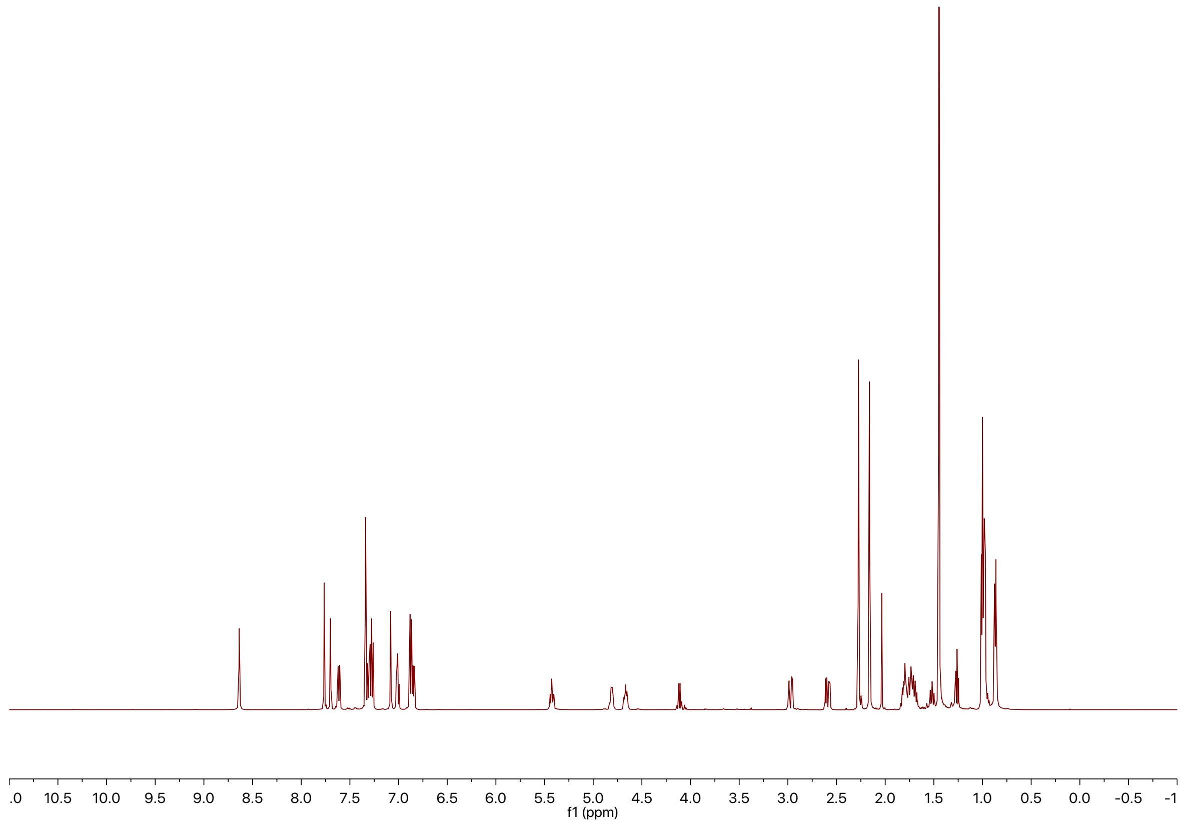

^{1}H-NMR (500 MHz, CDCl$_3$) of compound 29.

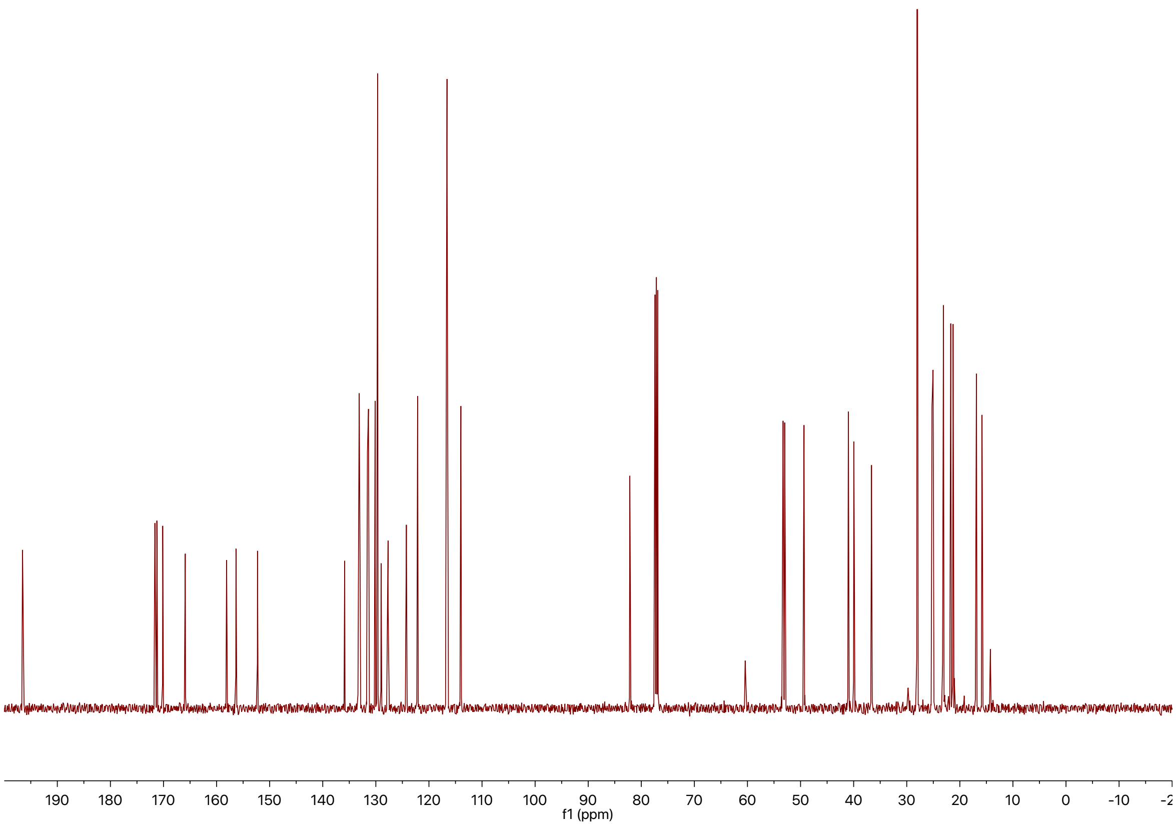

^{13}C-NMR (126 MHz, CDCl$_3$) of compound 29.

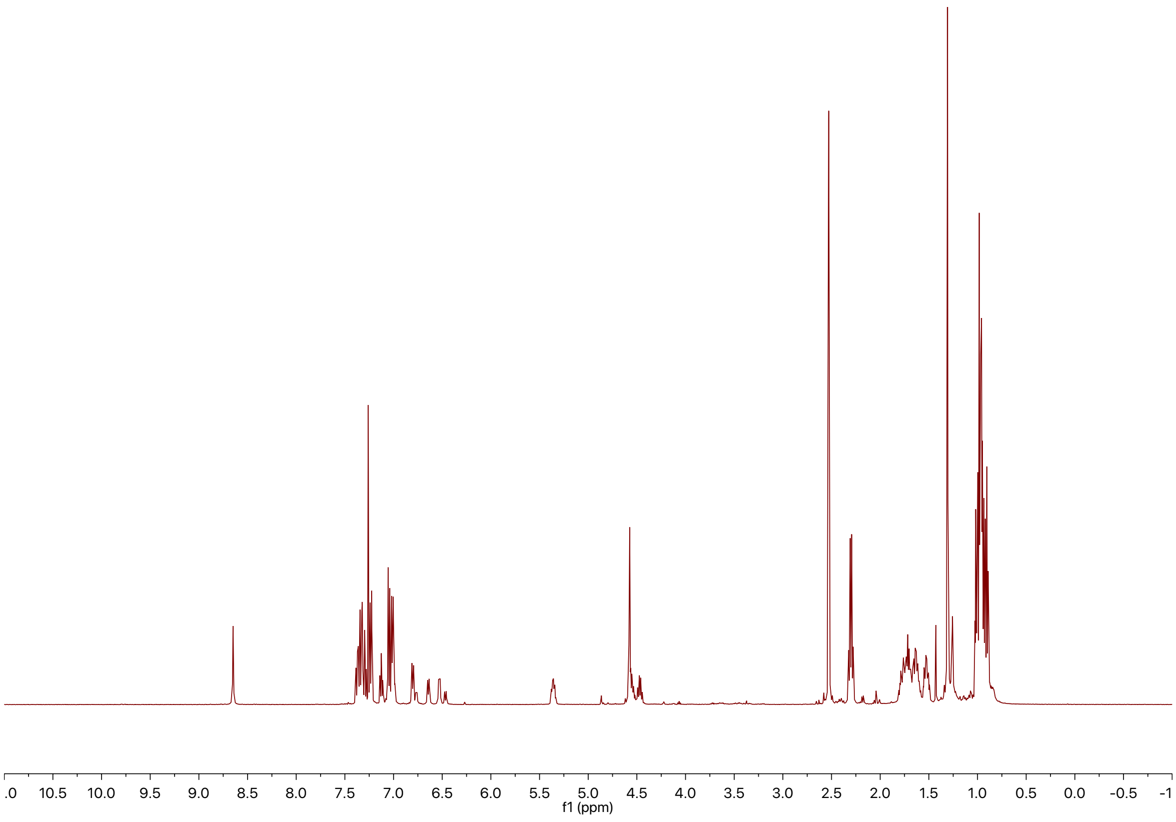

^{1}H-NMR (500 MHz, CDCl$_3$) of compound 30.

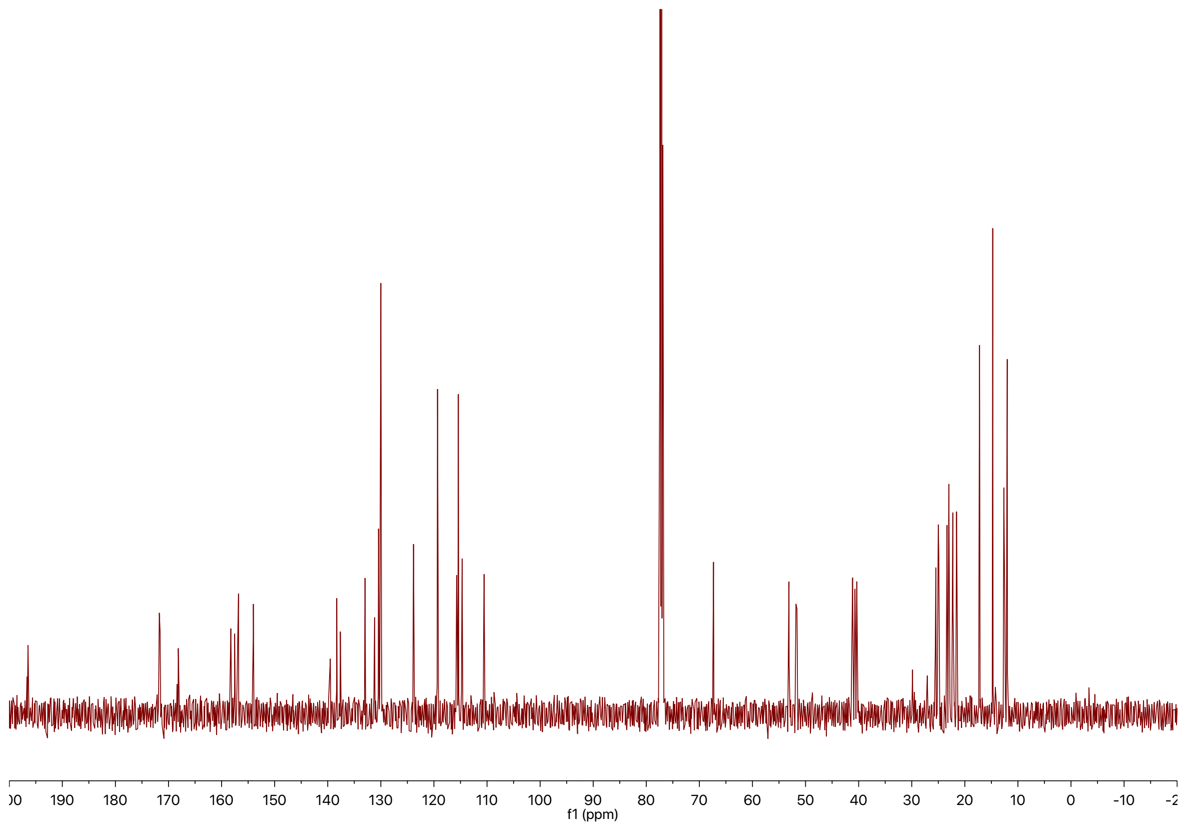

^{13}C-NMR (126 MHz, CDCl$_3$) of compound 30.

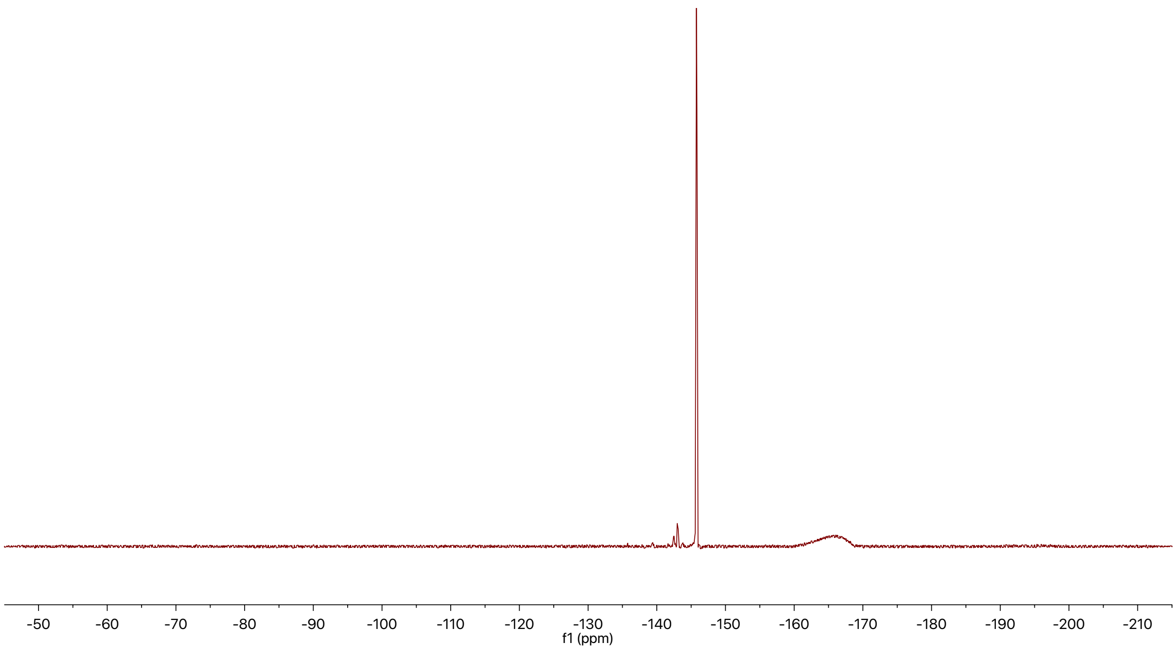

^{19}F-NMR (471 MHz, $CDCl_3$) of compound 30.

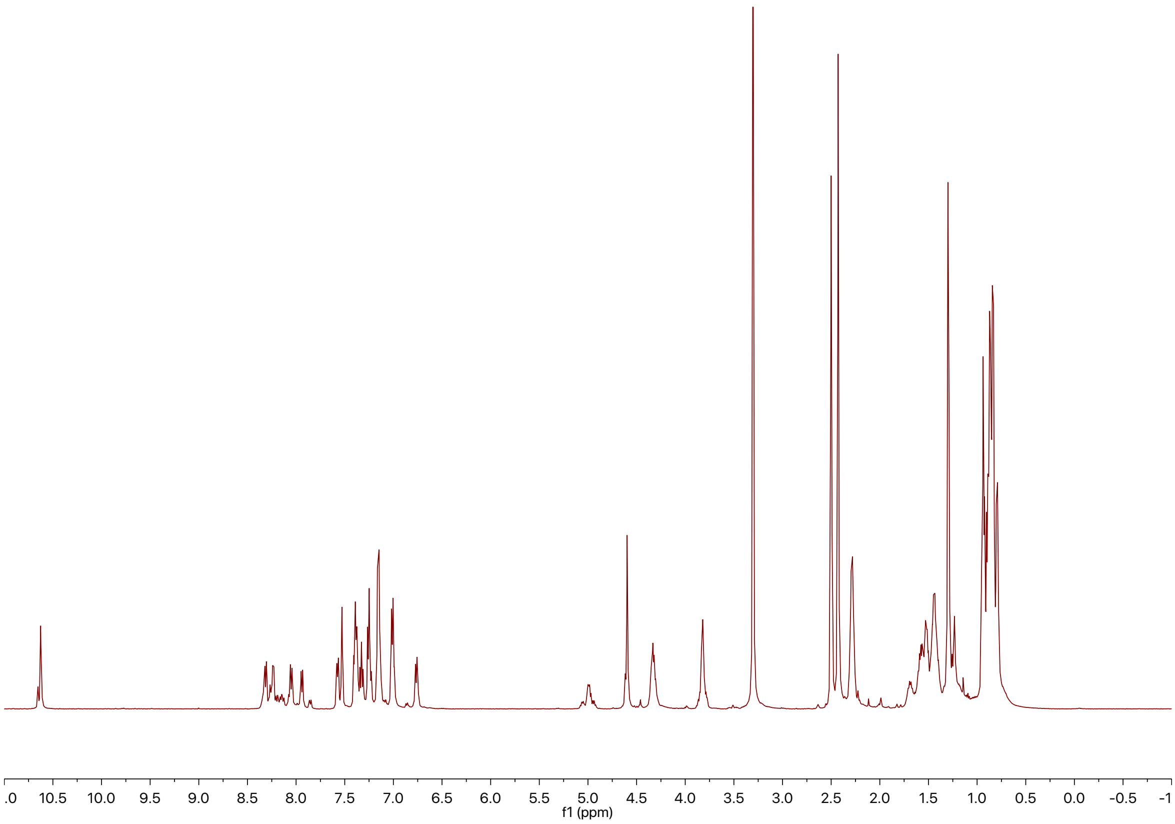

^{1}H-NMR (500 MHz, DMSO-d_6) of compound 31.

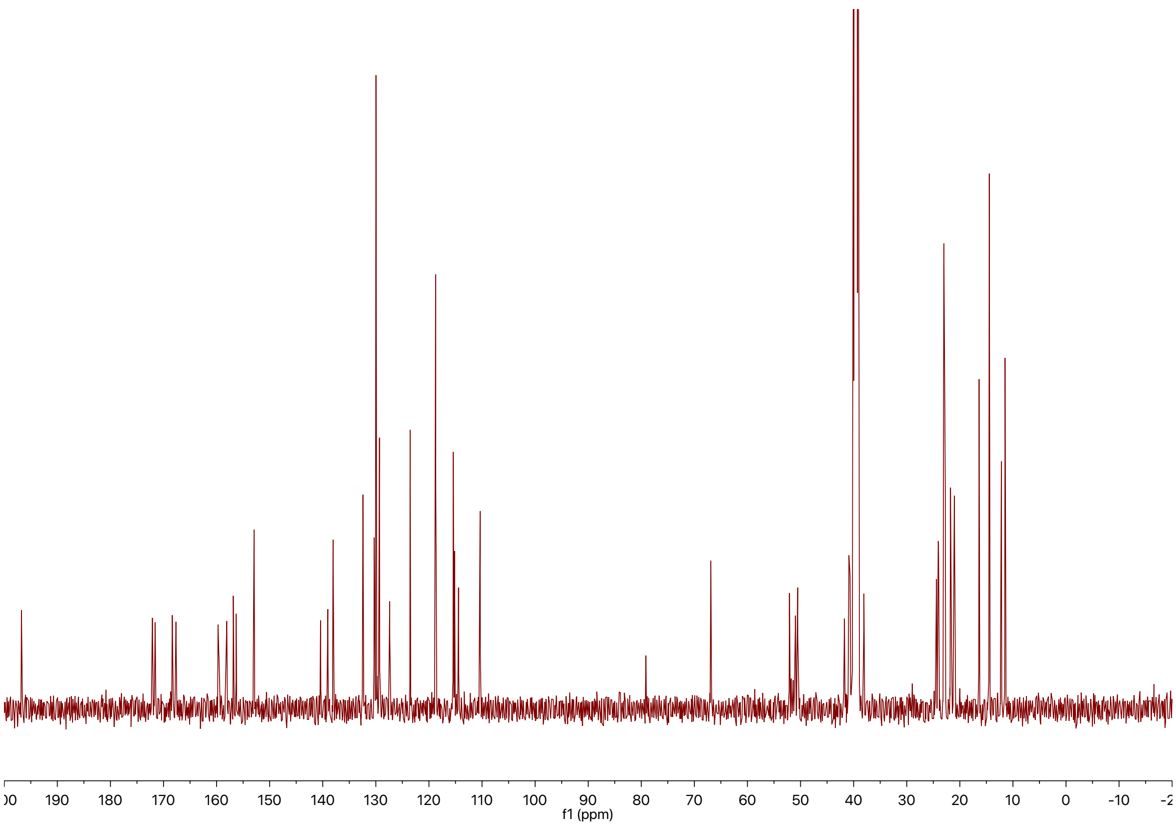

^{13}C-NMR (126 MHz, DMSO-d$_6$) of compound 31.

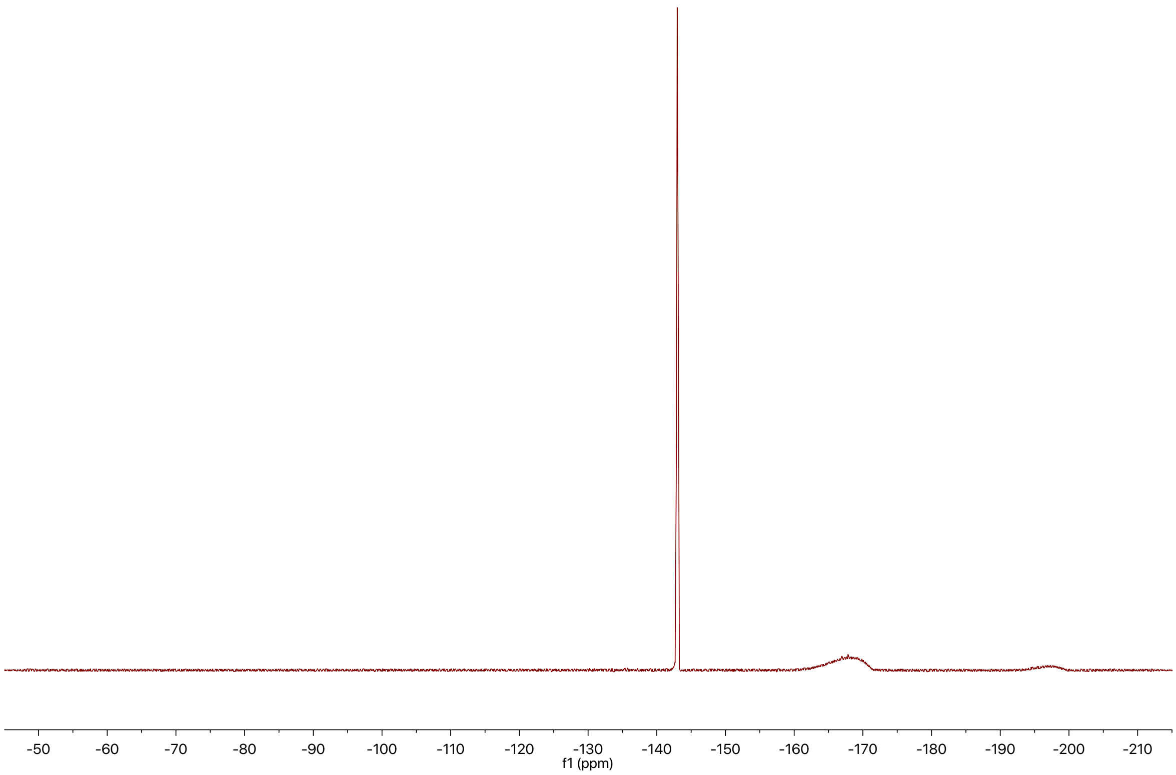

^{19}F-NMR (471 MHz, DMSO-d$_6$) of compound 31.

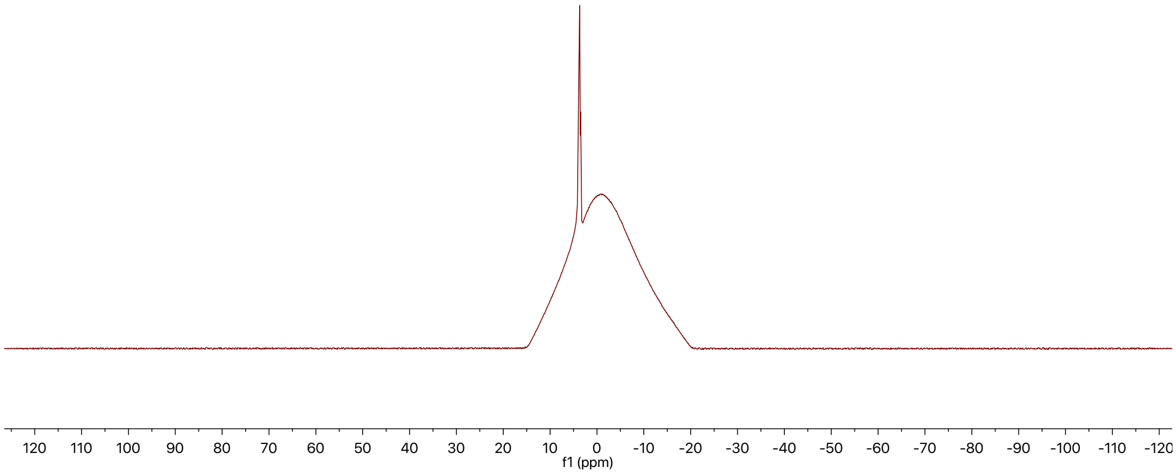

^{11}B-NMR (160 MHz, DMSO-d$_6$) of compound 31.

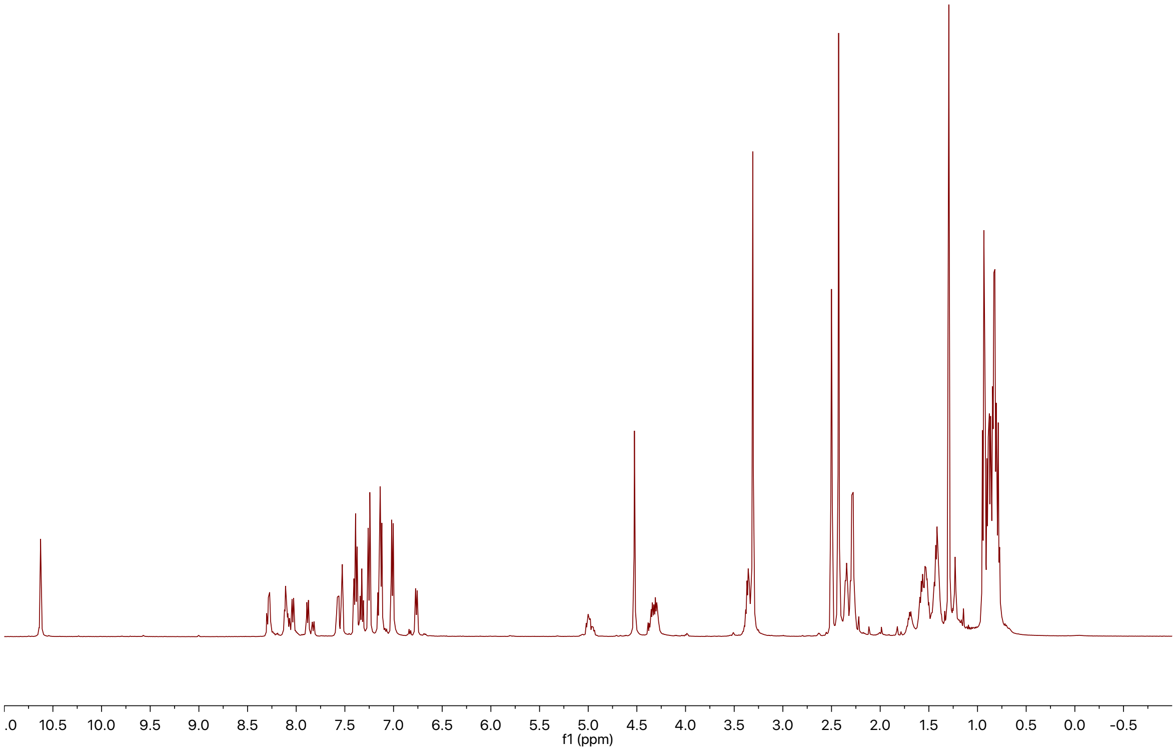

^{1}H-NMR (500 MHz, DMSO-d$_6$) of compound 32.

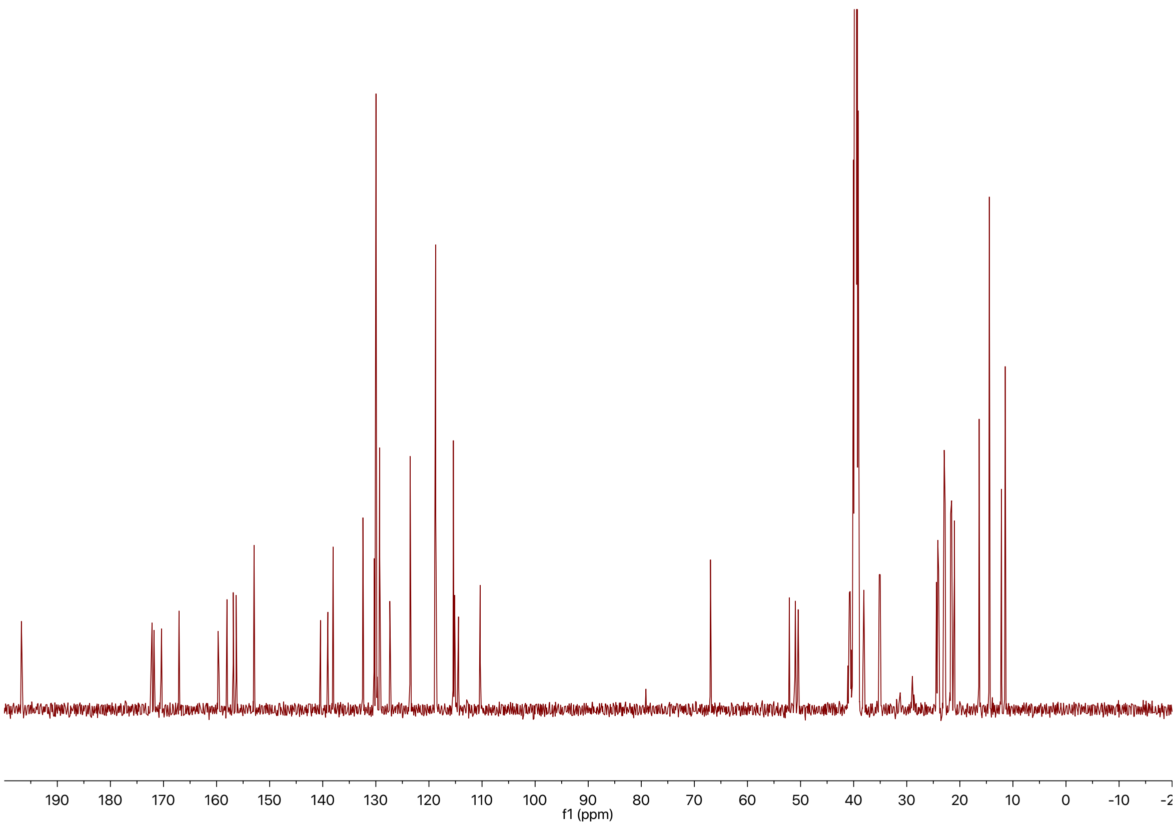

^{13}C-NMR (126 MHz, DMSO-d$_6$) of compound 32.

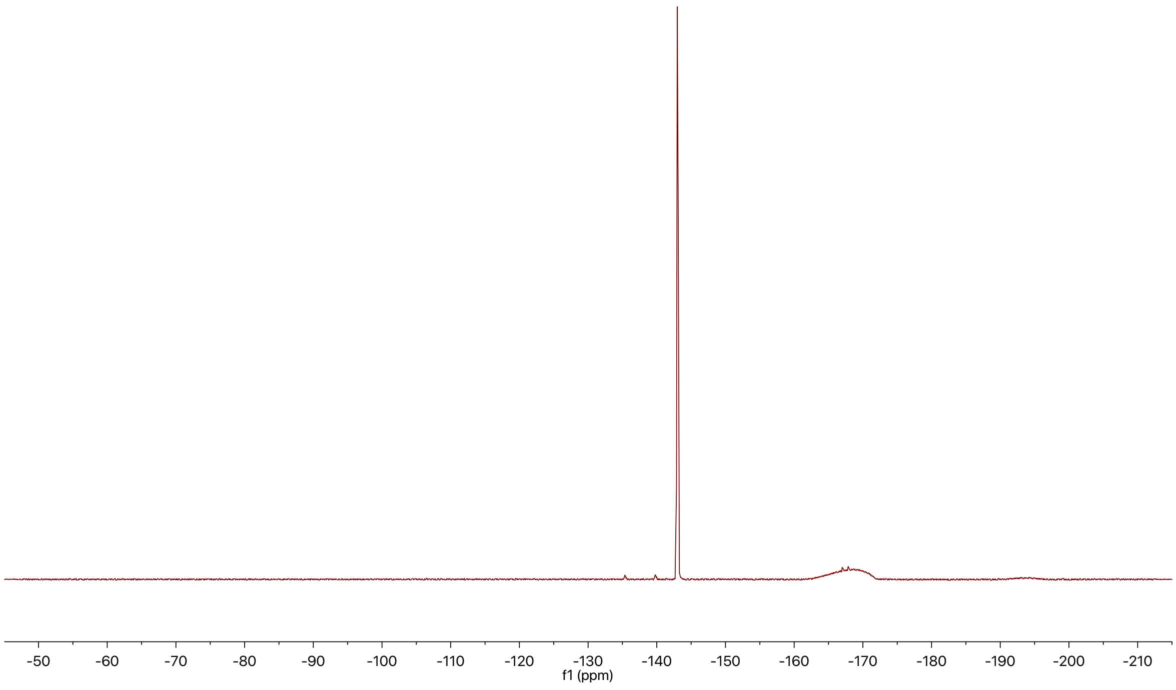

^{19}F-NMR (471 MHz, DMSO-d$_6$) of compound 32.

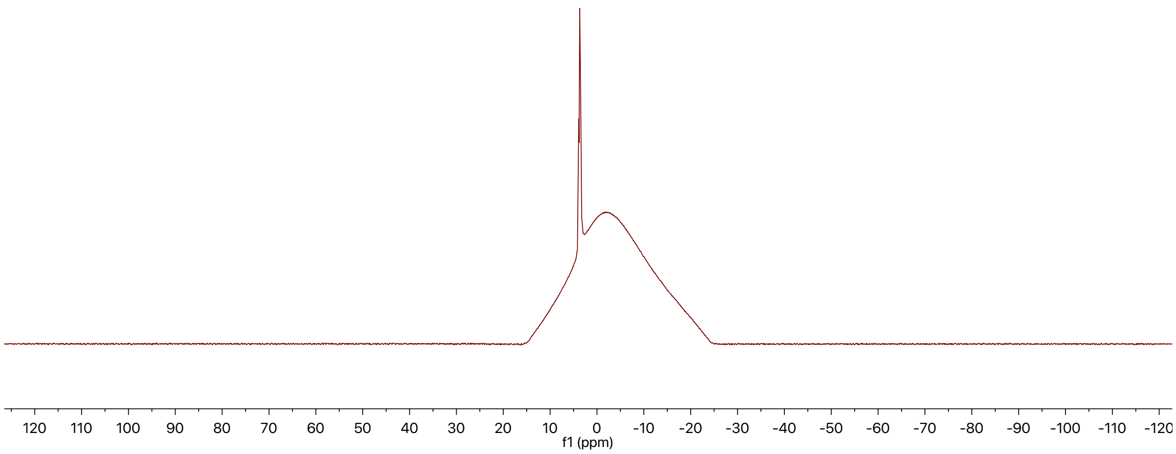

^{11}B-NMR (160 MHz, DMSO-d$_6$) of compound 32.

References

1. Braun, H. A.; Umbreen, S.; Groll, M.; Kuckelkorn, U.; Mlynarczuk, I.; Wigand, M. E.; Drung, I.; Kloetzel, P.-M.; Schmidt, B., *Journal of Biological Chemistry* **2005,** *280* (31), 28394-28401.
2. Stein, M. L.; Cui, H.; Beck, P.; Dubiella, C.; Voss, C.; Krüger, A.; Schmidt, B.; Groll, M., *Angewandte Chemie International Edition* **2014,** *53* (6), 1679-1683.
3. Dost, Z.; Atilgan, S.; Akkaya, E. U., *Tetrahedron* **2006,** *62* (36), 8484-8488.
